# Modelling of stellar convection

**DOI:** 10.1007/s41115-017-0001-9

**Published:** 2017-07-18

**Authors:** Friedrich Kupka, Herbert J. Muthsam

**Affiliations:** 1 Wolfgang Pauli Institute, Faculty of Mathematics, University of Vienna, Oskar-Morgenstern-Platz 1, 1090 Vienna, Austria; 2 Institut für Astrophysik, Universität Göttingen, Friedrich-Hund-Platz 1, 37077 Göttingen, Germany; 3 Faculty of Mathematics, University of Vienna, Oskar-Morgenstern-Platz 1, 1090 Vienna, Austria

**Keywords:** Convection, Stars, Modelling, Hydrodynamics, Numerics

## Abstract

The review considers the modelling process for stellar convection rather than specific astrophysical results. For achieving reasonable depth and length we deal with hydrodynamics only, omitting MHD. A historically oriented introduction offers first glimpses on the physics of stellar convection. Examination of its basic properties shows that two very different kinds of modelling keep being needed: low dimensional models (mixing length, Reynolds stress, etc.) and “full” 3D simulations. A list of affordable and not affordable tasks for the latter is given. Various low dimensional modelling approaches are put in a hierarchy and basic principles which they should respect are formulated. In 3D simulations of low Mach number convection the inclusion of then unimportant sound waves with their rapid time variation is numerically impossible. We describe a number of approaches where the Navier–Stokes equations are modified for their elimination (anelastic approximation, etc.). We then turn to working with the full Navier–Stokes equations and deal with numerical principles for faithful and efficient numerics. Spatial differentiation as well as time marching aspects are considered. A list of codes allows assessing the state of the art. An important recent development is the treatment of even the low Mach number problem *without* prior modification of the basic equation (obviating side effects) by specifically designed numerical methods. Finally, we review a number of important trends such as how to further develop low-dimensional models, how to use 3D models for that purpose, what effect recent hardware developments may have on 3D modelling, and others.

## Introduction and historical background

The goal of this review is to provide an overview on the subject of modelling stellar convection. It is supposed to be accessible not only to specialists on the subject, but to a wider readership including astrophysicists in general, students who would like to specialize in stellar astrophysics, and also to researchers from neighbouring fields such as geophysics, meteorology, and oceanography, who have an interest in convection from the viewpoint of their own fields.

A detailed introduction into the subject would easily lead to a book of several hundred pages. To keep the material manageable and thus the text more accessible we have made a specific selection of topics. The very recent review of Houdek and Dupret (2015) chiefly deals with the subject of the interaction between stellar convection and pulsation, but indeed contains an extended, detailed introduction into local and non-local mixing length models of convection that are frequently used in that field of research. An extended review on the capabilities of numerical simulations of convection at the surface of the Sun to reproduce a large set of different types of observations has been given in Nordlund et al. (2009). The large scale dynamics of the solar convection zone and numerical simulations of the deep, convective envelope of the Sun have been reviewed in Miesch (2005). Numerical simulations of turbulent convection in solar and stellar astrophysics have also been reviewed in Kupka (2009b). An introduction into the Reynolds stress approach to model convection in astrophysics and geophysics has been given in Canuto (2009).

Keeping these and further reviews on the subject in mind, we here focus on *computational aspects of the modelling of convection* which so far have found much less deliberation, in particular within the literature more accessible to astrophysicists. We are thus going to pay particular attention to computability in convection modelling and thus very naturally arrive at the necessity to describe both the advanced two-dimensional and three-dimensional *numerical simulation approach* as well as more idealized but also more affordable *models of convection*. The latter are not only based on the fundamental conservation laws, which are the foundation of numerical simulations of stellar convection, but introduce further hypotheses to keep the resulting mathematical model computationally more manageable.

Indeed, there are two different types of problems which make convection an inherently difficult topic. One class of problems is of a general, physical nature. The other ones relate to the specific modelling approach. We deal with both of them in this review. Following the intention of keeping this text accessible in terms of contents and volume we also exclude here the highly important separate topic of magnetohydrodynamics (MHD), since this opens a whole new range of specific problems. Even within the purely hydrodynamic context, some specific problems such as rotation–convection interaction had to be omitted, essential as they are, considering that this review has already about twice the default length applicable for the present series.

Before we summarize the specific topics we are going to deal with, we undertake a short tour on the history of the subject, as it provides a first overview on the methods and the problems occurring in dealing with a physical understanding and mathematical modelling of turbulent convection in stellar astrophysics.

### History

#### The beginning

The first encounter with stellar convection occurred, not surprisingly, in the case of the Sun, with the discovery of solar granulation, even if the physical background was naturally not properly recognized. Early sightings are due to Galileo and to Scheiner (Mitchell 1916 and Mitchell’s other articles of 1916 in that journal, see also Vaquero and Vázquez 2009, p. 143). Quite frequently, however, Herschel (1801) who reported a mottled structure of the whole solar surface is termed discoverer of solar granulation in the literature. The subject started to be pursued more vividly and also controversially in the 1860s (Bartholomew 1976). The photographic recording of solar granulation, the first one being due to Janssen (1878), clarified the subject of shapes of solar granules and remained the principal method of direct investigation for many decades to come.

These observations required a closer physical understanding. In 1904, Karl Schwarzschild raised the question whether the layering of the solar atmosphere was adiabatic, as was known to hold for the atmosphere of the Earth when strong up- and downflows prevail, or whether a then new concept of layering was appropriate which he dubbed radiative equilibrium (“Strahlungsgleichgewicht” in the original German version, Schwarzschild 1906). By comparing theoretical predictions with the observed degree of solar limb darkening he concluded that the solar atmosphere rather obeyed radiative equilibrium. This applies even from our present viewpoint in the sense that the convective flux, which is non-zero in the lower part of the solar atmosphere, is small compared to the radiative one.

Yet the very occurrence of granulation made it obvious that there had to be some source of mechanical energy able to stir it. For a period of time, the presence of flows in a rotating star, known through work of von Zeipel, Milne, and Eddington to necessarily occur, was considered a candidate. However, this turned out not to lead to a viable explanation when Eddington gave estimates of the velocities of these flows. Indeed, Eddington at first also considered convection not to be important for the physics of stars (cf. Eddington 1926), although he later on changed his opinion (Eddington 1938). The proper mechanism was figured out by Unsöld (1930). He noted that under conditions of the solar subsurface an (assume for the sake of discussion) descending parcel of material would not only have to do work in order to increase pressure and temperature, but that ionization work would be required as well. From such considerations Unsöld concluded that below the solar surface there was a convection zone to be expected and caused by the ionization of hydrogen. Another early line of thought had the stellar interior in mind and considered cases of energy sources strongly concentrated near the center of the star. For such a situation, convective zones were predicted to occur under appropriate circumstances by Biermann (1932). In that paper an analytical convection model was proposed, too: the mixing length theory of convection—which had initially not been developed to model solar convection and granulation.

#### “Classical” modelling

Once basic causes of solar granulation or rather solar and stellar convection zones had been identified in the early 1930s, theoreticians faced the problem to derive models of stellar and, in the first instance, of solar convection. Ideally, one would of course solve the Navier–Stokes (or Euler’s) equations of hydrodynamics, augmented with realistic microphysics and opacities, in some cases also elaborate radiative transfer calculations and other ingredients. Naturally that was out of question at a time when, at best, only mechanical calculators were available. As a consequence, models had been derived which were computationally (!) sufficiently simple to be, ultimately, incorporated into routine stellar structure or stellar atmosphere calculations. The mixing length paradigm, i.e., the concept of a characteristic length scale over which an ascending convective element survives, dissolving then and delivering its surplus energy, appears first in the work of Biermann (1932, 1942, 1948) and Siedentopf (1935). In her influential 1953 paper, Vitense developed a model where the essential transition region between radiative and convective zone was considered more accurately. In its improved form derived in her 1958 paper (Böhm-Vitense 1958), and in several variants thereof the mixing length model of stellar convection is still the most widely applied prescription in investigations of stellar structure and evolution (cf. Weiss et al. 2004). This is true despite of shortcomings, some of which were mentioned already in the original paper of Biermann (1932).

#### Non-local models and large scale numerical simulations

From a computational point of view the “classical” approach to modelling amounts to greatly reduce space dimensions from three to zero (in local models, where one has, at each point just to solve some nonlinear algebraic equation) or from three to one (in non-local models, where a depth variable is retained and ordinary differential equations result). Regarding the time variable, it may either be discarded as in mixing length theory or other local models of convection (Böhm-Vitense 1958; Canuto and Mazzitelli 1991; Canuto et al. 1996) or even some Reynolds stress models (Xiong 1985, 1986) or it may be retained, since that allows either finding a stationary solution more easily (Canuto and Dubovikov 1998) or making the theory applicable to pulsating stars, such as in non-local mixing length models (Gough 1977a, b) or other non-local models developed for this purpose (Kuhfuß 1986; Xiong et al. 1997).

The need for non-local models of convection was motivated by two physical phenomena:

1. There is “*overshooting*” of convective flow into neighbouring, “stable” layers and thus mixing between the two.
2. In pulsating stars *convection interacts with pulsation*. Convection may cause pulsation, drive it or damp it, or convection may be modulated by pulsation (see Houdek and Dupret 2015, for a review).

For a while the existence of *overshooting* had remained a controversial issue, as can be seen from the introductory summary in Marcus et al. (1983) who provided their own model for this process. Disagreement concerned both the modelling approach [the modal approach of Latour et al. (1976) is an example for a method “between analytical modelling and numerical simulation”] and the existence and importance of the phenomenon. The latter was settled later on (Andersen et al. 1990; Stothers and Chin 1991), but the disagreement on how to model it remained (cf. the comparison in Canuto 1993). As for *pulsating stars*, the classical formalism clearly required an extension. This development progressed from non-local mixing length models by Unno (1967) and Gough (1977a, b) to ever more advanced models including the Reynolds stress approach (Xiong et al. 1997) (see again Houdek and Dupret 2015). While the latter was pioneered by Xiong (1978), the most complete models intended for applications in astrophysics were published in a series of papers by Canuto (1992, 1993, 1997a), Canuto and Dubovikov (1998) and Canuto (1999). Nevertheless, most frequently used in practice are probably the non-local models of convection by Stellingwerf (1982) and Kuhfuß (1986) in studies of pulsating stars.

We note that in parallel to the developments in astrophysics the need for a non-local description of convection has also been motivated by laboratory experiments on the relation of the heat flux (which is measured by the Nusselt number) as a function of Rayleigh number in Rayleigh–Bénard convection. A transition between “soft” (Gaussian distributed) and “hard” turbulence in such flows was noted (Heslot et al. 1987) followed by the demonstration of the existence of a large-scale, coherent flow (Sano et al. 1989). These experiments are no longer compatible with the assumption of a simple scaling relation which underlies also the local mixing length model used in astrophysics (cf. its derivation in Spruit 1992). A much more complex set of scaling relations is required (see Grossmann and Lohse 2000; Stevens et al. 2013 for an attempt on a unifying theory) just to describe the interior of a convective zone as a function of fluid parameters (viscosity, heat conductivity) and system parameters (heat flux, temperature gradient). Such experiments have also been made for cases which exhibit what astrophysicists call overshooting and usually refer to what meteorologists and oceanographers describe as “entrainment”: field experiments and laboratory models of the convective, planetary boundary layer of the atmosphere of the Earth began in the late 1960s and early 1970s, respectively. Precision laboratory data on overshooting were obtained in water tank experiments (Deardorff and Willis 1985) followed by similarly accurate, direct measurements in the planetary boundary layer by probes (Hartmann et al. 1997). In both scenarios a convective zone is generated by heating from below and the convective layer is topped by an entrainment layer. Successful attempts to explain these data required the construction of either large scale numerical simulations or complex, non-local models of convection (cf. Canuto et al. 1994; Gryanik and Hartmann 2002 as examples). Although it is encouraging, if a model of convection successfully explains such data, this does not imply it also works in the case of stars: the physical boundary conditions play a crucial role for convective flows and the terrestrial and laboratory cases fundamentally differ in this respect from the stellar case which features no solid walls but can be subject to strong (and non-local) radiative losses and in general occurs and interacts with magnetic fields.

Given the long lasting need for models of convection which are physically more complete than the classical models, it is surprising that the latter are still in widespread use. However, as we discuss in Sect. 3, none of these more advanced models can achieve its wider region of applicability without additional assumptions and in most cases those cannot be obtained from first principles, the Navier–Stokes equations we introduce in Sect. 2, only. Moreover, such models usually introduce considerable computational and analytical complexity which until more recently, with the advent of space-based helio- and asteroseismology on the observational side and advanced numerical simulations on the theoretical side, were difficult to test. Furthermore, the traditional, integral properties of stars can easily be reproduced merely by adjusting free parameters of the classical models (for example, adjusting the mixing length to match the solar radius and luminosity, Gough and Weiss 1976).

However, the advent of computers has made it possible to solve, in principle, the “complete”, spatially multidimensional and time-dependent equations, often also for realistic microphysics and opacities and other physical ingredients as deemed necessary for the investigation at hand. Of course, in particular in early investigations, the space dimensionality had to be reduced to 2, microphysics had to be kept simple, and the like. But until today and for the foreseeable future it remains true that only a limited part of the relevant parameter range—in terms of achievable spatial resolution and computationally affordable time scales—is accessible in all astrophysically relevant cases.

Such numerical simulations request a style of work differing from the one applicable for the papers we have cited up to now. Whereas papers devoted to classical modelling are often authored by just one person, numerical simulations practically always require researchers working in teams.

If we consider *compressibility* and coverage of a few pressure scale heights as the hallmark of many convection problems in stellar physics, the first simulations aiming at understanding what might be going on in stellar convection, such as Graham (1975) and Latour et al. (1976), date from the mid-1970s.

Quite soon two rather different avenues of research were followed in the modelling community. In the *first* strand of research interest focussed on solar (and later on stellar) granulation. The two-dimensional simulations of Cloutman (1979) are probably the earliest work in this direction. Indeed it took only a short time until the basic properties of solar granulation could be reproduced, in the beginning invoking the anelastic approximation (see Nordlund 1982, 1984). In contrast to many contemporary papers this pioneering work was already based on numerical simulations in three dimensions. The topic has since evolved in an impressive manner and includes now investigations of spectral lines and chemical abundances, generation of waves, magnetic fields, interaction with the chromosphere, among others. We refer to the review provided in Nordlund et al. (2009). Since it has been completed, important new results have been achieved, regarding excitation of pressure waves, local generation of magnetic fields, high resolution simulations, and others. An impression on recent advances in such areas can be gained, e.g., from Kitiashvili et al. (2013). These simulations require, in particular, a detailed treatment of the radiative transfer equation, since they involve the modelling of layers of the Sun or a star which directly emit radiation into space, i.e., which are optically thin.

**Fig. 1:**
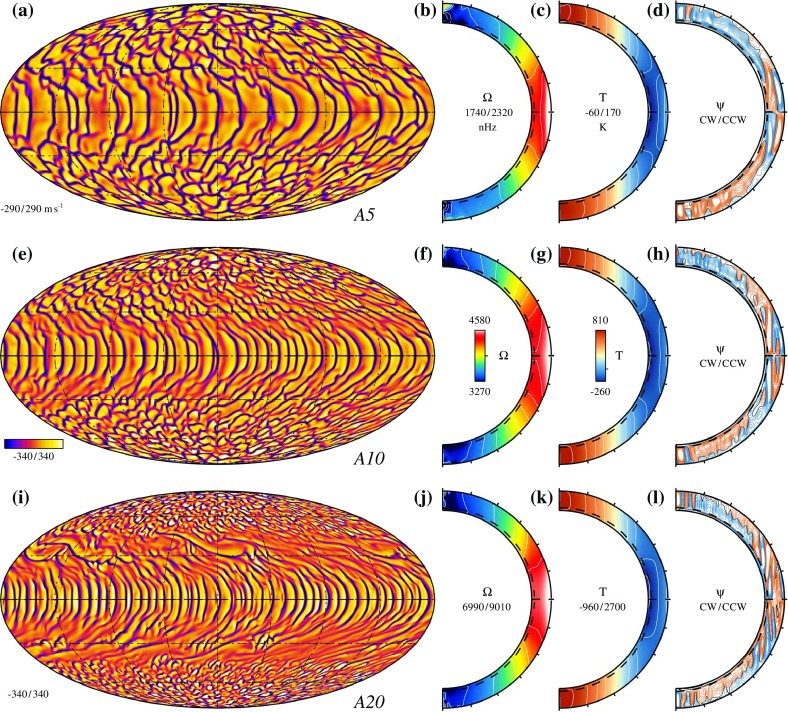
Convection cells and differential rotation in simulations of an F-type star (spherical shell essentially containing the convective zone; near surface regions not included in the calculations). Rotation rate increasing from *top* to *bottom*. *Left column* radial velocity of convective flows in the upper part of the computational domain. Following *columns* rotation rate, temperature deviation from *horizontal* mean and stream function of meridional flow. Differential rotation is clearly visible. Image reproduced with permission from Augustson et al. (2012), copyright by AAS

The *second* strand of investigations is directed more towards general properties of compressible convection and has stellar interiors in mind. As a consequence, it can treat radiative transfer in the diffusion approximation. Early work addressed modal expansions (Latour et al. 1976), but subsequently there was a trend towards using finite difference methods (Graham 1975; Hurlburt et al. 1984). In the course of time, the arsenal of numerical methods which were applied expanded considerably. Efforts to model a star as a whole or considering a spherical shell, where deep convection occurs, made it necessary to abandon Cartesian (rectangular) box geometry. Simulations based on spectral methods for spherical shells were hence developed (see, e.g., Gilman and Glatzmaier 1981). In the meantime, many investigations have addressed the question of convection under the influence of rotation (including dynamo action), the structure of the lower boundary of the convection zone in solar-like stars (tachocline), core convection in stars more massive than the Sun, and others. Figure 1 provides an example. A number of such recent advances are covered in Hanasoge et al. (2015).

A rather different form of convection, dubbed *semiconvection*, occurs during various evolutionary stages of massive stars. In an early paper Ledoux (1947) addressed the question of how a jump or gradient in molecular weight within a star would develop and derived what is today known as Ledoux criterion for convective instability. Semiconvection occurs when a star (at a certain depth) would be unstable against convection in the sense of the temperature gradient (Schwarzschild criterion) but stable considering the gradient in mean molecular weight which arises from dependency of chemical composition on depth (Ledoux criterion). In most of the literature the term semiconvection is restricted to the case where stability is predicted to hold according to the criterion of Ledoux (while instability is predicted by the Schwarzschild criterion), since both criteria coincide in the case of instability according to the Ledoux criterion which implies efficient mixing and no difference to the case without a gradient in chemical composition. Since application of one or the other of the criteria leads to different mixing in stellar models, calculations of stellar evolution are affected accordingly (see the critical discussion, also on computational issues, by Gabriel et al. 2014). In addition, thermal and “solute” (helium in stars; salt in the ocean) diffusivities play a role in the physics of the phenomena. Semiconvection gained interest with the advent of models of stellar evolution, specifically through a paper by Schwarzschild and Härm (1958). The interest was and is based on the material transport processes, and therefore of stellar mixing, thought to be effected by semiconvection. Unlike ordinary convection, where there existed and exists a standard recipe (in the form of mixing length theory) used in most stellar structure or evolution codes, such a recipe has not appeared for semiconvection (cf. Kippenhahn and Weigert 1994). Indeed, the semiconvection models which are used in studies of stellar physics are based on quite different physical pictures and arguments. Knowledge is in an early stage even considering the most basic physical picture. Likewise, numerical simulations referring to the astrophysical context have appeared only more recently. Early simulations which, however, remained somewhat isolated have been reported in Merryfield (1995) and Biello (2001). Only during the last few years activity has increased. For recent reviews on various aspects consult Zaussinger et al. (2013) and Garaud (2014) as well as the papers cited there (see also Zaussinger and Spruit 2013). The simulation parameters are far from stellar ones, but the ordering of the size of certain parameters is the same as in the true stellar case. Such simulations seem to consider a popular picture of semiconvection, many convecting layers, horizontally quite extended, vertically more or less stacked and divided by (more or less) diffusive interfaces (Spruit 1992), to be at least tenable, since it is possible to generate such layers also numerically with an, albeit judicious, choice of parameters.

### Consequences and choosing the subjects for this review

Considering the advances made during the last decades, why is convection still considered an “unsolved problem” in stellar astrophysics? The reason is that although numerical simulations can nowadays provide answers to many of the questions posed in this context, they cannot be applied to all the problems of stellar structure, evolution, and pulsation modelling: this is simply unaffordable. At the same time, no computationally affordable model can fully replace such simulations: uncertainties they introduce are tied to a whole range of physical approximations and assumptions that have to be made in those models, and to the poor robustness of their results and to the lack of universality of the model specific parameters.

Instead of providing an extended compilation of success and failure of the available modelling approaches, in this review we rather want to shed some more light on the origins of these difficulties and how some of these can be circumvented while others cannot. Our focus is hence—in a broad sense—on the *computational aspects of modelling stellar convection*. In particular, we want to provide an overview on which types of problems are accessible to numerical simulations now or in the foreseeable future and which ones are not. These questions are dealt with in Sect. 2 and motivate an overview in Sect. 3 on the state-of-the-art of convection modelling based on various types of (semi-) analytical models, since at least for a number of key applications they cannot be replaced by numerical simulations. Multidimensional modelling and the available techniques are reviewed in the next sections. The different variants of equations used in this context are reviewed in Sect. 4. There is a particular need for such a description, since a detailed summary of the available alternatives appears to be missing in the present literature, most clearly in the context of stellar astrophysics. We then proceed with an equally wanting review of the available numerical methods along with their strengths and weaknesses in Sect. 5. We conclude this review with a discussion on modelling specific limitations for the available approaches in Sect. 6. This section also addresses the hot topic of “parameter freeness” which is always around in discussions on the modelling of convection. Indeed, it had kept alive one of the most vivid and arguably one of the least helpful discussions in stellar astrophysics held during the last few decades. An effort is hence made to disentangle the various issues related to this subject. The limitations—including that one of claimed parameter freeness—are reviewed both from a more stringent and a more pragmatic point view and it is hoped that this can provide some more help for future work on the subject of stellar convection. To keep the material for this review within manageable size, the topic of interaction of convection with rotation and in particular with magnetic fields had mostly to be omitted. In a few cases we have provided further references to literature covering those topics, in particular with respect to the numerical methods for low-Mach-number flows discussed in Sect. 4. For a recent account of numerical magnetohydrodynamics, even in the more complex relativistic setting, we refer to Martí and Müller (2015).

## What is the difficulty with convection?

### The hydrodynamical equations and some solution strategies

What are the basic difficulties of modelling convection by analytical or numerical methods? To answer this question we first define the hydrodynamical equations which actually are just the conservation laws of mass, momentum, and energy of a fluid. In Sect. 4 we return to the numerical implications of their specific mathematical form.

The discovery of the hydrodynamical equations dates back to the eighteenth and nineteenth century. Analysis of the local dynamics of fluids eventually led to a set of partial differential equations which was proposed to govern the time development of a flow (see Batchelor 2000; Landau and Lifshitz 1963, e.g., for a derivation) for a fully compressible, heat conducting, single-component fluid. This was later on extended to include the forcing by magnetic fields. In the twentieth century the consistency of these equations with statistical mechanics and the limits of validity were demonstrated as well (Huang 1963; Hillebrandt and Kupka 2009, e.g.). A huge number of successful applications has established their status as fundamental equations of classical physics. Under their individual names they are known as continuity equation, Navier–Stokes equation (NSE), and energy equation and they describe the conservation of mass, momentum, and total energy. The term NSE is also assigned to the whole system of equations. In the classical, non-relativistic limit they read (we write $\partial _t f$ instead of $\partial f / \partial t$ for the partial derivative in time of a function *f* here and in the following):

1 $$\begin{aligned} \partial _t \rho +\,\mathrm{div}\,\left( \rho \varvec{u} \right)= & {} 0, \end{aligned}$$

2 $$\begin{aligned} \partial _t (\rho \varvec{u}) +\,\mathrm{div}\,\left( \rho (\varvec{u} \otimes \varvec{u}) \right)= & {} -\mathrm{div}\,{\varvec{\varPi }} - \rho \,\mathrm{grad}\,\varPhi , \end{aligned}$$

3 $$\begin{aligned} \partial _t \left( \rho E \right) +\,\mathrm{div}\,\left( (\rho E + p) \varvec{u} \right)= & {} q_{\mathrm{source}} +\,\mathrm{div}\,( {\varvec{\pi }} \varvec{u} ) - \rho \varvec{u}\cdot \,\mathrm{grad}\,\varPhi . \end{aligned}$$

where $q_{\mathrm{source}} = q_{\mathrm{rad}} + q_{\mathrm{cond}} + q_{\mathrm{nuc}}$ is the net production of internal energy in the fluid due to radiative heat exchange, $q_{\mathrm{rad}}$, thermal conduction, $q_{\mathrm{cond}}$, and nuclear processes, $q_{\mathrm{nuc}}$. At this stage of modelling they are functions of the independent variables of (1)–(3), the spatial location $\varvec{x}$ and time *t*. The same holds for the dependent variables of this system, $\rho , \varvec{\mu }= \rho \varvec{u}$, and $e = \rho E$, i.e., the densities of mass, momentum, and energy. We note that $\varvec{u} \otimes \varvec{u}$ is the dyadic product of the velocity $\varvec{u}$ with itself and $E=\varepsilon + \frac{1}{2}|\varvec{u}|^2$ is the total (sum of internal and kinetic) specific energy, each of them again functions of $\varvec{x}$ and *t*. The quantities $q_{\mathrm{rad}}$ and $q_{\mathrm{cond}}$ can be written in conservative form as $q_{\mathrm{rad}} = -\mathrm{div}\,\varvec{f}_{\mathrm{rad}}$ and $q_{\mathrm{cond}} = -\mathrm{div}\,\varvec{h}$, where $\varvec{f}_{\mathrm{rad}}$ is the radiative flux and $\varvec{h}$ the conductive heat flux whereas $q_{\mathrm{nuc}}$ remains as a local source term. Inside stars the mean free path of photons is about 2 cm and along this distance in radial direction the temperature changes are only of the order of ${\sim } 3\times 10^{-4}$ K (see Kippenhahn and Weigert 1994). This justifies a diffusion approximation for the radiative flux $\varvec{f}_{\mathrm{rad}}$ which avoids solving an additional equation for radiative transfer. Indeed, the diffusion approximation is exactly the local limit of that equation (Mihalas and Mihalas 1984) and it reads

4 $$\begin{aligned} \varvec{f}_{\mathrm{rad}} = -K_\mathrm{rad}\,\mathrm{grad}\,T, \end{aligned}$$

where $T=T(\rho ,\varepsilon ,\hbox {chemical composition})$ is the temperature and $K_\mathrm{rad}$ is the *radiative conductivity*,

5 $$\begin{aligned} K_\mathrm{rad} = \frac{4\,\mathrm{ac}\,T^3}{3 \kappa \rho } = \frac{16\sigma T^3}{3\kappa \rho }. \end{aligned}$$

The quantities a, c, and $\sigma$ are the radiation constant, vacuum speed of light, and Stefan–Boltzmann constant, while $\kappa$ is the *Rosseland mean opacity* (see Mihalas and Mihalas 1984; Kippenhahn and Weigert 1994). $\kappa$ is the specific cross-section of a gas for photons emitted and absorbed at local thermodynamical conditions (local thermal equilibrium) integrated over all frequencies (thus, $[\kappa ]= \mathrm{cm}^{2}\,\mathrm{g}^{-1}$ and $\kappa =\kappa (\rho ,T,\hbox {chemical composition})$, see Table 1). The heat flux due to collisions of particles can be accurately approximated by the diffusion law

6 $$\begin{aligned} \varvec{h} = -K_\mathrm{h}\,\mathrm{grad}\,T, \end{aligned}$$

where $K_\mathrm{h}$ is the heat conductivity (cf. Batchelor 2000). In stars, radiation is usually much more efficient for energy transport than heat conduction. This is essentially due to the large mean free path of photons in comparison with those of ions and electrons. Conditions of very high densities are the main exception. These are of particular importance for modelling the interior of compact stars (see Weiss et al. 2004). A derivation of the hydrodynamical equations for the case of special relativity, in particular for the more general case where the fluid flow is coupled to a radiation field, is given in Mihalas and Mihalas (1984). The latter also give a detailed discussion of the transition between classical Galilean relativity, a consistent treatment containing all terms of order $\mathrm{O}(v/\mathrm{c})$ for velocities *v* no longer much smaller than the speed of light *c*, and a completely co-variant treatment as obtained from general relativity. For an account of the theory of general relativistic flows see Lichnerowicz (1967), Misner et al. (1973) and Weinberg (1972), and further references given in Mihalas and Mihalas (1984). Numerical simulation codes used in astrophysical applications, in particular for the modelling of stellar convection, usually implement only a simplified set of equations when dealing with radiative transfer: typically, the fluid is assumed to have velocities $v \ll \,\mathrm{c}$, whence the intensity of light can be computed from the stationary limit of the radiative transfer equation (see Chap. 2 in Weiss et al. 2004). The solution of that equation allows the computation of $\varvec{f}_{\mathrm{rad}}$ and the radiative pressure $p_{\mathrm{rad}}$ to which we return below.

**Table 1:** Variables and parameters in the model equations used throughout this text

$\rho = \rho (\varvec{x},t)$	Gas density
$\varvec{u}=\varvec{u}(\varvec{x},t)$	Flow velocity
$\varvec{\mu }= \rho \varvec{u}= \varvec{\mu }(\varvec{x},t)$	Momentum density
$e = e(\varvec{x},t)$	Total energy density (sum of internal and kinetic one)
$E= e/\rho = E(\varvec{x},t)$	Specific total energy or total energy per unit of mass
$\varepsilon = E - \frac{1}{2}|\varvec{u}|^2 = \varepsilon (\varvec{x},t)$	Specific internal energy
$T=T(\rho ,\varepsilon ,\tilde{c})$	Temperature; for idealized microphysics also $T=T(\varvec{x},t)$
$p = p(\rho ,T,\tilde{c})$	Gas pressure; in Boussinesq approximation, $p=p(\varvec{x},t)$
*g*	Gravitational acceleration in direction $x_1$
$\varvec{g}$	Vector of gravitational acceleration, $\varvec{g}=(g,0,0)$
*I*	Identity matrix, entries of the unit tenor ***I***
$q_\mathrm{rad}$	Radiative heating rate
${\varvec{\sigma }}$	Viscous stress tensor ${\varvec{\pi }}$ for zero bulk viscosity $\zeta$
$\eta$	Shear (dynamical) viscosity (kinematic viscosity: $\nu =\eta / \rho$)
$K_\mathrm{rad} = K_\mathrm{rad}(\rho ,T,\tilde{c})$	Radiative conductivity; idealized also: $K_\mathrm{rad} = K_\mathrm{rad}(\varvec{x})$
$\chi _T = K/(c_p \rho ) = \chi (\rho ,T,\tilde{c})$	Radiative diffusivity; idealized also: $\chi _T = \chi _T(\varvec{x})$
$\kappa = \kappa (\rho ,T,\tilde{c})$	Opacity
$c_p=c_p(\rho ,T,\tilde{c})$	Specific heat under constant pressure
$\gamma$	Adiabatic index; general case: $\gamma =\gamma (\rho ,T,\tilde{c})$
$\varTheta$	Potential temperature
$\varvec{f}_{\mathrm{rad}} = \varvec{f}_{\mathrm{rad}}(\varvec{x},t)$	Radiative flux, its vertical component denoted by $F_\mathrm{rad}$

In this table, $\tilde{c}$ is used to indicate dependence on chemical composition

For numerical simulation of stellar convection Eqs. (1)–(6) are often augmented by a partial differential equation for the time evolution of the (divergence free) magnetic induction $\varvec{B}$ which also couples into the conservation laws for momentum and energy of the fluid, (2)–(3). A derivation of these equations and an introduction into magnetohydrodynamics can be found, for example, in Landau and Lifshitz (1984) and Biskamp (2008). Like the Navier–Stokes equations these can also be derived from the more general viewpoint of statistical physics (Montgomery and Tidman 1964) which allows recognizing their limits of applicability. For the remainder of this review we restrict ourselves to the classical, non-relativistic limit, (1)–(6), without a magnetic field. The radiative flux is obtained from the diffusion approximation (for the case of optically thin regions at stellar surfaces, which occurs in a few examples, it is assumed to be obtained from solving the stationary limit of the radiative transfer equation, see Weiss et al. 2004).

Returning to Eqs. (1)–(6) we note that internal forces per unit volume, given by the divergence of the pressure tensor $\varvec{\varPi }$, can be split into an isotropic part and an anisotropic one. The latter originates from viscous stresses. The isotropic part is just the mechanical pressure. It equals the gas pressure *p* from an equation of state, $p=p(\rho ,T,\hbox {chemical composition})$, if extra contributions arising from compressibility are collected into the *second* or *bulk* viscosity, $\zeta$ (see Batchelor 2000, for a detailed explanation). Thus,

7 $$\begin{aligned} {\varvec{\varPi }} = p {{\varvec{I}}} - {\varvec{\pi }}, \end{aligned}$$

where ${{\varvec{I}}}$ is the unit tensor with its components given by the Kronecker symbol $\delta _{ik}$ and the components of the tensor viscosity $\varvec{\pi }$ are given by (as for time *t* we abbreviate $\partial f / \partial x_j$ by $\partial _{x_j} f$)

8 $$\begin{aligned} \pi _{ik} = \eta \left( \partial _{x_k} u_i + \partial _{x_i} u_k - \frac{2}{3} \delta _{ik}\,\mathrm{div}\,\varvec{u} \right) + \zeta \delta _{ik}\,\mathrm{div}\,\varvec{u}. \end{aligned}$$

The dynamical viscosity $\eta$ is related to the kinematic viscosity $\nu$ by $\eta = \nu \rho$. Similar to $\kappa$ the quantities $\nu$ and $\zeta$ are functions of the thermodynamical variables $\rho , T$ (or $\varepsilon$), and chemical composition. Because $\varvec{\pi }$ is a tensor of rank two, a quantity such as $\varvec{\pi } \varvec{u}$ refers to the contraction of $\varvec{\pi }$ with the vector $\varvec{u}$. Note that (8) is linear in $\varvec{u}$ which is an approximation sufficiently accurate for essentially all fluids of interest to astrophysics. A detailed derivation of (7)–(8) is given in Batchelor (2000).

To model stellar conditions Eq. (2) has to be modified, since photons can transport a significant amount of momentum. This mechanical effect is represented by the radiation pressure tensor $P^{ij}$ (see Mihalas and Mihalas 1984) which is coupled into Eqs. (2)–(3). For isotropic radiation this problem can be simplified since in that case $P^{ij}$ can be written as the product of a scalar radiation pressure $p_{\mathrm{rad}}$ and the unit tensor ${{\varvec{I}}}$. Because the contribution of $\mathrm{div}\,(p_{\mathrm{rad}} {{\varvec{I}}})$ in (2) is additive, it is possible to absorb $p_{\mathrm{rad}}$ into the term for the gas pressure and treat it as part of the equation of state ($p_{\mathrm{rad}} = (1/3) a T^4$, see Weiss et al. 2004; Mihalas and Mihalas 1984). Such a procedure is exact at least as long as the diffusion approximation holds (Mihalas and Mihalas 1984) and allows retaining (2)–(3) in their standard form, a result of great importance for the modelling of stellar structure and evolution.

Finally, the gradient of the potential of external forces, $\phi$, has to be specified. The coupling of magnetic fields in magnetohydrodynamics as well as Coriolis forces in co–rotating coordinate systems *could* be considered as external forces. However, the *external potential itself* is usually just due to gravitation, where $\varvec{g} = -\mathrm{grad}\,\varPhi$. Equations (2)–(3) are thus rewritten as follows:

9 $$\begin{aligned}&\displaystyle \partial _t (\rho \varvec{u}) +\,\mathrm{div}\,\left( \rho (\varvec{u} \otimes \varvec{u}) \right) = -\mathrm{grad}\,p +\,\mathrm{div}\,\varvec{\pi } + \rho \varvec{g}, \end{aligned}$$

10 $$\begin{aligned}&\displaystyle \partial _t \left( \rho E \right) +\,\mathrm{div}\,\left( (\rho E + p) \varvec{u} \right) =\,\mathrm{div}\,( \varvec{\pi } \varvec{u} ) -\,\mathrm{div}\,\varvec{f}_\mathrm{rad} -\,\mathrm{div}\,\varvec{h} + \rho \varvec{u}\cdot \varvec{g} + q_\mathrm{nuc}. \nonumber \\ \end{aligned}$$

As implied by the discussion above, here *p* is usually meant as the sum of gas and radiation pressure and supposed to be given by the equation of state (cf. Sect. 6.3, 6.4, and 11 in Weiss et al. 2004). The gravitational acceleration $\varvec{g} = -\mathrm{grad}\,\varPhi$ is the solution of the Poisson equation $\mathrm{div}\,\mathrm{grad}\,\varPhi = 4\pi \mathrm{G}\,\rho$, where *G* is the gravitational constant. Since in all cases of interest here $q_\mathrm{nuc}$ is a function of local thermodynamic parameters ($\rho , T$, chemical composition, cf. Kippenhahn and Weigert 1994), we find that Eqs. (1), (9) and (10) together with (4)–(6) and (8) form a closed system of equations provided the material functions for $\kappa , K_\mathrm{h}, \nu , \zeta$, and the equation of state are known as well.

#### Solution strategies

While (1)–(10) have been known for a long time, analytical solutions or even just proofs of existence of a unique solution have remained restricted to rather limited, special cases. So how should we proceed to use the prognostic and diagnostic capabilities of these equations? One possibility is to construct approximate solutions by means of numerical methods. We focus on this approach in Sects. 4 and 5. An alternative to that is to approximate first the basic equations themselves. Famous examples for this approach are the Boussinesq approximation, stationary solutions, or the Stokes equations for viscous flows (see Batchelor 2000; Quarteroni and Valli 1994, e.g., and Sect. 4.3.1 below). The equations of stellar structure for the fully radiative case with no rotation (cf. Kippenhahn and Weigert 1994; Weiss et al. 2004) provide another example. The latter can also be obtained from models of the statistical properties of the flow (see below). In most cases simplified variants of the basic equations also require numerical methods for their solution. This is still advantageous as long as the computational costs of such approximate solutions are less demanding (cf. Sect. 5) than numerical solutions of (1)–(10).

Another possibility is the construction of a *different type of mathematical models* which *models properties of the hydrodynamical equations*. Staying most closely to the original equations are *model equations for volume averages* of (1)–(10). This is quite a natural approach, since also each astrophysical observation has a finite resolution in space, time, and energy, and in this sense refers to a volume average. Numerical solutions constructed with this goal in mind are usually termed *large eddy simulations (LES)*, although slightly different names are used to denote specific variants of it, for instance, iLES and VLES, abbreviations for implicit large eddy simulations and very large eddy simulations. The former refer to numerical solution methods for (1)–(10) where the numerical viscosity inherent to the solution scheme has the role of representing all effects operating on length scales smaller than the grid of spatial discretization used with the method. The latter implies that a significant amount of kinetic energy and dynamical interaction resides and occurs on such “unresolved” (“sub-grid”) length scales. An introduction to LES can be found in Pope (2000). In astrophysics it is common to make no clear distinction between such calculations and *direct numerical simulations (DNS)*. The latter actually refers to numerical approximations of (1)–(10) which *do not* assume any additional (statistical, physical) properties of the local volume average of the numerical solution to hold: all length scales of physical interest are hence resolved in such calculations, a requirement typically only fulfilled for mildly turbulent laboratory flows, viscous flows, and some idealized setups as used in numerical studies of the basic properties of (1)–(10). On the other hand, in *“hydrodynamical simulations”* of astrophysical objects it is often (implicitly) assumed that numerical viscosity of a scheme used to construct an approximate solution with moderate spatial resolution mimics the spatially and temporally averaged solution which is obtained with the same scheme at much higher resolution. Such simulations are actually iLES and hence clearly fall into the category of LES. We return to this subject further below.

Since an LES approach may be unaffordable or difficult to interpret or compare with observational data, further physical modelling is often invoked to derive mathematical model equations that are more manageable. A classical example are the standard equations of stellar structure and evolution (cf. Kippenhahn and Weigert 1994; Weiss et al. 2004) which account for both radiative and convective energy transport. These equations are actually mathematical models for *statistical properties* of Eqs. (1)–(10). More generally, *ensemble averaging* can be used to construct model equations, for instance, for some *mean density* ${\overline{\rho }}$ *and mean temperature* $\overline{T}$. The most common averages are one-point averages such as the Reynolds stress approach (see Sect. 3.3) which model statistical distributions that are functions of location and time only (cf. Lesieur 1997 and, in particular, Pope 2000). In turbulence modelling two-point averages are popular as well (see also Sect. 3.3). They deal with distribution functions that depend on the distance (or difference of locations) in addition to their spatial and temporal dependencies (Lesieur 1997; Pope 2000). The ensemble averaged approach requires additional, *closure assumptions* to construct complete sets of model equations.

Because the closure assumptions cannot be derived from (1)–(10) alone, alternatives have been sought for a long time. The coherent structures found in turbulent flows have been interpreted as a hint that geometrical properties may be taken as a guideline towards a new modelling approach (cf. Lumley 1989). When comparing such ambitious goals with more recent introductions into the subject of turbulent flows (Pope 2000; Tsinober 2009), progress achieved along this route is more modest than one might have expect one or two decades earlier (Lumley 1989). Interestingly, the analysis of structural properties of turbulent convection, for instance, has led to improved models on their statistical properties (Gryanik and Hartmann 2002; Gryanik et al. 2005), a nice example for why Tsinober (2009) has listed the idea that “statistics” and “structure” contrapose each other among the *common misconceptions about turbulent flows*.

To replace the NSE at the fundamental level by a discrete approach has already been proposed several decades ago (see the discussion in Lumley 1989), for instance, through the concept of cellular automata (cf. Wolf-Gladrow 2000). Today the *Lattice Boltzmann Methods (LBM)* have become a common tool particularly in engineering applications, but rather as a replacement of LES or direct numerical simulations instead of becoming an approach for more theoretical insights (for an introduction see, e.g., Succi 2001). For the study of fluids in astrophysics the *smooth particle hydrodynamics (SPH)* method has become the most successful among the discrete or particle-based concepts to model fluid dynamics (for a general introduction, see, e.g., Violeau 2012). SPH may be seen as a grid-free method to solve the NSE and in this sense again it is rather an alternative to (grid-based) numerical solutions of (1)–(10) and not an analytical tool. Until now these discrete methods, however, have found little interest for the modelling convection in astrophysics or geophysics. This is presumably because for many physical questions asked in this context there is not so much benefit from having very high local resolution at the extent of low resolution elsewhere. In Sects. 4 and 5 we discuss how also grid-based methods can deal with strong stratification, which may require high spatial resolution in a limited domain, and non-trivial boundary conditions as well.

A completely different goal has been suggested with the introduction of stochastic simulations of the multi-scale dynamics of turbulence (cf. Kerstein 2009). Contrary to LBM it does not evolve probability density functions, i.e., properties of particles, nor does it require the definition of kernels to model interactions as in SPH. Rather, stochastic maps for the evolution of individual particles are introduced which realize the interactions themselves. Clearly, at the largest scale and in three spatial dimensions, such an approach would become unaffordable. But it appears highly suitable to construct subgrid-scale models for conventional LES (see Kerstein 2009). This holds particularly, if the exchange of information (on composition, internal energy, etc.) is complex and crucial for the correct dynamics of the investigated system at large scales, for instance, in combusting flows.

### Spatial grids for numerical simulations of stellar convection

#### Constructing a grid for simulating the entire solar convection zone

How expensive would be a *hydrodynamical simulation* of an entire stellar convection zone or of a star as a whole? Let us first have a look at the spatial scales of interest in a star, specifically our Sun. Adapting these estimates to other stars is simple and does not change the basic arguments.

The solar radius has been measured to be $R_{\odot } \sim 695{,}500\,\mathrm{km}$ (cf. Brown and Christensen-Dalsgaard 1998 and Chap. 18.4c in Weiss et al. 2004). From helioseismology the lower boundary of the solar convection zone is $R / R_{\odot } \sim 0.713$ (Weiss et al. 2004, cf. Bahcall et al. 2005). The solar convection zone reaches the observable surface where $R / R_{\odot } \sim 1$. Its depth is hence about $D \sim 200{,}000\,\mathrm{km}$ considering overall measurement uncertainties (see also the comparison in Table 4 of Christensen-Dalsgaard et al. 1991). Differences of *D* of up to 10% are not important for most of the following. Another important length scale is given by solar granules which are observed to have typical sizes of about $L_\mathrm{g} \sim 1200 \ldots 1300\,\mathrm{km}$. Measurements made from such observations have spatial resolutions as small as ${\sim }35\,\mathrm{km}$ (cf. Spruit et al. 1990; Wöger et al. 2008). By comparison the highest resolution LES of solar convection in three dimensions, which has been published thus far (Muthsam et al. 2011), has achieved a horizontal and vertical resolution of $h \sim 3\,\mathrm{km}$. But this high resolution was limited to a region containing one granule and its immediate surroundings (Muthsam et al. 2011, regions further away were simulated at lower resolution).

This value of *h* is orders of magnitudes larger than the Kolmogorov scale $l_\mathrm{d}$ which quantifies length scales where viscous friction becomes important (cf. Lesieur 1997; Pope 2000). $l_\mathrm{d}$ can be constructed with dimensional arguments from the kinetic energy dissipation rate $\epsilon$ and the kinematic viscosity as $l_\mathrm{d} = (\nu ^3 \epsilon ^{-1})^{1/4}$. Due to the conservative form of (1)–(10) production of kinetic energy has to equal its dissipation. For the bulk of the solar convection, where most of the energy transport is by convection and no net energy is produced locally in the same region, one can estimate $\epsilon$ from the energy flux through the convection zone (see Canuto 2009) as $\epsilon \sim L_{\odot } / M_{\odot } \approx 1.9335\,\mathrm{cm}^2\,\mathrm{s}^{-3} \sim \,\mathrm{O}(1)\,\mathrm{cm}^2\,\mathrm{s}^{-3}$ using standard values for solar luminosity and mass (see Chap. 18.4c in Weiss et al. 2004, and references therein). From solar models (e.g., Stix 1989; Weiss et al. 2004) temperature and density as functions of radius can be estimated. With Chapman’s result (1954) on kinematic viscosity of fully ionized gases, $\nu = 1.2 \times 10^{-16} T^{5/2} \rho ^{-1}\,\mathrm{cm}^2\,\mathrm{s}^{-1}, \nu$ is found in the range of 0.25–$5\,\mathrm{cm}^{2}\,\mathrm{s}^{-1}$, whence $l_\mathrm{d} \approx 1\,\mathrm{cm}$ throughout most of the solar convection zone (Canuto 2009). Near the solar surface the fluid becomes partially ionized. From Tables 1 and 2 in Cowley (1990) $\nu$ is found in the range of 145 cm$^2$ s$^{-1}$ to 1740 cm$^2$ s$^{-1}$ for *T* between 19,400 and 5660 K. Thus, just at and underneath the solar surface, $\nu \sim 10^3\,\mathrm{cm}^2\,\mathrm{s}^{-1}$ whence $l_\mathrm{d} \approx 1\,\mathrm{m} \ldots 2\,\mathrm{m}$ in the top layers of the solar convection zone.

Another length scale of interest is the thickness of radiative thermal boundary layers. We note that the designation “boundary layer” strictly speaking refers to the geophysical and laboratory scenario where heat enters the system through a solid horizontal plate (a scenario also used in idealized numerical simulations with astrophysical applications such as Hurlburt et al. 1994; Muthsam et al. 1995, 1999; Chan and Sofia 1996, e.g.). However, the same length scale $\delta$ is equally important for convection zones without “solid boundaries” in the vertical direction, since it describes the length scale below which temperature fluctuations are quickly smoothed out by radiative transfer (in the diffusion approximation) and we use this notion for its definition.^1^ It is thus the length scale to be resolved for an accurate computation of the thermal structure and radiative cooling processes. Taking the diffusivity of momentum described by $\nu$ as a reference, the Prandtl number $\mathrm{Pr} = \nu / \chi$ for the solar convection zone is in the range of $10^{-9}$ near the surface and increases inwards to about $10^{-7}$ (see Sect. 4.1 in Kupka 2009b, for details). Since for diffusion equations one can relate the mean free path *l*, the diffusivity *d*, and the collision time *t* through $t \approx l^2/d$ to each other (cf. Chap. 18.3a in Weiss et al. 2004), we can compare heat and momentum transport on the same reference time scale to each other. This may be, for instance, the time scale of convective transport over the largest length scales appearing in a certain depth of the convective zone (the size of granules, e.g.). During this amount of time heat diffusion transports material over a distance $\delta = \sqrt{\chi t_\mathrm{ref}}$. Since this choice for $t_\mathrm{ref}$ is also the time scale during which kinetic energy is dissipated (cf. Sects. 1 and 2 in Canuto 2009), we may use it to compare $\delta ^2$ with $l_\mathrm{d}^2$ and obtain $\delta ^2 / l_\mathrm{d}^2 \sim \chi / \nu =\,\mathrm{Pr}^{-1}$ (note that the time scale cancels out in this ratio). Thus, $\delta \sim \,\mathrm{Pr}^{-1/2}\,l_\mathrm{d}$ and for the lower part of the solar convection zone, $\delta _\mathrm{min} \sim 30\,\mathrm{m}$ while^2^ near the surface, $\delta _\mathrm{surface} \sim 30\,\mathrm{km} \ldots 60\,\mathrm{km}$. We note that near the solar surface, $\chi$ varies rapidly and in the solar photosphere the fluid becomes optically thin, but for a rough estimate of scales these approximations are sufficient. Indeed, the steepest gradients in the solar photosphere are found just were the fluid has already become optically thick and $\delta _\mathrm{surface}$ is thus closely related to the resolution used in LES of solar (stellar) surface convection, as we discuss below.

Evidently, it is hopeless trying to resolve the Kolmogorov scale, as required by a DNS, in a simulation which encompasses the whole solar convection zone: this would require about $N_\mathrm{r}(l_\mathrm{d}) \sim 2\times 10^{10}$ grid points in radial direction. With *D* and $R_{\odot }$ the solar convection zone is estimated to have a volume of $V \sim 9\times 10^{32}\,\mathrm{cm}^3$ which yields the number of required grid points, $N_\mathrm{tot}(l_\mathrm{d}) \sim 9\times 10^{32}$ (a simulation of the total solar volume exceeds this by less than 60%). Even before taking time integration into account, it is clear that such a calculation is a pointless issue on semiconductor based computers (irrespectively of whether a DNS is considered to be really necessary or not).

The odds are not much better for a simulation to resolve $\delta$ throughout the solar convection zone, since this requires a grid with $h = \min (\delta ) = \delta _\mathrm{min} \sim 30~\mathrm{m}$. That resolution is more “coarse” by a factor of 3000 which reduces the number of (roughly equidistantly spaced) grid points by $2.7\times 10^{10}$ to $N_\mathrm{tot}(\delta _\mathrm{min}) \sim 3.3\times 10^{22}$ for the solar convective shell. If we take ten double words to hold the five basic variables $\rho , \mu , e$ and the derived quantities $\varvec{u}, T, P$ for each grid cell volume, we obtain a total of 80 bytes as a minimum storage requirement per grid point (most hydrodynamical simulation codes require at least five to ten times that amount of memory). We can thus estimate a single snapshot to take 2.6 YB (Yottabyte, $1\,\mathrm{YB} = 10^{24}$ bytes) of main memory. This exceeds current supercomputer memory capacities by seven to eight orders of magnitude.

As a minimum requirement for an LES of the entire solar convection zone one would like to resolve at least the radiative cooling layer near the surface. This is necessary to represent the radiative cooling of gas at the solar surface within the simulation on the computational grid, as it is the physical process which drives the entire convective flow (cf. Spruit et al. 1990; Stein and Nordlund 1998). From our previous estimates we would expect that $h = \min (\delta _\mathrm{surface})/2 \sim 15\,\mathrm{km}$, because two grid points are the minimum to represent a feature in a solution and thus catch a property of interest related to it.

Indeed, $h \lesssim 15\,\mathrm{km}$ is the standard resolution of LES of solar granulation (see Table 3 in Beeck et al. 2012 and Sect. 4 of Kupka 2009a). Then, radiative cooling is properly modelled, i.e., at $h = 12\,\mathrm{km}$ the horizontal average of the vertical radiative flux $F_\mathrm{rad}$ is found to be smooth in a one-dimensional numerical experiment even though its negative divergence, the cooling rate $q_\mathrm{rad}$, would require a ten times higher resolution (Nordlund and Stein 1991). From the same calculations *T* is found to change by up to $190\,\mathrm{K}\,\mathrm{km}^{-1}$ which at 10,000 K and at $h = 12\,\mathrm{km}$ is a relative change of ${\sim }23\%$ between two grid cells. In actual LES of about that resolution (Stein and Nordlund 1998) *T* changes vertically on average only by up to $30\,\mathrm{K}\,\mathrm{km}^{-1}$ and in hot upflows by up to $100\,\mathrm{K}\,\mathrm{km}^{-1}$, a relative change less than 4% (or up to 12% where it is steepest). As the maximum mean superadiabatic gradient $\partial \ln T / \partial \ln P$ is found to be about 2 / 3 in these simulations (Rosenthal et al. 1999), the corresponding changes in *P* between grid cells are up to 6% on average and 18% at most. Hence, the basic thermodynamical variables are resolved on such a grid despite opacity $\kappa$ and thus optical depth $\tau$ and the cooling rate $q_\mathrm{rad}$ vary more rapidly by up to an order of magnitude due to the extreme temperature sensitivity of opacity in the region of interest (Nordlund and Stein 1991).

The actual resolution demands are somewhat higher than the simple estimate of $h \approx 15\,\mathrm{km}$ which is anyway limited to regions where the approximation of radiative diffusion holds (optical depths $\tau \gtrsim 10$). Since it appears to be sufficient in practice (cf. Beeck et al. 2012; Kupka 2009b) we use it for the following estimate. By lowering the number of grid points along the solar convection zone in radial direction to $N_\mathrm{r}(\delta _\mathrm{surface}) \sim 13{,}000$ we obtain that $N_\mathrm{tot}(\delta _\mathrm{surface}) {\sim }2.7\times 10^{14}$ or ${\sim }4.3\times 10^{14}$ for the Sun as a whole. For grids of this size it is becoming possible to store one snapshot of the basic variables in the main memory of a supercomputer, if the entire capacity of the machine were used for this purpose: a minimum amount of 80 bytes per grid cell requires 21.6 PB (or 34.4 PB, respectively), although production codes are more likely to require several 100 PB – 1 EB for such applications.

One can further reduce this resolution demand by taking into account that the pressure scale height $H_p$ drastically increases from the top to the bottom of the convection zone. We recall its definition, $H_p = -(\partial r / \partial \ln p) \approx p/(\rho g)$, where *g* is the (negative) radial component of $\varvec{g}$. In the stationary case, for spherical symmetry and negligibly small turbulent pressure the two expressions are identical for $r > 0$, so the second one is almost always used to define $H_p$ (cf. Kippenhahn and Weigert 1994; Weiss et al. 2004). If we require the relative accuracy of *p* to be the same throughout the convection zone, it suffices to keep the number of grid points per pressure scale height constant or, simply, scale the radial grid spacing with $H_p$. Except for the surface layers, where $\mathrm{grad}\,T$ can become very steep, this should ensure comparable relative accuracy of numerical derivatives for all basic thermodynamic quantities throughout the solar convection zone under the assumption that microphysical data (equation of state, opacity, etc.) are also known to the same relative accuracy. With $h \approx 15\,\mathrm{km}$ and $H_p \approx 150\,\mathrm{km}$ at the solar surface and for a total depth of solar convection zone of about 20 pressure scale heights (cf. Stix 1989; Weiss et al. 2004, and references therein) one can thus reduce $N_\mathrm{r}(\delta _\mathrm{surface})$ to an optimistic $N_\mathrm{r}(\mathrm{minimal}) \sim 200$. Since pressure stratification occurs only vertically, the net gain is a factor of 65, whence $N_\mathrm{tot}(\mathrm{minimal}) \sim 4.5\times 10^{12}$. Because the solar interior region has its own resolution demands due to the temperature sensitivity of nuclear fusion (Kippenhahn and Weigert 1994; Weiss et al. 2004), there can be no further gain for models of the entire Sun. For simplicity we assume the overall scaling factor in comparison with models of the convective shell to be the same and obtain $N_\mathrm{tot}(\mathrm{minimal}) \sim 7.2\times 10^{12}$ for models of the Sun. Memory requirements as low as 0.33–0.5 PB are within reach of the current, largest supercomputers. Again, realistic production codes may require something like 2–20 PB for such a model. Such demands may mislead one to consider LES of that kind suitable for next generation supercomputers, but there are further, severe constraints ahead. As we discuss in Sect. 2.3 it is the necessary number of time steps which continues to prohibit this class of simulations for the Sun for quite a few years to come. Solar simulations hence have to be limited to smaller segments or boxes as domains which include the solar (and in general stellar) surface or alternatively to spherical shells excluding the surface layers. A list including also exceptions from these limitations and a summary of computational complexity are given in Sect. 2.6.

#### Computing grids for affordable problems

If one sufficiently limits the extent of the domain of the numerical simulation, its computational demands can be brought into the range of affordable problems. The construction of computing grids which are affordable on existing hardware is hence the first step to make LES of convection in stars viable. Two important ideas to do this have frequently been used and they can readily be generalized:

1. The *box-in-a-star* approach suggests to perform the numerical simulation only in a spatially limited domain the location of which is usually considered to be close to the surface of a star and include those layers emitting photons directly into space, i.e., the *photosphere* of the star. This is not in any way a necessary condition, as the same simulation technique can also be applied for layers located completely inside a star. But the *“3D stellar atmospheres”* are certainly the most prominent application of this kind since the pioneering work of Nordlund (1982). The main challenge in this approach is to define suitable *boundary conditions* to allow for an in- and outflow of energy. Usually, this is also assumed for mass and momentum in which case the boundary conditions are called *open*. Due to the strong stratification found near the observable surface of most stars the size of the dominant flow structure is small enough such that a Cartesian geometry can be assumed (cf. Stein and Nordlund 1998), except for the case of bright giants and supergiants which require a different way of modelling (see below). The gravitational acceleration is hence approximated to be constant along the vertical direction which coincides with the radial one of the actual star and the curvature of the star in that domain is neglected. This motivates the introduction of periodic boundary conditions in the plane orthogonal to the direction of gravity. The simulation domain has to be defined sufficiently wide for this approach to work (cf. Appendix A of Robinson et al. 2003). The choice of vertical boundary conditions is more subtle and it may depend on the type of star under investigation. A recent comparison of the impact of boundary conditions on numerical simulations of the solar surface can be found in Grimm-Strele et al. (2015a). A large amount of astrophysical literature gives credit to this approach. A detailed account for just the case of the Sun has already been subject to a review on its own (Nordlund et al. 2009). We note here that this basic idea is no sense limited to the case of stars, but is equally applicable to giant planets, the atmosphere of the Earth and meteorology in particular, to oceanography, or other types of flow problems whenever it is possible to reasonably model the influence of the environment of a simulation box through boundary conditions. Indeed, also in some of those other scientific disciplines the equivalent of a *box-in-a-star ansatz* already has a decade-long tradition in applications.
*Generalization: simulations with artificial boundaries inside the star.* The simulation can be designed such as to *exclude the near-surface layers* in numerical simulations of just those stars for which in turn the Cartesian box-in-a-star approach is particularly suited for simulations of their surface layers. Here, the upper (near surface) boundary condition is located sufficiently inside the star such that large scale flow structures can be resolved throughout the simulation domain. This permits to set up a *shell-in-a-star* approach where the curved geometry of a star (either a sphere or ellipsoid) is properly accounted for. In the spherical case, the stellar radius replaces the vertical coordinate used in box-in-a-star type simulations and a series of shells then builds up the simulation grid which may be a sector (in 2D) or a full shell (in 3D). Pioneered at about the same time by Gilman and Glatzmaier (1981) as its box-in-a-star cousin, this approach has since been applied to the case of (rotating) stars including our Sun and planets including the interior of our Earth even though the latter in terms of viscosity and, in particular, Prandtl number ($\mathrm{Pr} \gg 1$) is the extreme opposite^3^ of the Sun ($\mathrm{Pr} \ll 1$).
2. For several, special physical situations it is possible to perform full 3D LES of entire stars with a *star-in-a-box* approach: for supergiants, especially for AGB stars, such simulations are feasible as the large, energy carrying scales of the flow are no longer small compared to the stellar diameter (cf. Sect. 4.7 in Freytag et al. 2012, and references therein). This is similar to supernovae where spatial and temporal scales separated in earlier evolutionary stages by orders of magnitudes become comparable to each other leaving only the turbulent flame front to subgrid modelling (Röpke and Schmidt 2009). We note that the transition from this kind of simulation to box-in-a-star and shell-in-a-star cases is not a sharp one, since for AGB star simulations (Freytag et al. 2012) the central region of the star is also not included for lack of resolution.
*Generalization: simulations with mapped grids and interpolation between grids to generate natural boundaries.* Although in most cases not affordable for stellar simulations with realistic microphysics other than for the special case of supernovae, grid mapping and interpolation between grids can be used to avoid artificial boundaries inside a star and to trace the stellar surface layers to optimize resolution. This allows at least in principle to simulate an *entire star, with optimized grid geometry*. We return to the topic of such grids in Sect. 5.4.

For each of these scenarios the computational grid for 3D LES is nowadays between about 100 and 500 grid cells per spatial direction (for very few cases this value may currently range up to around 2000), an impressive development beyond ${\approx } 16$ cells which were the limit faced in the work of Gilman and Glatzmaier (1981) and Nordlund (1982). In case of only two instead of three spatial dimensions, the number of grid cells can be accordingly much larger, for instance, up to 13,000 cells along the azimuthal direction in the simulation of a full $360^{\circ }$ sector, i.e., a ring, located at equatorial latitude, in a 2D LES of the He ii ionization zone and the layers around it for the case of a Cepheid (Kupka et al. 2014) (see also Fig. 14). This way the computations have computer memory requirements which put them in the realm of equipment anywhere between workstations and the largest and fastest supercomputers currently available. But as already indicated, memory consumption and a large spread of spatial scales to be covered by a computational grid are not the only restrictions to affordability of numerical simulations.

### Hydrodynamical simulations and the problem of time scales

#### General principles

Hydrodynamical simulations based on variants of (1)–(10) are conducted to predict the time development of $\rho , \varvec{u}$, and *E* within a specified region in space starting from an initial state. Since in astrophysics that state cannot be determined in sufficient detail from observational data only, the initial conditions have to be constructed from approximations. For numerical simulations of stellar convection one-dimensional stellar structure or stellar atmosphere models can provide the necessary input to initialize the calculation. A more recent, but particularly detailed description of this procedure is given in Grimm-Strele et al. (2015a). Other basic variables such as the velocity field $\varvec{u}$ have to be chosen according to computational convenience since a “realistic guess” is impossible. If a “sufficiently similar” multidimensional state has been constructed from a previous simulation, it can be used to construct a new state through scaling (this is simple for changing resolution, where interpolation is sufficient, but quite subtle, if quantities such as the input energy flux at the bottom or the gravitational acceleration are to be changed). Unless obtained through scaling from a simulation with the same number of spatial dimensions, the initial state is slightly perturbed randomly. Each of $\rho , \varvec{u}, p$, or $\varvec{\mu }$ has been used for this purpose (see Sect. 3.6 in Muthsam et al. 2010, Sect. 2 in Kupka et al. 2012, and Sect. 2.7 in Mundprecht et al. 2013 for examples of such different perturbations being used with the *same* numerical simulation code).

The simulation is then conducted for a time interval $t_\mathrm{rel}$ during which the physical state is supposed to relax to a “typical state”. This is followed by simulation over a time interval $t_\mathrm{stat}$ adjacent to the relaxation time $t_\mathrm{rel}$. The numerical data obtained during $t_\mathrm{stat}$ are then considered suitable for physical interpretation. The physical meaningfulness of this procedure requires that an *ergodicity hypothesis* holds (Tsinober 2009): essentially, one expects that a sufficiently long time series of measurements or of a numerical simulation has the same statistical properties as an average obtained from several (possibly shorter) time series each of which is related to a different initial condition. This requires that the measured properties are invariant under time evolution (Chap. 3.7 in Tsinober 2009), an *“intuitively evident”* property of turbulence which is in fact very difficult to prove. Particularly, there are flows which are only turbulent in a limited domain, such as turbulent jets and wakes past bodies (Tsinober 2009). These may not even be “quasi-ergodic” which would ensure otherwise that the physical states in phase space are visited by a long-term simulation according to their realization probability.

**Fig. 2:**
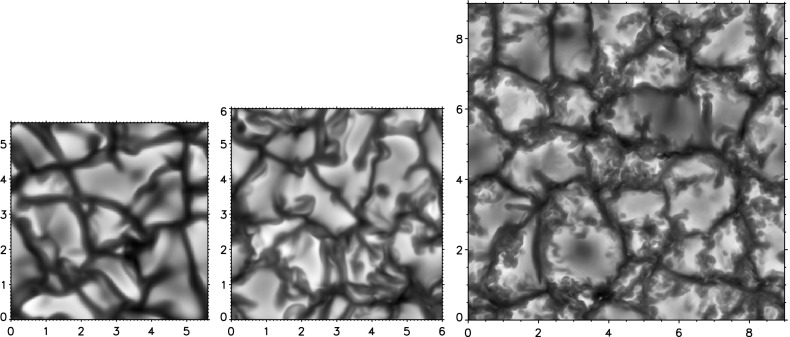
Tracing granules with the vertically emerging continuum intensity at 500 nm which results from numerical simulations with the CO$^5$BOLD code (*left figure*), the Stagger code (*middle figure*), and the MURaM code (*right figure*). Units of the two horizontal axes are Mm. While different resolution, numerical methods, and domain size result in different granule boundaries, the basic flow pattern remains unaltered. Image reproduced with permission from Beeck et al. (2012), copyright by ESO

Nevertheless, the assumption that *turbulent convective* flows “*forget*” their detailed initial conditions is considered to be well-confirmed by current research. The *mean thermal structure* (and also large-scale or global magnetic fields, the latter being excluded from a more detailed discussion here anyway) can have a longer “memory”, i.e., their initial state has an influence on the solution over long integration times, a principle used to speed up relaxation described further below in Sect. 2.3.4. But the mean thermal structure is also more influenced by the boundary conditions of the problem than the turbulent flow field which adjusts itself to a state that is often very different from its initial condition. Eventually, the granulation pattern of solar convection is found with each numerical code capable of doing such kind of simulations (cf. Fig. 1 in Beeck et al. 2012, reprinted here as Fig. 2 for convenience). Even if quite different solar structure models are used as initial states of a simulation, the same average thermal structure is recovered (cf. Sect. 3.3 of Grimm-Strele et al. 2015a). The numerical simulation approach is hence tenable for astrophysical applications. Thus, one can start from approximate models, relax the simulations towards a statistically stationary state (cf. Pope 2000), and perform a numerical study with one or a few long-term simulation runs. But what are the minimum and maximum time scales to be considered for this kind of numerical simulation? Let us consider minimum time scales first.

#### Time steps and time resolution in numerical simulations of stellar convection

In applications to helioseismology, for example, we might want to study the fate of sound waves near the solar surface whereas stellar evolution during core or shell burning of hydrogen or helium takes place on time scales which depend on nuclear processes, chemical composition, and the total stellar mass. As a result, the time scales of interest may range from a few seconds to more than $10^{17}\,\mathrm{s}$ (and even much smaller time scales are of relevance in stars other than the Sun, for instance, for white dwarfs, which in turn gradually cool on time scales of billions of years). Can one get around those 17 orders of magnitude when performing a “3D stellar evolution simulation”? Not without introducing some kind of averaging which means to introduce a new set of basic equations: whether grid-based or particle-based, the maximum allowed time steps as well as the required duration of a numerical simulation are properties which stem from the dynamical equations themselves and cannot easily be neglected. We discuss the most important time step restrictions in the following.

The best-known constraint on time integration is the Courant–Friedrichs–Lewy (CFL) limit due to advection (Strikwerda 1989). For a discretization of the NSE with mesh widths $\varDelta x, \varDelta y, \varDelta z$ in each of the three spatial directions it requires that the time step $\varDelta t$ is bounded by

11 $$\begin{aligned} \varDelta \,t_{\mathrm{adv}} \leqslant C_{\mathrm{adv}} \min \left\{ \varDelta x, \varDelta y, \varDelta z\right\} / {\max (\left| \varvec{u}\right| )}. \end{aligned}$$

In case of a variable mesh the minimum of (11) over each grid cell has to be taken. $C_{\mathrm{adv}}$ depends on both temporal and spatial discretization schemes chosen. This limit is obtained from linear (von Neumann) stability analysis of the advection terms in (1)–(3) and ensures that each signal, i.e., a change in the solution which propagates with velocity $\varvec{u}$, is taken into account during each time step. In practice, (11) cannot be overcome even by implicit time integration methods. The reason is that as long as the flow is not close to stationarity, throughout its evolution the solution changes on just that time scale $\tau \sim \varDelta \,t_{\mathrm{adv}}$, typically by several percent or more. Hence, even fully implicit time integration methods cannot exceed a value of $C_{\mathrm{adv}} \sim 1$. We refer to the discussion of the ADISM method in Sect. 3 of Robinson et al. (2003) where a time step at most five times that of an explicit method for the case of a solar granulation simulation had been achieved and the maximum $\varDelta t$ was indeed given by advection. Note that fast moving shock fronts are already taken into account by (11). In practice, attempts of increasing $\varDelta t$ beyond what would correspond to a value of $C_{\mathrm{adv}} \gtrsim 1$ will lead to fail in solving the non-linear system of equations obtained in fully or semi-implicit time discretization methods while explicit methods will run into the usual exponential growth of linear instabilities (cf. Strikwerda 1989). Since locally the flow can become slightly supersonic near the solar surface (Bellot Rubio 2009), for values of $h \sim 12$–15 km and a sound speed of roughly $10~\mathrm{km}\,\mathrm{s}^{-1}$ (see Fig. 18.11 in Weiss et al. 2004) we obtain that $\varDelta t \lesssim 1\,\mathrm{s}$ in an LES of the surface of the solar convection zone.

Sound waves which originate (Chap. 8 in Landau and Lifshitz 1963, Chap. 10 in Richtmyer and Morton 1967, Chap. 3.6 in Batchelor 2000) from the presence of $\mathrm{grad}\,p$ in (9) can introduce restrictions similar to (11). Tracking sound waves on a computational grid requires to resolve their motion between grid cells. As also revealed by a local characteristics analysis this requires that for explicit time integration methods the sound speed $c_\mathrm{s}$ is added to the flow velocity in (11), whence $\varDelta \,t_{\mathrm{cour}} \leqslant C_{\mathrm{adv}} \min \left\{ \varDelta x, \varDelta y, \varDelta z\right\} / {\max (\left| \varvec{u}\right| +c_\mathrm{s})}$ (see Chap. 12 in Richtmyer and Morton 1967, cf. Muthsam et al. 2010). If accurate tracking is not needed, particularly for low Mach number flows, where sound waves carry very little energy, this restriction can be avoided by numerical methods which use an additive splitting approach or analytical approximations to allow implicit time integration of the $\mathrm{grad}\,p$ term (see Sects. 4, 5).

The application of additive splitting methods is motivated by the structure of (9)–(10) where each term corresponds to a particular physical process which in turn can impose a time step restriction $\tau \leqslant \varDelta t$ on a numerical method. This algebraic structure of the NSE simplifies the construction of semi-implicit or implicit–explicit methods which can remove such restrictions as long as the solution changes only by a small amount during a time interval $\tau \sim \varDelta \,t$. According to linear stability analysis (Strikwerda 1989) terms representing diffusion processes such as viscous friction, $\mathrm{div}\,\varvec{\pi }$ and $\mathrm{div}\,(\varvec{\pi } \varvec{u})$, conductive heat transfer, $\mathrm{div}\,\varvec{h} =\,\mathrm{div}\,(K_\mathrm{h}\,\mathrm{grad}\,T)$, and radiative transfer in the diffusion approximation, $\mathrm{div}\,\varvec{f}_\mathrm{rad} =\,\mathrm{div}\,(K_\mathrm{rad}\,\mathrm{grad}\,T)$, give rise to restrictions of the following type: $\varDelta \,t_{\mathrm{visc}} \leqslant C_{\mathrm{visc}} \min \left\{ (\varDelta x)^2, (\varDelta y)^2, (\varDelta z)^2\right\} / {\max (\nu )}$ and, in particular,

12 $$\begin{aligned} \varDelta \,t_{\mathrm{rad}} \leqslant C_{\mathrm{rad}} \min \left\{ (\varDelta x)^2, (\varDelta y)^2, (\varDelta z)^2\right\} / {\max (\chi )}. \end{aligned}$$

For realistic stellar microphysics $\varDelta \,t_{\mathrm{visc}}$ poses no restriction, since in practice $\varDelta \,t_{\mathrm{visc}} \gg \varDelta \,t_{\mathrm{adv}}$ for achievable spatial resolutions (Sect. 2.2). However, condition (12) can lead to serious limitations not only in convection studies with idealised microphysics (cf. Kupka et al. 2012), but even more so for the simulation of convection in stars such as Cepheids (Fig. 9 in Mundprecht et al. 2013). This restriction can be avoided using fully implicit time integration methods (cf. Dorfi and Feuchtinger 1991, 1995; Dorfi 1999) or more cost-efficient implicit–explicit methods (e.g., Happenhofer 2013). Moreover, within the photospheric layers of a star condition (12) is relieved, if the radiative transfer equation is solved instead of the diffusion approximation, which anyway does not hold in an optically thin fluid. For the linearization of this problem it was shown (Spiegel 1957) that in the optically thin limit the relaxation rate of temperature perturbations by radiation is proportional to the (inverse) conductivity only and with a smooth transition to a quadratic dependence on grid resolution for the optically thick case represented by Eq. (12) (see Sect. 3.2 in Mundprecht et al. 2013):

13 $$\begin{aligned} \varDelta \,t_{\mathrm{rad}}&\lesssim \min \left( \frac{c_\mathrm{p}}{16\kappa \sigma T^{3}}\left( 1-\frac{\kappa \rho }{k}\,\mathrm{arccot}\frac{\kappa \rho }{k}\right) ^{-1}\right) \nonumber \\&=\min \left( \frac{1}{\chi }\,\frac{1}{3(\kappa \rho )^2}\left( 1-\frac{\kappa \rho }{k}\,\mathrm{arccot}\frac{\kappa \rho }{k}\right) ^{-1}\right) . \end{aligned}$$

The symbols used here have mostly been introduced in Table 1 and the minimum is obtained by relating the inverse size *k* of the perturbation to the grid spacing: $k=C_\mathrm{rad}/\min \{\varDelta x,\varDelta y, \varDelta z\}$ and $C_\mathrm{rad}$ depends on the numerical method (typically, $C_\mathrm{rad} \approx 1$). From Taylor expansion it is straightforward to see that in the limit of large optical depth $(\kappa \,\rho k^{-1} \rightarrow \infty )$ Eq. (13) coincides with (12), if we take the maximum of $\chi$ in both equations. For small optical depth $(\kappa \,\rho k^{-1} \rightarrow 0)$ the dependence on *k* and thus grid resolution disappears: $\varDelta \,t_{\mathrm{rad}}\lesssim \min (c_\mathrm{p} / (16\kappa \sigma T^{3}))=\min ((3 \chi (\kappa \rho )^2)^{-1})$ which is to be compared with the optically thick case where $\varDelta \,t_{\mathrm{rad,thick}}\lesssim (3 \chi (\kappa \rho )^2)^{-1} (3(\kappa \rho )^2/k^2)$. From taking the ratio $\varDelta \,t_{\mathrm{rad,thick}}/\varDelta \,t_{\mathrm{rad}}=3(\kappa \rho )^2/k^2$ and considering constant values of grid spacing it becomes evident that $\varDelta \,t_{\mathrm{rad,thick}}$ and thus Eq. (12) is far more restrictive than (13). Firstly, the product $\kappa \rho$ is orders of magnitudes smaller for the outermost layers of a star than for its interior. Furthermore, for a finite *T* and $c_\mathrm{p}$ the quantity $\varDelta \,t_{\mathrm{rad}}$ becomes large for the outermost layers as long as $\kappa$ continues to drop. Changing to the physically appropriate criterion (13) concurrently with solving the full radiative transfer equation instead of resorting to the diffusion approximation hence removes the unnecessary restrictions of the latter for optically thin fluids. However, also if the radiative transfer equations are solved, a high radiative cooling rate $q_\mathrm{rad}$ may still introduce prohibitively small time steps $\varDelta \,t \leqslant \varDelta \,t_{\mathrm{rad}}$. Examples for this problem are A-type stars (Freytag and Steffen 2004; Kupka et al. 2009) or the lower photosphere of Cepheids (Mundprecht et al. 2013). Again this restriction can be resolved by means of implicit time integration methods (see Dorfi and Feuchtinger 1991, 1995) as long as the relative changes of the solution in each grid cell, after some initial relaxation, remain small.

The pure source terms which are due to gravitational acceleration, $\rho \varvec{g}$, and the generation of energy by nuclear reactions, $q_\mathrm{nuc}$, do not directly depend on grid resolution. As such they can be neglected in asymptotic stability analyses (Strikwerda 1989), but can at least in some special cases cause time step restrictions. For the diffusive phase of hydrodynamical simulations of semi-convection buoyancy poses a very moderate restriction: $\varDelta \,t_\mathrm{buoy} \leqslant t_\mathrm{buoy} = \min \left\{ (\varDelta r)^{1/2} / \max (g_\mathrm{r})\right\}$ (where only the vertical or radial grid spacing, $\varDelta r$, and its associated component of gravitational acceleration, $g_\mathrm{r}$, are important, see Kupka et al. 2012 for references). This becomes irrelevant as soon as convective mixing sets in Kupka et al. (2012). Nuclear energy generation in turn sets the time scale of stellar evolution as a whole Weiss et al. (2004) and thus is usually the longest time scale of interest in stellar physics, except in late stages of nuclear burning of massive stars and, of course, during supernovae (Kippenhahn and Weigert 1994).

Hence, in most cases, when using suitable implicit time integration methods, *the time step of a hydrodynamical simulation of stellar convection can become as large as* $\varDelta \,t_{\mathrm{adv}}$, *but not larger than that, since this is the time scale on which convection changes the local state in a grid cell*.

#### Implications from $\varDelta \,t_{\mathrm{adv}}$ for performing LES of stellar convection zones

As we have seen in Sect. 2.3.2 the numerical time integration of (1), (9), and (10) is in any case restricted to a step $\varDelta t$ at each time *t* which is bounded by the minimum (11) of $\varDelta \,t_{\mathrm{adv}}$ over the entire simulation domain for that *t*. Usually, this restriction is most severe for the surface layers of a star. First of all, a much higher spatial resolution is required for a physically meaningful representation of the mean structure along the vertical direction near the top of a star. This is caused by the much smaller scale heights near the surface (Chap. 6.1 in Kippenhahn and Weigert 1994) and the efficient cooling of stars in optically thin layers (e.g., Kippenhahn and Weigert 1994; Weiss et al. 2004). Secondly, the velocity $\varvec{u}$ is also much higher in just those layers. This can be understood by considering the term $\mathrm{div}\,\left( (\rho E + p) \varvec{u} \right)$ in the energy equation (10) which is just the divergence of the *advected flux*, i.e., the sum of convective (or enthalpy) flux and flux of kinetic energy. The vertical components of these fluxes are split as

14 $$\begin{aligned} F_\mathrm{adv} = (\rho E + p) u_\mathrm{vert}= & {} (\rho \varepsilon + p) u_\mathrm{vert} + \frac{1}{2} \rho \varvec{u}^2 u_\mathrm{vert} \nonumber \\= & {} \rho h u_\mathrm{vert} + \frac{1}{2} \rho \varvec{u}^2 u_\mathrm{vert} = F_\mathrm{conv} + F_\mathrm{kin}, \end{aligned}$$

and $h = \varepsilon + p/\rho$ is the specific enthalpy. Evidently, these fluxes result from the non-vanishing velocity field $\varvec{u}$ in convective zones. If, as is the case inside the upper part of the convection zone of the Sun, $F_\mathrm{adv}$ accounts for almost the entire vertical transport of energy (e.g., Stein and Nordlund 1998; Weiss et al. 2004; Grimm-Strele et al. 2015a) and taking into account the much lower density and pressure near the top (cf. Stein and Nordlund 1998; Weiss et al. 2004, or any other model of the solar surface layers), it is clear that the velocity has to increase towards the top of the solar convection zone to maintain a constant luminosity throughout the envelope of the star (cf. Chap. 4 in Kippenhahn and Weigert 1994 and for quantitative estimates Table 14.1 in Weiss et al. 2004). The latter is an indicator of thermal equilibrium which holds during most stellar evolution phases (Kippenhahn and Weigert 1994; Weiss et al. 2004) when no major sources or sinks of energy exist in a convective zone in a stellar envelope other than oscillations around such an equilibrium state (cf. Chaps. 4, 6, and 39 in Kippenhahn and Weigert 1994). In the end, $|\varvec{u}|$ is large(st) near the surface and $\varDelta \,t_{\mathrm{adv}} \lesssim 1\,\mathrm{s}$ limits LES of only the solar surface as well as also any LES of the entire solar convection zone, even if restrictions due to sound waves are eliminated by a (semi-) implicit method as discussed in Sects. 4 and 5. Hence, the time step of an LES of the entire solar convection zone is limited at least by $\varDelta \,t_{\mathrm{adv}}$ as obtained for the top of the simulation domain. The same holds for other stars with surface convection zones, if they are included in the simulation domain. This is one important reason why the surface layers are excluded in current numerical simulations of the lower part of the convection zone in the Sun and in similar stars (cf. Miesch 2005).

#### Duration of numerical simulations of stellar convection

For how long do we have to conduct an LES of stellar convection? To this end the following time scales are of interest: the free fall time or time scale of hydrostatic equilibrium ($t_{\mathrm{hyd}}$), the related acoustic time scale ($t_{\mathrm{ac}}$), the convective turn over time scale ($t_{\mathrm{conv}}$), the time scale for relaxation towards statistical equilibrium ($t_{\mathrm{rel}}$), the time required to achieve statistical stationarity when evaluating a physical quantity ($t_{\mathrm{stat}}$), the time scale of stellar pulsation ($t_{\mathrm{osc}}$), the time scale of thermal adjustment ($t_{\mathrm{therm}}$), the Kelvin–Helmholtz time scale ($t_{\mathrm{KH}}$), and the nuclear evolution time scale ($t_{\mathrm{nuc}}$). There are also time scales related to rotation, concentration diffusion, magnetic field interaction, e.g., but their role either follows from an extension of the following discussion or requires more general dynamical equations than (1)–(10) beyond the scope of this review.

A brief discussion of $t_{\mathrm{hyd}}, t_{\mathrm{therm}}$, and $t_{\mathrm{nuc}}$ can be found in Chap. 0.2 of Weiss et al. (2004). $t_{\mathrm{hyd}}$ is relevant for stars which are not yet or no longer in hydrostatic equilibrium, i.e., during early phases of star formation or during a supernova. It is of the order of the time it takes for a sound wave to travel from the surface of a star to its centre. For the Sun $t_{\mathrm{hyd}}$ is about 1 h (Weiss et al. 2004). Except in the case of exploding stars, stellar convection takes place in conditions of approximate hydrostatic equilibrium, hence we do not consider $t_{\mathrm{hyd}}$ any further.

The convective turn over time scale $t_{\mathrm{conv}}$ can in general be defined as

15 $$\begin{aligned} t_{\mathrm{conv}} =\int _{r_\mathrm{a}}^{r_\mathrm{b}} u_x^{-1}(r)\,\mathrm{d}r, \end{aligned}$$

where $r_\mathrm{b} - r_\mathrm{a}$ is either the vertical (radial) height *H* of the simulation box or an interval contained inside it, $(r_\mathrm{b} > r_\mathrm{a})$, and $u_x = \langle (u-\langle u\rangle )^2\rangle ^{0.5}$ is the root mean square difference of the local vertical velocity and its horizontal mean, usually also averaged in time. If measured over the entire length *H* this time scale in practice is always longer than the acoustic time scale or *sound crossing time* $t_\mathrm{ac}$. The latter is given by

16 $$\begin{aligned} t_\mathrm{ac} = \int _{r_\mathrm{a}}^{r_\mathrm{b}} c_\mathrm{s}^{-1}(r)\,\mathrm{d}r, \end{aligned}$$

where $c_\mathrm{s}$ is the local, horizontally averaged sound speed. Following Chaps. 3 and 4 in Kippenhahn and Weigert (1994) and Chap. 17.4 in Weiss et al. (2004) the local Kelvin–Helmholtz time scale is obtained from the virial theorem as

17 $$\begin{aligned} t_{\mathrm{KH}} = \left( -3{\int _{{M_s(r_\mathrm{a})}}^{M_s(r_{\mathrm{b}})}} p \rho ^{-1}\,{\mathrm{d}}M_s\right) / {\mathcal {L}}, \end{aligned}$$

with the luminosity ${{\mathcal {L}}}$ given by ${{\mathcal {L}}} = 4\pi r^2 F_\mathrm{tot}$ for the case of a spherically symmetric star with mass ${{\mathcal {M}}}$. $M_s(r)={{\mathcal {M}}}-M_r$ is the total mass found in the shell when integrating downwards from the surface (note the sign due to the direction of integration and see also Sect. 4.3 in Kupka 2009b, where an extended discussion on the subject of numerical simulation time scales for stellar convection is given). This is also the time scale over which an energy flux of size $F_\mathrm{tot}$ against the direction of $\varvec{g}$ can be sustained by (gravitational) potential energy (Kippenhahn and Weigert 1994; Weiss et al. 2004).

**Fig. 3:**
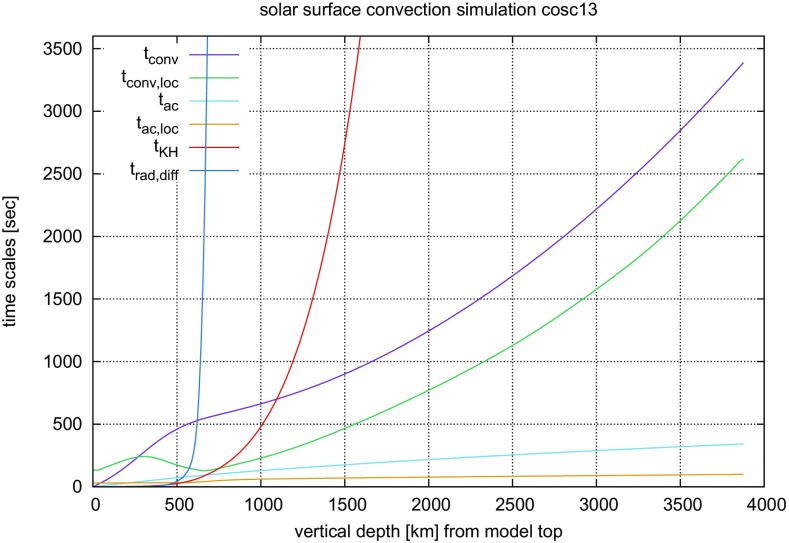
Time scales for a numerical simulation of convection at the solar surface with the ANTARES simulation code (Muthsam et al. 2010) (details on the simulation: Belkacem et al. 2015, in prep.). The solar photosphere extends down to about 700 km, the layer for which the largest temperature gradients are found and the region just around that depth level is known as the superadiabatic peak

$t_\mathrm{KH}$ is often close to the time scale $t_\mathrm{therm}$ on which *thermal equilibrium* is reached, i.e., when local energy production as well as gains and losses through energy transport balance each other (Chap. 5.1 in Weiss et al. 2004, for details on when and why this occurs see Chap. 5.3 in Kippenhahn and Weigert 1994). If the thermal adjustment is due to radiative transfer in the diffusion approximation, it can be estimated from $t_\mathrm{therm} \approx t_\mathrm{rad, diff}$, where

18 $$\begin{aligned} t_\mathrm{rad, diff} \approx (r_\mathrm{b} - r_\mathrm{a})^2 / \chi , \end{aligned}$$

and $\chi$ is the radiative (thermal) diffusivity (we recall that the diffusion approximation of radiative transfer generally holds for stellar interiors, cf. Mihalas and Mihalas 1984; Weiss et al. 2004, and note that for locally rapidly varying $\chi$ this definition can be modified for more accurate estimates). In this case, also $t_\mathrm{KH} \approx t_\mathrm{therm}$ and inside radiative (convectively stable) zones these three time scales hence often agree to within less than an order of magnitude. But this is not always the case, since local energy sources (or sinks) and compression also contribute to thermal adjustment and particularly inside convective zones $t_\mathrm{rad, diff}$ can be much longer than $t_\mathrm{KH}$ or $t_\mathrm{therm}$ (see Fig. 3). Under special circumstances such as an isothermal core in an evolved star even $t_\mathrm{KH}$ and $t_\mathrm{therm}$ largely differ, too (see Chap. 5.3 in Kippenhahn and Weigert 1994 for details). In any case, relaxation to a statistically stationary state of a star requires the simulated domain of the object to be in *thermal equilibrium* (Chap. 5.1 of Weiss et al. 2004) and hence $t_\mathrm{therm}$ is of major interest to any LES of stellar convection. In case there is no flow and no local energy sources, the only thermal energy transport is through radiative (or heat) diffusion, whence $t_\mathrm{therm} = t_\mathrm{rad, diff}$, which follows straightforwardly from the dynamics of the temperature field being described by the heat equation (see Chap. 5.3 in Kippenhahn and Weigert 1994). If energy can be stored through compression, as in a pulsating star, or there is energy generation by nuclear processes, a more general equation for temperature evolution has to be considered and if convection or other driving mechanisms of a non-zero flow occur, the time scale of changes according to the energy equation (10) have to be considered. In Chaps. 5.3 and 6.4 of Kippenhahn and Weigert (1994) it is demonstrated for both the hydrostatic and the non-hydrostatic case, how one can estimate $t_\mathrm{therm}$ from the temperature (or, in the end, energy) equation to be

19 $$\begin{aligned} t_\mathrm{therm} \approx t_\mathrm{KH} \end{aligned}$$

except for cases where $L \approx 0$ and, consequently, the difference between the time scale for reacting to a perturbation from equilibrium ($t_\mathrm{therm}$) and the time scale to transport a certain amount of energy in equilibrium ($t_\mathrm{KH}$) becomes relevant, for then $t_\mathrm{KH} \gg t_\mathrm{therm}$. We also note here that the kinetic energy contained in the flow of an LES of stellar convection is usually negligibly small compared to the thermal energy contained in the simulation volume (even for the case of the Sun with its very efficient, quasi-adiabatic convection it is less than 0.1% in a case similar to that one shown in Fig. 3, as was demonstrated by Grimm-Strele et al. 2015a—see their Fig. 12, whence the discussion of relaxation of thermal energy of Kippenhahn and Weigert 1994 applies here, too).

Since $t_\mathrm{therm}$ can be very long, it is advisable to construct suitable initial conditions which allow reaching thermal equilibrium quickly within the LES itself. Otherwise, excessive relaxation times $t_{\mathrm{rel}} \sim t_{\mathrm{therm}}$ occur. For instance, one can consider the vertical (radial) temperature and pressure profile of a one-dimensional model of stellar structure or a suitably deep reaching stellar atmosphere model for an initial state. This avoids $t_{\mathrm{rel}}$ to become a few 100 h for a simulation of solar surface convection instead of once or twice $t_{\mathrm{conv}}$, where the latter is evaluated for the entire box depth *H* and is between 1 and 2 h for a solar granulation simulation as depicted in Fig. 3 (see also Grimm-Strele et al. 2015a). We note that frequently, convective turn over time scales are approximated and evaluated “locally” as $t_{\mathrm{conv,loc}} = u_x/(2 H_p)$. The evaluation of variables often takes place somewhere below the superadiabatic peak. In that case, $t_{\mathrm{rel}} \sim 5 t_{\mathrm{conv,loc}}$ to $10 t_{\mathrm{conv,loc}}$. Since there is some arbitrariness in the location and the reference length scale ($2 H_p$, e.g.), we prefer to refer to use $t_{\mathrm{conv}}$ as given by Eq. (15). Also a local acoustic time scale can be defined this way from local sound speed and pressure scale height, $t_{\mathrm{ac,loc}} = c_s/(2 H_p)$. Figure 3 compares some of those time scales for an LES of solar convection. By virtue of a suitable solar 1D model which had been used to initialize the numerical simulation, $t_{\mathrm{rel}} < 2 t_{\mathrm{conv}}$ was sufficient for this simulation before the statistical evaluation of the simulation could be started. The latter was made for $t_{\mathrm{stat}} > 100 t_{\mathrm{ac}}$ to study damping of solar oscillations, which—as pointed out in the pioneering work by Stein and Nordlund (2001)—can be found in and studied also by means of numerical simulations. We note here that while $t_{\mathrm{osc}} \gtrsim t_{\mathrm{ac}}$, mode damping occurs on time scales $t \gg t_{\mathrm{ac}}$. In comparison, $t_\mathrm{KH}$ grows to 84 h at the bottom of the simulation whereas $t_{\mathrm{rad, diff}}$ reaches even 70,000 years. The latter would be lowered by merely an order of magnitude if instead of $H^2$ as in Eq. (18) one considers $t_\mathrm{rad, diff, loc} = H_p^2/\chi$. However, both $t_\mathrm{rad, diff}$ and $t_\mathrm{rad, diff, loc}$ are totally irrelevant in this context, since the radiative flux is negligibly small in this part of the solar convection zone. Thus, thermal relaxation is not determined by radiative diffusion and $t_\mathrm{KH} \lll t_\mathrm{rad, diff, loc} \ll t_\mathrm{rad, diff}$.

**Fig. 4:**
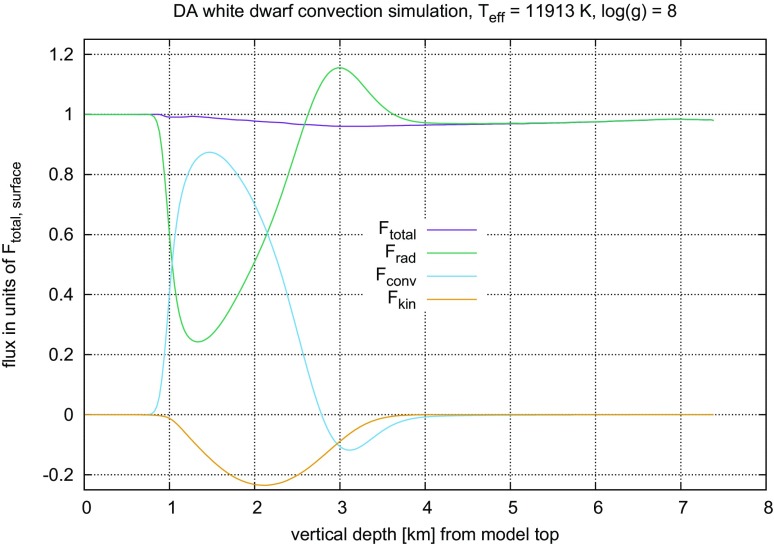
Vertically outward directed energy fluxes scaled in units of the surface flux $F_{*} = \sigma T_\mathrm{eff}^4$ for an LES of convection at the surface of a DA type white dwarf with the ANTARES simulation code (Muthsam et al. 2010) (Kupka et al. 2017, submitted; in that article the flux data are scaled with respect to the input flux at the *bottom* which corresponds to a $T_\mathrm{eff}$ of 11,800 K). The photosphere extends down to 1 km, the convectively unstable zone ranges from 0.8 to 2 km, and below 4 km the entire flux transport is essentially due to radiation. No flow is permitted through the lower vertical boundary where a purely radiative energy flux enters

**Fig. 5:**
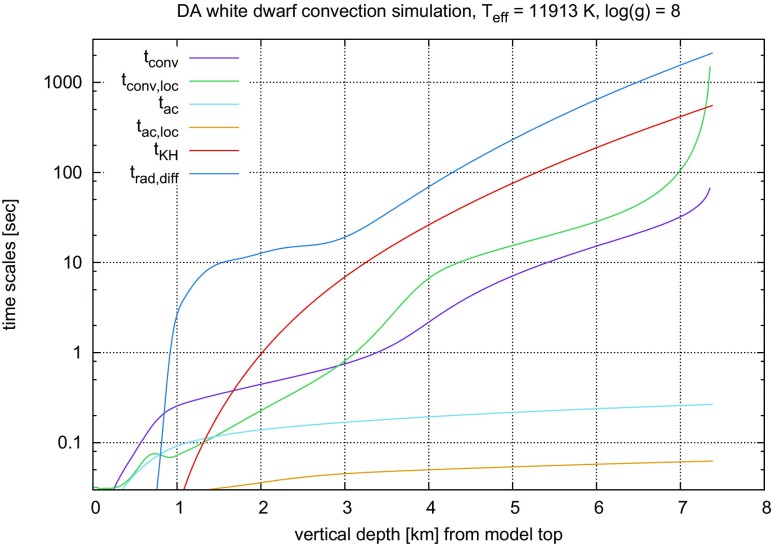
Time scales for a numerical simulation of convection at the surface of a DA type white dwarf with the ANTARES simulation code (Muthsam et al. 2010) (for details on this simulation cf. Kupka et al. 2017, submitted; the figure shown in this review contains additional quantities from the same data set). $t_{\mathrm{rel}}$ was about 10 s leaving a residual difference in flux constancy of up to 4% (see Fig. 4)

The situation is quite different for the case of a DA type white dwarf with a shallow surface convection zone caused by the ionization of hydrogen. No accurate guesses of the thermal structure are possible from 1D models due to the uncertainty of their convection model parameters (in particular the mixing length) and their neglect of a sizeable overshooting below the convection zone which alters the local stratification (see Fig. 4). Thermal relaxation can be helped here by prerelaxation for 5 s with an LES in 2D starting from a carefully chosen 1D model. The resulting stratification is used to construct a new 1D model from which the 3D LES is started and $t_{\mathrm{rel}}$ was 10 s or ${\approx } 40\,t_{\mathrm{ac}}$ for the simulation shown. To further reduce the residual flux error of up to 4%, as seen from the total, vertically outward directed energy flux $F_\mathrm{total}=F_\mathrm{rad}+F_\mathrm{conv}+F_\mathrm{kin}$ in Fig. 4 for the lower part of the model (between 5 and 7 km), down to a value of 2% would require at least doubling again $t_{\mathrm{rel}}$ at which point the accuracy limit imposed by the radiative transfer solver would be reached (notice the dip in $F_\mathrm{total}$ at 1 km and for a discussion of flux conservation see, e.g., Hurlburt et al. 1984, 1994; Canuto 1997a). The extent of conservation of total energy flux is thus an indicator of whether statistical and thermal equilibrium have been reached. Clearly, for this simulation $t_{\mathrm{conv,loc}}$ taken inside the convection zone is a useless measure or relaxation. Rather (see Fig. 5), we have $t_{\mathrm{rel}} \approx t_{\mathrm{conv}}$ if $t_{\mathrm{conv}}$ is evaluated *close to* the bottom of the convective zone, but notice the closed bottom boundary forces $t_{\mathrm{conv}}$ to diverge where $u_x=0$. Alternatively, $t_{\mathrm{rel}} \approx t_{\mathrm{KH}}$ if the latter is evaluated at a depth of 4 km. Below that layer the total flux is essentially due to radiation and thus convection does not modify the thermal mean structure and the initial state is sufficiently close to statistical equilibrium also for the 3D LES (the turbulent pressure $p_\mathrm{turb}$ is less than 0.01% of the total pressure there, too). Thus, $t_{\mathrm{rel}} \approx t_{\mathrm{KH}}(x_\mathrm{rel})$, where $x_\mathrm{rel}$ is the highest vertical layer for which the thermal stratification is found unaltered from the initial condition independently of simulation time. The idea behind this definition is that for both the solar case considered above, where the lower part of the simulation domain is quasi-adiabatically stratified, and for the white dwarf example where the same region is subject to purely radiative energy transport in the sense that $F_\mathrm{total} \approx F_\mathrm{rad}$ (even if there are still high velocity fields), the *initial, thermal stratification can be accurately guessed* and thus the fluid is *already in thermal equilibrium in that region*. Thermal relaxation hence is needed only for layers lying *above* $x_\mathrm{rel}$. To compute $t_{\mathrm{rel}}$ from Eq. (17) we set $r_a = x_\mathrm{rel}$ and $r_b = x_\mathrm{top}$. We note that $t_{\mathrm{rel}} \approx t_{\mathrm{KH}}(x_\mathrm{rel})$ also holds for the solar simulation depicted in Fig. 3, which is also supported by the results presented in Grimm-Strele et al. (2015a). We hence suggest $t_{\mathrm{rel}} \approx t_{\mathrm{therm}}(x_\mathrm{rel})$ as most appropriate estimate of $t_{\mathrm{rel}}$ for a simulation of stellar convection to attain a thermally relaxed state when using typical starting models as initial conditions and use $t_{\mathrm{therm}}(x_\mathrm{rel}) \approx t_{\mathrm{KH}}(x_\mathrm{rel})$ for conditions for which $t_{\mathrm{therm}} \sim t_{\mathrm{KH}}$ is valid (cf. Chap. 5 of Kippenhahn and Weigert 1994). This yields a good approximation for the scenarios shown in Fig. 3 and Fig. 5 and the numerical experiments of Grimm-Strele et al. (2015a).

For the entire Sun, $t_\mathrm{KH}$ is about $2 \times 10^7$ years (Chap. 17.4 in Weiss et al. 2004). As explained in Sect. 4.3 of Kupka (2009b), if the initial condition is sufficiently far from thermal or even hydrostatic equilibrium, a much stronger energy flux can be driven and $t_\mathrm{KH}$ becomes much smaller due to a much larger ${{\mathcal {L}}}$. However, once closer to equilibrium, ${{\mathcal {L}}}$ also approaches its equilibrium value and further changes occur much more slowly. Thus, as noted in Grimm-Strele et al. (2015a), a small adjustment of the input energy or entropy at the bottom of the simulation domain of an LES of just the solar surface will trigger a long process of very slow thermal relaxation of the quasi-adiabatic layers of the convective interior. Indeed, if the inflowing entropy or internal energy of a numerical simulation with open, penetrable lower vertical boundary were required to change by a few percent, $t_{\mathrm{rel}} \sim t_{\mathrm{therm}}=t_{\mathrm{therm}}(x_\mathrm{bottom})$ cannot be avoided which in practice means $t_{\mathrm{rel}} \sim t_{\mathrm{KH}}(x_\mathrm{bottom})$ (cf. Grimm-Strele et al. 2015a), which we obtain from setting $r_a = x_\mathrm{bottom}$ and $r_b = x_\mathrm{top}$ in Eq. (17). This holds unless a better guess of the thermally relaxed stratification is constructed to serve as a new starting model. A suitable initial condition of an LES of convection should thus ensure that $t_{\mathrm{rel}} \ll t_{\mathrm{therm}}(x_\mathrm{bottom})$ whereas $t_{\mathrm{therm}} \ll t_{\mathrm{nuc}}$ is guaranteed anyway by the physical state of a star through all but some of the final evolutionary stages (cf. also Weiss et al. 2004).

For state-of-the-art LES of stellar convection, $\varDelta \,t_{\mathrm{adv}} \ll t_{\mathrm{conv}}$ by factors of a few 1000 to a few 10,000 depending on the *size of the simulation domain* and the *resolution of the simulation*. Ideally, $t_{\mathrm{rel}} \sim t_{\mathrm{conv}}$, but this depends very much on the ability to *guess a thermally relaxed state*. This is usually possible, if the stratification is quasi-adiabatic in the entire lower part of the simulation. At this point it is important to remember that $t_{\mathrm{therm}}$ only refers to thermal relaxation within the simulation domain and not for the entire object. Since the actual time step $\tau$ of a simulation will be somewhat less than $\varDelta \,t_{\mathrm{adv}}$, as discussed in Sect. 2.3.2, relaxation towards a statistically stationary state eventually requires some $10^5$ to a few $10^6$ time steps in current LES of stellar convection.

**Fig. 6:**
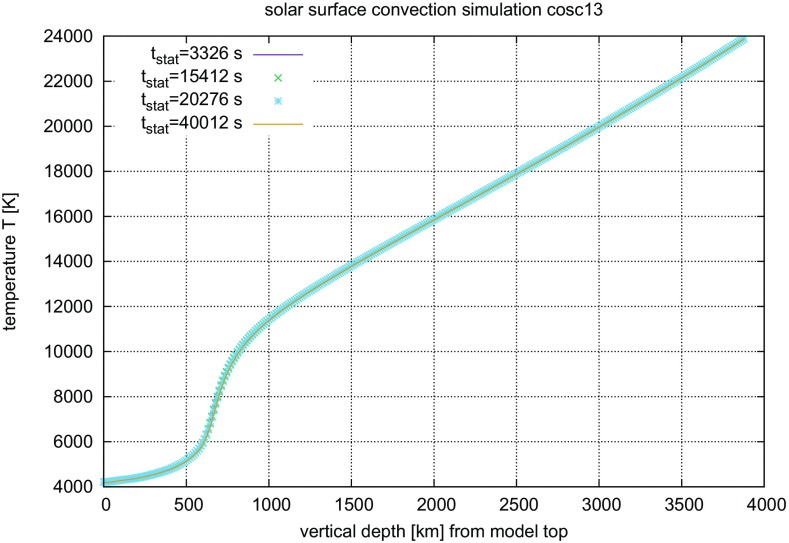
Mean temperature $\overline{T}$ as a function of box depth for an LES of the convective solar surface with the ANTARES simulation code (Muthsam et al. 2010) (for details on this simulation cf. Belkacem et al. 2015, in prep.). Already a rather short averaging over $t_{\mathrm{stat}} \approx t_{\mathrm{conv}}$, where $t_{\mathrm{conv}} \approx 3388\,\mathrm{s}$ (see Fig. 3), suffices to compute this quantity

**Fig. 7:**
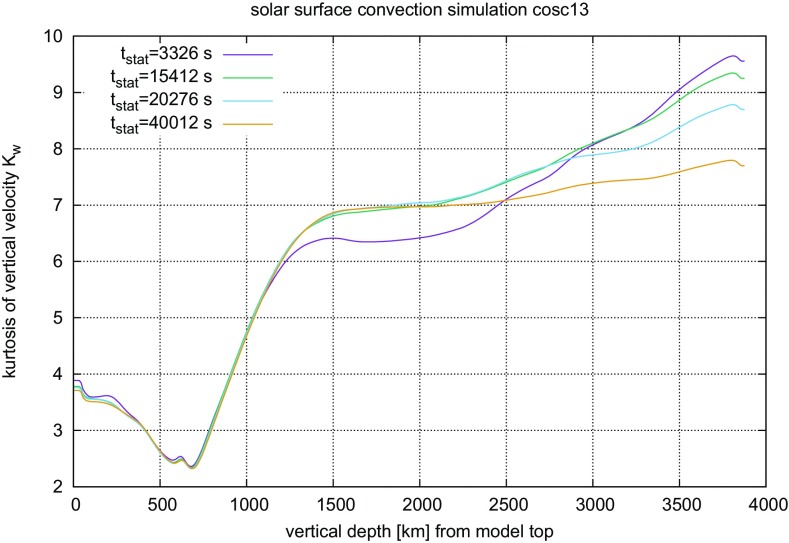
Kurtosis of vertical velocity as a function of box depth for a numerical simulation of convection at the solar surface with the ANTARES simulation code (Muthsam et al. 2010) (see also Fig. 6). Since $t_{\mathrm{conv}} \approx 3388\,\mathrm{s}$ (see Fig. 3), a much longer averaging of at least $t_{\mathrm{stat}} > 10 t_{\mathrm{conv}}$ is required to compute this quantity (here, $t_{\mathrm{stat}}/t_{\mathrm{conv}}$ is about 0.98, 4.55, 5.98, and 11.81)

The time $t_{\mathrm{stat}}$ required to obtain well converged statistical averages from an LES of stellar convection depends very much on the observations the simulation data shall be compared to and the physical quantity of interest. The mean temperature $\overline{T}$ or the turbulent pressure can be inferred from an LES over just $t_{\mathrm{stat}} \approx t_{\mathrm{conv}}$, as can be seen from Fig. 6 where the temperature profiles of short and long time averaging for a simulation of convective solar surface layers are indistinguishable. A zooming in around the region at a depth coordinate of 700 km, where the temperature gradient is steepest, would reveal there is a slow drift which shifts that region inwards (to the right on the plot) by one simulation grid cell between the shortest and the longest averaging (the former being contained in the latter). This is at the accuracy limit of the simulation. It would even disappear when normalizing the depth scale onto a common reference depth, such as to have a depth of zero where $T=T_\mathrm{eff}$. Data to compute synthetic spectral line profiles usually also require rather short simulation runs as the photons mostly stem from layers with very short adjustment times. For the Sun for both cases $t_{\mathrm{stat}}$ is hence of the order of 1 h or again just about $10^5$ time steps (of course, for this to hold it is fundamental to know a good initial condition such that thermal relaxation is only required for the upper and mid part of the simulation domain, as is the case in the example(s) shown above). Studying stellar oscillations is a different story as is the computation of higher order moments of the basic, dependent variables. While typically $t_{\mathrm{ac}} \leqslant t_{\mathrm{osc}} \leqslant t_{\mathrm{conv}}$, one requires $t_{\mathrm{stat}}$ to be $100\,t_{\mathrm{ac}}$ to $400\,t_{\mathrm{ac}}$ to obtain data suitable to study mode damping (cf. Belkacem et al. 2015, in prep.). Likewise, for a fourth order moment such as $K_w = \overline{(w - \langle w\rangle _\mathrm{h})^4} / (\overline{(w - <w>_\mathrm{h})^2})^2$, which is of interest to Reynolds stress modelling and to modelling in helio- and asteroseismology (Belkacem et al. 2006a, b), a similar duration of the LES in terms of $t_{\mathrm{stat}}$ is required, as is demonstrated by Fig. 7. We note here that $\langle \cdot \rangle _\mathrm{h}$ refers to an instantaneous horizontal average while the overbar denotes an ensemble average obtained from time averaging horizontal averages (see Sect. 3). Hence, for such quantities simulations taking $10^6$ to even $10^8$ time steps may have to be performed and the latter is close to the limit achievable for 3D LES on computational grids with several 100 cells per direction with common supercomputing resources. As a final remark on this topic we would like to point out that contrary to a study of mode damping and driving, where the time correlation is of direct physical interest, the situation is different for physical properties which are expected to reach a quasi-equilibrium state as a function of time, such as $\overline{T}$ or $K_w$. In this case, the number of *realizations* achieved in a simulation is relevant and thus a longer time series can be replaced by a shorter time series in a simulation with larger horizontal extent at identical grid resolution. Trading points in time with points in space is advantageous for quantities with a large horizontal correlation length. But it is also more costly in terms of computer memory and in the end it is likely to require a similar number of floating point operations to achieve the same level of statistical convergence as depicted in Fig. 7 for a simulation of more limited horizontal extent (6 Mm per direction in that case) made over a long time interval.

#### Implications from $t_{\mathrm{rel}}$ and $t_{\mathrm{stat}}$ and summary on computational costs

As we have just seen, the duration of a numerical simulation of stellar convection is determined both by requirements of relaxation, i.e., $t_{\mathrm{rel}}$, and the computation of the physical quantities of interest which requires a simulation over a time scale $t_{\mathrm{stat}}$. In the best case, numerical methods allow choosing a time step determined solely by the rate of change of the solution to the system of dynamical equations constructed from (1)–(10). In many cases, (semi-) implicit methods can take care of purely *numerical* restrictions imposed by (radiative) diffusion or sound waves (see Sect. 2.3.2 and also Sects. 4, 5). Then, $\varDelta t \ \approx \varDelta \,t_{\mathrm{adv}}$. Throughout most of the life time of a star, $t_{\mathrm{nuc}}$ is larger than any of the other time scales of interest and $t_{\mathrm{hyd}}$ plays no role either. Thus, $t_{\mathrm{rel}} + t_{\mathrm{stat}}$ determines the duration of the simulation and

20 $$\begin{aligned} N_t = \frac{t_{\mathrm{rel}} + t_{\mathrm{stat}}}{\varDelta t} \end{aligned}$$

its number of time steps and thus the total cost for a given spatial discretization (this is trivially generalized to cases of variable time steps). One can attempt to minimize $t_{\mathrm{rel}}$ by a proper initial guess for the vertical stratification to have $t_{\mathrm{rel}} \ll t_{\mathrm{therm}}(x_\mathrm{bottom})$, since often $t_{\mathrm{therm}} \approx t_{\mathrm{KH}}$. This is no problem for LES of the surface of solar-like stars or red giants since there the layers underneath the observable photosphere are close to adiabatic and thus a thermally relaxed stratification is easy to guess whence $t_{\mathrm{rel}} \approx t_{\mathrm{therm}}(x_\mathrm{rel}) \approx t_{\mathrm{KH}}(x_\mathrm{rel})$ and in practice also $t_{\mathrm{rel}} \approx t_{\mathrm{conv}}(x_\mathrm{bottom})$, and in general

21 $$\begin{aligned} t_{\mathrm{rel}} \approx \max (t_{\mathrm{conv}}(x_\mathrm{bottom}),t_{\mathrm{therm}}(x_\mathrm{rel})). \end{aligned}$$

We note that in case of global numerical simulations of stellar convection with rotation as discussed in Brun and Toomre (2002) or Miesch (2005), e.g., this definition has to be extended to also account for the “spin-up time” of the system (until the flow reaches an equilibrium with respect to rotational motion) and the rotation time scale of the system. Moreover, cases of stellar convection exist where a good initial condition is more difficult to obtain, as was shown with the example in Sect. 2.3.4. However, there are no generally applicable shortcuts for $t_{\mathrm{stat}}$: some quantities such as mean temperature profiles of a relaxed simulation or spectral line profiles for the latter are computable at rather modest efforts, i.e., $t_{\mathrm{stat}} \approx t_{\mathrm{conv}}$, while other calculations such as damping of pressure modes or higher order moments may require $t_{\mathrm{stat}}$ to be several orders of magnitudes larger than $t_{\mathrm{conv}}$. For grids with a few hundred grid points along the vertical (radial) direction, $t_{\mathrm{conv}}$ is typically a few 1000 to a few 10,000 times $\varDelta \,t_{\mathrm{adv}}$ which may be readily understood from Eq. (11) due to the role of advection for convective flow (cf. Sect. 2.3.2). In the end, we have to deal with values of $N_t$ in the range of $10^5$ to $10^8$. The technique of grid refinement as used, e.g., in Muthsam et al. (2010) and Mundprecht et al. (2013) in the ANTARES code, allows pushing these numbers somewhat, since the individual $\varDelta \,t_{\mathrm{adv}}$ on each grid differ. This way one can hope to gain one or at the very most two orders of magnitudes in achievable time steps or local resolution, particularly, if an efficient workload distribution on parallel computers can be achieved. That brings further scientific questions into the realm of computable problems, although it does not *fundamentally* change the limitations imposed by the spread of time scales ($\varDelta t, t_{\mathrm{rel}}, t_{\mathrm{stat}}, \ldots$). We return to these considerations in Sects. 2.5 and 2.6, where we discuss the potential and limitations of 2D simulations in alleviating the computational costs of LES of stellar convection and where we distinguish computable from non-computable problems, respectively.

### Effective resolution and consequences of insufficient resolution

We return to the problem of spatial resolution. Insufficient grid spacing can severely alter the results of any numerical solution of a differential equation up to the level of uselessness and (1)–(10) are no exception to this general statement. An important example in the context of stellar convection modelling is the stellar photosphere: if the vertical temperature and pressure gradients in this region are not resolved with a sufficient number of grid points, the radiative cooling rate, the flow velocities, and the convective flux may differ from results of a resolved simulation by factors of 4 and more (Mundprecht et al. 2013). The same authors also conclude that resulting light curves may be severely “polluted” by artifacts as just one of many further consequences. So clearly, resolving the basic, vertical stratification is essential to any LES of stellar convection.

If we consider instead the velocity field, issues may be more subtle. Sections 2 and 4 of Kupka (2009b) deal with the question why *observations* of solar granulation reveal rather laminar looking structures and what resolution is necessary to actually resolve turbulence caused by the shear between up- and downflow, i.e., the granules and the intergranular network of downflows, on the computational grid. To summarize and extend that discussion let us bear in mind that the horizontal resolution of solar observations is at best ${\sim }35$ km as achieved in the SUNRISE experiment (Barthol et al. 2008). At such length scales the gas becomes optically thin in the photosphere. This also limits the vertical observational resolution in a way that conclusions via spectroscopy can only be drawn from comparisons of *different* spectral lines formed at *different photospheric depths*.

As is also argued in Kupka (2009b), in the solar photosphere small scale ($l \sim 10$ km) temperature fluctuations have cooling time scales of ${\sim }0.1$ s. Note that this is often smaller than $\varDelta t$ of a simulation with that grid size (!). Hence, intensity fluctuations at that level are smoothed out due to strong radiative cooling and at such scale lengths also the contributions of velocity fluctuations to Doppler broadening have to remain small: this is just the length scale on which the *effective viscosity* $\nu _\mathrm{eff}$ of the simulation acts (originating from either numerical, artificial, or subgrid scale viscosity, see Pope 2000 and Sect. 2.1.1) and even for 3D LES of moderate resolution it is *well below the spatial resolution of observations*. Consequently, such hydrodynamical simulations can already fully explain the observed spectral line profiles (cf. Nordlund et al. 2009). 3D LES with grid refinement have achieved a maximum resolution of about 3 km thus far (Muthsam et al. 2011) (which permits recognizing “structures” down to the level of ${\sim }6$ km). At that resolution the vorticity is clearly that of a highly turbulent flow (compare Muthsam et al. 2010, 2011) which extends the results of 3D LES of moderate resolution of 15 km vertically and 25 km horizontally, where vorticity tubes had been found to form in downflows (Stein and Nordlund 2000).

We conclude that the resolution necessary for a 3D LES of stellar convection depends on the physical problem which is investigated. While stellar spectra may be well reproduced with moderate resolution LES which thus have a large $\nu _\mathrm{eff}$ due to their relatively coarse grid, other physical phenomena are sensitive to a proper resolution also of smaller scales. An example is mixing, particularly into neighbouring, “stably” stratified layers.

In Sect. 4.2 of Kupka (2009b) an estimate for the necessary resolution to observe shear driven turbulence on a grid as used for LES of solar surface convection is made and yields values of $h \sim 4$ km which currently is achievable only in simulations with grid refinement (see Fig. 13 and Sect. 5.6.1). At lower resolution the energy carrying and the dissipating scales overlap and such simulations critically rely on the assumption that the basic model of turbulent viscosity (manifesting itself as numerical viscosity, hyperviscosity, or subgrid scale viscosity, e.g.) properly represents the volume averaged action of unresolved scales on resolved scales. The indications of turbulence generated in strong downdrafts as discussed in Stein and Nordlund (2000) thus have a model dependence which is acceptable for predictions of many solar properties (cf. Stein and Nordlund 2000; Nordlund et al. 2009), but can eventually be confirmed only by simulations of higher resolution as just mentioned, since observational tests can be insensitive to such flow properties. Is the situation any different for the bottom of the solar convection zone? In Sect. 6 of Kupka (2009b) the problem of the Peclet number has been discussed in this context. We extend this argument by taking into account our considerations on feasible simulation grids such as $N_\mathrm{tot}(\mathrm{minimal})$ introduced in Sect. 2.2. Due to its large grid size this would lead to a completely unrealistic Peclet number at the bottom of the solar convection zone and thus would *not* predict the correct amount of overshooting into the stably stratified layers underneath it as a result of improperly accounting for the effects of heat exchange on the local flow. This can be understood from the following considerations.

The Peclet number is used to quantify the importance of convective in comparison with conductive heat transport and is frequently defined as the product of Reynolds and Prandtl number: $\mathrm{Pe} =\,\mathrm{Re}\cdot \,\mathrm{Pr} = (U L / \nu )\cdot (\nu /\chi ) = (U L / \chi )$. Here, *L* is to be taken as the typical length at which most of the kinetic energy is being transported and $U=U(L)$ is the velocity at that scale. With *U* in the range of $10\ldots 100\,\mathrm{m}\,\mathrm{s}^{-1}$ (cf. Table 6.1 in Stix 1989) and *L* in the range of several tenth of $H_p$ to $1 H_p$ (which is about 50,000 km close to the bottom of the convection zone in standard solar models) and $\chi \sim 10^7\,\mathrm{cm}^{2}\,\mathrm{s}^{-1}$ following from values of $\nu$ and Pr mentioned above, we have that Pe is in the range of several $10^5$ to $5\times 10^6$. That is quite different from the top of the solar convection zone (Kupka 2009b) where Pe is found to be around 10, as can be obtained from the data given in Sect. 2.2.1, whence $\chi \sim 10^{11}\ldots 10^{12}\,\mathrm{cm}^{2}\,\mathrm{s}^{-1}$, and from taking $L\sim 1200$ km and $U(L) \sim 3\,\mathrm{km}\; \mathrm{s}^{-1}$ (see Sect. 2.1 and 6 of Kupka 2009b). A numerical simulation with an effective viscosity $\nu _\mathrm{eff}$ can achieve an effective Peclet number $\mathrm{Pe}_\mathrm{eff} =\,\mathrm{Re}_\mathrm{eff} \cdot \mathrm{Pr}_\mathrm{eff} = (U L / \nu _\mathrm{eff}) \cdot (\nu _\mathrm{eff} / \chi )$. This holds for both direct numerical simulations of overshooting with idealized microphysics such as those of Hurlburt et al. (1994), Muthsam et al. (1995) and Brummell et al. (2002) as well as those with a more realistic microphysics as described in Brun and Toomre (2002) and Miesch (2005). As shown in Kupka (2009b) such simulations can hardly exceed $\mathrm{Re}_\mathrm{eff} \sim 1500$ and given that $\mathrm{Pr}_\mathrm{eff} \lesssim 0.1$ to avoid viscosity strongly influencing the process of heat exchange of plumes, e.g., with their environment and other flow properties, we thus have $\mathrm{Pe}_\mathrm{eff} \sim 150$ at best for high resolution, state-of-the-art 3D numerical simulations of overshooting. As a consequence, $\mathrm{Pe} \approx \mathrm{Pe}_\mathrm{eff}$ for LES of convection in stellar atmospheres whereas $\mathrm{Pe} \gg \,\mathrm{Pe}_\mathrm{eff}$, if the same technique is applied to the case of overshooting below the solar convection zone. In Brummell et al. (2002) the strong dependence of overshooting on $\mathrm{Pe}$ is demonstrated in their simulations. The profiles of vertical velocity fluctuations and entropy change in a way which cannot be reproduced by a linear or exponential fit function, but requires clearly a non-linear one.

This has consequences also for models of convection which are calibrated with or motivated by numerical simulations. The application of a model of overshooting probed at the low $\mathrm{Pe}$ of stellar atmospheres (Freytag et al. 1996; Ludwig et al. 2002; Tremblay et al. 2015) to the case of stellar interiors (Herwig 2000), which through implementation into the MESA code (Paxton et al. 2011) has found widespread use (e.g., Moore and Garaud 2016), is thus an extrapolation over many orders of magnitudes from the low $\mathrm{Pe}$ into the high $\mathrm{Pe}$ regime. Given the experience from direct numerical simulations in 3D such as Brummell et al. (2002) this is hence a phenomenological procedure which no longer can claim to be solely based on hydrodynamical simulations. In more realistic simulations of the deep solar convection zone as described by Brun and Toomre (2002) and Miesch (2005) the resolution is not far from that one of $N_\mathrm{tot}(\mathrm{minimal})$, which in turn has a local resolution of only $0.1 H_p$ whereas the extent of the overshooting zone is supposed to be just a fraction of that distance (Basu et al. 1994; Monteiro et al. 1994; see also the upper limit provided by Roxburgh and Vorontsov 1994). It is thus not surprising that no 3D LES with a realistic account of overshooting below the solar convection zone exists. The grid underlying $N_\mathrm{tot}(\delta _\mathrm{surface})$ would probably be sufficient to solve this problem, but it is unaffordable with current computational resources (see Sect. 2.6.2). This can be estimated from $\mathrm{Pe}_\mathrm{eff} =\,\mathrm{Re}_\mathrm{eff} \cdot \mathrm{Pr}_\mathrm{eff}$ with $\mathrm{Pr}_\mathrm{eff} \lesssim 0.1$ and $\mathrm{Re}_\mathrm{eff} \approx (L/h)^{4/3}$ (see Sect. 4 in Kupka 2009b) for a simulation with grid spacing *h* at a length scale *L*. Equidistant grid spacing and requiring $\mathrm{Pr}_\mathrm{eff} \approx 0.1$ thus leads to $3\times 10^4 \lesssim N \lesssim 6\times 10^5$ per direction for reaching $10^5 \lesssim \,\mathrm{Pe}_\mathrm{eff} \lesssim 5\times 10^6$. Values of *h* are then between $\min (\delta _\mathrm{surface})/2 \sim 15\,\mathrm{km}$ and an intimidating ${\approx } 1\,\mathrm{km}$ or less. But what happens if the “correct” values for $(U L / \chi )$ are achieved for larger grid spacing? In that case the momentum diffusivity of the numerical scheme (whether be due to artificial diffusion, subgrid-scale viscosity, numerical viscosity, or the like) exceeds radiative diffusivity which in the region of interest, at the lower boundary of the solar convection zone, transports most of the energy flux. Systematic differences to a simulation with sufficient resolution could not be excluded, because such a low resolution simulation would have $\mathrm{Pr}_\mathrm{eff} > 1$ or even $\mathrm{Pr}_\mathrm{eff} \gg 1$.

### Reducing dimensions: 2D simulations as an alternative to 3D ones?

Already the first attempts of numerically solving the hydrodynamical equations in the 1960s have involved the idea of reducing the number of spatial dimensions from three to two (Smagorinsky 1963; Lilly 1969). Later on it has also been used in the study of convection in astrophysics (e.g., in Hurlburt et al. 1984; Sofia and Chan 1984; Freytag et al. 1996; Muthsam et al. 2007; Mundprecht et al. 2013; Viallet et al. 2013; Pratt et al. 2016). The main motivation behind it is naturally the reduction of the computational complexity of the problem.

How much resolution can we gain when performing a 2D LES instead of a 3D one with $N_x = N_y = N_z = 1000$ grid points per direction and a time step $\varDelta t$ determined by Eq. (11)? If we increase the resolution from $h = \varDelta x = \varDelta y = \varDelta z$ to $\xi = h / 10$, we have to decrease $\varDelta t$ to $\tau = \varDelta t / 10$. *So we only gain an order of magnitude in resolution by switching from a 3D simulation to a 2D one*. It is straightforward to see from Eq. (12) that the situation is worse, if transport by diffusion is not taken care of by implicit time integration methods: this allows only for $\xi = h / \root 4 \of {1000} \approx 0.1778\,h$, as a time step $\tau = \varDelta t / \sqrt{1000} \approx 0.03162 \varDelta t$ would take its toll. Of course, for some applications this gain might be crucial. But a resolution of $\xi$ instead of *h* can also be obtained by grid refinement as implemented into the ANTARES code (Muthsam et al. 2010) (see also Sect. 5.6.1). As the 3D geometry can be kept, this approach is preferable to a reduction of dimensions whenever applicable. *If instead the spatial resolution is left unaltered, one can increase the maximum interval for time integration by three orders of magnitude by performing a 2D simulation instead of a 3D one*. Thus, $N_t$ as defined in Eq. (20) can be increased from a range of $10^5$ to $10^8$ to a range of $10^8$ to $10^{11}$. This may be decisive when studying the time development of stellar oscillations and their interaction with stellar convection for stars such as Cepheids. If both higher resolution and longer time integration are required, it may be necessary to combine a 2D LES with grid refinement as in Mundprecht et al. (2013).

However, this computational gain has a price tag: a change of the physics is induced by restricting the motion to only two instead of three dimensions. This is of importance in particular, if the problem to be studied by the numerical simulation has one of the following properties:

- It involves symmetry breaking with respect to the horizontal coordinate. In most 2D simulations the vertical (or radial) coordinate is not removed from the problem, so the dimensional reduction occurs for one of the horizontal coordinates. Rotation allows a distinction between polar and azimuthal flow, so discrepant results are to be expected for rotating systems.
- Magnetic fields occur. Magnetohydrodynamics deals with inherently 3D phenomena.
- The small scale structure of the flow is important.

The latter is especially important for turbulent flows including stellar convection. Despite the observed flow patterns of the latter are usually laminar at accessible observational resolution and the occurrence of turbulence is not a property of the convective instability but rather a consequence of the flow it causes (cf. Sect. 2 of Kupka 2009b), turbulence is one reason for small scale structures to occur in a flow which make 2D and 3D simulations differ when compared to each other.

It has however been argued that two-dimensional turbulent flows are of interest to physics in general for the following reasons (Chap. 8.8 in Tsinober 2009): it may be useful to treat turbulence in *quasi-two-dimensional systems* such as large-scale geophysical flows. Secondly, it is more accessible to statistical physics. And thirdly, the process of predominant stretching of the vorticity gradient in two dimensions has some similarity with the process of vortex stretching in three dimensions. But there are also arguments why two-dimensional chaotic flows cannot be considered as turbulent flows (Tsinober 2009, Chaps. 1.2.2, 8.8, and 8.9.1): there is no vortex stretching in the proper sense, no net production of mean strain (Eq. C53 in Tsinober 2009) which is a conserved quantity instead, and no self-amplification of velocity derivatives (such as strain). Thus, not only energy but also enstrophy is conserved in “2D turbulence” (in models of turbulence in 2D this leads to an “inverse cascade of turbulent kinetic energy”, towards larger scales, cf. Lesieur 1997). As a consequence, in two-dimensional flows large-scale “vortices” are produced out of small scale structures. Indeed, these structures are well-known also from numerical simulations of stellar convection in two-dimensions. We specifically refer to Muthsam et al. (2007) as an example, since due to the very high resolution of a grid cell size of less than 3 km of their simulations, the three-dimensional counterpart of such simulations is clearly in the turbulent regime because of the shearing stresses between up- and downflows (Kupka 2009b). 2D simulations also show much stronger shock fronts than 3D simulations at comparable resolution.

In a direct comparison between 2D and 3D direct numerical simulations of compressible convection for idealized microphysics it has been observed (Muthsam et al. 1995) that the 2D simulations lead to larger overshooting zones—mixed regions next to the convectively unstable layers themselves—and that also higher velocities are needed to transport the same amount of energy flux. As a result, if a high level of accuracy such as in line profile and abundance determinations of stellar photospheres is required, quantitative (and even qualitative) differences can be observed (Asplund et al. 2000).

It should thus be kept in mind that 2D LES cannot replace 3D LES, if the turbulent nature of the flow and the detailed geometrical structure of the flow are important or if high quantitative accuracy is needed. For instance, following comparisons between 2D and 3D direct numerical simulations of convection with a composition gradient of an active scalar (i.e., a gradient of helium in a mixture of hydrogen and helium in the case of a star), it was found that while layered semi-convection may well be investigated and quantitatively be described by 2D simulations (Moll et al. 2016), this is not the case in the fingering regime (Garaud and Brummell 2015). The latter can appear when the composition gradient drives the convective instability and is counteracted by the temperature gradient, which is just the other way round for layered semi-convection. The fingering regime is characterised by small-scale structures as opposed to extended layers, so this difference is intuitive, but in general, this may be realized only in hindsight. Thus, while 2D LES can be used as a tool for pioneering research, care has to be taken once quantitative results are to be predicted, since there *may be unacceptable systematic differences to the full 3D case depending on the physical problem at hand*.

### Computable problems and alternatives to non-computable problems

In the following we summarize this section by a discussion which distinguishes problems in stellar convection modelling which can be dealt with by 3D (or 2D) numerical simulations as computable problems from its “non-computable” siblings for which other means of modelling have to be used.

#### Modelling inherent problems versus the problem of scales

So what are the main limitations to solve a problem in stellar convection modelling?

1. The restriction may be of some basic, physical nature. Examples include incomplete data to describe the microphysical state of the fluid: uncertainties in the equation of state, in opacities, in nuclear reaction rate, and the like. For stars these quantities are now known at least approximatively. In the same realm the proper determination of an initial state may be difficult, for example, for stars with global scale magnetic fields which we can measure through spectropolarimetry only for their surface layers. In that case one either can restrict the problem to physically idealized settings or make trial and error numerical experiments to find out the sensitivity on the initial condition or a lack thereof.
2. The limited computational resources put restrictions on the simulation domain and the resolution in time and space. This introduces the necessity to model the exterior of a simulated domain through boundary conditions, for instance, in global, star-in-a-box-type simulations such as Pratt et al. (2016) but also in local, box-in-a-star-type simulations such as Grimm-Strele et al. (2015a). The spatially unresolved scales are taken care of by some hypothesis such as a subgrid scale model (cf. Smagorinsky 1963; Pope 2000) which is the counterpart of closure conditions in 1D models of stellar structure and evolution (the assumption that numerical viscosity or hyperviscosity takes care of those is just a variant of the same approach). Any numerical simulation can thus cover only a limited interval in time from which conclusions have to be drawn, typically involving arguments of *statistical stationarity* and *quasi-ergodicity* (even though these terms are hardly ever used in publications, at least in the field of astrophysics).

The first type of problems is inherent to modelling: our knowledge of the initial state of the system is incomplete and this remains so for each snapshot in time obtained during a numerical simulation. This cannot be overcome just by improving computing power. The second type of problem is related to the large spread of scales in time and space as observed in turbulent convective flows (Lesieur 1997), particularly in the case of stars or planets, and the physical hypotheses (such as quasi-ergodicity) or models (in the case of boundary conditions) we use to reduce the computational restrictions and thus the computing power required to run such simulations.

#### A list of examples: doable and undoable problems for LES of stellar convection

Given the current state-of-the-art in numerical modelling and in computing technology, we can thus provide a list of examples from hydrodynamical simulations of stellar convection which are “computable” as opposed to some which are not. We explicitly show a number of cases, since unrealistic ideas about what can be computed with LES and what is unaffordable are common.

As a reference we consider a solar granulation simulation which resolves the radiative boundary layer, so $h \lesssim 15\,\mathrm{km}$, for instance, $h \approx 12\,\mathrm{km}$ as in the simulation shown in Fig. 6. As in that example the resolution of the horizontal direction could be lower, i.e., 1 / 3, but for simplicity we take it to be identical. A simulation box with a horizontal width of 6 Mm then requires 500 grid points per direction and with a depth of 4.8 Mm we end up having 400 grid points vertically and thus $N=10^8$ grid points in total. A typical simulation with relaxation and gathering statistical data over between 10 and 20 solar hours (to compute higher order moments or for studying the damping of p-modes) will then take $N_t=10^6$ time steps. Such a task is doable and requires depending on the code, numerics and its effective resolution, number of CPU cores (few dozen to few hundred) and efficiency of parallelization, a few weeks on large department computers or in projects running on national super computers. We assign a complexity number $C=1$ to this problem. Starting from it we now reinvestigate different astrophysical problems related to stellar convection with respect to their computability and collect the results in Table 2.

**Table 2:** A collection of computable (affordable) and non-computable (unaffordable) 2D and 3D numerical simulations of stellar convection

Problem	*N*	$N_t$	C	affordable
3D, solar granules, 6 Mm box	$10^8$	$10^6$	1	Yes
3D, the whole solar surface	$4.2\times 10^{12}$	$10^6$	$4\times 10^4$	Within 20 years?
3D, turb. granules, 6 Mm box	$6.4\times 10^9$	$4 \times 10^6$	256	becoming
3D, turb. whole solar surface	$2.7 \times 10^{14}$	$4 \times 10^6$	$1.1\times 10^7$	Non-computable
3D, low res. 200 Mm box	$8\times 10^9$	$10^6$	80	Yes, at the limit
3D, low res. entire c.z.	$4.5\times 10^{12}$	$5\times 10^8$	$2.25 \times 10^7$	Non-computable
3D, low res. stellar evolution	$4.5\times 10^{12}$	$5\times 10^{18}$	$2.25 \times 10^{17}$	Non-computable
3D, mode damping, entire c.z.	$3 \times 10^{14}$	$5\times 10^9$	$1.5 \times 10^{10}$	Non-computable
3D, subsurface global solar c.z.	$3 \times 10^9$	$10^5$	3	Yes
2D, short period Cepheid, $10^{\circ }$	$10^6$	$10^8$	1	Yes
2D, short period Cepheid, $360^{\circ }$	$3.6\times 10^7$	$10^8$	36	Yes, at the limit
3D, short period Cepheid, $360^{\circ }$	$10^{12}$	$10^8$	$10^5$	Within 20 years?

For different problems we compare number of grid points *N*, number of time steps $N_t$, computational complexity *C* relative to the reference problem, and its computability aspects (rounded values for better readability)

The non-computable cases may of course be accessible one day to quantum computers or other, advanced technology. However, the case of $C\sim 1.5\times 10^{10}$ appears to be too distant for any reasonable prediction and the case of $C\sim 2.25\times 10^{17}$ remains to be extremely unlikely to be accessible for at least this century

Turb., turbulent; res., resolution; c.z., convection zone

How about computing the whole solar surface at this resolution? With $R_{\odot } \sim 695{,}500\,\mathrm{km}$ (Brown and Christensen-Dalsgaard 1998), its area is ${\sim }42{,}300$ times larger than the 6 Mm box just considered. We are thus dealing with $N=4.2 \times 10^{12}$ points and while stationary quantities may be computed with one snap-shot from such a simulation (thanks to quasi-ergodicity), relaxation still requires $t_{\mathrm{rel}}$ as defined in Eq. (21) and likewise the pulsational damping is a time dependent process (even though the statistical sampling is much better in this case). The complexity of this problem is thus $C \approx 40{,}000$. Returning to an argument already discussed in Sect. 2.4, if this simulation should reveal a *turbulent* flow in the sense that the turbulence occurring in the simulation is *generated by shear stresses acting between resolved scales* of the flow and thus is independent of the reliability of numerical viscosity, subgrid scale viscosity, or hyperviscosity to act as models for the *volume average of a flow* simulated at a lower resolution (cf. again Sect. 4.2 in Kupka 2009b), then $h \lesssim 4\,\mathrm{km}$, for example, $h \approx 3\,\mathrm{km}$. Such a high resolution simulation clearly separates energy carrying scales from dissipating ones already through its fine grid, but this increases the number of grid points by a factor of $4^3 = 64$ and that one of time steps by a factor of 4. The complexity level for the solar granules in a box simulation is thus increased from $C=1$ to $C \approx 256$, for the whole surface to $C \approx 1.1 \times 10^7$, which appears non-computable on solid-state based type of hardware. Whether it will one day become accessible to quantum computers (see also Table 2) only time can tell. We recall that for a short time interval (30 min or so) and a single granule this problem is computable *today* thanks to grid-refinement (Muthsam et al. 2011). Is it possible to make a simulation of a “big chunk” of the solar convection zone? Such a 3D LES should contain its upper 10% (or 20 Mm) or so in depth and 100–200 Mm wide: this is already some $8^{\circ }$–$16^{\circ }$ in azimuthal distance and marks the limit doable without introducing unacceptable errors (flux differences between top and bottom of much more than 10%) due to ignoring the approximately spherical geometry of the Sun. With a grid vertically varying in depth according to the pressure scale height (see Sect. 2.2.1) about 500 points may be needed vertically and 2000–4000 points per horizontal direction. If we consider the larger problem only, we have $N=8\times 10^9$. With a similar spatial resolution at the solar surface ($h \approx 12\,\mathrm{km}$), the time steps remain the same except for some longer relaxation due to $t_{\mathrm{conv}}(x_\mathrm{bottom})$, but roughly, $N_t$ remains the same and thus $C=80$. This is just barely computable on the largest present day supercomputers and indeed such calculations are already being done.

Let us now consider simulations of the entire solar convection zone. A very low resolution simulation with grid stretching and refinement might require only about $N \approx N_\mathrm{tot}(\mathrm{minimal}) \sim 4.5\times 10^{12}$ points. Clearly, at $N_t = 10^6$ time steps this simulation would be nowhere near relaxation. Given that solar rotation has a time scale of slightly less then a month, one would expect that the spin-up phase for the differential rotation would be similar to what is observed for the global simulations excluding the actual solar surface (Brun and Toomre 2002; Miesch 2005) which means at least a year, thus $N_t \approx 5\times 10^8$. This does not imply that such a simulation (based only on realistic microphysics and without boosting conductivities, introducing artificial fluxes, etc.) would be thermally relaxed, because guessing the right stratification in the overshooting zone is difficult. We leave this example at this point and conclude its complexity to be $C = 2.25 \times 10^7$. If one were to use such a simulation for “3D stellar evolution”, we have to increase $N_t$ (for a time scale of $10^{10}$ years) to $N_t \approx 5\times 10^{18}$ and $C = 2.25 \times 10^{17}$. Such model would have to include the interior, too, so $C > 10^{18}$. This is for a very low resolution simulation and evidently it is a waste of time to even consider it with semiconductor based computing technology. If one were to use such a simulation to study solar p-modes, a higher resolution would be needed (to truly resolve the dynamics at the surface). In this case, $N_r \approx 800$ still has an optimistically low number of points and we have to consider such an increase of resolution also in horizontal direction, thus $N \approx 3 \times 10^{14}$. To compete with helioseismological observations which span more than a decade one might want to increase $N_t$ to span 10 years and thus $N_t \approx 5\times 10^9$ and for this computation $C = 1.5 \times 10^{10}$. This is why it is completely hopeless to consider a “realistic simulation of *global* solar convection and pulsation” which contains the *whole* solar convection zone in the simulation domain. It is also very simple to see that a direct (all scale resolving) numerical simulation of the solar convection zone is even further beyond reach. But then why is it that *“global simulations of the solar convection zone are computable”*? The key is that *they leave out the surface layers* (see the review in Miesch 2005). With some 800 points vertically one may cover the rest of the convection zone plus overshooting underneath. Since the scales are large, an angular resolution of ${\approx } 0.2^{\circ }$ or some 2000 points can already give acceptable results, thus $N \approx 3 \times 10^9$. The anelastic approximation used in this field (Miesch 2005) (see Sect. 4.3.2) filters out the sound waves and advective velocities are much smaller than at the stellar surface which allows for much larger time steps. Circumventing the relaxation problem, this can push $N_t$ down to $N_t \approx 10^5$ and thus $C=3$. Even if the numerics may be more involved, such a computation is readily doable with present resources (radiative transfer can always be treated in the diffusion approximation which eases the computational costs compared to simulations of stellar surface convection).

We now briefly turn to the problem of 2D LES of Cepheids as performed by Mundprecht et al. (2013, 2015). The 2D framework requires a significantly lower computational effort than its 3D counterpart. However, time steps have to be small (shock fronts, strong radiative losses), while integration times have to be long. As it turns out, with grid refinement a simulation with $N \approx 10^6$ allows a good width of the simulation (${\approx } 10^{\circ }$) as well as a depth covering the region from the surface to the first radial node (below the surface at a distance of 42% of the stellar radius or some 11 Gm in the model of Mundprecht et al. 2015, who, however, had a smaller grid and a lower resolution for the stellar surface). The grid stretching used in this simulation (surface cells more than 100 times smaller than near the bottom) has its toll on the time step. Moreover, a sufficiently large number of pulsation periods has to be calculated (one to several dozens) following an equally long relaxation. Unless radiative transfer is integrated implicitly, one thus has $\varDelta t \sim 0.2~\mathrm{s}$ and if 60 pulsation periods of about 4 days are to be covered, we have $N_t \approx 10^8$ and $C \approx 1$ for this problem. We note that semi-implicit time integration methods could help to accelerate this calculation by an order of magnitude, but not much more than that (since a larger time step is traded for solving a large, coupled system of non-linear equations each time step). If we repeat this calculation for a full circle, the workload increases by a factor of 36 (in *N* and in *C*). A 3D version of that calculation (which assumes the equivalent of 36,000 points in the azimuthal direction) would require $N \approx 10^{12}$ while $N_t$ stays the same, thus $C \approx 10^6$ and accounting for the success of semi-implicit methods, $C \approx 10^5$. But how about if we were to follow a long period Cepheid and resolve the variations of pulsational amplitudes as observed for Polaris? If we were to follow our sample 2D calculation as just explained over, say, 400 stellar years, we are already at $C \approx 600$. However, for a long period object, resolution requirements are clearly more extreme, time steps more restrictive, so $C \approx 10^5$ easily, and this is not yet a $360^{\circ }$ calculation, let alone a 3D one. Clearly, it is completely unrealistic to seriously plan such a calculation at the moment.

Thus, in considering problems for hydrodynamical simulations of stellar convection, it is very easy to switch from a perfectly doable project to discussions of a completely unrealistic one. Numerical simulations of this kind are at the cutting edge of computational technology and while some problems are now standard problems and others well within reach, many problems in the field remain incomputable with this approach.

#### The continued necessity of 1D models of stellar convection

One might claim that computer technology advances exponentially, but this is an extrapolation based on the number of circuits per area in a technology for which some barriers appear to have been reached (clock frequency) while others are not so far away any more (size of computing elements, with quantum effects starting to appear already when reducing the current 14 nm process to a 5 nm one, etc.). A naive extrapolation of computing speed also ignores that the more and more massive parallelization the current development of computing technology requires is indeed becoming increasingly challenging to make full use of and hence, the estimates from Sect. 2.6.2 should be fairly robust when allowing for uncertainties in achievable computational complexities *C* within one (or at the very most two) orders of magnitude. As a consequence, there is no way we can abandon 1D models of stellar convection now or during the next few decades, since we still require them in applications inaccessible to 3D or even to 2D LES now and definitely for many years to come. In the next section we thus discuss some of the challenges faced by one dimensional modelling.

## One dimensional modelling

There is no complete statistical or any other low-dimensional description of turbulent flows which can be derived from the basic hydrodynamical Eqs. (1)–(10). A rather detailed and well accessible introduction into the state-of-the-art of modelling turbulent flows can be found in Pope (2000). *None of the known approaches yields a closed, self-contained model without introducing additional assumptions, hypotheses which cannot be strictly derived from the basic equations alone.*

One-dimensional (1D) model of turbulent convection are based on the assumption that it is possible to predict the horizontal average of physical quantities such as temperature *T* or density $\rho$—without knowing the detailed time evolution of the basic fields $\rho , \varvec{\mu }= \rho \varvec{u}$, and $e = \rho E$—as a function of location *x* and time *t* for different *realizations* of the flow, i.e., initial conditions. They hence result from a double averaging process: one over the horizontal variation of the basic fields or any dependent variable such as *T* and a second one over a hypothetical *ensemble* of (slightly different) initial conditions. So the horizontal averaging is an additional step, since ensemble averaged model equations may also be constructed for the three-dimensional (3D) case.

The *quasi-ergodic hypothesis*, which underlies also the interpretation of any numerical simulation of stellar convection or in fact any other turbulent flow, assumes that *the time average of a single realization*, which is given by one initial condition, *is equal to an average over many different realizations* (obtained through different initial conditions) at any time *t* in the limit of averaging over a large time interval and a large ensemble (Pope 2000). This cannot be proven to hold for all flows since in particular there are also known counterexamples, but for some flows such as statistically stationary (time independent), homogeneous (location independent) turbulent flows it can be corroborated even directly from numerical simulations (Chap. 3.7 of Tsinober 2009).

It is thus not a completely hopeless enterprise from the very beginning to construct 1D models of turbulent flows and indeed there are well-known, simple flows for which models have become available that are sufficiently accurate in practice (cf. Pope 2000). It is of course a different story to what extent it is possible to succeed in these efforts in the case of turbulent convection in stars or in planets (interior of gaseous giant planets, oceans and atmosphere at the surface of terrestrial planets). Instead of deriving one model in detail or advertising another, in the following we discuss some principles which should be taken into account when applying published models or when comparing them to each other, to numerical simulations, or to observational data.

### Requirements for models of turbulent convection

As also pointed out in Zilitinkevich et al. (1999) and Gryanik et al. (2005), any physically consistent parametrization or closure hypothesis introduced in modelling turbulent flows should fulfill the following properties:

1. correct physical *dimension*;
2. *tensor invariance*—this refers to higher order correlations constructed from products of functions or any derivatives thereof. Especially with respect to the latter, if an approximation is to be used in coordinate systems other than a Cartesian one, a co-variant form of the hypothesis may even be required;
3. respecting *symmetries*, particularly concerning sign changes of variables;
4. physical and mathematical *realizability* of the approximation.

While requirement 1 is straightforward, properties like invariance to sign change of involved variables can be a more subtle issue. Mironov et al. (1999) discuss the consequences for a third order correlation, $\overline{w' \theta '^2}$, where $w'$ is the difference of the vertical velocity and its horizontal average and $\theta '$ is the same type of difference for the case of temperature. If ensemble (or actually time) averages of this quantity are computed from either numerical simulations or measurements of a convective zone, a change of sign $w' \rightarrow -w'$ implies $w' \theta '^2 \rightarrow -w' \theta '^2$. Mironov et al. (1999) show how a closure hypothesis which ignores this symmetry fails in describing this *flux of potential temperature*, $\overline{w' \theta '^2}$, in the transition region between convectively stable and unstable stratification as opposed to a superficially similar one which actually does respect that symmetry. Hence, requirement 3 is important. Another crucial issue is realizability: if a hypothesis is non-realizable, the probability of finding, for instance, velocity and temperature fields which correspond to the modelling expression, is actually *negative*, i.e., mathematically impossible. An example is the assumption of a supposedly quasi-normal distribution function which by definition has a kurtosis $K_w:= \overline{w'^4}/ \overline{w'^2}^{2}$ of 3 that is also claimed to be highly skewed. For instance, let $S_w > \sqrt{2}$, where $S_w := \overline{w'^3}/ \overline{w'^2}^{1.5}$ (see Gryanik et al. 2005 for details and also André et al. 1976a, b for further references). But a distribution function with $K_w = 3$ and $S_w > \sqrt{2}$ is non-realizable, hence, such a model of convection cannot be physically meaningful. We note that for a compressible flow it is more natural to consider density weighted (or Favre) averages (see Sect. 3.3.1), but in practice realizability also has to hold for the plain Reynolds average assumed. A model failing on requirement 4 is physically useless and mathematically meaningless. Requirement 2 is probably the most often violated one of these four and its consequences may show up only, once the approximations are supposed to hold in polar instead of Cartesian coordinates.

To check these requirements is hence useful to determine the physical and mathematical consistency of a model or detect limitations of the region of applicability of a model.

### Phenomenological approach

As stated in Chap. 5 of Tsinober (2009), there is no commonly accepted definition of a phenomenology of turbulence. In a strict sense, it may refer to anything except direct experimental results, direct numerical simulations (with all spatial and time scales of interest resolved), and the small set of results which can be obtained from first principles (Tsinober 2009), i.e., Eqs. (1)–(10). More commonly, models of turbulent flows are called phenomenological, if they introduce a concept such a rising and falling bubbles which cannot be derived directly from (1)–(10) nor at least confirmed by experiment or numerical simulation, but which is used for deriving the mathematical expressions of the model. Thus, in Canuto (2009) the well-known mixing length treatment or mixing length theory (MLT) of convection is considered a phenomenological model and indeed following the derivation of Weiss et al. (2004) it is clear MLT deals with the properties of fictitious bubbles that are not observed in convective flows anywhere (Sun, Earth atmosphere and oceans, laboratory experiments of convection, or numerical simulations of convection in these objects).

However, since this kind of modelling has been accessible to scientists already decades ago and the most crucial free parameter of the model, the mixing length relative to a reference scale (most frequently the local pressure scale height), provided enough flexibility to adapt the predictions of the model to different physical situations, it has become the workhorse of astrophysical convection modelling already in the 1960s, when the first edition of Weiss et al. (2004) was written. This situation has not changed since those days, which is unfortunate, as we discuss in the following.

#### Models and physics

At the heart of any of the phenomenological models, but also of more advanced models of turbulent flows, is the concept of *turbulent viscosity*. Introduced by Boussinesq (1877) its idea is to model the Reynolds stress of a flow to be proportional to the mean rate of strain (see Chap. 4.4 in Pope 2000), as if the (main) effect of turbulence on the velocity field is just to boost the kinematic viscosity $\nu$ up to an *effective viscosity* $\nu _\mathrm{eff} = \nu + \nu _\mathrm{turb}$. A related and very similar idea is that of *turbulent diffusivity*, a generalization of Fick’s law of gradient diffusion, where turbulence induces an *effective diffusivity* of a conserved scalar $\phi$, i.e., $\chi _\mathrm{eff} = \chi + \chi _\mathrm{turb}$ and thus $\overline{\varvec{u} \phi '} = -\chi _\mathrm{turb} \nabla {\overline{\phi }}$. However, while the conditions of validity are well understood for the case of diffusion due to molecular motion, where the mean free path is small against the variation of the gradient of the “driving quantity”, such as temperature for the case of heat diffusion, and thus a first order Taylor expansion applies also on mathematical grounds, this is usually not the case for turbulent diffusivity and turbulent viscosity. Hence, these quantities are quite different from their “molecular counterparts” and should be understood as *physical models* to describe data.

The computation of turbulent viscosity is thus *model dependent*, even if measurements or a direct numerical simulations were at hands. Thus, care should be taken not to confuse the underlying physical processes with a concept that is actually a model on its own (cf. Tsinober 2009).

#### Mixing length treatment

One way to compute turbulent viscosity involves using a mixing length. Indeed, this is just what the mixing length had been invented for by Prandtl (1925). Discussions and illustrations how the idea of a mixing length is motivated by velocity profiles of turbulent channel flow can be found in Chaps. 7.14, 7.1.7, and 10.2.2 of Pope (2000). The “interior” region of such a flow is separated from the solid wall, which acts as a boundary condition, by a so-called viscous boundary layer. Contrary to a uniform turbulent viscosity $\nu _\mathrm{turb} = f(x)$, which varies only a long the direction *x* of the mean flow, the mixing length allows modelling how $\nu _\mathrm{turb}$ varies across the flow, as a function of distance from the boundary of the domain.

Biermann (1932) then used this idea, among others, to model the heat transport by convection inside a star. Following the notion by Unsöld (1930) that the solar photosphere must be unstable to convection due to the lowering of the adiabatic gradient by partial ionization, Siedentopf (1933) realized that all stars with $T_\mathrm{eff} \lesssim 10{,}000~\mathrm{K}$ must have convection up to their observable surface and that the newly invented treatment of convection can explain solar granulation (Siedentopf 1935). Through a number of improvements (Biermann 1942, 1948; Vitense 1953) the model eventually obtained its form suggested by Böhm–Vitense that is used even today (Böhm-Vitense 1958; Weiss et al. 2004). Those improvements were essentially devoted to account for the radiative heat loss of the fluid which was usually depicted as consisting of moving bubbles that exchange heat with their environment.

In its most compact form (cf. Heiter et al. 2002) the convective flux of a stationary, local convection model such as MLT is computed from

22 $$\begin{aligned} F_\mathrm{conv} = K_\mathrm{turb} \beta = K_\mathrm{rad} T H_p^{-1} (\nabla -\nabla _\mathrm{ad})\varPhi (\nabla -\nabla _\mathrm{ad},S) \end{aligned}$$

for regions where

23 $$\begin{aligned} \nabla > \nabla _\mathrm{ad}, \quad \hbox {with}\quad \nabla =\partial \ln T / \partial \ln P \quad \hbox {and}\quad \nabla _\mathrm{ad}=(\partial \ln T / \partial \ln P)_\mathrm{ad} \end{aligned}$$

i.e., the linear, local criterion for convective instability by Schwarzschild (1906) must hold (the adiabatic temperature gradient follows from the equation of state, see Weiss et al. 2004). Outside those regions it is assumed that $F_\mathrm{conv} = 0$ (no overshooting of flow into “stable” layers). We note that in this version the criterion Eq. (23) ignores the counteracting, stabilizing effect of viscous friction, which at stellar Prandtl numbers in any case is negligibly small. Further details on local stability criteria can be found in Kippenhahn and Weigert (1994) and Weiss et al. (2004). The radiative conductivity $K_\mathrm{rad}$ has already been introduced in Eq. (5), and as before $H_p$ is the pressure scale height, whereas $\varPhi = K_\mathrm{turb} / K_\mathrm{rad}$ is the ratio of turbulent to radiative conductivity, *P* is the (gas) pressure, and *T* is the temperature. The total energy flux $F_\mathrm{tot}$ in turn follows from $F_\mathrm{rad} + F_\mathrm{conv} = F_\mathrm{tot}$ under the assumption that $F_\mathrm{kin} = 0$. Finally,

24 $$\begin{aligned} \beta = -\left( \frac{dT}{dr}-\left( \frac{dT}{dr}\right) _\mathrm{ad}\right) = T H_p^{-1} \left( \nabla -\nabla _\mathrm{ad}\right) \end{aligned}$$

is the superadiabatic gradient, a function of radius *r* (or depth *z*) and the thermodynamically determined adiabatic gradient $\nabla _\mathrm{ad}$. The convective efficiency *S* is just the product of Prandtl and Rayleigh numbers, $\mathrm{Ra}$ and $\mathrm{Pr}$, and reads

25 $$\begin{aligned} S =\,\mathrm{Ra}\cdot \mathrm{Pr} = \frac{g\alpha _\mathrm{v}\beta l^4}{\nu \chi }\cdot \frac{\nu }{\chi }, \end{aligned}$$

where *g* is the local surface gravity, $\alpha _\mathrm{v}$ is the volume expansion coefficient, $\nu$ the kinematic viscosity and the radiative diffusivity $\chi$ follows for known $c_p$ from $K_\mathrm{rad} = c_p \rho \chi$ (see Table 1). Note that *S* only depends on buoyancy and radiative diffusion, a useful parametrization, since in stars viscous processes act on much longer timescales than either radiation or buoyancy and hence convection. In the case of MLT, the function $\varPhi (S)$ is given by $\varPhi (S) = \varPhi ^\mathrm{MLT}$ as

26 $$\begin{aligned} \varPhi ^\mathrm{MLT} = \frac{729}{16} S^{-1} \left( \left( 1 + \frac{2}{81} S\right) ^{1/2}-1\right) ^3, \end{aligned}$$

where *S* is computed from

27 $$\begin{aligned} S = \frac{81}{2} \varSigma , \quad \varSigma = 4 A^2 (\nabla -\nabla _\mathrm{ad}), \quad A = \frac{Q^{1/2} c_p \rho ^2 \kappa l^2}{12 a c T^3} \sqrt{\frac{g}{2 H_p}}, \end{aligned}$$

and $Q = T V^{-1}(\partial V/\partial T)_p = 1-(\partial \ln \mu / \partial \ln T)_p$ is the variable, average molecular weight. For this compact form of writing the MLT expression of the convective flux we refer to Canuto and Mazzitelli (1991, 1992) and Canuto et al. (1996) who point out its equivalence with the variant of MLT introduced by Böhm-Vitense (1958). Indeed, this notation and its compact formulation have already been used in much earlier work such as Gough (1977a) as well as in later work (Heiter et al. 2002, e.g.). Finally, *l* is the *mixing length*, usually parametrized as

28 $$\begin{aligned} l = \alpha H_p, \end{aligned}$$

and the mixing length scale height parameter $\alpha$ is calibrated by comparison with some data. Clearly, $F_\mathrm{conv}$ as computed from Eqs. (22)–(28) is just a function of local thermodynamic quantities, the difference of the local and the adiabatic temperature gradient, and the mixing length *l*. It is akin to a diffusion model, $F_\mathrm{conv} = K_\mathrm{turb} \beta$, similar to the radiative flux $F_\mathrm{rad}$ in stellar interiors, Eq. (4).

This phenomenological approach to compute $K_\mathrm{turb}$ is now quite different from the mixing length as used in engineering sciences for shear flows. Prandtl (1945) and Kolmogorov (1942) independently from each other realized that in the approach of computing $\nu _\mathrm{turb} = u l_\mathrm{m}$, the reference velocity *u* should be related to the turbulent kinetic energy *K* instead of the mean velocity gradient times the mixing length. Thus, $u = c K^{1/2}$ and $\nu _\mathrm{turb} = c K^{1/2} l_\mathrm{m}$. Through Kolmogorov’s similarity hypotheses the mixing length is then related to the dissipation rate $\epsilon$ of turbulent kinetic energy via $u(l)=(\epsilon l)^{1/3}$, whence

29 $$\begin{aligned} \epsilon = c_{\epsilon } K^{3/2} / l_\mathrm{m} \end{aligned}$$

(see Chaps. 6.1.2 and 10.3 in Pope 2000, for convenience we have used the notation of Canuto 1993; Canuto and Dubovikov 1998 here; *c* and $c_{\epsilon }$ are model parameters). In engineering problems the mixing length $l_m$ (to be specified for each case) is used alongside a differential equation for *K*. This is hence a *non-local* model, as it explicitly accounts for the fact that turbulent kinetic energy (TKE) is also transported by the flow itself and it models this process through a differential equation. Since it requires only one such equation in addition to algebraic ones, this approach is called a *one-equation model* (Chap. 10.3 in Pope 2000). Purely algebraic models for $\nu _\mathrm{turb}$ only work for rather simple types of flows and the original prescription of the mixing length model already fails for decaying grid turbulence or the centerline of a round jet, as the mean velocity is constant across the flow in that case and thus $\nu _\mathrm{turb}$ is mispredicted as zero (Pope 2000).

However, even the one-equation model is outdated in engineering applications of fluid dynamics. Most commercial computational fluid dynamic codes use the *K*–$\epsilon$ *two-equation model* (Jones and Launder 1972) as their basic tool to model turbulent flows (cf. the discussion in Chap. 10.4 of Pope 2000). This approach avoids the computation of a mixing length by specifying a differential equation for the dissipation rate $\epsilon$ (cf. Canuto 1992, 1993, 1997a; Canuto and Dubovikov 1998).

The situation is quite different in astrophysics: *non-local mixing-length models*—to which we shortly return below—are typically used only in studies of stellar pulsation. Unless a problem is accessible to LES the *local, algebraic* MLT model of Böhm-Vitense (1958), as given by Eqs. (22)–(28) above, has remained the most popular way to compute $F_\mathrm{conv}$ in the vast majority of astrophysical applications independently of the fact that this form of modelling has been abandoned in engineering sciences a long time ago. There are several reasons for this resiliency of the MLT model in stellar astrophysics:

- It is the standard model of convection and used in a large number of entire grids of stellar evolution and stellar atmosphere models which in turn are used in other astrophysical applications, for instance, stellar isochrones, photometric calibrations, and grids of stellar spectra for stellar population synthesis.
- It is easy to incorporate into a code and its basic calibration is simple: match the solar luminosity and effective temperature (or radius) at the present solar age. This is always achievable for the different versions of MLT (Gough and Weiss 1976).
- Alternative models are more difficult to calibrate: one has to deal with more free parameters which again lack universality, just as the stellar mixing length does. Note the dependency of mixing length on stellar type, where much smaller scale lengths are required for A-type stars compared to solar type stars (cf. Gough and Weiss 1976 vs. Kupka and Montgomery 2002): *the standard MLT calibration is not universal*, a deficiency usually neglected in stellar modelling, but this also holds for the usually considered more complex models of convection such as non-local MLT.
- Until the advent of sufficiently accurate observations from helioseismology and sufficiently advanced LES, it was difficult to falsify MLT by proving it cannot get the temperature and pressure structure right in a way that cannot be fixed by just tuning $\alpha$.

Indeed, in spite of all its merits as a means of modelling convection in the pioneering days of stellar astrophysics, thanks to the very high accuracy of current observational data and numerical simulations, stellar MLT has now been falsified in several ways and *we see no way to reconcile it other than by ignoring those tests*. The latter all demonstrate MLT cannot correctly predict the convective surface layers of a star so as to recover temperature and pressure profile, sound speed profile, asymmetry between up- and downflows, and kinetic energy available for mode driving with state-of-the-art accuracy. The tests mentioned include:

- Failure to recover solar p-mode frequencies due to mispredicting the temperature and pressure profile (Baturin and Mironova 1995; Rosenthal et al. 1999) which in helio- and asteroseismology is known as the *near surface effect*.
- Failure to predict a sufficiently large rate of driving of solar p-modes (Samadi et al. 2006) in contrast with an approach based on a 3D LES and a closure model with plumes (Belkacem et al. 2006a, b).

This comes along with all the problems found from stellar spectroscopy and photometry (Smalley and Kupka 1997; Gardiner et al. 1999; Heiter et al. 2002; Smalley et al. 2002) let alone if very accurate spectral line profiles as obtainable from 3D LES in Asplund et al. (2000) and Nordlund et al. (2009) have to be computed. From this viewpoint classical MLT has been falsified. That claim holds unless one merely expects the model to provide the correct depth of the surface convection zone and the stellar radius which is given by calibrating $\alpha$ (cf. again Gough and Weiss 1976) and ignores the inconsistencies which the model imposes on predictions for observable quantities that require accurate modelling of stellar surface layers.^4^

Clearly, models beyond local MLT are necessary and *time dependent, non-local MLT models* (Gough 1977a, b; Unno 1967) alleviate some of the problems found with helioseismology, particularly, if both *non-locality and non-adiabaticity* are taken into account, as summarized in the review of Houdek and Dupret (2015). The latter also give a derivation of local, time-independent MLT from a kinetic theory of accelerating eddies point of view (as in Gough 1977a, b), since this can more easily be generalized to the time dependent, non-local case than the alternative approach of Unno (1967), which is discussed there as well. However, it is impossible to avoid mathematical inconsistencies of the following type during the derivation of MLT models, namely that some variables must not vary much over a mixing length *l* while the latter necessarily has to be large to predict the correct solar radius as in Gough and Weiss (1976) and the mentioned variables clearly change along a distance *l*. The same inconsistencies also arise, if a phenomenological analysis of rising and cooling bubbles is made in deriving the model (see Weiss et al. 2004, and references therein), or if the model is formulated just as one imitating heat diffusion with an enhanced, effective diffusivity (cf. Sect. 3 of Canuto 2009), or if it is derived as a one-eddy (delta function) approximation in the context of a more general, two-point closure model of turbulent convection (Canuto 1996).

#### A more recent, parameter-less sibling

In an attempt to remove the need of a mixing length, Pasetto et al. (2014) constructed a model for the convective flux where, as several times before, it is claimed that it *does not depend on any free parameter*.

A word of caution should be given to *any* such claim already now, whether made in favour of a convection model or a numerical simulation: *they all either depend on parameters related to the modelling of the flow or the flow such models consider is so idealized as to have but little relation to any real world flow.* We return to this sobering statement further below.

Indeed, in their derivation, Pasetto et al. (2014) assume the Boussinesq approximation (discussed in Sect. 4.3.1) which is also used in MLT. But from the beginning the idea of *convective elements* is invoked which supposedly travel distances small compared to distances over which temperature, pressure, and density vary significantly so that gradients of these quantities could develop. While this is just the basis for the validity of the diffusion approximation, it is used in Pasetto et al. (2014) together with the Boussinesq approximation to motivate the assumption that convective flow in stars is irrotational and hence a potential flow. Comparing to LES of stellar convection or direct numerical simulation of convection for idealized microphysics, neither of these assumptions can found to be justified: Figs. 15 and 16 of Muthsam et al. (2010) show the norm of vorticity and a volume rendering of the difference between pressure and its horizontal average: clearly, strong vorticity appears especially close to downdrafts (Fig. 15) and even well defined, tornado-like vortex tubes appear in the flow (Fig. 16) already at slightly below 10 km resolution. These vortex tubes penetrate well into upflow regions underneath the solar convection zone, once a resolution of 3 km is reached (cf. Fig. 13). Hence, the assumption of a potential flow for solar convection is completely at variance with LES of solar convection. The flow is clearly turbulent, while the model of Pasetto et al. (2014) excludes turbulence from the beginning.

Although it might still be possible that *statistical averages* predicted from such a model agree with *some of the data* obtained from observations and numerical simulations, there is at least no obvious physical and mathematical basis for the agreement. The simplifications made in Pasetto et al. (2014) to remove the mixing length, an attempt which they indeed succeed in, is paid for by other simplifications: not just the Boussinesq approximation, but also irrotationality (and thus complete exclusion of turbulence) and the introduction of a dynamics of “convective elements” (in the end fluid bubbles akin to MLT) which are heuristically motivated: such features cannot be identified in observations of solar convection or convection in the atmosphere of the Earth or in numerical simulations of these based on the fundamental Eqs. (1)–(10). This limits the tools developed in Pasetto et al. (2014) to predict the ensemble averaged quantities of interest to stellar convection modelling (superadiabatic and mean temperature gradient, etc.) and the region of applicability of the model.

A much stronger, detailed criticism of the model of Pasetto et al. (2014) has recently been published in Miller Bertolami et al. (2016). It addresses the internal consistency of the model, the stringency of the tests the model has passed, and other issues. We suggest the reader to compare the original papers and instead of repeating further details we prefer to repeat the general comment made just above: a “parameter free” description of a flow has so far been only found for flows for which drastic simplifications are assumed to hold for their basic properties. Which price is the lower one to pay (a “parameter free” model or a physically more complete model with parameters that require calibration) is probably best judged by comparisons to observational data and, where possible, advanced hydrodynamical simulations that solve the fundamental Eqs. (1)–(10) provided that the model assumptions made appear acceptable.

#### Non-local mixing length models

The models of Gough (1977a, b) and Unno (1967) are examples of non-local mixing length models. Their detailed derivation and in particular the extensions necessary to use them in the context of radially pulsating stars is discussed in Houdek and Dupret (2015). One particular feature of the model of Gough (1977a, b) is to consider several basic quantities, i.e., the convective flux $F_\mathrm{conv}$, the superadiabatic gradient $\beta$, and the turbulent pressure $p_\mathrm{turb}=\overline{\rho u_3 u_3} =\overline{\rho w^2}$, as averages over vertical distances which accounts for the fact that these clearly change over the typical travel distance of eddies (Sect. 3.3.1 of Houdek and Dupret 2015), contrary to the assumptions of the models discussed in Sects. 3.2.2 and 3.2.3 above. In the end one arrives at a two-equation (second order), non-local model (see also Canuto 1993), where, however, a number of simplifications had to be made to compare the model to a Reynolds stress model of convection). The benefits of this extension when dealing with pulsating stars have already been discussed Houdek and Dupret (2015). But neither the phenomenological style of modelling of the dynamics of *bubbles* or *convective eddies* is avoided this way, nor the introduction of a mixing length itself.

The most advanced generalization of the mixing length approach as used in astrophysics is probably the model by Grossman et al. (1993). They started from the idea of deriving Boltzmann-type transport equations for fluid blobs very similar to the derivation of the fundamental NSE themselves (cf. Huang 1963; Hillebrandt and Kupka 2009). In a next step they arrived at a hierarchy of moment equations which by the nature of their approach and the similarity to the NSE is structurally very similar to higher order moment equations which are derived in the Reynolds stress approach directly from the NSE. On the other hand, for deriving the NSE themselves progressing from the Boltzmann-type transport equations for the distribution functions of microphysical particles to the Maxwell–Boltzmann transport equations, which describe the dynamics on the level of averaged, macroscopic quantities such as the mean particle number density, eventually allows the derivation of the closed system (1)–(3), i.e., the NSE (see Huang 1963; Hillebrandt and Kupka 2009). This is possible thanks to the scale separation between microphysical processes and macroscopic ones. But that is not the case for macroscopic equations which are supposed to describe the dynamics of “fluid particles”: in the end one gets stuck with a large number of terms which cannot be computed from within the model itself unless one constructs a whole set of different scale lengths, mixing lengths for that matter. Thus, while Grossman et al. (1993) can easily rederive the original MLT model and suggest how generalizations accounting for anisotropic velocity fields and a concentration (mean molecular weight) gradient should look like, their approach provides no tools how to close the resulting systems of equations other than by a large set of hypotheses on physical processes occurring over certain scale lengths. The similarities of the resulting moment equations with those obtained in the Reynolds stress approach of Canuto (1992, 1999) are probably indicative for rather investigating the latter at that level of modelling complexity, since the one-point closure turbulence models used in Canuto (1992, 1999) are not tied to the idea of fluid bubbles travelling a number of scale lengths difficult to specify.

#### Further models specifically designed to calculate overshooting

In parallel to the non-local extension of MLT there have been many attempts to develop models of the inherently non-local process of overshooting where layers of fluid locally stable to convection are mixed because of processes going on in layers which are located at some distance and which are unstable in that same sense. The inconsistencies which can arise when combining (and possibly confusing) local concepts with non-local ones, as happened in the derivation of many models of convective overshooting, were heavily criticized by Renzini (1987). We hence only discuss here a few examples of models which have become popular beyond the group of authors who had originally developed them.

An often used model to estimate overshooting above convective stellar cores is the integral constraint derived by Roxburgh (1978). Criticism raised by Baker and Kuhfuß (1987) concerned the neglect of contributions which become important in superadiabatic stratification. This was refuted by Roxburgh (1989) to not apply for the case of stellar cores. Hence, Zahn (1991) suggested Roxburgh’s constraint to be applicable to the case of penetrative convection above convective stellar cores which permitted him to combine it with his own model. Thereby he avoided the external calibration of the geometrical extent of overshooting which in turn remains necessary, when the model of Zahn (1991) is applied to overshooting underneath the solar envelope.

Criticism on the physical completeness of the approach of Roxburgh (1989) was raised again (Canuto 1997b) regarding its neglect of the role of turbulent dissipation for energy conservation. Canuto (1997b) also summarized problematic approximations such as assuming a subadiabatic stratification that is tied to a zero convective flux, negligible superadiabaticity in the overshooting region, negligible kinetic energy dissipation, and the applicability of Eq. (29) to compute the latter, shortcomings to be found in the vast majority of models proposed to calculate overshooting when modelling stellar convection zones.

The model of Zahn (1991) centres around the properties of plumes observed in adiabatic convection which penetrate into stable stratification. In this sense it is related to the mass-flux models used in meteorology to which we turn in Sect. 3.3.1. The applicability of Zahn’s model is restricted to the case of *convective penetration* which occurs for convection at high Peclet number close to adiabatic stratification (in Zahn 1991 the term *overshooting* is used to only refer to the case of low Peclet number and non-adiabatic stratification, to which the model is not applicable). Hence, it can be used to estimate overshooting underneath the solar convection zone or above a convective core of a massive star, but not for the case of convective overshooting in A-stars or hot DA white dwarfs (such as that one shown in Fig. 4). The model requires external information to become fully predictive and is also subject to some of the approximations criticized by Canuto (1997b).

More recently, inspired by the results of numerical simulations Rempel (2004) proposed a model for overshooting based on an ensemble of plumes generated within the convection zone. One problem that remains also with this type of model is the computation of the filling factors of the plumes in a physical parameter space for which a reliable numerical simulation is not available.

A different model inspired by numerical simulations is the notion of an exponential decay of the velocity field in the overshooting region, as originally proposed by Freytag et al. (1996), which we have briefly discussed already in Sect. 2.4. This model is derived from numerical simulations of stellar convection at rather low Peclet number which occurs in A-stars and hot DA white dwarfs. As follows from the discussion in Sect. 2.4 and the physical considerations made in Zahn (1991) and Rempel (2004), the region of applicability of this model is probably restricted to the outermost part of an overshooting zone which is dominated by waves rather than plumes. It remains to be shown whether such a model can be applied to the case of overshooting at the bottom of the solar convection zone and above the convective core of a massive star without resorting to highly phenomenological patching, since the physics of the layers closer to the convection zone motivated the completely different models of overshooting proposed by Zahn (1991) and Rempel (2004).

Clearly, this is not a satisfactory situation. A different route of modelling than the suite of special cases considered here appears to be necessary.

### More general models of turbulent convection

A number of techniques have been developed for the modelling of statistical properties of turbulent flows which are not built on the phenomenological idea of providing an ensemble average for the somewhat vaguely defined concepts of bubbles or blobs of fluid, although we have to add here that the actually available models are based on rather continuous transitions and mixtures of ideas. Blobs and bubbles, however, cannot easily be identified with the coherent structures that are indeed found in turbulent flows (cf. Lumley 1989; Lesieur 1997; Tsinober 2009) and thus provide no real advantage in deriving closure relations despite they might still implicitly be referred to in some of the more advanced models. For general introductions into modelling turbulent flows, we refer to Lesieur (1997) and Pope (2000), and for critical discussions on the techniques used, Lumley (1989) and Tsinober (2009) provide valuable information. We note for the following that some of the work discussed below actually does rely on a mixing length, but in the sense of Eq. (29), i.e., for computing the dissipation rate of kinetic energy. That usage is based on the idea of scale separation and the assumption of an inertial range rather than on the concepts developed for astrophysical MLT in Biermann (1932). We also note that some of the methods discussed below have also been used in the phenomenological models already introduced in Sect. 3.2 together with their model specific input.

#### Summary of methods

In dealing with turbulent flows a very ancient idea is that of a splitting of the basic fields such as velocity into an average and a fluctuation around it,

30 $$\begin{aligned} A_i = \overline{A_i} + A_i', \end{aligned}$$

where $A_i$ may be a scalar such as temperature *T* or the component of a vector field $\varvec{u}$. A key property of each of those averages is that $\overline{A_i'} = 0$. This *Reynolds decomposition* or *Reynolds splitting* was first suggested in Reynolds (1894) and allows the derivation of dynamical equations for a mean flow and fluctuations around the latter (see Chap. 4 in Pope 2000). In dealing with compressible flows it is of advantage to perform the Reynolds splitting for the conserved or at least density weighted variables, i.e.,

31 $$\begin{aligned} T = \widetilde{T} + T'' = \frac{\overline{\rho T}}{{\overline{\rho }}} + T'', \quad u_i = \widetilde{u_i} + u_i'' = \frac{\overline{\rho u_i}}{{\overline{\rho }}} + u_i'' \end{aligned}$$

as proposed by Favre (1969). This is known as *Favre averaging*. Variables which already relate to a quantity per volume such as $\rho$ (mass) or pressure *p* (internal energy) remain subject to the standard Reynolds decomposition, i.e., $\rho = {\overline{\rho }} + \rho '$ and $p = \overline{p} + p'$. In spite of that it has been used in astrophysical convection modelling only by a few authors, for instance, by Canuto (1997a). In analogy with the Reynolds average now $\widetilde{\rho T''} = 0$ as well as $\widetilde{\rho u_i''}=0$ whereas $\widetilde{T''} \ne 0$ as well as $\widetilde{u_i''} \ne 0$ (for exact relations and their derivation we refer to Canuto 1997a). These averages are hence *ensemble averages* of the variables appearing in (1)–(3) (see the discussions in Sects. 2.1.1 and 2.3.4 and for a more general introduction Pope 2000). As already discussed in Sect. 2.3.4 the construction of such averages may or may not involve also a spatially “horizontal average” when dealing with vertically stratified flows such as stellar convection whereas it always also assumes an average over initial conditions or time.

Specifically tailored to deal with turbulent convection is the *mass flux average*. There, quantities are averaged horizontally separately over areas of up- and downflow. In meteorology this has been used since the early work of Arakawa (1969) and Arakawa and Schubert (1974). A comprehensive review of this method is given in Mironov (2009). The mass flux average is used in meteorology as an alternative to the one-point closures based on Reynolds averaging for the purpose of deriving parametrized models for physical processes not resolved on the spatial grids affordable in numerical weather prediction models (see also Mironov 2009). Alternatively, it may be used to inspire closure approximations for the Reynolds stress approach discussed below (Mironov et al. 1999; Zilitinkevich et al. 1999; Gryanik and Hartmann 2002; Gryanik et al. 2005).

We note here that as is discussed in Chap. 5.4 of Tsinober (2009), it is essentially the decomposition of the basic variables combined with their mathematical representation which results in the appearance of a “cascade” (of transfer of energy, momentum, etc.). In particular, Tsinober (2009) points out that the appearance of a cascade of energy transport in Fourier space, crucial to many (in particular two-point closure) models of turbulent flows, is a *feature of the mathematical tool used to study the flow*. One should not mix this up with properties such as the physical scales of energy input and dissipation, as chosen by Kolmogorov when proposing his famous hypotheses (Kolmogorov 1941, cf. Chap. 6 in Pope 2000), which are *independent of the chosen decomposition*: the idea of

- statistical isotropy of motions at small scale (local isotropy hypothesis),
- the existence of a universal equilibrium range (first similarity hypothesis predicting an upper length scale below which the statistics of small scale motions depends on $\nu$ and $\epsilon$ only),
- an inertial subrange (second similarity hypothesis predicting a subrange within the former with a smallest length scale above which the statistics of motions depends only on $\epsilon$).

Equation (29) is a direct consequence thereof. This picture, intended to model flows of high Reynolds numbers $\mathrm{Re}$, was developed completely independent and ahead of its Fourier representation and is rather related to the idea of structure functions (cf. Chap. 6 in Pope 2000 as well as Hillebrandt and Kupka 2009 and Chap. 5.2 in Tsinober 2009). We note that $\mathrm{Re} = U(L) L / \nu \gg 1$ refers to velocities *U*(*L*) at length scales *L* which contain most of the kinetic energy of the flow. The strategy that this analysis could be conducted in Fourier space to provide further insight and mathematical means of modelling was suggested only thereafter by Obukhov (1941a, b) and Heisenberg (1948a, b). Finally, von Neumann (1963) realized that the *Richardson–Kolmogorov cascade picture*, used by Kolmogorov (1941) only in a footnote as a qualitative justification of his hypothesis of local isotropy at high $\mathrm{Re}$, is a process occurring in Fourier space (Chap. 5.4.2 in Tsinober 2009, see Panchev 1971 for an overview of such models). The term cascade is due to Onsager (1945, 1949), (see Chap. 5.4.1 in Tsinober 2009). In Sect. 2.1 of Kupka (2009b) it is discussed why observations of solar granulation or even standard LES thereof *cannot* reveal any such scaling other than by chance: basically, the achievable resolution is too small to identify “Kolmogorov scaling” in the data.

Once the averaging procedure has been defined, the basic Eqs. (1)–(3) are split into equations for the means and the fluctuations around them Pope (2000). By the non-linearity of (1)–(3) products of means and fluctuations of the basic variables appear such as $\overline{A_i A_j}$ and $A_i' A_j'$. One can construct dynamical equations for them by multiplying the dynamical equations for the fluctuations with the fluctuation of the same or other basic variables (i.e., $A_j' \partial _t A_i'$ etc.; dynamical equations for products of means are not needed). The required mathematical transformations are straightforward and involve only the product rule and basic algebraic operations (cf. Canuto 1992; Pope 2000). This procedure has first been proposed by Keller and Friedmann (1925). It is at this point where the *closure problem* comes in: any dynamical equation for a variable described as a product of *n* factors depends on variables which are the product of $n+1$ factors (i.e., $A_i' A_j'$ depends on $A_i' A_j' A_k'$). The *Friedman–Keller chain* thus consists of an infinite hierarchy of moment equations. For small Reynolds numbers a proof of the convergence of this hierarchy to a unique solution was given in Vishik and Fursikov (1988). Based on work by Fursikov in the early 1990s, the case of large Reynolds numbers was satisfactorily solved as well for a slightly idealized version of the full set (1)–(3) with the theorem of Fursikov and Emanuilov (1995). It states that for periodic boundary conditions and constant viscosities and diffusivities with suitably regular external forces and under the assumption that an exact solution exists for the original, dynamical equations (i.e., the NSE), the Friedman–Keller chain of approximations converges sufficiently fast (in an exponential sense) to a unique solution. While the exact rate of convergence cannot be determined by Fursikov and Emanuilov (1995), it assures the mathematical meaningfulness of the entire approach. In practice, the hierarchy is truncated at a certain order according to affordability. Additional assumptions, the *closure hypotheses*, have to be introduced to obtain a complete, predictive system of equations. Since for this reason the resulting mathematical model is not directly (ab initio) derived from the fundamental Eqs. (1)–(3), it has to be checked separately that the requirements formulated in Sect. 3.1 are fulfilled to ensure that the approximation obtained this way is mathematically and physically meaningful.

The following techniques are frequently used in deriving models of statistical properties of turbulent flows in general and turbulent convection in particular.

1. *One-point closure* models are popular in the context of the *Reynolds stress approach*. Also most phenomenological models such as MLT may be considered as one-point closure models in the sense that they consider averages of variables evaluated at certain locations at a particular point in space and in time, just as in Eq. (30). The Reynolds stress approach differs from those in deriving variables for higher order correlations including $\overline{u_i' u_j'}$, too, a quantity neglected in MLT but of major physical importance as it is directly related to the basic, non-linear advection represented by the term $\mathrm{div}( \rho (\varvec{u} \otimes \varvec{u}))$ in Eq. (2). Most non-local models of convection used in or proposed for stellar astrophysics are of this type as well.
2. In contrast, *two-point closure* models are the main tool in studying (statistically) isotropic or homogeneous turbulence. For an extended discussion of available methods see Lesieur (1997). The main idea there is to consider correlations of functions evaluated at *different points in space*, such as velocity differences which appear in the hypotheses of Kolmogorov. Already since the work of Heisenberg (1948a, b) and other contemporary authors, it has become common to transform the exact dynamical equations for such correlations into Fourier space and construct models for them in the latter and eventually use those to predict one-point correlations such as the convective flux. Representatives of this approach, which are widely used in stellar astrophysics, are the models of Canuto and Mazzitelli (1991, 1992) and Canuto et al. (1996). These are *local* models of convection and for reasons of mathematical complexity this approach is hardly used for *non-local* ones other than for deriving closure hypotheses for one-point closure models.
3. *Diagram techniques* are a method sometimes used to compute quantities in the context of two-point closure models by expansion and summation over all (infinite) contributions similar to quantum field theory. In turbulence theory the *renormalization group approach* underlying these techniques has to face the difficulty of so-called *infrared divergences*, i.e., contrary to quantum electrodynamics (QED) the boundary conditions at a finite region in space matter and hence one has to deal with functions that take over the role of a simple, constant scalar such as the charge of the electron in QED. A detailed introduction into this approach is given in McComb (1990). Its best known application in convection modelling in stellar astrophysics is the model of Canuto and Dubovikov (1998), where it has been used to compute some of the time scales that appear in the one-point closure Reynolds stress models derived in that paper.

#### Non-local models of turbulent convection

The model of Kuhfuß (1986) is quite different from its non-local predecessors proposed in Unno (1967), in Gough (1977a, b), and in Stellingwerf (1982) in the sense that it does not rely on dynamical equations describing the behaviour of convective eddies or bubbles from the outset and it does not just use the diffusion approximation to merely model overshooting. Rather, it starts straight from the full hydrodynamical equations and applies the anelastic approximation (see Sect. 4.3.2) and the diffusion approximation (for non-local transport processes) in a consistent manner. Only a simplified version of the model is used in practice which requires to solve a differential equation for the (turbulent) kinetic energy in addition to other dynamical equations required by stellar structure and stellar pulsation modelling. It is used to account for overshooting and for the time dependence of convection in radially pulsating stars. In this sense it competes directly with the earlier models published in Gough (1977a, b) and in Stellingwerf (1982). However, as already pointed out in Canuto (1993), the diffusion approximation for the flux of kinetic energy is highly incomplete. This can be seen from comparisons with 3D direct numerical simulations of fully compressible convection as discussed in Kupka and Muthsam (2007b) and in Kupka (2007). They demonstrated that the downgradient drastically underestimates the flux of kinetic energy and third order moment of vertical velocity in the interior of convection zones with different efficiencies of radiative transfer, even if the free parameter of the approximation is tweaked to fit their profiles in the overshooting zone (see also Chan and Sofia 1996). The model is even less consistent for (third order) cross correlations of velocity and temperature fluctuations (see also Sect. 3.3.3). From this point of view the model described in Kuhfuß (1986) can thus at best only account in a rather rudimentary way for the non-locality of turbulent convection. Similar holds for its model of the convective flux when probed with 2D LES of a Cepheid Mundprecht et al. (2015). In spite of the stability and relative simplicity of this class of models it thus appears desirable to consider more general models of turbulent convection.

#### Non-local Reynolds stress models

When proceeding towards physically more complete models of convection the issue of realizability becomes more and more important, since both mathematically and physically the models increase in complexity and the interplay between the different approximations may have unwanted side effects leading to numerical instability or even unphysical solutions. This is probably why the so-called downgradient approximation (DGA) has remained so popular in non-local models of stellar convection. It assumes that there is a gradient in one or several of the averages of the products of the fluctuating (turbulent) quantities, for instance the second order moment $\overline{{u'_3}{u'_3}}$, which gives rise to a flux that has the form of a third order moment, for example, $\overline{{u'_3}{u'_3}{u'_3}}$ or even the flux of kinetic energy $F_\mathrm{kin}$ (see below). This flux is assumed to be proportional (and opposite) to the gradient just introduced times a *diffusivity* which involves the quantity being transported by the flux. Consistently applied to all correlations of third order for the velocity and temperature fields this is a generalization of the model of Kuhfuß (1986) discussed in Sect. 3.3.2. That in turn is a generalization of the idea of *turbulent diffusion* introduced in Sect. 3.2.1 where the driving gradient is obtained from the mean of the basic variable ($\overline{T}$, etc.). For processes occurring on small scales such as radiative and heat diffusion, Eq. (4) and Eq. (6), this allows a very accurate description of transport (of energy, momentum, etc.). However, for turbulent transport there is no reason why the quantity transported should not vary strongly along typical scales along which the transport occurs (a “mean free path” in the flow). The Taylor expansion underlying the diffusion approximation hence cannot be expected to hold and in this sense the downgradient approximation for turbulent transport is a model with a limited region of applicability. This appears to be the case also in comparisons to numerical simulations of compressible convection to which we return below (cf. Kupka and Muthsam 2007a, b, c; Kupka 2007). Apparently, the DGA of third order moments is more likely to hold at the boundary of convective zones and in regions of overshooting (see also Chan and Sofia 1996). In spite of these shortcomings, it has been frequently used also for non-local Reynolds stress models to which we turn in the following.

Xiong has been the first to promote the use of the Reynolds stress approach to derive models of stellar convection (see Xiong 1978). If we recall the notation and discussion from Sect. 3.3.1 and consider the plain Reynolds average (30) such that $w' = w - \overline{w}$ is the fluctuating part for the vertical velocity, $\theta ' = T - \overline{T}$ is its counterpart with respect to temperature, and $q^2 = {u'_1}^2+{u'_2}^2+{u'_3}^2 = 2\,K$ is the turbulent kinetic energy resulting from the sum of both vertical and horizontal components of velocity fluctuations, a Reynolds stress model aims at first deriving dynamical equations for these quantities directly from the Navier–Stokes equations (possibly within the Boussinesq approximation, but not necessarily so, see Canuto 1993, 1997a). The models also consider the cross-correlation $\overline{{u'_i}{u'_j}}$ which is known as the *Reynolds stress*. Additional hypotheses have to be assumed to obtain a *closed* system of differential equations, hence their name *closure hypotheses* or *closure assumptions*. Such hypotheses cannot be expected to hold for all physical scenarios and even for the same type of flow (such as compressible convection) it may be difficult to find one which is not very sensitive to the physical parameters of the system. To express

1. asymmetry between up- and downflows,
2. non-locality of the generation of enthalpy and kinetic energy fluxes,
3. and non-local processes related to the generation of buoyancy

a certain minimum complexity of the model appears inevitable and in this sense the Reynolds stress models are more complicated and physically more complete than the non-local convection models discussed so far. A Reynolds stress model thus provides dynamical equations at least for

32 $$\begin{aligned} \overline{q^2}, \quad \overline{{\theta '}^2}, \quad \overline{w'\theta '}, \quad \overline{{w'}^2}. \end{aligned}$$

If a gradient in mean molecular weight (and thus concentration, say of helium, e.g.) is to be accounted for, additional correlations of *second order* appear similar to those just given in Eq. (32). It is these quantities that are modelled by the approach of Xiong (1978, 1985) and Xiong et al. (1997). The quantities appearing in (32) are closely related to the already known ones from convection modelling in general and as frequently computed from hydrodynamical simulations: turbulent pressure $p_\mathrm{turb}=\overline{\rho {w'}^2} \approx {\overline{\rho }}\,\overline{{w'}^2}$, convective (enthalpy) flux $F_\mathrm{conv} = \overline{\rho h' w'} \approx c_\mathrm{p}\,{\overline{\rho }}\,\overline{w'\theta '}$ (where enthalpy fluctuations $h'$ have been approximated), flux of (turbulent) kinetic energy $F_\mathrm{kin} = \frac{1}{2}\overline{\rho \,q^2\,w'} \approx \frac{1}{2}{\overline{\rho }} \overline{q^2 w'}$, and potential energy contained in the fluctuations of temperature (or alternative variables such as enthalpy or entropy), related hence to $\overline{{\theta '}^2}$. Approximations are made for correlations of third (or even fourth) order which are expressed in terms of second order correlations, hence the name *second order closure* (SOC). Indeed, one of the just mentioned quantities, $F_\mathrm{kin}$, actually stems from a third order correlation and in Xiong (1978, 1985) it is approximated by the downgradient approximation (DGA) like in the non-local model of Kuhfuß (1986). Since the DGA introduces serious restrictions, as already mentioned above, the model is certainly only a rather incomplete description of convective overshooting from the unstable convection into neighbouring, “stable” layers. The model of Xiong (1978, 1985) has been applied to a number of problems from stellar astrophysics: overshooting in massive stars, Xiong (1986), and in the shallow convection zone of late B to early F type stars, Xiong (1990), were among the earliest ones.

A more complete model (Xiong et al. 1997) was published following the work of Canuto (1992, 1993) where it had been proposed to consider the full dynamical equations for third order moments, close them at fourth order through the (eddy damped) quasi-normal approximation, assume them to be stationary and thus obtain algebraic equations for the third order moments which allow a second order closure. The detailed procedures of Canuto (1992, 1993) and Xiong et al. (1997) are, however, different, so it is not advisable to conclude from results for one of the models on the other. The model of Xiong et al. (1997) was applied to compute overshooting below the solar convection zone (Xiong and Deng 2001), and to compute pulsational stability (Xiong et al. 2015), although mixed results have been reported on the latter (Houdek and Dupret 2015).

The models of Xiong (1978, 1985) and Xiong et al. (1997) still contain a mixing length, which is used to compute the dissipation rate of turbulent kinetic energy, $\epsilon$, according to Eq. (29). Canuto (1992) first proposed to abandon this procedure and suggested to instead consider its computation from a dynamical equation, which models the exact (and complicated to close) equation for this quantity, as done in the engineering community for already a long time at that point in the more basic $K-\epsilon$ model of shear driven turbulence. Hence its designation as a *fully non-local* model of turbulent convection, since the model avoids the use of a mixing length also for the computation of $\epsilon$. In Canuto (1993) the originally used Boussinesq approximation was eased by accounting for pressure fluctuations through a linear expansion.^5^ In Canuto and Dubovikov (1998) a turbulence model based on the diagram technique mentioned in Sect. 3.3.1 was used to compute the time scales that appear in the Reynolds stress approach of Canuto (1992, 1993) and which are related to the dissipation of temperature fluctuations and to cross-correlations between (fluctuations of) the pressure gradient and velocity as well as temperature fluctuations. In this improved form the fully non-local Reynolds stress model was first solved^6^ for the case of compressible convection in Kupka (1999). Direct numerical simulations appropriate to study overshooting in an idealized setting which is fairly similar to that one found in A-type stars and hot DA white dwarfs (apart from a much lower Reynolds number and much higher Prandtl number assumed for the 3D calculations) have been evaluated and compared to the fully non-local Reynolds stress model. It was found that not all terms in the model of third order moments of Canuto (1993) could be kept as this would prohibit converging solutions. In this form the model delivered promising results which was considered an indication that it should work at least for shallow convection zones with strong radiative losses (low Peclet number and in this sense inefficient convection). This deficiency may also be at the root of the problems of the approach taken by Xiong et al. (1997, 2015) and Xiong and Deng (2001) and discussed in Houdek and Dupret (2015). It was corrected in a new model for the third order moments in Canuto et al. (2001). In this form, now based on the most complete model as proposed by Canuto (1992, 1993), Canuto and Dubovikov (1998) and Canuto et al. (2001), the model was used to study the convection zone in A-type stars as a function of effective temperature in Kupka and Montgomery (2002). A reasonable qualitative and even rough quantitative agreement was found when comparing those results to 2D LES as discussed in Freytag et al. (1996) for the standard choice of parameters of the Reynolds stress model. MLT by comparison requires lowering $\alpha$ from values of 1.6–2, as used in solar modelling, down to a value of 0.36 just to match the maximum of the convective flux. It also cannot account for the huge amount of overshooting in terms of pressure scale heights found from both the Reynolds stress model and the 2D LES. In Marik and Petrovay (2002) the model of Canuto and Dubovikov (1998) without compressibility corrections and assuming the downgradient approximation and additionally a fixed ratio between $\overline{q^2}$ and $\overline{{w'}^2}$ (i.e., a fixed degree of anisotropy of the velocity field) was solved for the layers at the bottom of the solar convection zone. It was found to lead to a small amount of overshooting in agreement with helioseismic measurements as opposed to a simple downgradient model which is used in one-equation non-local models of convection and which predicts a much larger overshooting. In its full form the model—with compressibility corrections, without fixed anisotropy, and avoiding the downgradient approximation—was then applied to the case of shallow surface convection zones in hot DA white dwarfs in Montgomery and Kupka (2004). They considered slightly higher effective temperatures compared to that one for the numerical simulation shown in Fig. 4 in this review. Remarkably, despite MLT requires a much larger parameter $\alpha$ for this case, the fully non-local Reynolds stress model again agrees reasonably well in a qualitative sense and roughly quantitatively with 2D LES from the literature (e.g., Freytag et al. 1996).

This, however, already denotes the region of applicability of this model in its original form. A detailed comparison between shallow convection with high radiative losses (which always occur also at the boundary of a convection zone) and a case of deep convection zones with overshooting, again for the case of idealized microphysics and thus fully resolved on all length scales (i.e., a direct numerical simulation), using the same 3D hydrodynamical code and set up presented in Muthsam et al. (1995, 1999) as used in Kupka (1999), revealed deficiencies of the used closure model (Kupka and Muthsam 2007a, b, c; Kupka 2007). Indeed, the closures for the cross-correlations $\overline{{w'}^2 \theta '}$ and $\overline{w' {\theta '}^2}$ as well as for $\overline{{w'}^3}$ were found unsatisfactory (Kupka and Muthsam 2007b; Kupka 2007). The downgradient approximation of these quantities performs even less satisfactorily.

**Fig. 8:**
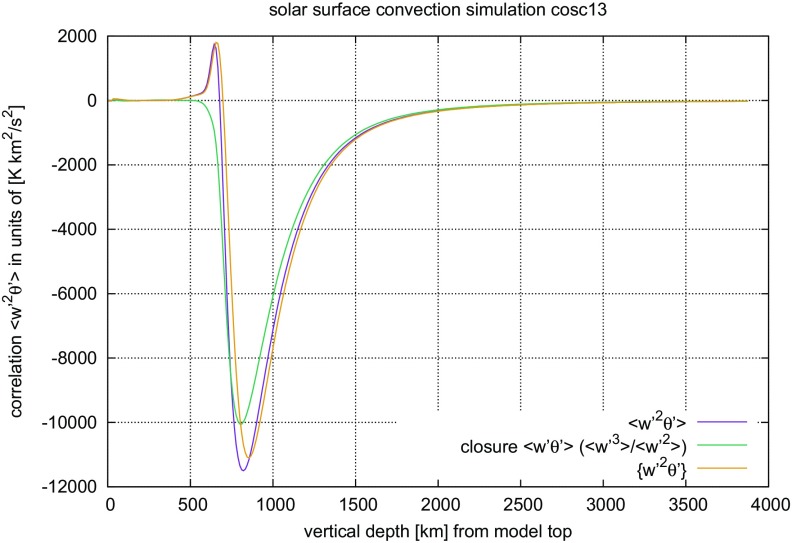
Correlation $\overline{w'^2\theta '}$ at the solar surface computed from a numerical simulation with ANTARES (Muthsam et al. 2010) (details on the simulation: Belkacem et al. 2015, in prep.) in comparison with a closure relation and the Favre average $\{w'^2\theta '\}=\overline{\rho w'^2\theta '}/{\overline{\rho }}$. For details see text

**Fig. 9:**
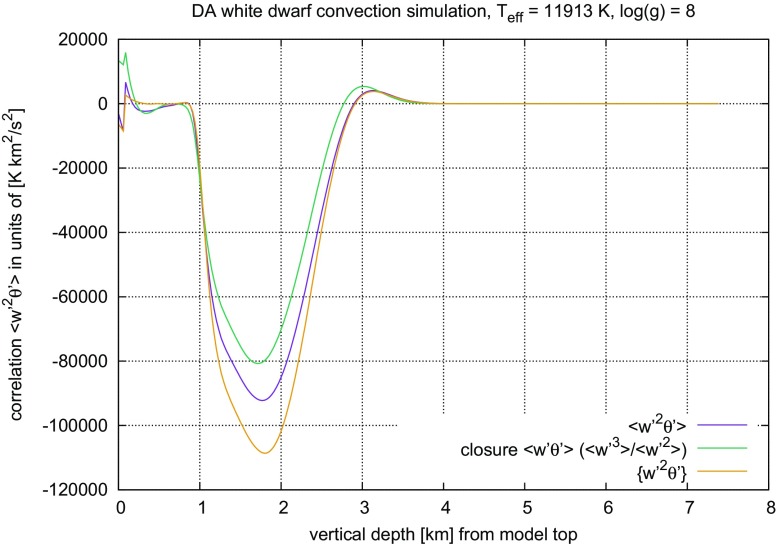
Correlation $\overline{w'^2\theta '}$ at the surface of a DA white dwarf computed from a numerical simulation with ANTARES (Muthsam et al. 2010) (details on the simulation: Kupka et al. 2017, submitted) in comparison with a closure relation and the Favre average $\{w'^2\theta '\}=\overline{\rho w'^2\theta '}/{\overline{\rho }}$. For details see text

Thus, the closures used in these models require improvements to extend the region of applicability of the whole approach to proceed beyond what is possible with the more commonly used one-equation non-local models of convection. One such possible alternative has been suggested in the meteorological community and is based on a two-scale mass flux approach, where up- and downflows are not strictly coupled to regions of hot and cold flow with respect to their horizontal average (see Zilitinkevich et al. 1999; Mironov et al. 1999; Gryanik and Hartmann 2002; Gryanik et al. 2005). This approach provides closures for the combinations of $w'$ and $\theta '$ if the skewness of both is known (so this is not just a second order closure since it requires to know two third order quantities, $S_w$ and $S_{\theta }$). One example is the relation

33 $$\begin{aligned} \overline{w'^2\theta '} \approx \overline{w'\theta '} (\overline{w'^3}/\overline{w'^2}), \end{aligned}$$

which has also been proposed in Canuto and Dubovikov (1998) in an alternative model for closing their Reynolds stress equations (this particular closure can already be derived from the standard mass flux approach as in Arakawa (1969) and Arakawa and Schubert (1974), since it only depends on $S_w$). Figures 8 and 9 demonstrate the results for the case of the 3D LES of the Sun and a white dwarf, as introduced already in Sect. 2. A remarkable agreement is found, very similar to what had been found in Kupka and Robinson (2007) for a different set of 3D LES (with closed vertical boundary conditions) for the Sun and a K-dwarf. This has likewise been found in Kupka and Muthsam (2007b) and Kupka (2007) for direct numerical simulations of compressible convection for both the case of a shallow zone and a deep zone with efficient convective transport. However, as also pointed out in Kupka (2007), just putting the best closures around together does not mean that the resulting Reynolds stress model is an improvement. The assumptions underlying the closures have to be compatible otherwise the resulting model might even be unstable. We note here that another closure of this type is shown in Figs. 15 and 16 in Sect. 6.2. In spite of this limitation when using them in self-consistent, stand-alone models, these closures have already been used in an improved model of p-mode excitation of solar-like oscillations (Belkacem et al. 2006a, b; Samadi et al. 2008). There, the model also takes input from 3D LES.

We also note here that the Favre average of the cross correlation deviates quite a bit less from the plain Reynolds average than one might have intuitively expected (see Figs. 8, 9 here and also Figs. 15, 16 from Sect. 6.2). The deviations hardly exceed 20% even within the superadiabatic layer, where the fluctuations of pressure, temperature, and density are largest. This might be interpreted as an indication that the complex account for full compressibility, as proposed in the model in Canuto (1997a), might not be necessary at this stage of modelling, since it is a “higher order effect” in comparison with getting just the Reynolds averaged closures right.

The overview on the use of the Reynolds stress approach for modelling stellar convection given here is not exhaustive, since our focus has been to demonstrate how physically complete these models have become. Recent efforts as presented in Canuto (2011) have concentrated on providing a framework for an easier implementation of the approach: the idea is to be able to increase the physical completeness of the model step-by-step which in practice is easier than starting from the most complete model and simplifying it in turn. Likewise, as an alternative to the full model of Xiong (1985) and Xiong et al. (1997), a $K{-}\omega$ model was proposed in Li (2012), where an equation for the inverse time scale associated with dissipation rate of kinetic energy, $\omega \equiv \tau ^{-1} \equiv \epsilon /\overline{q^2}$, replaces that one for $\epsilon$. This is actually a two-equation model as is standard in modelling of flows in engineering applications and is thus simpler than a full Reynolds stress model. In this respect it belongs to the non-local models discussed in Sect. 3.3.2. Its local limit was used in stellar evolution calculations and compared to the mixing obtained with the full $K-\omega$ (see Li 2012).

#### Rotating convection and two remarks

We note that the above discussion of the Reynolds stress approach is far from complete. A very natural extension is the case of convection in a rotating environment. Indeed, astrophysical objects, whether stars or planets, all rotate, and in some cases even very rapidly. The interaction of convection with rotation is known to lead to the phenomenon of differential rotation which cannot just be studied with numerical simulations such as those presented by Brun and Toomre (2002) and many others (we refer again to Miesch 2005 for a review), but also by extensions of the types of convection models we have presented here. Examples and an overview on this field of modelling can be found in Rüdiger (1989). As is necessary by the very nature of rotation many of the analytical or semi-analytical models are no longer “1D models” in the traditional sense (a more recent example is the model of Rempel 2005 which has also co-inspired a new numerical technique to which we return in Sect. 4.5). Indeed, the most complete among the Reynolds stress models available at the moment, published in Canuto (1999), also accounts for differential rotation (in addition to convection, double-diffusive convection, diffusion, and overshooting). In its full form it provides actually a 3D model of a star, although the formalism suggests a route to gradually simplify this model (this is again addressed, in a more systematic way, in Canuto 2011 and the series of papers introduced therein). From a physical point of view we note that these models contain no fundamentally new techniques other than those already introduced: one-point ensemble averages, possibly Favre averaging, closure hypotheses, renormalization group techniques and two-point closures to compute specific quantities, possibly results from and calibrations with numerical simulations, etc. This holds despite for each model some specific approximations are suggested to deal with the problems at hands.

As a remark we note that the most adequate modelling strategy for convection is constrained by the specific astrophysical questions which are studied. For example, our understanding of solar and stellar activity requires modelling of surface and interior convection zones which *does* take into account the interaction of convection with magnetic fields (see Miesch 2005 and further literature cited therein). Likewise, numerical simulations and a proper accounting of effects due to deviations from local thermal equilibrium have to be considered in high precision determinations of solar (and stellar) element abundances (Nordlund et al. 2009). In each of these cases a convection model which reduces the physical description to values of horizontal averages cannot be expected to yield an acceptably accurate description. On the other hand, for other problems such as stellar evolution over very long time scales, there is no alternative to such strategies, at least not within the foreseeable future, as we summarized in Sect. 2.6.

We conclude this part with a second, optimistic remark: although presently the non-local convection models still have a lot of weaknesses even if considered in their physically most complete form, they have the potential to substantially improve the modelling of stellar convection over the classical, local MLT approach. This is well needed, since multidimensional, hydrodynamical simulations are just not applicable to all problems in which stellar convection matters (see also Sect. 2).

## Multidimensional modelling: the equations

As has been discussed in Sect. 2 one basically knows the equations which one wishes to solve for stellar convection: these are the Navier–Stokes (or Euler) equations of hydrodynamics, properly augmented with various ingredients according to the precise physical problem at hand.

It is not always prudent or possible to numerically solve these equations as they are when dealing with stellar convection problems. Often, in particular near the stellar photosphere, convective velocities are large (roughly of the order of the speed of sound), such as in solar granulation. In the interior of many stars, however, convective motions are very slow in terms of sound speed. In those cases, the usual numerical methods for time integration of the Navier–Stokes or Euler equations would take a prohibitively long time for execution, the reason being that, without special measures, one has to track the sound waves. This results in a very small time step, completely in disagreement with the much larger time scale on which the solution itself changes: a computation of that sort would be stalling. Methods are therefore requested to explicitly circumvent this difficulty. Such methods come in two basic flavours. One way is to modify the Navier–Stokes or Euler equations themselves in such a manner that the sound waves are eliminated. Such methods are described in this chapter, Sects. 4.3–4.6. The alternative leaves the Navier–Stokes or Euler equations unchanged but makes use of numerical methods enabling time steps compliant with the actual changes in the solution. Such approaches are described later on, see Sect. 5.3.

### Conservation laws

The equations of hydrodynamics, which are at the core of our considerations, express the conservation (balance) of mass, momentum and energy. To understand the numerical methods used for their solution a short description of properties of conservation laws is therefore in order.

#### The 1D case

Consider a function $v=v(x,t)$, defined along some *x*-interval and for all times *t*. Typically in applications to follow, *v* will be a *density* (mass-, momentum- or energy-density). We assume that there exists a flux function *f*(*v*), so that $\int _{x_0}^{x_1}v\,dx$ changes by a flux through the boundaries $x_0$ and $x_1$ according to a flux function *f* in such a way that for all times *t*, for all time increments $\tau$ and for all points $x_0, x_1$ we have

34 $$\begin{aligned} \int _{x_0}^{x_1}v(x,t+\tau )\,dx= & {} \int _{x_0}^{x_1}v(x,t)\,dx\nonumber \\&-\left( \int _{t}^{t+\tau }f(v(x_1,t))\,dt-\int _{t}^{t+\tau }f(v(x_0,t))\,dt\right) .\nonumber \\ \end{aligned}$$

In this way, *f*(*v*(*x*, *t*)) actually describes the flux of quantity *v* through position *x* at time *t*. Subtracting the first term on the right hand side, multiplying by $\frac{1}{\tau }$ and letting $\tau$ tend to zero, we obtain

35 $$\begin{aligned} {\partial _t}\left( \int _{x_0}^{x_1}v(x,t)\,dx \right) =-(f(v(x_1,t))-f(v(x_0,t))) \quad \forall x_0, x_1. \end{aligned}$$

Using this equation, proceeding similarly in the variable *x* ($x_1\rightarrow x_0$) and finally writing *x* instead of $x_0$, we obtain $\partial _t v(x,t)=-\partial _xf(v(x,t))$, which we typically will use in the form

36 $$\begin{aligned} \partial _t v(x,t)+\partial _xf(v(x,t))=0. \end{aligned}$$

Conversely, integration of Eq. (36) leads back to Eq. (35). These equations therefore express conservation, and equations of the form Eq. (36) are called *conservation laws* (in the case of one spatial dimension). The transition from the original integral form Eq. (34) holds true when the main theorem of calculus is applicable, i.e., when *v* and *f* are continuously differentiable. It should be noted that this condition is not always fulfilled in stellar convection (in particular in granulation) and that situations (shocks) may occur in which the solution is discontinuous. In these cases, Eq. (35) which directly expresses the physical conservation principle has to be considered the basic one.

#### The 3D case

In the case of several spatial dimensions similar considerations apply. We now have, for the 3D case, $\varvec{x}=(x,y,z)^*$ or $\varvec{x}=(x_1,x_2,x_3)^*$ where the asterisk denotes the transpose. We again consider a scalar function $v(\varvec{x},t)$ which describes a conserved quantity, which means that there is a vector valued *flux function* $\varvec{f}(\varvec{v},t)$, such that, for each bounded domain $\varSigma$ in space, we have

37 $$\begin{aligned} {\partial _t}\left( \int _\varSigma v(\varvec{x},t)\,d\varvec{x}\right) + \int _{\partial \varSigma }\langle {\varvec{f}(v(\varvec{x},t)),\varvec{n}}\rangle \,d\sigma = 0. \end{aligned}$$

Here, $\partial \varSigma$ denotes the boundary of $\varSigma$ and $\varvec{n}$ the outward pointing normal and $d\sigma$ the surface element. In three dimensions, we will use the notation $\varvec{f}=(f,g,h)$ or $\varvec{f}=(f_1,f_2,f_3)$ (no asterisk this time). $f_i$ is the flux in direction $x_i$.

Using Gauss’ theorem, we can write the boundary integral in Eq. (37) as a volume integral and obtain

38 $$\begin{aligned} {\partial _t}\int _\varSigma v(\varvec{x},t)\,d\varvec{x}+ \int _{\varSigma }{{\mathrm{div}}}{\varvec{f}(v(\varvec{x},t)})\,d\varvec{x}= 0. \end{aligned}$$

Taking the time-derivative inside the integral, we have then that

$$\begin{aligned} \int _\varSigma \left( {\partial _t}v(\varvec{x},t)\,d\varvec{x}+ {{\mathrm{div}}}{\varvec{f}(v(\varvec{x},t))}\,d\varvec{x}\right) = 0 \quad \forall \varSigma . \end{aligned}$$

If the integrand is continuous we can conclude that

39 $$\begin{aligned}&\displaystyle {\partial _t}v(\varvec{x},t) + {{\mathrm{div}}}{\varvec{f}(v(\varvec{x},t))} = 0, \hbox { or}\end{aligned}$$

40 $$\begin{aligned}&\displaystyle \partial _t v(x,t) + \partial _xf(v(x,t))+\partial _yg(v(y,t))+\partial _zh(v(z,t)) = 0. \end{aligned}$$

In hydrodynamics in 3D space, we typically deal with five densities of conserved quantities: mass density $$\rho $$, momentum density $$\mu _j$$ in direction *j* ($$j=1,2,3)$$ and some energy density *e*. We are therefore led to consider a vector valued function $$\varvec{v}=(\rho ,\mu _1,\mu _2,\mu _3,e)^*$$ in the hydrodynamic case. For each component, a conservation law applies and there is, for the *i*th component $$v_i$$, a flux function $$\varvec{f}_i(\varvec{v},t)=(f_{ij})_{j=1,2,3}$$. We assemble these flux functions into a matrix valued flux function $$\varvec{f}=(f_{ij})$$, so that the *conservation law in differential form* reads

41 $$\begin{aligned} \partial _tv_i(\varvec{x},t)+\sum _{j=1}^3\partial _{x_j}f_{ij}(\varvec{v}(x,t)) = 0. \end{aligned}$$

For validity of the *differential* form of the conservation laws the considerations above require that smoothness properties are fulfilled (interchange of differentiation and integration and, even more basically, differentiability of the functions). Otherwise, the differential form may not hold true of even be meaningful. This has profound implications both for the physics and for the numerical treatment of astrophysical flows.

### Compressible flow: the Euler and Navier–Stokes equations

#### The basic equations

We recall from Sect. 2 that the equations properly describing purely hydrodynamic stellar convection are the Navier–Stokes equations augmented by equations describing the actions of microphysics, radiation transfer, and gravity. Viscosity acts on very small scales only and cannot be directly resolved in numerical calculations as follows from the discussion in Sect. 2.2.1. Hence, the inviscid subset (plus a radiative heating term and gravity) plays a dominant role, i.e., the augmented Euler equations. We return to the non-trivial implications of this transition in Sect. 4.2.3.

We recall Eqs. (1)–(10) from Sect. 2.1 and for the remainder of Sects. 4 and 5 we set $q_\mathrm{nuc} = 0$ and $\varvec{h}=0$. The latter is mathematically indistinguishable from radiative transfer in the diffusion approximation and $q_\mathrm{nuc} \ne 0$ leads to source terms specific to convection in stellar cores or nuclear burning shells only. Using the symbols of Table 1, both Euler and Navier–Stokes equations can be written as follows:

42a $$\begin{aligned}&\displaystyle \partial _t\rho +{{\mathrm{div}}}{}\rho \varvec{u}= 0, \end{aligned}$$

42b $$\begin{aligned}&\displaystyle \partial _t \varvec{\mu }+ {{\mathrm{div}}}{(\rho \varvec{u}\otimes \varvec{u}+ p\varvec{I})} {- {{\mathrm{div}}}\varvec{\pi } } = \rho \varvec{g},\end{aligned}$$

42c $$\begin{aligned}&\displaystyle \partial _t e + {{\mathrm{div}}}{(e+p)\varvec{u}} {-{{\mathrm{div}}}\varvec{u}^*\varvec{\pi } } + q_\mathrm{rad} = \rho \varvec{u}^*\cdot \varvec{g}{,} \end{aligned}$$

where the Euler case is distinguished by $\varvec{\pi } = 0$ and we use the more stringent notation for the transpose introduced above. The basic fluxes for the Euler equations, i.e., for $\varvec{\pi } = 0$ and also neglecting $q_\mathrm{rad}$, are provided in Table 3 and have rather obvious physical interpretations.

For the entire discussion provided in this and the following section we assume a prescribed acceleration *g* or $g(x_1)$ due to gravity pointing in direction $x_1$. We only consider scenarios for which a self-consistent determination of *g* is not necessary and thus $g = \mathrm{const.}$ or $g= -GM r^{-2}$ for problems with deep convection and spherical symmetry, i.e., for the non-rotating case.

The *Lagrangian* or *substantial* derivative is often useful in interpreting hydrodynamic equations. Consider a physical quantity (density $\rho$ for example). The Lagrangian derivative, denoted by $D_t$, describes the rate of change of the quantity when moving with the fluid. In 1D, the vector $\mathbf {r}=(u,1)$ describes the vector of the trajectory of a fluid moving with velocity *u* in the (*x*, *t*)-plane in one unit of time. Hence, for some function $\phi , D_t\phi$ is the directional derivative $D_t\phi =\partial _{\mathbf {r}}\phi =u\partial _x\phi +1\partial _t\phi$. In multidimensions, we similarly have

43 $$\begin{aligned} D_t\phi =\partial _t\phi + \sum u_i\partial _{x_i}\phi . \end{aligned}$$

**Table 3:** Advective (hyperbolic) fluxes for the 3D Euler (and Navier–Stokes) equations

Density	$f_\rho = (\mu _1,\mu _2,\mu _3) = (\rho u_1,\rho u_2,\rho u_3 )$
*x*-Momentum	$f_{\mu _1}=(\mu _1 u_1+p,\mu _1 u_2,\mu _1 u_3)$
*y*-Momentum	$f_{\mu _2}=(\mu _2 u_1,\mu _2 u_2+p,\mu _2 u_3)$
*z*-Momentum	$f_{\mu _3}=(\mu _3 u_1,\mu _3 u_2,\mu _2 u_3+p)$
Energy	$f_e=(e+p)(u_1,u_2,u_3)$

#### Radiative heating

We recall that the radiative heating term ${q}_\mathrm{rad}$ is derived from the radiative energy flux, $\varvec{f}_\mathrm{rad}$ (integrated here over all frequencies). In order to determine this flux one has, in principle, to solve the equations of radiative transfer (for all frequencies, in practice for some sample of frequencies). Combining these with the hydrodynamic equations one arrives at the equations of radiation hydrodynamics (see Castor 2007; Mihalas and Mihalas 1984).

In studying convection near the stellar surface (granulation) it is necessary to solve at least the stationary equation of radiative transfer (or some really good approximation to it). Due to the non-locality of radiation the numerical treatment differs in fundamental ways from the numerics of the hydrodynamic equations. Anyway, once the radiation field is determined (in general in several spectral bands), the radiative heating rate $q_\mathrm{rad}$ can be determined from the radiative flux $\varvec{f}_\mathrm{rad}$ via

44 $$\begin{aligned} q_\mathrm{rad}={{\mathrm{div}}}{\varvec{f}_\mathrm{rad}}. \end{aligned}$$

Since in the following we do not treat stellar atmospheres we need not deal with the numerics of the radiative transfer equation in itself. Rather, we make use of the fact that, for large optical depth, the solution of the transfer equation converges to the solution of the numerically simpler *diffusion approximation*,

45 $$\begin{aligned} \varvec{f}_\mathrm{{rad}}=-K_\mathrm{{rad}}{{\mathrm{grad}}}{T}, \end{aligned}$$

introduced in Sect. 2.1. Contrary to the full radiative transfer equation the gradient of *T* can be computed locally, in accordance with all the other terms in the hydrodynamic equations. The radiative (Rosseland) conductivity $K_\mathrm{{rad}}$ contains a weighted harmonic mean of monochromatic opacities (mean over all frequencies) and can easily be interpolated from tables, in particular those due to the Opacity Project (cf. Seaton et al. 1994; Seaton 2005). Mathematically, the ensuing term ${{\mathrm{div}}}{q_\mathrm{{rad}}=-{{\mathrm{div}}}(K_\mathrm{{rad}}{{\mathrm{grad}}}{T})}$ and ${{\mathrm{div}}}{q_\mathrm{{rad}}}$ reduces to $-K_\mathrm{{rad}}\varDelta {T}$ in the case of constant $K_\mathrm{{rad}}$.

#### Viscosity

In Eq. (8) we have written the tensor viscosity $\varvec{\pi }$ in its most general form, i.e., for the case of a non-zero bulk viscosity, also known as “second viscosity” or expansion viscosity, $\zeta$ as it is derived in Landau and Lifshitz (1963) or Batchelor (2000). For a mono-atomic gas with no internal degrees of freedom, $\zeta =0$, while in most fluids for which their constituents (atoms, molecules, etc.) have internal degrees of freedom, $\zeta \approx \eta$, see Chap. 3.4 in Batchelor (2000). A detailed discussion of a formalism to compute $\zeta$, if the atoms or molecules of the fluid are subject to a slow relaxation back into thermal equilibrium after having been driven away from it by compression or expansion, is given in Landau and Lifshitz (1963) and in this case $\zeta$ may become large. In astrophysics, it has always been assumed that $\zeta \approx 0$ or at most $\zeta \approx \eta$ and can thus be neglected for reasons described below, until Cowley (1990) identified the exchange between translational energy of electrons and their internal binding energy in hydrogen atoms as a possible candidate for the aforementioned slow relaxation process. This could make $\zeta$ large enough to be of relevance for the solar photosphere (see also Sect. 2.1 in Kupka 2009b). Apparently, this result has not been verified by other authors and no applications of it seem to have been published. For the remainder of this text we hence assume $\zeta$ to be small and neglect it in the following.

The components of the viscosity tensor $\varvec{\pi }$ in the momentum equation (Eq. 42b) and the energy equation (Eq. 42c) in this case reduce to

46 $$\begin{aligned} \pi _{ik}= \eta \left( (\partial _{x_{k}}u_i+\partial _{x_{i}}u_k)-\frac{2}{3}{{\mathrm{div}}}{\varvec{u}}\right) . \end{aligned}$$

As we have discussed in Sect. 2.2.1 the viscosity coefficient and, hence, the corresponding length scale on which viscosity effectively smoothes the solution is orders of magnitude smaller than the affordable numerical grid spacing. It is thus common to neglect $\pi _{ik}$ through setting it to zero and thus neglecting “molecular viscosity” entirely. One instead *solves the Euler equations of hydrodynamics rather than the Navier–Stokes equations (NSE)*. However, this step has an unexpected price tag. The Navier–Stokes equations are the fundamental equations of hydrodynamics for a very good reason: not only has the term $\pi _{ik}$ an essential role for the basic properties of turbulence (cf. Tsinober 2009). But much more fundamentally, the Euler equations have a much larger function space for their solutions. More specifically, their weak solutions in case of discontinuities such as shocks in general are *not unique*, as holds for nonlinear hyperbolic equations already in the case of a scalar equation, see Chap. 14.1.4 in Quarteroni and Valli (1994), even more so for the coupled set of the five components of (42a)–(42c). In contrast, the NSE “automatically” fulfill the first and second law of thermodynamics which is *not* the case for (42a)–(42c) when $\pi _{ik}=0$. Indeed, *already for the basic Riemann problem there are cases with an infinite number of solutions* (Quarteroni and Valli 1994). To pick a unique solution which also is compatible with thermodynamics one has to enforce an additional constraint: the (weak) solution has to be that one which is obtained from the full NSE (i.e., $\eta > 0$ and $\pi _{ik} \ne 0$) in the limit of $\eta \rightarrow 0$ (if a strong solution to (42a)–(42c) exists, it is smooth by definition and the non-uniqueness problem is not encountered). This solution is hence also called *entropy solution*. It is compatible with thermodynamics and its existence and unique dependence from the initial conditions (for physically relevant hyperbolic conservation laws) has been shown in the mathematical literature (for references see Quarteroni and Valli 1994). As a result, physically and mathematically useful solution methods for (42a)–(42c) have convergence to the entropy solution as a built-in property. We return to these issues in Sect. 5.2.4 in the context of Riemann solvers.

Thus, the solution methods are all subject to some kind of “viscosity” (schemes which do not will rapidly crash in simulations that develop shocks). Some schemes are based on “artificial viscosity” which is constructed such as to be formally similar to the physical terms such as $\pi _{ik}$. However, their coefficients are orders of magnitude larger than the physical ones which in turn is the justification to neglect $\pi _{ik}$ itself. Other numerical methods have some sort of diffusivity built in by basic design, not necessarily recognizable at the first glance. In one way or another, *viscosity is in practice always included in the scheme*, although one might basically strive to *keep it as small as possible*.

### Equations for low-Mach-number flows

All of the modified equations described in the following (with the exception of those discussed in Sect. 4.5) set out from a basic, horizontally averaged configuration where physical quantities are a function of depth (*x* or $x_1$) only. At least in the applications to stellar convection, this *background state* is usually assumed to be hydrostatic. Convection is then represented by perturbations of the background state and approximate equations describing the dynamics of these perturbations are derived.

#### The Boussinesq approximation

The Boussinesq approximation can be considered as a simpler variant of the anelastic approximation discussed below (Sect. 4.3.2). In order to be valid it requires, however, more stringent conditions regarding the object or simulation domain to be investigated than applies for the anelastic approximation. These conditions are that the layer thickness (in terms of pressure scale height for practically all cases) and the relative horizontal variations of physical quantities are ${\ll }1$ (Spiegel and Veronis 1960). A consequence thereof is that the ensuing velocities have to be small compared to the speed of sound. The resulting mathematical simplicity has made this approximation useful for early investigations, in particular for work on stability. See, e.g., Spiegel (1971). However, against what the Boussinesq approximation would require, stellar convection zones are, in general, not thin, and if they are, such as the hydrogen ionization zone of A-type stars, velocities and horizontal fluctuations can happen to be quite large (Steffen et al. 2005; Kupka et al. 2009), even supersonic.

Still, at least in investigations of semiconvection the Boussinesq approximation is useful even today (see Mirouh et al. 2012; Zaussinger and Spruit 2013), as its underlying assumptions are fulfilled in interesting cases here.

For presenting the Boussinesq equations we set out from a plane-parallel basis configuration which is slightly perturbed as described above. We denote horizontal means by an overbar and deviations from it by primes. So, we have for the example of pressure,

47 $$\begin{aligned} p(\varvec{x},t)=\overline{p}(x_1)+p^\prime (\varvec{x},t). \end{aligned}$$

We assume that the basic state is constant in time and at rest. Therefore, we have for velocity $\varvec{u}=\varvec{u}^\prime$, and we omit the prime in that case. Then, under the basic assumptions of the Boussinesq approximation the continuity equation, Eq. (42a), can be shown to reduce to

48 $$\begin{aligned} {{\mathrm{div}}}\varvec{u}= 0, \end{aligned}$$

which is formally equivalent to the fact that for an *incompressible* flow the velocity field is divergence free, i.e., just this condition holds true.

Density *variations* need, under these basic assumptions, to be retained only where they couple to gravity. Subtracting the momentum equation for the basic, static configuration (i.e., the condition of hydrostatic equilibrium), what is left from the momentum equation reads

49 $$\begin{aligned} \partial _{t}\varvec{\mu } +{{\mathrm{div}}}(\rho \varvec{u}\otimes \varvec{u}+ p^{\prime }\varvec{I})- {{\mathrm{div}}}\varvec{\pi } = \rho ^{\prime }\varvec{g}. \end{aligned}$$

To proceed we now write it as an equation for velocity $\varvec{u}$, use that $\rho ^\prime /\rho \approx -T^\prime /T$, and omit the diffusive terms for ease of notation:

50 $$\begin{aligned} \partial _t \varvec{u}+ {{\mathrm{div}}}{\left( \varvec{u}\otimes \varvec{u}+ \frac{1}{\rho }p^\prime \varvec{I}\right) } = -\frac{T^\prime }{T} \varvec{g}. \end{aligned}$$

The energy equation may in this case most clearly be written as a temperature equation,

51 $$\begin{aligned} \partial _t T + \sum _{i=1}^3 u_i\partial _{x_i}T -\chi _T\varDelta T = 0, \end{aligned}$$

where $\chi _T$ is the (radiative) temperature diffusion coefficient. –This last equation just expresses that the temperature field changes, in the comoving sense, according to a heat conduction equation.

In working numerically with the Boussinesq approximation, there is an essential structural difference to the original Euler or Navier–Stokes equations. Similar to them, $\varvec{u}$ and *T* (or $T^\prime$) obey the time-evolution Eqs. (50), (51). The continuity equation, Eq. (42a), originally a time evolution equation as well, appears now as a constraint, Eq. (48). When advancing the solution in time numerically, $p^\prime$ (or *p*) must therefore be determined in a way which leads to a divergence free velocity field at later times, or the divergence condition has to be satisfied in other ways. This has a bearing on the numerical treatment to which we will turn later on.

#### The anelastic approximation

During the last few decades, the anelastic approximation has been the tool most frequently applied for modelling (deep) stellar convection. Like the Boussinesq approximation, it filters out sound waves. Contrary to the Boussinesq case it allows the investigation of convection occurring in a truly layered background stratification. The background is described by functions ${\overline{\rho }}(x_1),\ldots$. We assume it to be in hydrostatic equilibrium and at rest although provisions for slow changes in the background stratification can be made.

There are two assumptions at the heart of the anelastic approximation. Firstly, the relative fluctuations of the thermodynamic quantities $p^{\prime },\ldots$ around their mean state must be small, and so must be the Mach number of the ensuing flow. Secondly, the flow must not exhibit time scales faster than the crossing time of the domain (with typical flow velocities).

For the case of adiabatic flows in a layered atmosphere the anelastic equations have been derived in Batchelor (1953) based on physical arguments. Later on, these equations have been derived as the first set of equations beyond the trivial ones (hydrostatic equilibrium) in the sense of a perturbation approach in Ogura and Phillips (1962).

To catch the gist of the approach, let us first consider the continuity equation, Eq. (42a), for modification. Since $\partial _t{\overline{\rho }}=0$ (we assume the basic configuration to be static) we can write it in the form

52 $$\begin{aligned} \partial _t\rho ^{\prime }+{{\mathrm{div}}}{{\overline{\rho }}\varvec{u}}+{{\mathrm{div}}}{\rho ^{\prime }\varvec{u}}=0. \end{aligned}$$

The relative fluctuations are assumed to be small, $\rho ^{\prime }/\rho <\epsilon \ll 1$ etc., where we understand such inequalities always as in magnitude. We similarly require the Mach number *M* to be small, $M < \epsilon$. If the nontrivial spatial scales are some reasonable (not overly small) fraction of the vertical extent of the system, *L*, there results a characteristic time $\tau$ for changes of $\rho ^{\prime }$, namely $\tau =L/M$. Under these assumptions, the most important remaining part of the continuity equation can easily be identified. Just for one moment we consider density scaled by ${\overline{\rho }}$ and velocity by speed of sound. Then the three terms in (52) are, in turn, of order $O(M/\tau )=O(\epsilon M/L), O(M/L)$, and $O(\epsilon M/L)$. So we retain

53 $$\begin{aligned} {{\mathrm{div}}}{{\overline{\rho }}\varvec{u}}=0 \end{aligned}$$

as the continuity equation to be used in the anelastic approximation.

In a sense, this is the most fundamental change applied to the Navier–Stokes equations. Whereas the original Navier–Stokes equations are evolution equations for the basic variables, the continuity equation in the anelastic formulation acts as a constraint to the other equations which still are evolution equations (see below). It is also this form of the continuity equation which prevents the derivation of the wave equation obeyed by the sound waves and, in fact, eliminates the sound waves.

There are various forms of the anelastic equations. Below we will describe a little more closely some of them whose properties have been discussed and compared recently in the astrophysical literature. We start, however, with a version which has been used in early studies of solar granulation and which illustrates the use of these approximations quite well.

#### Nordlund’s approach

Let us sketch a way of how to proceed with the anelastic equations by referring to an early and influential paper on solar granulation by Nordlund (1982). Here, a Poisson equation for the pressure is derived from the anelastic continuity Eq. (53) together with the momentum equations:

54 $$\begin{aligned} \varDelta p = \nabla \left( \rho \varvec{g}-\rho (\varvec{u}\cdot \nabla )\varvec{u}\right) . \end{aligned}$$

This equation then essentially replaces the continuity equation, Eq. (53). In time-evolution, the horizontal momenta are advanced directly. In order to fulfil the basic divergence condition, Eq. (53), the ${\bar{\rho }} \varvec{u}_x$ (vertical) component is obtained by integrating Eq. (53) from the bottom ($x_\mathrm{bot}$) up to any depth *x*, obtaining

55 $$\begin{aligned} ({\bar{\rho }} \varvec{u}_x)|_{x_\mathrm{bot}}^x(\cdot ,y,z)=-\int _{x_\mathrm{bot}}^x(\partial _y{\bar{\rho }}\varvec{u}_y+\partial _z{\bar{\rho }}\varvec{u}_z))(\xi ,y,z)\,d\xi , \end{aligned}$$

which allows evaluation of $\varvec{u}_x$ at any point provided this quantity is prescribed at the lower boundary (for example set to 0 invoking impenetrable boundary conditions). Incidentally, in this paper Fourier expansion is used in the horizontal directions for easy solution of the Poisson equation, and the basic procedure is actually carried out in the (horizontal) Fourier description. Since the pressure equation contains the term $(\varvec{u}\cdot \nabla )\varvec{u}$ some iteration between pressure and velocity field at any time step is required.

Solar granulation is surely not the physical phenomenon of choice to be modelled via the anelastic approximations since the basic requirements are violated, for example, that the Mach number be small. Nevertheless, in the early days of granulation modelling (where the anelastic approximation was chosen for numerical viability with computers of that time) fundamental properties of granulation which have stood the test of time have been unravelled in that way.

#### Several types of anelastic approximations

The “anelastic approximation” is by no means a single, well defined set of equations derived from the Euler or Navier–Stokes equations. At the heart of that matter is the fact that the anelastic approximation cannot be obtained from the Navier–Stokes equations by an expansion where, then, only the low-order terms are retained. For reasons to be discussed below there exists a considerable variety of flavours. More often than enforcing the divergence condition in the way outlined above it is derived by specific forms of expansions involving a small parameter connected with the departure from the basic, initial structure. However, such theories where the argumentation ultimately leads to the divergence condition (53),

$$\begin{aligned} \nabla {\bar{\rho }}\varvec{u}= 0, \end{aligned}$$

are considered to belong to the class of anelastic approximations. Already here, however, variants of the anelastic approximation divert from each other when it comes to the precise meaning of ${\bar{\rho }}$. This quantity may either refer to the density of the initial, closely adiabatic layering, or to the horizontal average of the density as it evolves during the simulation.

Substitution of the continuity equation proper by Eq. (53) precludes evolving the density fluctuations $\rho ^\prime$ *via* the continuity equation which naturally would determine that quantity. Rather, one has to resort to the energy equation (basically) to evolve, for example, temperature, and to use that quantity to derive a term for the momentum equation which contains the effect of $\rho ^\prime$ on momentum.

At the beginning of the anelastic approximation there is the “original anelastic approximation” due to Ogura and Phillips (1962). It extended the condition $\nabla {\bar{\rho }} \varvec{u}= 0$ which had been derived by Batchelor (1953) by a momentum and energy equation. In their derivation, the basic assumptions are that the variation of potential temperature $\varTheta$ across the layer considered is small (so that, in practice, the layering is close to adiabatic) and that the time-scales to be retained are ${>}1/N, N$ denoting the Brunt–Väisälä frequency.

Gough (1969) has allowed for a possibly superadiabatic and time-varying background and, in particular, included radiative transfer as described by the diffusion approximation.

Lipps and Hemler (1982) allow for a slowly varying potential temperature of the background configuration as a function of depth by performing a rigorous scale analysis as opposed to more physical reasoning present in many other papers. A further set of anelastic equations is due to Bannon (1996). This paper gives a heuristic derivation of the equations after having stated the physical assumptions and devotes also attention to overshooting motions and waves.

An anelastic approximation often used in the astrophysical context is due to Gilman and Glatzmaier (1981). In that paper the equations are derived in polar coordinates with a spherical shell in mind.

Later on, that approach has been extended to also include magnetic fields (Glatzmaier 1984). In this model a diffusive energy flux based on the entropy gradient is incorporated. It is supposed to represent the effects of subgrid scale motions. The picture behind it is that the starting model is already nearly isentropic. A more common use of a subgrid flux based on the temperature gradient (i.e., more or less invoking the diffusion approximation for radiative transfer) might counteract this basic assumption. Rather, it is the small scale motions which are assumed to homogenize the material in the sense of entropy at the small scales. More recently, for purposes of modelling overshoot beyond a convection zone, the assumptions on the diffusive term have been changed, this time allowing for a strongly non-isentropic basic state. Now, a diffusion term again based on the temperature gradient is invoked in order to achieve an outwardly directed diffusive flux even in subadiabatic layers (Rogers and Glatzmaier 2005).

“*The different notation and the different thermodynamics used in the various anelastic treatments leads to some confusion*”, as stated in Brown et al. (2012). Other differences add to the diversity. The basic layering may be assumed close to adiabatic or not. The equation of state, for example, may be applied in its exact or the linearized version. An ideal equation of state may be assumed and basically enter the derivations. When in spherical geometry, some metric terms in the differential operators may be disregarded. Since much of the material comes from meteorology, atmospheric moisture may be implemented in a specific way. Furthermore, different assumptions or approximations abound in the derivations.

For the benefit of the reader who might wish to more closely get acquainted with the essence of the anelastic approximation, we direct attention to two papers which each approach the topic in stylistically quite a different way and which both feature good readability.

Verhoeven et al. (2015) consider a simple physical system. They assume a layer with fixed, impenetrable boundaries at the top and the bottom with a temperature difference between them being held constant. The gas obeys simple microphysics and other idealizing assumptions, for example a constant heat conductivity. The setting is such that for the adiabatic part hydrostatic equilibrium applies. One essential control parameter, $\epsilon$, is a normalized measure of the superadiabatic part of the temperature jump between top and bottom. Decomposing density in the adiabatic (static) and superadiabatic part (in the form ${\rho }_\mathrm{ad}$ and $\epsilon \rho _\mathrm{sad}$) the (exact) continuity equation can then be written in the form

56 $$\begin{aligned} \epsilon \partial _t\rho _\mathrm{sad}+{{\mathrm{div}}}(({\rho }_\mathrm{ad}+\epsilon \rho _\mathrm{sad})\varvec{u}) = 0, \end{aligned}$$

which shows that with $\epsilon \rightarrow 0$ the time-derivative of the fluctuating density looses importance. This ultimately filters out sound waves and leads, in the limit, to the usual anelastic constraint, Eq. (53), on the velocity field, derived here in a somewhat different way than we have done earlier on. The other anelastic equations can be obtained similarly, the arguments on what to drop out being more involved, however. The equations for $\rho _\mathrm{sad}, \varvec{u}$ and $T_\mathrm{sad}$ (using an analogous decomposition of temperature) turn out to be independent of $\epsilon$ in their setting. $\epsilon$ appears only as a scaling factor for the ultimate density, $\rho _\mathrm{ad}+\epsilon \rho _\mathrm{sad}$, and temperature, $T_\mathrm{ad}+\epsilon T_\mathrm{sad}$.

Incidentally, by letting another control parameter, *D*, which is a normalized measure of the depth of the system, tend to zero, the Boussinesq approximation can be derived.

The second paper we want to address here is due to Lantz and Fan (1999). It provides, firstly, a short but thorough discussion of basics of mixing length theory and then proceeds deriving a variant of the anelastic approximation, pointing out conceptual similarities to many aspects of mixing length. It furthermore works out the anelastic approximation via a *scaled expansion*. Quite illuminating is a detailed discussion of questions on proper scalings which ultimately also pertain to the range of validity of the approximation. There are also various algorithmic items being discussed. Among them is the question how the usual Poisson equation for the pressure (perturbation) can be obviated and how basically just *one* thermodynamic variable needs to be advanced in time provided that one is ready to accept a subgrid scale model plus the physical assumption of near-adiabaticity of the atmosphere under which a term involving fluctuating pressure can be dropped.

#### The anelastic approximation: tests

The discussion above obviously triggers the question about closer information on reliability and efficiency of the anelastic approximation. Such investigations can be conducted along different lines. Assessments of validity (other than scrutiny of the assumptions underlying a specific variant of the approximations) are undertaken either by numerical integration of benchmark cases, using the full (nonlinear) anelastic equations, or by linearizing them about the static state, checking eigenmodes and eigenfrequencies predicted in that way against those derived from the full Navier–Stokes equations.

Specifically, one may set out from a convectively unstable layer and let it evolve either under the full equations or the anelastic approximation. Such an investigation, already alluded to above, has been carried out by Verhoeven et al. (2015). As mentioned already, they assume a box with a gas held at fixed temperatures at top and bottom and adopt simple microphysical coefficients (ratio of specific heats $\gamma =5/3$, constant radiative conductivity, etc.).

One essential control parameter in that work is $\epsilon =\varDelta T/T_{\small {\mathrm{bottom}}}$, i.e., the ratio of the $\varDelta T$, namely the superadiabatic part of the temperature difference between bottom and top, to the temperature at the bottom. In addition, the Rayleigh number is varied (ranging from $10^4$ to $10^7$ in these tests). Furthermore, there is the Prandtl number (assumed to be 0.7) and a parameter characterizing depth.

Otherwise identical models are calculated invoking the anelastic approximation as discussed around Eq. (56) above on the one hand and, on the other hand, solving the full Navier–Stokes equations. In a nutshell, it is found that for $\epsilon =0.3$ global diagnostic quantities (such as heat flux, velocity or kinetic energy, all averaged over the domain) deviate by about $30\%$ from their anelastic counterpart. For smaller values of $\epsilon$ they converge approximately linearly to their anelastic value when decreasing this parameter. In more superadiabatic situations ($\epsilon >0.3$) this approximately linear scaling breaks down. Somewhat against expectation, larger density contrasts reduce the deviations between results based on the full equations and the anelastic approximation, respectively, under otherwise similar parameters.

In a related discussion, Brown et al. (2012) focus on somewhat different aspects, namely the question how well the anelastic equations perform in regimes away from the ones under which they have frequently been derived, namely under the assumption of a nearly isentropic stratification. In particular, subadiabatic stratifications are in the focus of interest there and attention is directed towards the modelling of gravity waves.

Meritoriously, the three different anelastic prescriptions considered (plus the original hydrodynamic equations) are cast into a consistent form (essentially their Table 1). Specifically, in one brand of anelastic equations dealt with (ANS for short) the momentum equation is the same as in the original fluid dynamic equations. The second set is the Lantz–Braginsky–Roberts (LBR) formulation where, in comparison to the full equations, a term including the entropy gradient of the reference atmosphere is being dropped (which amounts to the explicit assumption of an isentropic basic layering). The third variant is the Rogers–Glatzmaier (RG) formulation (Rogers and Glatzmaier 2005).

In that paper, the approaches mentioned above (linearization and working with the nonlinear equations) are both considered. Regarding the linearized version, it turns out that the ANS (and probably also the RG variant which has been investigated in less detail) yields unsatisfactory eigenfunctions or eigenfrequencies for gravity waves in a physically simple case (isothermal atmosphere). The LBR variant fares better and is recommended already on behalf of that fact. The authors ascribe the different behaviour to issues of energy conservation: for the LBR equations, a conservation principle for energy (kinetic $+$ potential) can be derived, whereas for the ANS equations a similar principle applies only to a sort of pseudo-energy (where in the integration an additional factor, depending on the layering of the basic structure, appears). For the RG equation, proper energy conservation is granted only for infinitesimally small amplitudes (setting aside rather specialized other cases). The basic finding concerning the necessity of energy conservation properties of schemes is corroborated by the fact that an investigation of a rather different basic scenario (a non-hydrostatic model of the moist terrestrial atmosphere) similarly stresses its importance for veracity of the results (Bryan and Fritsch 2002). Mass conservation alone is not sufficient.

As a result (also of their nonlinear simulations) the authors recommend to alter one’s anelastic system so as to enforce energy conservation if necessary, namely by modifying the momentum equation, even if that should mean to violate momentum conservation.

#### The anelastic approximation: considerations

A number of points raised above make it clear that the anelastic method’s applicability and the proper choice of a variant is in general a matter not decided easily. On the positive side, it seems that such methods may work properly also in regimes where one would not expect this from the outset (see for example the remarks on the LBR model above).

Applicability of the anelastic equations in the case of rapidly rotating systems has been questioned by Calkins et al. (2015). Their analysis pertains to the case of low Prandtl- and high Taylor number configurations (the Taylor number Ta is the square of the ratio between Coriolis and viscous forces). The analysis performed in that paper shows that, for sufficiently low or high values of those numbers, respectively, *linear* motions (which are the ones with which the paper is dealing) are such that the Mach number is small, yet, in basic contrast to the assumptions of the anelastic approximations, the derivative $\partial _t\rho ^{\prime }$ is crucial in determining the ensuing motions. The $\partial _t\rho ^{\prime }$-term being large comes about with a concomitant change in the basic structure of the flow with increasing Taylor number. For small rotation rates, the force balance is essentially between pressure, viscosity and buoyancy forces. With larger rotation rates, this changes to a balance between pressure, Coriolis and inertial forces horizontally. For still higher rotation rates, a geostrophic force balance (Coriolis vs. pressure force in the horizontal) applies. That naturally casts doubts on *nonlinear* results concerning such systems (Verhoeven and Glatzmaier 2017). They confirm that close to the stability limit of convection the anelastic approximation is inaccurate. (The pseudo-incompressible approximation, described in Sect. 4.4, performs better there, but does a bad job in other physical regimes.) However, for fully developed turbulent convection their simulations show good agreement between the anelastic approximation and results from the full Navier–Stokes equations.

An investigation by Wood and Bushby (2016), addresses the *onset* of convection in the case of rapid rotation, low viscosity and low Mach number. In that case convection is known to often be oscillatory. When comparing linearized results of the Euler equations for the system at hand with those based on the Boussinesq approximation or a number of anelastic approximations it turns out that, with one exception, all these are unsatisfactory in that they yield valid results only for unexpectedly small ranges of parameters (such as the height of the domain), if at all. The limitations are more severe than those upon which the approximations are built anyway from the outset. So, for an ideal gas, the ratio of the domain height to the usual scale heights (pressure, $\ldots$) must be smaller than the Prandtl number for validity of the results. That renders most schemes useless for that purpose. Only a specific variant of a soundproof system (these are discussed in the following subsubsection) fares better. For clarity we should note that the analysis pertains to *oscillatory* convection only, not to direct convection. Anyway, these results corroborate the view that validity of the anelastic approximation cannot be taken as granted when moving into new physical regimes.

Klein et al. (2010) point to a problem which previous studies have paid attention but scarcely. (For example, Ogura and Phillips (1962) check in their version of the anelastic equations that the time-scale of the Brunt–Väisälä frequency, which separates acoustic and gravity waves, is respected). The general point is that, depending on the basic structure of the layer, the time-scales for sound waves, internal gravity waves and advection may happen to fulfill $t_\mathrm{snd} \ll t_\mathrm{grav} \ll t_\mathrm{adv}$. Consequently, the question arises whether gravity waves are represented correctly by any specific set of anelastic equations which do not explicitly address the fact that *three* rather different time-scales may be present. By analyzing the linear properties of the anelastic models versus those of the full Navier–Stokes models the paper provides ranges (in terms of degree of layering) within which the anelastic models perform faithfully in this respect. At the same time, the range of validity (in terms of strength of layering) of the early Ogura–Philips model (Ogura and Phillips 1962) is extended considerably beyond the original estimate. These results allow the applicability of the anelastic equations for a specific problem to be assessed more reliably.

A different issue is that many of the simulations referring to convection in rotating shells (having the Sun or solar-like stars in mind) actually make use of a Prandtl number of *O*(1). That may, on the one hand, be enforced by numerical considerations. On the other hand, such a choice may be interpreted as referring to a “turbulent Prandtl number”, i.e., in the sense of subgrid modelling, despite of the criticism such a concept may experience. In applications, the turbulent Prandtl number is often chosen from a practical point of view, such as keeping the numerical method stable with its smallest possible choice rather than referring to theoretical or experimental considerations for its value for sheer want of these in the astrophysical regime. That point naturally applies to any simulation of the sort we have in mind here, not only those based on the anelastic equations. Simulations using a relatively low Prandtl number (down to ${\mathrm{Pr}}=0.125$) can, for example, be found in Brun and Toomre (2002).

### The pseudo incompressible and the low Mach number model

#### Basics

Durran (1989) has further developed the anelastic approximation to what is being called the *Pseudo Incompressible approximation* (PI for short). The basic assumptions are that the Mach number is small and that, this time, the normalized horizontal *pressure* fluctuations are small as well. This rests on the observation that, after all, the *pressure* perturbations are responsible for sound waves. Such an approach has proved useful earlier on in problems of combustion.

It is requested that the (Lagrangian) time-scale of the disturbances are large in comparison with the time-scale of sound propagation. Furthermore, an idealized equation of state is assumed. Both, temperature and density fluctuations about the horizontal mean are, however, not requested to be small.

In the pseudo incompressible approximation the anelastic velocity constraint, ${{\mathrm{div}}}{{\overline{\rho }}\varvec{u}}=0$, Eq. (53), is now replaced by

57 $$\begin{aligned} {{\mathrm{div}}}{{\overline{\rho }}\,{\overline{\varTheta }}\,\varvec{u}}=RHS, \end{aligned}$$

where $\varTheta$ denotes the potential temperature and the right hand side *RHS* involves quantities depending on the horizontally averaged state. The pseudo incompressible approximation has been extended to also be applicable to a time-varying background by Almgren (2000).

In deriving what is termed the *Low Mach Number model*, Almgren et al. (2006) start from essentially the same basic assumptions on which the pseudo incompressible approximation rests. They allow, however, for a nontrivial equation of state which has been assumed in the derivation and algebra in Durran (1989). In the Low Mach Number model, the basic velocity constraint now reads

58 $$\begin{aligned} {{\mathrm{div}}}{{\beta _0}({x_1})\varvec{u}}=RHS, \end{aligned}$$

where the function $\beta _0$ depends only on depth $x_1$ and involves the horizontally averaged structure and the equation of state. The non-trivial right-hand-side takes effects of compressibility into account (heating, change of the horizontally averaged structure).

In the numerical realization of the system as sketched in Almgren et al. (2006) an elliptic equation is derived for the pressure, similar to the typical procedure with the anelastic approximation. When marching forward in time, basic hydrodynamic variables are first evaluated for the new time level. Then, at this new time level, the pressure equation is solved an used to change the provisional velocity field to one obeying the velocity constraint Eq. (58). In such a way, the method is implemented in the MAESTRO code (Almgren et al. 2006; Nonaka et al. 2010).

Vasil et al. (2013) developed what they call the *generalized pseudo-incompressible model* (GPI). Unlike PI, GPI enjoys energy conservation (not just conservation of a pseudo-energy) and applies also to a more general equation of state than PI does.

#### Tests and considerations

Some tests of the Low Mach Number approach are provided in Almgren et al. (2006). A rising bubble, originally in pressure equilibrium horizontally (but with appropriate perturbations of density and temperature) is considered in the anelastic approximation, the Low Mach Number model and the full hydrodynamic equations. In a sense, working with a Low Mach Number model may be easier than working with the full equations for reasons other than the mere low Mach number of the situation: discretizations of the full equations excite pressure perturbations which may spoil the solution unless countermeasures are taken. Naturally, such difficulties do not arise when working with the approximate equations.

The importance of conserving energy rather than just some pseudo-energy is emphasized again in Vasil et al. (2013). This is exemplified by the spatial structure of eigenfunctions referring to gravity waves in a layered medium, where the Low Mach Number approach yields unsatisfactory results. In a similar vein, the energy flux (as a function of height) in an isothermal layer is constant in the Navier–Stokes and PI/GPI approaches, whereas it diverges near the top of the layer for the Low Mach Number method. Yet, all simplifications of the Navier–Stokes equations seem to have difficulties in reproducing the correct dispersion relations for gravity waves in one part of the parameter space or the other.

Assessment of veracity of the various equations (anelastic, Low Mach, PI) is rendered to be quite delicate a matter according to work by Lecoanet et al. (2014). Here, in simulations basically investigating the kind of diffusion one should preferably include for subgrid modelling, it is the anelastic approximation which surprisingly performs better than the PI approach for a specific case. For that odd behaviour an explanation, based in specific aspects of the PI equation of state, is offered. Anyway, that teaches one how easily, in that area, rather subtle causes may lead to unexpected and possibly wrong results.

#### The quasi-hydrostatic approximation

In meteorology, the quasi-hydrostatic approximation is frequently used. It addresses phenomena of long *horizontal* wavelengths as compared to *vertical* wavelengths, i.e., the vertical extent of the atmosphere, so that for the grid-spacings one has $h_\mathrm{vert}\ll h_\mathrm{horiz}$. It then makes sense to suppress the *vertical* sound waves and to thus eliminate their stringent effect on the numerical timestep. Horizontally, some acoustic waves are admitted (Lamb waves).

In the quasi-hydrostatic approximation this is achieved by assuming balance of pressure and gravity forces in the depth-direction, i.e., $\partial _{x_1}p=-\rho g$. This brings about useful relations between $D_t\rho$ and $D_t p$ (and hence $\partial _t \rho$ and $\partial _t p$). Making, in addition, use of the equation of state one ultimately arrives at *one* evolution equation for a thermodynamic quantity and evolution equations for the *horizontal* velocity components. The vertical (slow) velocity component is obtained from a diagnostic equation at the new time level.

For a closer description of this approximation consult, e.g., Kasahara and Washington (1967) and Arakawa and Konor (2009). For astrophysical applications (e.g., stellar radiative zones) this method has recently been extended to even the MHD case and a code has been developed. See Braithwaite and Cavecchi (2012) and Boldt et al. (2016).

We explicitly point out that we have included a short description of that approach just for granting easy access to the most basic issues. It is clear that the quasi-hydrostatic approximation is not suited for convection simulations in the astrophysical context. Already the condition $h_\mathrm{vert}\ll h_\mathrm{horiz}$ for obtaining a reasonably sized time-step is in conflict with typical convection simulations where horizontal length scales simply are, as a rule, smaller than or comparable to vertical ones. There are also theoretical issues which render the method to be valid only for waves with horizontally large wavelengths. About the precise limitations there is an ongoing debate even in meteorology.

### The reduced speed of sound technique

The basic point here is to change the continuity equation, Eq. (42a), to

59 $$\begin{aligned} \partial _t\rho +\frac{1}{\xi ^2}{{\mathrm{div}}}{\rho \varvec{u}} = 0 \end{aligned}$$

by introducing a parameter $\xi , 0<\xi \le 1$. That reduces the speed of sound by the factor $\xi$ and alleviates time-step restrictions imposed by the sound speed. Hotta et al. (2012) provide tests of the method for a zone unstable against convection. As described there, some approximations are also made in the momentum and energy equations.

Unlike the methods discussed up to now an algorithmic advantage is that there is no need to solve an elliptic equation, at least if not the size of the coefficients in the viscous or dissipative terms dictates that either from the outset or when applying the larger time-step permitted with some choice of $\xi <1$.

In practice it may be necessary to apply a depth-dependent value for the parameter, $\xi =\xi (x_1)$, in order to obtain acceptable time-steps. In that case the continuity equation, Eq. (42a), is no longer in conservation form. At least in some tests given in the paper loss of mass conservation is not deemed bothersome. The authors prefer tolerating it in favour of using a conservative form for the modified continuity equation, *viz.* $\partial _t\rho +{{\mathrm{div}}}{\frac{1}{\xi ^2}\rho \varvec{u}} = 0$. The argument is that the true continuity equation, Eq. (42a), has the same stationary solutions as the modified form given in Eq. (59). That would not hold true for the conservative modification. For this method the energy (internal $+$ kinetic) does not strictly obey a conservation law.

A variant of the method which fares better in terms of energy conservation (it conserves energy in the linear approximation around an adiabatic base state) is described by Hotta et al. (2015). The method has also been applied for investigations of the MHD case for rotating (quasi-solar) convection.

### The “stratified” method

The “stratified” method (Chan et al. 1994; Chan 1994; Cai 2016) evolves the problem *via* a time-splitting approach. Basically, *linear* waves are integrated in time via an implicit method, the rest explicitly. If (as is the purpose of the method) the time-step used is substantially larger than explicit time-marching would allow, the implicit time integration represents the linear waves (acoustic, possibly gravity) not accurately, but stably, thus avoiding the numerical instabilities which make explicit time-marching unfeasible.

In addition to that basic splitting concept, two approximations are made. The horizontal variation of density is ignored in the momentum equation (outside of the linear advection terms) and only linear terms in the horizontal variation of the thermodynamic variables are retained in the energy equation. As a consequence, more of the original terms are retained than holds true for the anelastic approximation. In particular arguments are given for the benefit in accuracy when retaining the $\partial _t\rho$-term in the continuity equation.

In the papers just cited the method is implemented for the spherical case (using spherical harmonics in the lateral directions and finite differences vertically) and in Cartesian coordinates (using expansions via trigonometric functions horizontally and Tchebycheff polynomials vertically). The implicit treatment of the terms for linear waves requires the solution of a block-tridiagonal system of linear equations. Numerical difficulties plus additional computational overhead in the spectral methods seem to have motivated the neglect of products with three factors in the variations mentioned above.

It is argued in Cai (2016) that in comparison to the reduced speed of sound technique several terms are retained in the “stratified” approach. Results for a low Mach number convective test case (a slightly superadiabatic zone with idealized physical parameters) shows good agreement in horizontally averaged quantities between calculations based on the the reduced speed of sound technique as given in Hotta et al. (2012) and the “stratified” calculations from Cai (2016).

### Changing the model parameters

As mentioned on several occasions, it is impossible in our area to produce models which truly match the physical parameters of the star they purport to describe. This holds true in particular for molecular diffusivities. Their numerical counterpart must, in one way or the other, always be much higher than microphysics of the stellar material would dictate.

Here we turn to a different, purposeful change of *model parameters* as compared with the physical object. The aim of the many different approaches we have described above is to avoid the difficulties brought about by the speed of sound in the case of low Mach number convection by *changing the equations*. The aim of such a deliberate change of parameters, in contrast, is to allow affordable simulations *using the unaltered Navier–Stokes equations*, yet to preserve important characteristic quantities such as the Rossby number in rotating convection. Thus, the complications and uncertainties brought about by working with modified equations would be avoided. The immediate practical benefit is the possibility to make use of the whole arsenal of numerical methods which have been developed for the Euler and Navier–Stokes equations. Such an approach is envisaged by Wang et al. (2015) in connection with their CHORUS code.

## Multidimensional modelling: numerical methods

### Stellar convection modelling: the numerical problem

From the standpoint of numerical modelling the main problems are these:

- *Spatial scales, extreme parameters:*
  General aspects of spatial scales, extreme parameters etc. in stellar convection have been discussed in Sect. 2.
  Just because the extremely large Reynolds number and similar parameters are inaccessible to direct numerical simulation it is mandatory to do one’s best to reach the most extreme parameters of that sort in the actual calculation in order to resolve as much of the conceivably essential small scales of the turbulent flows. Other reasons (e.g., narrow ionization fronts) may as well produce small spatial scales, calling for numerics which yields *high resolution per gridpoint* for general trustworthiness.
- *Flow properties: highly subsonic or otherwise.* A special issue encountered mainly in convection deep inside of stars (and in planetary astrophysics, rarely elsewhere in astrophysics) is, in the first place, the occurrence of very subsonic flows. For the usual methods based on the Euler equations the Courant–Friedrichs–Lewy condition limits the applicable time-step to such values that the fastest signal transverses only one grid spacing (or some fraction thereof, see also Sect. 2). Applying such time-steps, the physical processes of actual interest can virtually stall during computation, leading to impractical demands in terms of computer time. Any efficient type of numerical approach must, however, lead to a sensible change of the variables in one time-step.
  This difficulty can basically be attacked in two ways, namely:
  - *Modify* the Euler or Navier–Stokes equations in order to filter out sound waves or to reduce their speed. Approaches of that type have been the subject of Sects. 4.3–4.7. (Only in the method described in the last subsection has the goal of eliminating the computational difficulties originating from a large speed of sound been reached by keeping the basic equations but modifying the physical parameters purposefully.) The resulting equations can then be solved with numerical methods often not dissimilar to those we are going to describe in this section.
  - *Keep* the original Euler or Navier–Stokes equations and develop methods which directly cope with the problem of disparate characteristic velocities, this time at the level of the numerical method itself. In computational astrophysics that approach has been taken up relatively recently and important strides have been made. These developments fit well in the present, numerically oriented section and are therefore discussed here.
  Thus, one can choose between either a twofold approximation (approximate the equations analytically in a first step and subsequently numerically) or a onefold approximation (work on the numerical level only).
- *Geometry:* For some types of simulations it may be appropriate to work with simple box geometry. That holds true, for example, for modelling of solar granulation where one can use a small box (in terms of solar diameter) containing a small part of the photosphere including some regions below (“box in a star”).
  For simulations covering a sizeable portion of the star (a sector, a spherical shell, the whole star) the situation is different.
  For a sector not containing the center of a star it may be sufficient to work with polar or spherical coordinates. This means some marked change as compared to Cartesian coordinates. However, that change is still not as drastic as brought about by a simulation of a spherical shell or a whole sphere. The ensuing problem is then that in spherical coordinates terms of the Euler and Navier–Stokes equations have geometric factors like $\frac{1}{r\sin \theta }$ etc. ($\theta$ denoting the polar distance of the point). This leads to singularities at the center and along the polar axis. Dealing with them is numerically awkward. In addition, convergence of longitude circles near the poles leads to small grid spacing when using a rectangular $(r,\phi ,\theta )$ grid and hence unduly small timesteps. This problem can be overcome by putting a “star in a box”.
  A different approach, actually the one most often used when modelling whole spheres or spherical shells in the past, is to use a spectral method, expanding the lateral part of the dependent variables in spherical harmonics and working in this representation. The variation of these functions is distributed evenly over the sphere. The radial part can be treated by any type of discretization (with some difficulties again if the center of the star is to be included).

In addition, there is of course the problem of magnetic fields. This review deals, however, with the hydrodynamic aspects only.

### Methods for general hydrodynamics

Setting special issues aside the *numerical* challenge encountered in modelling stellar convection in multidimensions stems either from the hyperbolic terms or the diffusive terms in the Navier–Stokes equations, Eqs. (42a)–(42c). As discussed in Sect. 2, the diffusive terms call for closer consideration mainly when the diffusivities (conductivities) and the time-step dictated by flow properties conspire as to make implicit treatment of the diffusive terms unavoidable. In that case, (semi-) implicit time integration is required, incurring the necessity of solving a large linear or non-linear system of equations originating from the diffusivities. A similar problem is encountered by the Poisson equation (or a similar type of equation) appearing in the treatment of low Mach number flows (anelastic approximation, etc.).

By and large, the solution of the hyperbolic (Euler part) of the hydrodynamic equations seems, however, to be the part demanding most attention when developing a code purporting the investigation of stellar convection or similar phenomena. This is witnessed by the fact that in the publications describing the basics of the code regularly much more space is devoted to the hyperbolic part than to the (implicitly treated) diffusive terms. Still, one should not underestimate the difficulties of writing an efficient solver for those (elliptic) systems of equations. To achieve fast convergence on highly parallel machines is still a nontrivial task. On the other side, elliptic equation solvers are much more often demanded in science, engineering etc. than solvers for hyperbolic problems are called for. Therefore, we refer to the literature (e.g., the book by Trangenstein 2013) and deal here mainly with problems rooting in the hyperbolic part of the Navier–Stokes equations, i.e., the Euler equations.

Even if usually designing methods for the hyperbolic part is largely decoupled from treatment of the viscous terms (or the latter are omitted at all), specific problems pop up when dealing with the Euler equations only, indicative of the fact that one should ideally better work on the Navier–Stokes equations.

The main problem from the standpoint of numerics is that solutions of the Euler equations in general develop discontinuities, in particular shocks. These preclude naive use of the differential form of the Euler equations. Rather, one has to look for weak solutions. The problem here is, however, that in general weak solutions lack uniqueness. Admissible solutions must be found which are a limit of solutions for the viscous case with viscosity tending to 0. That puts special demands on numerics.

Furthermore, if discontinuities are present, traditional numerical methods—which are often based on Taylor expansion—lose their justification. They must implicitly contain or be equipped with a high degree of numerical or artificial diffusivity to prevent development of discontinuities in the numerical sense. That brings about massive degradation of resolution. As a consequence, much of the newer work on method development is aimed at achieving high resolution despite of the basic difficulties. We will deal here mainly with methods which, in one way or the other, address that task.

#### Numerical conservation methods: a few basic issues

The Euler or Navier–Stokes equations express (for the situation under consideration) the conservation (balance) of mass, momentum, and energy. As a consequence, the idea that one’s numerics should do likewise on the discretized level has immediate appeal. Such methods are called *conservative*. Beyond that point just mentioned there are two reasons which render a conservative approach desirable.

An obvious *practical* issue is that convection simulations typically request time-integration covering a long time (in terms of the sound crossing time of the domain) in order to arrive at relaxed, statistically stationary solutions as discussed in Sect. 2. If, for example, artificial mass loss should occur due to lack of conservation properties, its undesirable effects on the simulation are easily imagined.

A more *theoretical* consideration is that the solutions of the hyperbolic part of the equations (the Euler equations for our purpose) frequently develop shocks or other (near-) discontinuities, i.e., very steep gradients. (Numerically, a proper and a near-discontinuity cannot really be distinguished.) In particular, one rewrites equations with discontinuous solutions in the *weak form* which does not contain the first derivatives in the basic variables any longer. For a large class of problems which matter here, Lax and Wendroff (1960) have shown that *if* a numerical solution obtained by a conservative method converges at all with increasing grid-refinement, it converges to a weak solution. Conversely, Hou and LeFloch (1994) have shown that for non-conservative schemes *discontinuities in the solution must propagate at a wrong speed*. A large part of the codes used in the present area is based on conservative schemes. This holds true for codes solving the Euler or Navier–Stokes equations. For codes using the anelastic or the low Mach number approximation the situation is different due to the fact that the basic theory often does not represent conservation principles. From a more practical standpoint, the equations are frequently not formulated for the physically conserved quantities but, e.g., for velocity $\varvec{u}$ instead of momentum density $\varvec{\mu }$.

With the exception of a few more special approaches discussed later we will consider conservative methods working on a rectangular grid. For simplicity, we will assume spatial grid spacing *h* to be uniform and equal in the various coordinate directions and we will often deal with the 2D case instead of 3D for ease of notation. Besides the grid points $(x_i,y_j)=(ih,jh)$ also half-numbered gridpoints will appear, e.g., $x_{i+\frac{1}{2}}=(i+\frac{1}{2})h$. They give the boundaries of the grid cells.

In time we proceed by time-increments $\tau$, so that the *n*th time step corresponds to $t_n=n\tau$. Discretizing both in space and time, $v_{i,j}^n$ is the approximation for $v(x_i,y_j,t_n)$.

Given the form of the hyperbolic conservation law (in 2D)

$$\begin{aligned} \partial _t v + \partial _x f(v) + \partial _y g(v) = 0, \end{aligned}$$

a conservative numerical semidiscretization (in space only, time being considered later on) will be

60 $$\begin{aligned} \partial _t\bar{v}_{i,j}+\frac{\hat{f}_{i+1/2,j}-\hat{f}_{i-1/2,j}}{h}+ \frac{\hat{g}_{i,j+1/2}-\hat{g}_{i,j-1/2}}{h}=0. \end{aligned}$$

The method works on cell averages which are denoted by $\bar{v}_{i,j}$ and evaluated for the grid cell $(x_{i-1/2},x_{i+1/2})\times (y_{j-1/2},y_{j+1/2})$. The essential point at this level is the choice of the *numerical flux functions* $\hat{f}$. Obviously, $\hat{f}_{i+1/2,j}$ for example is expected to represent $\int _{y_{j-1/2}}^{y_{j+1/2}}f(v(x_{i+1/2},y))\,\mathrm{d}y$ to high order. (If we are obviously working at a single time level we may omit the argument *t* in the functions.) The numerical flux function $\hat{f}_{.,.}$ will depend on numerical values of the basic point variables *v* or cell averages $\bar{v}$, respectively.

If the method is only second order in space, there is no need to distinguish cell average values from point values since $\bar{v}_{i,j}=v_{i,j}+O(h^2)$.

Turning back to the issue of *semi*discretization, the choice of the temporal discretization is independent of the spatial discretization to some degree in most schemes. It is often performed by a Runge–Kutta scheme (see Sect. 5.5). In a few cases, the two kinds of discretizations are, however, interwoven intimately.

For clarity we want to note here that there are two ways to obtain conservative schemes in terms of variables used. Either one can use the *conserved variables* (variable densities) $(\rho ,\varvec{\mu },e)$ or the *natural* variables (density, velocities, pressure or temperature) as long as the flux function is calculated properly. The difference in using one set or the other is purely numerical since typically operations such as interpolations are being applied which may be more accurate for one set or the other.

#### Stability, dissipation

While details on time-integration will be dealt with more closely in Sect. 5.5, we want to address one basic point here. Time marching can either be done explicitly or implicitly. As the most simple case let us consider Euler forward time-integration,

61 $$\begin{aligned}&\frac{\bar{v}_{i,j}^{n+1} - \bar{v}_{i,j}^{n}}{\tau }+\frac{\hat{f}_{i+1/2,j}^{n}-\hat{f}_{i-1/2,j}^{n}}{h}+ \frac{\hat{g}_{i,j+1/2}^{n}-\hat{g}_{i,j-1/2}^{n}}{h}=0\quad \hbox {or}\nonumber \\&\quad \bar{v}_{i,j}^{n+1} = \bar{v}_{i,j}^{n}-\frac{\tau }{h}\biggl [\left( \hat{f}_{i+1/2,j}^{n}-\hat{f}_{i-1/2,j}^{n}\right) + \left( \hat{g}_{i,j+1/2}^{n}-\hat{g}_{i,j-1/2}^{n}\right) \biggr ]=0, \end{aligned}$$

i.e., *explicit* time-marching. It is not advocated here for actual use in itself, but it defines the stages of the widely used Runge–Kutta methods (see Sect. 5.5). If, instead, we make use of Euler backward time-differentiation we obtain

62 $$\begin{aligned} \bar{v}_{i,j}^{n+1} = \bar{v}_{i,j}^{n}-\frac{\tau }{h}\biggl [\left( \hat{f}_{i+1/2,j}^{n+1}-\hat{f}_{i-1/2,j}^{n+1}\right) + \left( \hat{g}_{i,j+1/2}^{n+1}-\hat{g}_{i,j-1/2}^{n+1}\right) \biggr ]=0. \end{aligned}$$

This *implicit* method requires now the solution of a nonlinear set of equations for each time step because $\hat{f}_{i+1/2,j}^{n+1}$ etc. contains the variable *v* at timestep ${n+1}$.

The advantage is that an implicit method (with really suitable choices of the type of time integration and the form of $\hat{f}$) allows larger time steps than is the case for explicit methods. For these the time step is restricted by the Courant–Friedrichs–Lewy condition (see, for example, Trangenstein 2009 and Sect. 2), so that stability is only granted for time steps $\tau$ obeying $\tau \le c \tau _\mathrm{signal}$ where *c* is a constant of order unity and $\tau _\mathrm{signal}$ the shortest time a signal (here: a sound wave) needs to cross one grid spacing. Suitable implicit methods allow much larger time steps as far as *stability* is being concerned. But that does not imply that the stable solution is also an *accurate* one. It will be so only under special circumstances. In our area such methods are useful for low Mach number flows when sound waves are physically unimportant. Then, in 1D and potentially 2D and 3D, the heavy load of solving the said system of equations may pay off due to the larger time-step one can apply. The nature of the system arising from such a discretization is unfortunately such that it does not facilitate its numerical solution. A way out of that difficulty is the use of specific *semi*implicit or preconditioning procedures as we are going to describe later on.

As a consequence, all methods we are dealing with (for normal flows, not low Mach) are explicit in the *hyperbolic* part. The diffusive terms (in particular radiative diffusivity) are, however, parabolic and give rise to an *elliptic* equation in case a (semi-)implicit time marching method is used. Indeed, implicit treatment is actually often warranted because in a number of situations these diffusive terms would impose very severe timestep limitations for explicit methods. On the fortunate side, for the resulting elliptic equations very efficient methods exist (e.g., multigrid, conjugate gradient; see, for example, Trangenstein 2013).

#### Classical finite difference schemes

In stellar hydrodynamics, classical finite difference schemes seem to be used more often in MHD than in pure hydrodynamics in the newer codes. They have the advantage of easy programming, but they also come along with some difficulties regarding stability and resolution as we will discuss shortly.

The calculation of the numerical flux function is easy. For example, in the MURaM code (Vögler et al. 2005) first derivatives in the *x*-direction are approximated by

$$\begin{aligned} v_x(x_i)\sim \frac{1}{12h}(-v_{i+2}+8v_{i+1}-8v_{i-1}+v_{i-2}), \end{aligned}$$

which leads to the numerical flux function

$$\begin{aligned} \hat{f}_{i+1/2}=\frac{7}{12}\bigl (f_{i+1}+f_i\bigl )- \frac{1}{12}\bigl (f_{i+2}+f_{i-1}\bigl ). \end{aligned}$$

Here, as always, $f_i$ denotes the physical flux function at the central (whole-numbered) gridpoint.

Together with the usual Runge–Kutta schemes for time integration this kind of spatial discretization is, however, unstable. In MURaM stabilization is achieved by (artificial) diffusivities. Such diffusive terms are added to all equations, including the continuity equation. For total diffusivity, two types of diffusion coefficients are added. One is nonzero in regions of compression and serves to keep shocks or steep gradients stable, whereas the other one is positive in all of the domain and aims to achieve general numerical stabilization.

#### Riemann solvers

A number of methods to be described below is based on solutions of Riemann problems. They can be considered both as successors of *Godunov’s method* which we deal with shortly and as application of the *upwind principle*.

**Fig. 10:**
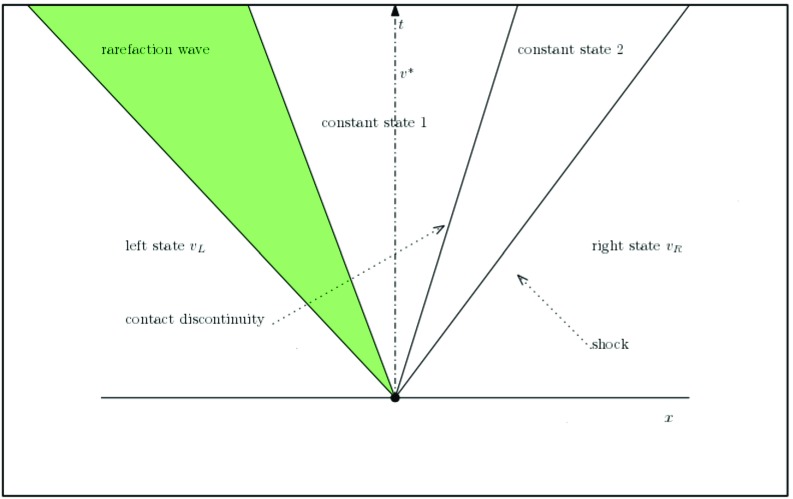
The solution of a Riemann problem for the 1D Euler equations is self-similar. There are four constant states, separated in turn by a centered rarefaction wave (across which the variables are continuous), a contact discontinuity (jump in density but not in pressure) and a shock (jump in density and pressure)

In the *Riemann problem* for Euler’s equations of hydrodynamics (in 1D) we consider, at time $t=0$, a constant left state $\varvec{v}_L$ for $x<0$ and a constant right state $\varvec{v}_R$ for $x>0$. We can expect solutions which are constant along lines $x/t=\hbox {const}$ in the $x{-}t$ plane due to the fact that the conservation law as well as the initial condition is invariant under the coordinate transformation $(\xi ,\tau )=(\theta x,\theta t)$ for arbitrary $\theta >0$. This applies therefore also to the solution, so that $\varvec{v}(x,t)=\varvec{v}(\xi ,\tau )$, whence the values at points (*x*, *t*) and $(\theta x,\theta t)$ are identical and therefore depend on $\frac{x}{t}$ only. Figure 10 shows the general structure of the solution of a Riemann problem for the 1D Euler equations of hydrodynamics, Eqs. (42a)–(42c).

$\varvec{v}^*$, the value at $x=0$, constant in time for $t>0$, is used in determining the flux function of *Godunov’s method* and other methods which we are going to describe. To proceed from step *n* corresponding to time $t_n$ to time $t_{n+1}$ Godunov’s method proceeds as follows. The solution $\varvec{v}^n$ is assumed to be constant on the intervals, namely ${\bar{\varvec{v}}}_i^n$ on the *i*th interval $x_{i-1/2}<x<x_{i+1/2}$. At the left grid point $x_{i-1/2}$ we *solve* the Riemann problem with input data ${\bar{\varvec{v}}}_{i-1}^n$ and ${\bar{\varvec{v}}}_{i}^n$. That leads to the flux function

63 $$\begin{aligned} \hat{f}_{i-1/2}^\mathrm{Godunov} = f\left( \varvec{v}_{i-1/2}^*\right) . \end{aligned}$$

As long as the signals emanating from the neighbouring cell boundaries do not arrive at that location this flux function is *exact* (to the extent the Riemann solver is) and constant in time.^7^ We consider that case and want to advance by a time increment $\tau$. For one moment, we denote the solution by $\varvec{w}(x,t_n+\tau )$ and assume further, for one moment again, that $\varvec{v}^n$ is exact. We then *average* over one interval $(x_{i-1/2},x_{i+1/2})$ and the time interval $(t_n,t_n+\tau )$ and get exactly that

$$\begin{aligned} \int _{x_{i-1/2}}^{x_{i+1/2}}\varvec{w}(x,t_n+\tau )\,\mathrm{d}x=h{\bar{\varvec{v}}}_i^n -\tau f\left( \varvec{v}_{i+1/2}^*\right) + \tau f\left( \varvec{v}_{i-1/2}^*\right) . \end{aligned}$$

Godunov’s method finishes now by a *reconstruction*, namely by defining the discrete solution at time $t_{n+1}$ by setting $\bar{v}_i^{n+1}$ as the value of $\varvec{w}(\cdot ,t_{n+1})$ averaged over the spatial interval, i.e.,

64 $$\begin{aligned} {\bar{\varvec{v}}}_i^{n+1}={\bar{\varvec{v}}}_i^{n}-\frac{\tau }{h}\left( f\left( \varvec{v}_{i+1/2}^*\right) - f\left( \varvec{v}_{i-1/2}^*\right) \right) . \end{aligned}$$

Thus, starting the cycle with the reconstruction step, it proceeds in the form *reconstruct*-*solve*-*average* (RSA).

A number of methods to be discussed later on follow essentially an RSA-scheme. As a prerequisite, we concentrate now on the *solution* of a Riemann problem. For information on Riemann problems see the classic book by Courant and Friedrichs (1999) and Toro (2013).

*Riemann problem and upwinding* Before turning towards solution avenues for the Riemann problem we consider its connection to the concept of *upwind discretization*. We set out from the simple scalar advection equation

65 $$\begin{aligned} \partial _tv(x,t)+\alpha \partial _xv(x,t)=0, \end{aligned}$$

assuming $\alpha >0$ for sake of definiteness. Since it describes a motion with speed $\alpha$, the solution of the Riemann problem with initial data $v_l, v_r$ is

$$\begin{aligned} v^*=v_l. \end{aligned}$$

Combining this with Godunov’s procedure, we obtain

66 $$\begin{aligned} v_i^{n+1}=v_i^{n}-\alpha \frac{v_i^{n}-v_{i-1}^{n}}{h}, \end{aligned}$$

so that the spatial differencing has to be biased towards the direction from where the wind blows (*upwind principle*). Central or downwind spatial differencing would not only be something different from Godunov’s method but also be unstable (with this and the usual Runge–Kutta methods for time-marching).

An essential point is that it can be shown that the above numerical approach is a better approximation to an equation containing an additional diffusive term, the diffusion coefficient depending on grid size *h* and tending to 0 if *h* does so. In other words, the Riemann strategy, i.e., upwinding, introduces a sort of *numerical diffusivity* which can be interpreted to have a stabilizing effect on the numerical solution.

Historically, upwinding was introduced very early in this area by Courant et al. (1952).

We next turn to various solution strategies for the Riemann problem.

*Exact solution* The exact solution of the problem amounts to solving nonlinear equations. This is frequently deemed expensive and may be technically difficult in the presence of Coriolis terms, radiative transfer and other complicating factors. Two widely used *approximate* solvers will therefore be discussed below. Yet, there have been strides towards efficient exact solution strategies, e.g., by Colella and Glaz (1985), and this method is implemented in the APSARA code (Wongwathanarat et al. 2016).

While from the standpoint of basic physics and general virtue the exact solution is the best one, that may not apply from the standpoint of numerics. As mentioned in context with Godunov’s method for the advection equation, the Riemann solver also acts in the sense of a numerical diffusivity. The kind and degree of diffusivity provided by the exact solution may not be appropriate for each type of basic numerics. In such a case, a more diffusive Riemann solver may be required.

*The Harten–Lax–van Leer solver* At time $t=0$ we set out with a left constant state $\varvec{v}_l$ for $x<0$ and a right one $\varvec{v}_r$ for $x>0$. In the original form the Harten–Lax–van Leer approximate Riemann solver simplifies the basic structure of the solution so that in addition to the left and the right state there is just one central, intermediate state in between of them, $\varvec{v}^{HLL}$. The intermediate state $\varvec{v}^{HLL}$ is separated from the left and right one in the (*t*, *x*)-plane by straight lines in the (*x*, *t*)-plane corresponding to wave speeds $s_l$ and $s_r$. The simplest choice is $s_l=u_l-c_l$ (where $u_l$ and $c_l$ are the velocity and sound speed in the left state), and similarly $s_r=u_r+c_r$. For other possibilities see, for example, Toro (2013). Once estimates are given for these speeds (for details see Harten et al. 1997) the intermediate state can be computed as

$$\begin{aligned} \varvec{v}^{HLL}= \frac{s_r\varvec{v}_r-s_l\varvec{v}_l+\varvec{f}(\varvec{v}_l)-\varvec{f}(\varvec{v}_r)}{s_r-s_l}. \end{aligned}$$

Plugging that value into the flux we get

67 $$\begin{aligned} \varvec{f}^{HLL} = {\left\{ \begin{array}{ll} \varvec{f}_{l} &{}\quad s_l \ge 0 \\ \varvec{f}_{r} &{}\quad s_r\le 0 \\ \frac{s_r\varvec{f}_l-s_l\varvec{f}_r+s_ls_r(\varvec{v}_l-\varvec{v}_r)}{s_r-s_l} &{}\quad s_l<0<s_r. \\ \end{array}\right. } \end{aligned}$$

The sequence of fans originating from the zero-point in the (*t*, *x*)-plane occurring in the original Riemann problem is replaced by a simpler fan structure with two discontinuities separating different areas (states). There are extensions of the original HLL method with a richer fan structure such as the HLLC approach (where a Central wave is restored) or the HLLE approach with a special method to derive the largest and smallest signal velocity (Einfeldt 1988). We again refer to Toro (2013) for details.

*The Roe solver* The solver devised by Roe (1997) is in wide use. It rests upon the following principles and considerations. *Riemann problems for linear conservation laws are easy:* Consider a hyperbolic conservation law

68 $$\begin{aligned} \partial _x\varvec{v}+\partial _x{\varvec{f}}(\varvec{v})=0, \end{aligned}$$

so that the Jacobian of $\varvec{f}$ w.r.t. $\varvec{v}, A:=\partial _{\varvec{v}}\varvec{f}$, has a complete set of real eigenvalues and eigenvectors. Then by applying the chain rule Eq. (68) can be written as

69 $$\begin{aligned} \partial _t\varvec{v}+ A(\varvec{v})\partial _x\varvec{v}= 0. \end{aligned}$$

We assume now that the conservation law is linear, i.e., that *A* is independent of $\varvec{v}$. Let *T* be a matrix which diagonalizes $A, TAT^{-1}=\tilde{A}$, the diagonal matrix $\tilde{A}$ containing the eigenvalues $\alpha _i$ of *A*. Then, with $\varvec{w}=T\varvec{v}$, Eq. (68) can be written in the form

70 $$\begin{aligned} \partial _t\varvec{w}+ \tilde{A}\partial _x\varvec{w}= & {} 0\hbox {, i.e.,} \end{aligned}$$

71 $$\begin{aligned} \partial _tw_i + \alpha _i\partial _xw_i= & {} 0 \end{aligned}$$

for the *i*th component of $\varvec{w}$. Riemann problems for such advection equations are however trivially being solved by the upwind principle as explained around Eq. (65). There remains only to recover $\varvec{v}$ using $\varvec{v}=T^{-1}\varvec{w}$. In practice, for the Euler equations the necessary eigenvalues are known (velocity $u_x$ and $u_x\pm c$ for a Riemann problem in *x*-direction, *c* being the speed of sound), and the eigenvectors follow immediately.

*Roe’s procedure* Starting from the Euler equations $\partial _t\varvec{v}+\partial _x\varvec{f}(\varvec{v}(x,t))=0$ and setting $A=\partial _v\varvec{f}(v(x,t))$ Roe has devised how to construct a matrix *R*, now called *Roe matrix*, which depends on two states, the left and the right state of the Riemann problem in practice. This matrix has the same eigenvalues as the original Jacobian, and it moreover satisfies

- consistency: $R(\varvec{v},\varvec{v})=A(\varvec{v})$ for all states $\varvec{v}$ and
- Rankine–Hugoniot property (see below): $R(\varvec{v}_l,\varvec{v}_r)(\varvec{v}_r-\varvec{v}_l)=\varvec{f}_x(\varvec{v}_r)-\varvec{f}_x(\varvec{v}_l)$.

The last equality is connected with the Rankine–Hugoniot conditions across a single discontinuity moving with speed *s*, viz.

72 $$\begin{aligned} \varvec{f}_x(\varvec{v}_r)-\varvec{f}_x(\varvec{v}_l)=s(\varvec{v}_r-\varvec{v}_l), \end{aligned}$$

so that the Roe matrix does a correct job for a single discontinuity which is physically important because the Rankine–Hugoniot conditions are expressing conservation principles according to the following considerations.

For explanation, it is sufficient to consider one component of this equation, for example the continuity equation. It reads $f_x(\rho _r,\cdots )-f_x(\rho _l,\cdots )=s(\rho _r-\rho _l)$ where $f_x$ is the flux function in *x*-direction ($\mu _x$ by the way). Why is that correct?—If *s* happens to be 0 (because we deal with a stationary contact discontinuity or a stationary shock) then we clearly must have $f_x(\rho _r,\cdots )=f_x(\rho _l,\cdots )$ which just amounts to Eq. (72) for that case, because otherwise matter would be created or destroyed at the discontinuity. If $s\ne 0$ then, now using a new coordinate system moving with speed *s*, we have, in these coordinates, a new flux function $f^*$, namely $f_x^*=f_x-\rho s$. The additional mass flux is caused by the motion of the new system with respect to the old one. In the new coordinate system, the velocity of the discontinuity is $s^*=0$, and applying the above result for a zero velocity discontinuity yields Eq. (72).

If the equation of state is nontrivial, for example in an ionization region, specific modifications of the original Roe solver may be required for proper functionality.

*Choice of the solver* Approximate Riemann solvers may give unphysical solutions. For example, the Roe solver may lead to negative densities. In such places one may switch, for example, to the HLLE solver which is proven to yield positive densities and energies (Einfeldt et al. 1991). At sonic points (where one characteristic speed is 0) the Roe solver may yield unphysical results of a different kind (expansion shocks which violate thermodynamics). It is common use to switch, on such locations, to another solver, perhaps of the HLL-family. The original HLL solver leads to large artificial viscosity so that its general use may not be advisable. We, however, again emphasize once more the great flexibility of HLL schemes in terms of waves included and refer, in addition to the HLLE solver already mentioned, to Linde’s solver (Linde 2001), now termed HLLL.

#### Classic higher order schemes using Riemann solvers

Here we discuss a few methods using Riemann solvers which by now can be considered classic and highly developed. Their aim is to remove the basic drawback of Godunov’s method, namely its limitation to first order accuracy in space and consequently low resolution per gridpoint plus unwanted high numerical diffusivity. Basically, the reconstruction step in these methods is designed resorting to higher order polynomial functions. These methods or likes of them are frequently at the roots of modern codes in our area. A few more recent methods which basically can be subsumed in this category will, however, be discussed separately later on.

Importantly, these methods are *nonlinear* even for linear conservation laws, e.g., $\partial _tv+\partial _x(a(x,t)v(x,t))=0$ for some prescribed function *a*(*x*, *t*). So, for example, Godunov has shown in his basic paper (1959) that, for the advection equation, a monotone solution will stay monotone in general only for *linear* discretizations accurate to *first order* in space. From this follows the necessity of developing such intrinsically nonlinear schemes which indeed can be constructed so as to yield high accuracy and a substantial degree of immunity against artificial oscillations and the like. Many of them are of the RSA type.

*Reconstruction with piecewise linear functions* The first higher order scheme of that type has been MUSCL (van Leer 1997 and the previous papers in that series which present a number of variants and many general ideas). A similar method developed at the same time by Kolgan (1972), see also Kolgan (2011), seems to have been but little known in the Western countries for a long time.

Concentrating here on typical *spatial* discretization we set out with a *reconstruction step* (given grid size averages $\bar{v}_i$ for one component of the conservation law) yielding piecewise linear functions $v_i(x), (x_{i-1/2}\le x \le x_{i+1/2})$. We write them in the form

73 $$\begin{aligned} v_i(x)=\bar{v}_i+\sigma _i(x-x_i) \hbox { for } (x_{i-1/2}\le x \le x_{i+1/2}). \end{aligned}$$

One is free to choose $\sigma _i$ in terms of the conservative property, because $v_i(x)$ yields $\bar{v}_i$ when averaged over the interval independently of $\sigma _i$. $\sigma _i=0$ results in Godunov’s approach.

**Fig. 11:**
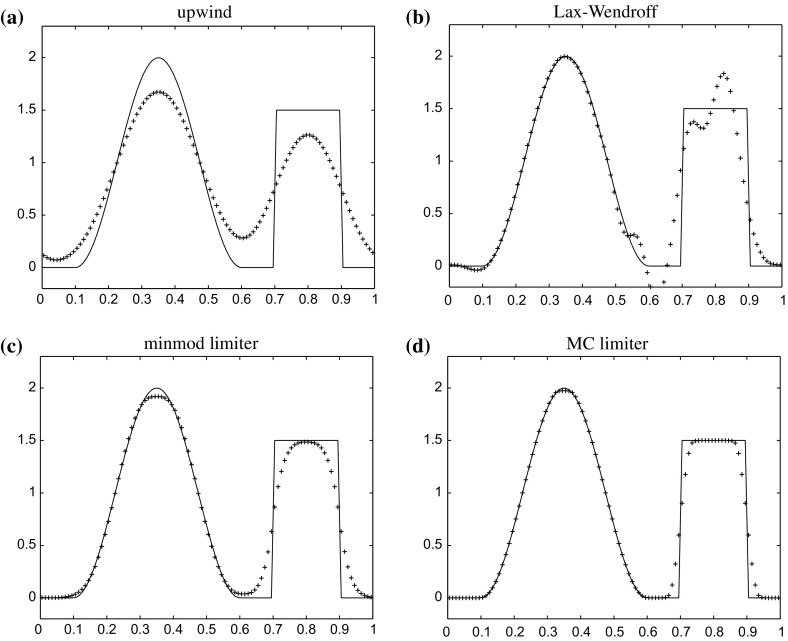
Solution of the advection equation. An initial profile (*solid line*) is moved with constant speed for one revolution (periodic boundary conditions) using various numerical schemes. Upwind (Godunov) is extremely smoothing the solution. Lax–Wendroff performs well in the smooth part but fails on discontinuities. The minmod and the monotonized central differences (MC) limiters perform reasonably or well in the smooth part and work fairly acceptably near jumps. Image reproduced with permission from LeVeque (1998), copyright by Springer

For the advection equation, Eq. (65), with positive speed $\alpha$ the symmetric choice $\sigma _i=\frac{\bar{v}_{i+1}-\bar{v}_{i-1}}{2h}$ leads to Fromm’s method, the upwind choice $\sigma _i=\frac{\bar{v}_{i+1}-\bar{v}_{i}}{h}$ to the Beam–Warming scheme and the downwind choice $\sigma _i=\frac{\bar{v}_{i}-\bar{v}_{i-1}}{h}$ to the Lax–Wendroff scheme. The latter one is particularly interesting because it leads to a 3-point stencil whereas the others need 4 points. It can be generalized to systems in several ways.

The issue is that these methods lead to much better results than Godunov in smooth parts of the flow but unacceptable results in case of discontinuous solutions, in particular unphysical oscillations. Both of these effects are clearly visible in the first column of Fig. 11. This can to a very considerable part be remedied by changes in the slope so that, however, the order of approximation is kept. For example one might apply the minmod function on the slopes. It returns the smallest number by modulus or zero, *viz*.

74 $$\begin{aligned} {{\mathrm{minmod}}}(a,b) = {\left\{ \begin{array}{ll} b &{}\quad |a|\ge |b| \\ a &{}\quad |a|\le |b| \\ \end{array}\right. } \end{aligned}$$

in case *a* and *b* have the same sign and

75 $$\begin{aligned} {{\mathrm{minmod}}}(a,b) = 0 \end{aligned}$$

if they have opposite sign, and similarly for more arguments.

A different variant for choosing the slope actually used is

76 $$\begin{aligned} \sigma _i={{\mathrm{minmod}}}\left( \sigma ^\mathrm{Fromm},2\sigma ^\mathrm{BeamW}, 2\sigma ^\mathrm{LaxW}\right) . \end{aligned}$$

This leads to the monotoniced central difference (MC) method. The factor 2 in two places within the minmod function is intentional. See LeVeque (1998). We take our Fig. 11 from that source. It shows the effects of various schemes for an advection problem. The improvement in dealing with the discontinuity using limited slopes is readily visible in the second row of that figure.

*Piecewise quadratic reconstruction (PPM, Piecewise Parabolic Method)* Moving to higher order interpolation, the PPM method (Piecewise Parabolic Method) devised by Colella and Woodward (1984) has found wide popularity and is being used in a number of codes in astrophysical fluid dynamics. As evident already from its designation, it uses piecewise quadratic instead of piecewise linear functions on each interval. The basic interpolation process sets out by a piecewise linear approximation similar to what has been considered above. This approximation is constructed in such a way as to maintain monotonicity if such should occur in the data (average) values. At extrema, the slope is set to zero. Special criteria are applied in order to figure out whether the zone under consideration contains a discontinuity. In that case, the parabola is steepened and adjacent zones are treated in specific ways in order to avoid over- and undershooting. These processes do not change the zone averages. Various ways to achieve that are in use as described, e.g., in Colella and Woodward (1984) or Fryxell et al. (2000). Various implementations use modifications of the original interpolation process for improving properties near steep gradients or of other aspects.

The piecewise parabolic functions are in general not continuous across cell boundaries. As a consequence, solutions of Riemann problems are a part of the PPM methodology. Since the functions involved are not constant on both sides of the jump, considerations are necessary how to define reasonable left and right constant states for which the Riemann problem then may be solved, keeping accuracy considerations in mind. This can be done using a sort of Lagrangian hydrodynamics for that purpose as in Colella and Woodward (1984) or an Eulerian approach, e.g., Fryxell et al. (2000). As already emerges from these remarks, the PPM method is not solely on a specific sort of spatial reconstruction at a *given* time level but fluxes may in the sense of a predictor step be defined at step $\frac{1}{2}$ when moving from time step 0 to step 1. This serves to make the approximation scheme second order in time. In space, it has many third order features. Special reconstruction processes near discontinuities or extrema (where the usual monotonicity constraints naturally do not apply) will reduce the formal order of spatial accuracy. That holds also true for Strang type splitting applied in multidimensions (see Sect. 5.5). Overall, PPM methodology has found applications in many variants from use in low Mach number flows, e.g., Nonaka et al. (2010), to relativistic astrophysics, see Martı and Müller (1996), or Mignone et al. (2005).

#### ENO (essentially non-oscillatory) schemes

The ENO class of methods has originally been developed within the context of hyperbolic conservation laws, in particular the Euler equations. See Harten et al. (1987) for an early paper. A part of the methodology is concerned with a specific sort of essentially non-oscillatory interpolation. It is by now used in a wide variety of contexts. Specific considerations pertaining to hydrodynamics have then to be added.

***ENO-type interpolation*** Consider a standard 1D interpolation problem, for simplicity on equidistant nodes $x_j=jh$, with function values $w_j$. Consider the simple case that for some index *i*, $w_i=w_{i-1}=\cdots =0$, but $w_{i+1}=1$ so that there is a jump in the data. Wishing to interpolate near position $x_i$ to second order, using a parabola, we might adopt the stencil $S_i=\{x_{i-1},x_{i},x_{i+1}\}$. To the left of $x_i$ the corresponding parabola will yield obviously artificial negative values, a (negative) overshoot. In particular, if such a procedure is applied repeatedly, it can cause oscillations and eventually render the calculations futile. The stencil $S_{i+1}$, starting at $x_i$, would not produce better results either. On the other hand, the stencil $S_{i-1}=\{ x_{i-2},\ldots \}$ would perform well, perfectly indeed in the specific case.

The smoothness of a polynomial can be measured by the magnitude of its highest nontrivial derivative, and for an interpolation polynomial the corresponding divided differences are, up to a factor, just those derivatives (Stoer and Bulirsch 2002). As is well known, especially the higher divided differences of tabular values are particularly large at positions of a jump in the data.

From this it follows that choosing the interpolating polynomial should not proceed with a fixed stencil. Rather, in the above example, the three-point stencil kept could be augmented by one additional gridpoint to the left or the right, forming two new stencils. For these new candidate stencils a smoothness measurement (e.g., using third order divided differences this time) can be applied and the smoothest one being adopted together with its third order interpolating polynomial. The procedure can be extended to higher order interpolation in an obvious way, and this constitutes the *classical ENO interpolation principle*. If one proceeds until to a stencil with $r+1$ points, an *r*th order polynomial used for interpolation will ensue. If, in case a jump exists at all, there are $r+1$ well-behaved points at least on *one* side of this jump, it will yield an accurate approximation, the truncation error being $O(h^{r+1})$.

Let us return to the parabolas above belonging to the stencils $S_{i-2}, S_{i-1}$ and $S_i$. Let us denote these functions by $p_{i-2}, p_{i-1}$ and $p_i$. We might wish to obtain a better approximation for, say, $w(x_{i-1/2})$ (the function underlying our tabular data). If the position in the interval ($x_{i-1/2}$ in our case) is specified, it can be shown that there exist positive weights $\omega _1^*, \omega _2^*, \omega _3^*$, independent of the data $w_j$, such that $\sum _j\omega _j^*=1$ and

$$\begin{aligned} \omega _1^*p_{i-2}(x_{i-1/2})+ \omega _2^*p_{i-1}(x_{i-1/2})+ \omega _3^*p_{i}(x_{i-1/2})=w(x_{i-1/2}) + O(h^{5}). \end{aligned}$$

This process can be expanded to higher order, so that when basically using stencils with $r+1$ nodes each we obtain a truncation error of $O(h^{2r+1})$ instead of $O(h^{r+1}).$

In itself, this procedure would run again into the original difficulties in case of jumps. In the *weighted ENO approach* (Liu et al. 1994; Jiang and Shu 1996) one replaces the “ideal” weights $\omega _1^*$ etc. by weights $\omega _1$ etc. depending on the smoothness of the corresponding polynomial. With these adaptive weights full order of approximation is obtained, truncation error $O(h^{2r+1})$, if the underlying data are sufficiently smooth, whereas the approximation error decreases to $O(h^{r+1})$ if a jump is present (or less if lack of smoothness is more severe so that no high order approximation is possible).

*Reconstruction* $\mathcal {R}$, *averaging* $\mathcal {A}$ In a finite volume scheme, the basic dependent variables appear as cell averages $\bar{v}_i$, defined at (integer) gridpoints. Yet, for fluxes point values are needed at half-integer gridpoints. A reconstruction procedure yielding them is consequently needed. This can be accomplished by a discrete variant of differentiating the integral of a function as follows. The (sort of discrete) indefinite integral of $v, V_{i+\frac{1}{2}}:=\sum _{j\le i}\bar{v}_i$, is defined on half-integer gridpoints. An interpolation polynomial (in the sense of ENO interpolation, hence avoiding steep gradients as best as possible) for *V* can be constructed and differentiated, yielding the reconstructed point value. In that sense,

77 $$\begin{aligned} v_{i+1/2}=\mathcal {R}(\bar{v})_{i+1/2}. \end{aligned}$$

Note, however, that actually *two* values of $v_{i+1/2}$ will be obtained, one left-sided, starting with the interval $(x_{i-\frac{1}{2}},x_{i+\frac{1}{2}})$, one right sided, starting with $(x_{i+\frac{1}{2}},x_{i+\frac{3}{2}})$. In hydrodynamics, when calculating flux functions using the reconstructed values, this leads then to considering a Riemann problem. (Note that upwinding which would lead to just *one* point value is not appropriate here. Basically, the present reconstruction task has nothing to do with conservation laws and directions of signals, and even if it had, in hydrodynamics *v* will be a mix of signals arriving from both sides unless the flow is supersonic.)

The averaging process $\mathcal {A}$ (in the sense of approximating $\int _{x_{i-1/2}}^{x_{i+1/2}}v(x)\,\mathrm{d}x$) is simple: use the average of the ENO interpolating polynomial *p* in the relevant interval for the $v_j$-values:

78 $$\begin{aligned} \bar{v}_i=\mathcal {A}(v)_i:=\frac{1}{h}\int _{x_{i-1/2}}^{x_{i+1/2}}p(x)\,\mathrm{d}x. \end{aligned}$$

*ENO for hydrodynamics: the finite volume scheme* Using the above notation and a representation similar to Grimm-Strele et al. (2014), one Euler forward step (or rather one Runge–Kutta stage) starting from the $\bar{v}$-values with time increment $\tau$ for $\partial _tv+\partial _xf(v)=0$ takes the form (possibly including a Riemann solver in step 3)

1. The $\bar{v}_i$ are given
2. $$v_{i\pm 1/2}=(\mathcal {R}{\bar{v}})_{i\pm 1/2}$$
3. $$\hat{f}_{i\pm 1/2}=f(v_{i\pm 1/2})$$
4. $$\bar{v}_i^{new}=\bar{v}_i -\frac{\tau }{h}(\hat{f}_{i+ 1/2}- \hat{f}_{i- 1/2}) $$

*The Shu–Osher finite difference scheme: 1D case* This form has been derived and worked out by Shu and Osher (1988, 1989). Contrary to conservation forms with which we have exclusively dealt with up to now it is a finite difference form and acts on point values $v_i$. Even so, it has the appearance of a conservation form in semidiscretization, namely

79 $$\begin{aligned} \partial _tv_i=-\frac{1}{h}(\tilde{{f}}_{i+1/2}-\tilde{{f}}_{i-1/2}) \end{aligned}$$

with an appropriate flux like function $\tilde{{f}}$.

As we will see, there is no obvious advantage for the 1D-case over the finite volume method described above. The method comes into full effect in multidimensions as we will explain shortly. Merriman (2003) has shown that by proper consideration the derivation of the basic form can be very simple.

Following him, we set out from a 1D conservation equation

80 $$\begin{aligned} \partial _tv+\partial _xf(v)=0. \end{aligned}$$

Applying the averaging operator defined above we obtain

81 $$\begin{aligned} \partial _t\mathcal {A}v+\frac{\varDelta f}{h}=0, \end{aligned}$$

where $(\varDelta f)(x) = f(x+\frac{h}{2})-f(x-\frac{h}{2})$. The interchange of $\mathcal {A}$ and $\partial _x$ and $\partial _t$ is permitted *since the grid spacing* *h* *is constant* in space and time. We apply $\mathcal {A}^{-1}$ to Eq. (81) and use^8^ that $\mathcal {A}^{-1}\varDelta =\varDelta \mathcal {A}^{-1}$ to obtain

82 $$\begin{aligned} \partial _tv+\frac{1}{h}(\varDelta \mathcal {A}^{-1}f), \end{aligned}$$

so that in the notation of Eq. (79) we have

83 $$\begin{aligned} \tilde{f}=\mathcal {A}^{-1}f. \end{aligned}$$

The algorithm for the Shu–Osher form in 1D hence is similar to the one described above save for exchanges in averaging and reconstruction:

1. The $v_i$ are given
2. $$\bar{f}_i:=\mathcal {A}(f)_i$$
3. $$\tilde{f}_{i\pm 1/2}=\mathcal {R}(\bar{f})_{i}$$
4. $${v}_i^{new}={v}_i -\frac{\tau }{h}(\tilde{f}_{i+ 1/2}- \tilde{f}_{i- 1/2}) $$

*The Shu–Osher finite difference scheme: the multidimensional case* For explanation it suffices to treat the 2D case. For simplicity of notation, we assume equal (not necessary) and constant grid spacing *h* in both directions. We then have 1D averages, e.g., along the *x*-axis, $\mathcal {A}_x(v)_i(y)=\frac{1}{h}\int _{x_{i-1/2}}^{x_{i+1/2}}v(\xi ,y)d\xi$. $\mathcal {A}_y$ is defined similarly, and

84 $$\begin{aligned} \mathcal {A}:=\mathcal {A}_x\mathcal {A}_y=\mathcal {A}_y\mathcal {A}_x \end{aligned}$$

returns the average over a square of size $h\times h$. The meanings of $\mathcal {R}_x, \mathcal {R}_y$ and $\mathcal {R}$ (the last one being the 2D reconstruction) should be obvious. We have $\mathcal {R}=\mathcal {R}_x\mathcal {R}_y=\mathcal {R}_y\mathcal {R}_x$, so that all averaging and reconstruction procedures are ultimately of the 1D type. Applying $\mathcal {A}$ and $\mathcal {R}$ to the equation $\partial _tv+\partial _xf(v)+\partial _yg(v)=0$ yields algorithms looking precisely as those describe above (with a *g*-term added, of course).

The essential advantage with the *finite difference* variant concerns the accuracy of the boundary fluxes and hence the method. The fluxes are exact to the order the basic procedures $\mathcal {A}, \mathcal {R}$ are exact. For example, the flux at the left vertical side of a grid cell should accurately approximate an integral over the *y*-direction. In a normal finite volume method one only gets a point value with respect to *y*, probably for $y_j$, i.e., one applies in effect the midpoint rule when averaging the flux over the interval $(y_{j-1/2},y_{j+1/2})$ which is only second order accurate. So, the accuracy is compromised unless one is ready to use a few quadrature nodes in this interval in order to increase the degree of accuracy. Naturally that comes at a substantial increase in computing time. Contrarily, for the Shu–Osher form this is built in due to the application of $\mathcal {A}_y$ such that the accuracy is of the order of the basic averaging and reconstruction procedure.

As a concluding remark to the Shu–Osher form let us note that it is not only valid for a constantly spaced grid as discussed above. Merriman (2003) has shown the existence of precisely one class of non-equidistant grids for which the conclusions also hold true. Similarly to logarithmic grids that allows for stretched grids. In the astrophysical context this has already been applied, for example, when modelling pulsation–convection interactions (e.g., Mundprecht et al. 2015) where enormously different scales prevail at the surface of the star and deeper down.

*ENO methods for systems of equations* The treatment of hyperbolic systems of equations with ENO methodology (for the 1D case still) is in most cases reduced to the case of scalar equations.

We start with the hyperbolic system

85 $$\begin{aligned} \partial _t\varvec{v}+ \partial _x\varvec{f}(\varvec{v})=[\partial _t\varvec{v}+ A\partial _x\varvec{v}]=0 \end{aligned}$$

where $A(\varvec{v})$ is again the Jacobian

86 $$\begin{aligned} A(\varvec{v})=(\partial _{\varvec{v}}\varvec{f}(\varvec{v})). \end{aligned}$$

Since the $n\times n$-system of equations is assumed hyperbolic, there exist eigenvalues $\alpha _1,\ldots ,\alpha _n$ and a corresponding complete set of eigenvectors $\varvec{r}^j$ which we assemble in a matrix $R:=(\varvec{r}^1,\ldots ,\varvec{r}^n)$. In case of the Euler equations the eigenvalues are velocity *u* and $u\pm c, c$ denoting the sound velocity.

We define $\varvec{w}$ via $\varvec{v}=R\varvec{w}$. If we assume for one moment that *A* is constant and use $\varvec{w}$ rather than $\varvec{v}$ in Eq. (85) (multiplying from the left with $R^{-1}$) we get

87 $$\begin{aligned} \partial _t\varvec{w}+ D \partial _x \varvec{w}= 0, \end{aligned}$$

where $D=R^{-1}AR$ is the diagonal matrix containing the eigenvalues $\alpha _i$. Hence, the equations decouple and read

88 $$\begin{aligned} \partial _t \varvec{w}_i + \alpha _i \partial _x\varvec{w}_i = 0 \quad (i=1,\ldots ,n). \end{aligned}$$

In general, however, *A* will not be constant. We wish to apply essentially the same procedure in this case and focus on a cell around some grid point $\xi$ at time *t*. In order to proceed near that point, we apply one and the same matrix of eigenvectors *R* for $A(\varvec{v}(\xi ,t))$ in the whole vicinity $\xi$ where ENO leads us to and transform Eq. (85) to the form

89 $$\begin{aligned} \partial _t\varvec{w}+\partial _x\bigl (R^{-1}\varvec{f}\bigr )({\varvec{w}}) = 0, \end{aligned}$$

i.e., a conservation law for $\varvec{w}$ with flux function $\varvec{f}^*=R^{-1}\varvec{f}$. The ENO procedure for the stencils which appear when starting from $\xi$ is carried out using that equation, and finally the results are transformed back to $\varvec{v}$.

Of course, in the general case decoupling of equations will not be complete as it is in Eq. (87). Setting that point aside for one moment we see that this grants the possibility to use the proper upwind direction for each “field” $w_i$.

*Remarks on the numerical flux functions* A few remarks concerning numerical flux functions are in order. Consider firstly that at a cell boundary $x_{i+1/2}$ there exist two values for *v*, namely $v^-_{i+1/2}$ and $v^+_{i+1/2}$ corresponding to reconstructions belonging to the cell left or right of $x_{i+1/2}$, respectively. One consequently faces a sort of Riemann problem. Frequently, a Roe-type flux is used with a switch to a Lax–Friedrichs flux near a sonic point to avoid the physical inconsistencies we have mentioned in connection with the Roe approximate Riemann solver.

A second point concerns what is now called Marquina flux splitting (Marquina 1994; Donat and Marquina 1996). The basic issue is that when evaluating the Jacobian of the flux function at a half-integer grid point which is located near a strong jump no sort of average of the dependent variables may lead to a proper version of the Jacobian. The limits when moving to $x_{i+1/2}$ from the left or from the right may be markedly different. In the Marquina approach two Jacobians are used at those locations, corresponding to two ENO procedures based on bias to the left and to the right. Those fluxes are then kept which lead from one side to the other. A closer description in conjunction with the ENO approach is provided in Fedkiw et al. (1996).

#### Some other higher order methods

Recently, a number of other numerical approaches have started to be used in codes which are relevant to our context. They basically work with control cells (simplices, cubes or more general hexahedral regions, etc.) and consider the interaction of the cells by evaluating the flux across the cell interfaces. Typically, the solutions of these methods are discontinuous over cell interfaces so that a sort of Riemann solver is being applied for the calculation of fluxes.

In Colella et al. (2011) and Wongwathanarat et al. (2016) fourth order for cell averages, for example, is achieved by using Taylor expansion keeping the second derivative, which, in itself, needs to be calculated to second order only for sufficient accuracy. In particular, the method is considered as a basis for the mapped grids technique as described in Sect. 5.4.

Schaal et al. (2015) and Wang et al. (2015) describe high-order discontinuous Galerkin and high-order spectral difference methodologies with a special view towards application in the case of “irregular” domains (most probably spheres or spherical shells). One very important point in particular for long-term runs as required for relaxation or dynamo action is that these methods are also capable of preserving not only linear but also angular momentum which is unlike what most numerical methods can accomplish.

#### Spectral methods

If the solutions of the equations are represented as linear combinations of basis functions at all, these basis functions have been locally used polynomials in most of the methods treated above (including the version of the spectral difference method as applied in Wang et al. (2015) just mentioned above which is termed “spectral” nonetheless for somewhat different terminological reasons). Spectral methods which we consider now instead represent the spatial part of the functions via *global* basis functions, i.e., functions being $\ne 0$ in all of the spatial domain (generically). For a thorough presentation of spectral methods see Canuto et al. (2006).

The best known case is of course the trigonometric approximation

90 $$\begin{aligned} u_N(x)=\sum _{k=-N}^N \tilde{u}_k\phi _k(x) \end{aligned}$$

with

91 $$\begin{aligned} \phi _k(x)=e^{ikx} \quad (x\in [-\pi ,\pi )) \end{aligned}$$

in the complex notation. (In practical calculations, sines and cosines are used instead.) In multidimensional calculations, tensor products of 1D basis functions may be used, akin to $\phi _k(x)\psi _l(y)$.

For our class of problems, there are two main possible advantages when using spectral methods, namely

- dealing with sphericity (problem specific)
- possibly rapid convergence to the solution with increasing *N*.

With respect to the first item, spectral methods are indeed often used when dealing with *spherical shells*. The difficulty with many grid based methods is the convergence of longitude circles towards the polar axes (see the discussion in Sect. 5.4). It is then tempting to expand the angular part of functions in spherical harmonics,

92 $$\begin{aligned} Y_{l}^m(\theta ,\psi )=c_{lm}P_{l}^m(\cos \theta )e^{im\psi }=c_{lm}P_{l}^m(\cos \theta )\phi _m(\psi ) \end{aligned}$$

where $\theta$ is the polar distance, $\psi$ the longitude, $\phi _m$ from Eq. (91) and $c_{lm}$ an appropriate normalization constant. $P_{l}^m$ denote the associated Legendre polynomials. In that way, sphericity is automatically dealt with.

Before discussing the more general pros and cons, let us have a look at the gist of spectral methods in a simple 1D case using trigonometric functions just introduced around Eq. (91). In applications, one can

1. make use of simplicity of differentiation, viz. $\partial _x\phi _k(x)=ik\phi _k(x)$,
2. the orthogonality of the trigonometric functions, viz.
  93 $$\begin{aligned} \langle \phi _k\phi _l\rangle :=\frac{1}{2\pi }\int _{-\pi }^\pi \phi _k(x)\bar{\phi _l}(x)=0 \hbox { for} \quad k\ne l\hbox { and} \end{aligned}$$
3. the fact that products of the $\phi _k$’s are simple: $\phi _k\phi _l=\phi _{k+l}$.

We apply such expansions to Burger’s equation,

94 $$\begin{aligned} \partial _tu + \frac{1}{2}\partial _x\frac{u^2}{2}=0. \end{aligned}$$

The nonlinearity due to $u^2$ is similar to nonlinearities occurring in the Euler equations, Eqs. (42a)–(42c), whence our interest. Ideally the left hand side should be 0 or, equivalently, orthogonal to *all* functions $\phi _k$. The *Galerkin approximation* stipulates that it be orthogonal to all $\phi _k$ ($-N\le k \le N$) for some prescribed natural number *N*. Inserting functions of the form

95 $$\begin{aligned} u(x,t)=\sum _{l=-N}^N\tilde{u}(t)\phi _l(x) \end{aligned}$$

into the left hand side of Eq. (94), using the properties listed above and finally requesting the inner products with the functions $\phi _k$ ($-N\le k \le N)$ to vanish, we obtain

96 $$\begin{aligned} \dot{\tilde{u}}_k(t) +i\sum _{l=-N}^Nl\tilde{u}_l(t)\tilde{u}_{k-l}(t)=0 \quad (-N\le k\le N). \end{aligned}$$

Note that for some values of *l* in the second sum $k-l$ will fall outside of the range of indices considered here. For numerical work, they will have to be dropped.

One advantage of the Fourier–Galerkin formulation is in the approximation error. Methods based on polynomials of order *n* can achieve (in a large class of problems and *provided the solution is sufficiently smooth*) an approximation error of $O(h^{n+1})$ and with guarantee not better (in the generic case). In contrast, for a large class of problems (*and solutions being differentiable infinitely often*) approximation error for the Fourier–Galerkin formulation can be shown to be $O(h^{n+1})$ for *each *natural number *n*. In practice, convergence is very rapid once a feature is resolved with a specific number of basis functions. On the other hand, if there is a discontinuity or, in practice, a steep gradient somewhere convergence will be degraded not only locally, but globally.

A disadvantage of the Fourier–Galerkin method is its numerical complexity. The second sum in Eq. (96) requests *O*(*N*) multiplications for each value of *k*, hence overall $O(N^2)$ operations at each timestep which is unfavourable for all values of *N* which are not quite small.

In practice, therefore, a variant of the above procedure, the *collocation method* is used more often. Here, one switches between *u*, defined spatially on $2N-1$ equidistant gridpoints and the Fourier picture (the coefficients $\tilde{u}_k$). In the example, $u^2$ is computed in real space, on the gridpoints (*O*(*N*) operations!); the resulting discrete function is transformed to its Fourier image via the fast Fourier transform (complexity $O(N\log N)$, much better than $O(N^2)$). Conversely, also the transition from the Fourier picture to real space is needed which has the same computational complexity. Note, however, that on massively parallel machines that issue is not trivial due to completely different ordering principles (spatial ordering in real space, ordering by Fourier index *k* in Fourier space). It is not said that collocation methods have the same accuracy as comparable Galerkin methods. In addition, Galerkin methods do generically better to retain conservation properties than collocation, albeit for special forms of time integration only (Canuto et al. 2006).

Furthermore, things get more complicated when using, for example, the Legendre polynomials (as in the spherical harmonics) because they lack the beautiful properties of the trigonometric functions so that fast transforms do not exist (see, e.g., Clune et al. 1999).

The use of spherical harmonics is, however, greatly facilitated if the main task is to solve a Poisson equation (as often occurs in the anelastic approximation) because the spherical harmonics are eigenfunctions of the Laplace operator.

For a detailed discussion we again refer the reader to Canuto et al. (2006).

### Direct low Mach number modelling

Direct low Mach number modelling refers to the numerical solution of the unaltered Euler (or Navier–Stokes) equations with very slow flows in mind. In Sect. 4 a number of approaches were introduced aimed at modelling deep, low Mach number convection. These approaches modified the basic equations. In the last few years developments have been initiated to achieve the same goal more directly. Instead of an approximation being performed in two steps (approximate equations are derived firstly and these are then approximated in the numerical sense) the strategy is now to set out from the original Euler or Navier–Stokes equations and to develop numerical methods which in themselves are not subject to the stringent Courant–Friedrichs–Lewy timestep restrictions. In this subsection we present three such approaches. Since in the low Mach case the problem consists in the high velocity of sound enforcing unduly small timesteps for the usual numerical methods, it is the Euler equations which are under consideration here.

#### The method of Kwatra et al.

Kwatra et al. (2009) have devised a method which largely keeps the original approach for numerically advancing the Euler equations in time (for example, by an ENO method). Basically, the flux function is decomposed into two parts, one being the advective part and the second one the non-advective part (i.e., the one which contains the pressure *p*). The advective part is time-advanced in the usual way yielding, among others, preliminary versions of the conserved variables. An evolution equation for the pressure is used to predict it at the new time level. After various considerations, the ultimate fluxes are computed and applied in the usual way. From the viewpoint of computational complexity the only new task is the requirement to solve a generalized Poisson (or, more precisely, Helmholtz) equation for the pressure. Solving such a so-called strongly elliptic or *V*-elliptic equation is a standard problem in numerical mathematics and can be accomplished very efficiently, for instance, by means of multigrid methods even in a highly parallelized way. The region of applicability of this approach has been enhanced among others by introducing two species (such as hydrogen and helium in semiconvection) in Happenhofer et al. (2013).

We note in passing that it is possible to apply the method also to high Mach number flows with reasonable efficiency, e.g., supersonic ones, without any change. While flows of high speed are of course not the main application of this method, this capability may be helpful for specific problems for which one has to deal with low and high Mach number flows simultaneously (a numerical simulation of solar convection reaching from the surface deep into the interior might be one such example). It should also be noted that, as the sound speed tends to infinity, the generalized Poisson equation approaches that one for an incompressible flow.

One additional advantage of the method is that, once the pressure is split off, the eigenvalues of the remaining hyperbolic system are all equal (to the macroscopic velocity). That virtually eliminates the need for transformation to the eigensystem and makes the Riemann problem easier (Happenhofer et al. 2013).

#### A preconditioned Godunov-type method

In Sect. 5.2 we have discussed a number of methods which can be considered a successor of Godunov’s method. Such methods can be turned into efficient algorithms for the low Mach case as worked out by Miczek et al. (2015). Two basic requirements for a low Mach number solver have been formulated by Dellacherie (2010). Taking care of these and of principles of preconditioning (see Turkel 1999, for a review) they analyze the Roe matrix and devise a Roe solver which yields numerical fluxes. These fluxes allow time steps of the CFL restrictions based on material velocities also for very low Mach number flows. At the same time, the method is applicable to the case of “normal” Mach numbers, too.

#### Solving the Euler equations implicitly

Viallet et al. (2011) are developing the MUSIC code (see Sect. 5.6.16). The goal is to model essentially whole stars or stellar envelopes, respectively, in 2D and in 3D with emphasis on situations requiring low Mach number modelling. With this in mind, an implicit method for the Euler (and Navier–Stokes) equations in multidimensions has been developed. Very recently, a semi-implicit module has been described (Viallet et al. 2016). It treats advective terms explicitly whereas sound waves or compressional work are treated implicitly. This module aims for stability under large time steps rather than for accuracy and is intended for eventual use as a preconditioner to the fully implicit basic procedure.

### Sphericity and resolution

The “classic” procedure when dealing with sphericity consists in developing the lateral part of the dependent variables in spherical harmonics as, for example, done in the Glatzmaier code (Sect. 5.6.11) and its descendants. In this way one problem of the use of polar coordinates (singularities of the equations along the polar axis) is obviated, the other one (singularity at the center) retained. For numerical aspects of this spectral approach see Sect. 5.2.8. Consider in particular that for very large problems (high order harmonics involved) a problem may be rooted in the Legendre function (Clune et al. 1999) for which no fast algorithm akin to fast Fourier transform for the trigonometric functions exists.

Sticking to polar coordinates has nevertheless some appeal, in particular when only a part of a spherical shell is modelled. Then, the polar axis problem has no bearing. The relative similarity to straight rectangular grids allows much of the basic numerics described in Sect. 5.2 to be taken over.

Even for a whole spherical shell, polar coordinates are applicable by using so called Yin–Yang grids introduced by Kageyama and Sato (2004). This approach makes use of the equatorial belts of two spherical grids with polar axes tilted against each other in such a way that, together, the whole sphere or spherical shell is covered. The equations can then, on each grid, be advanced for example with the existing scheme designed for polar coordinates. A problem is that in the overlapping regions the boundaries of the grid cells will not match. Hence, the conservative property of the code will be lost unless one resorts to a flux correction procedure on the boundaries between the parts of the two grids which are actually in use (Peng et al. 2006; Wongwathanarat et al. 2010).

Given that methods on rectangular (cubic) grids are widespread and *ceteris paribus* usually the best ones it is tempting to stick to them even in the spherical case. Putting one’s *star-in-a-box* has immediate appeal and is provided, for example, in the CO5BOLD code (Sect. 5.6.7). Perhaps the most important drawback of this method is that resolution may not be granted where desperately needed (for example, in the upper layers of Cepheids, RR Lyr stars or red giants) unless an elaborate grid refinement algorithm is available.

More recently, mapping techniques have found interest. Here, the stellar sphere (say) is the image of a cube under a map. The numerical integrations are performed for the equations transformed to the cube. An essential point to be considered in this case is the so-called free stream property. For a closed surface it is well known that the surface integral over the normal vectors vanishes, $\int d\mathbf {n}=\mathbf {0}$. The free stream property stipulates that the analogous condition holds true for the discrete method used to calculate fluxes over cell boundaries (Wongwathanarat et al. 2016). In Grimm-Strele et al. (2014) WENO methods for such curved coordinates in physical space have been investigated. It turns out that the free stream property does not hold true for the Shu–Osher form of the method so that one must resort to the original finite volume formulation, if higher than second order is requested. In Wang et al. (2015) and Wongwathanarat et al. (2016) issues of just how to properly implement the mapping and its interaction with the basic numerics are discussed thoroughly.

#### Resolution

Section 5.6 clearly testifies how much activity presently is invested into the development of new codes. To a considerable degree this development is driven by the quest for high resolution per gridpoint. This quest is motivated both by the highly turbulent character of the flows with many spatial scales involved and by simple geometrical facts such as the strong variations in scale height when moving from the atmosphere to deeper layers in the Sun and other stars.

Let two examples serve to illustrate the importance of resolution per grid point which naturally is relevant in particular for computationally highly demanding cases where one cannot simply increase the number of grid points.

The first example for the importance of adequate resolution is quite recent and concerns an important issue in *magneto*hydrodynamics. Experience shows that it is easier to obtain dynamo action resulting in a significant part of an *ordered* magnetic field (of interest for an understanding of the solar cycle) in convection simulations with comparatively high viscosity and magnetic diffusivity. Lowering those values (which for the Sun are unattainably small by numerical means) leads, however, to an increase of disordered fields. Yet, use of still smaller numbers for these values and an extreme number of gridpoints and hence resolution (as requested by the small scale structures then developing) lets the *ordered* part of the magnetic field increase again (Hotta et al. 2016), reinforcing prospects for modelling of solar and stellar magnetic cycles.

Just for completeness we notice that important effects of resolution occur also in the purely hydrodynamic case. For example Guerrero et al. (2013) find somewhat surprisingly that one of their low resolution models fits the solar rotation profile more closely than a higher resolved one. Might not still higher resolution lead one again closer to the observations?

The second example concerns the convection–pulsation interaction in Cepheids (Mundprecht et al. 2013). With low order radial pulsation in mind, a large part of the star must be modelled. In contrast, the atmosphere and the hydrogen convection zone which are important observationally and dynamically are quite thin, leading from the outset to extremely disparate spatial scales. As a consequence and in addition, the calculations are very demanding in terms of computational resources, even in 2D. If, in each direction, only half of the grid points which have ultimately been used are employed, the convective flux in the hydrogen ionization zone shrinks to one fourth of its adequate value. Without check (which may be impossible in leading edge calculations) a model which is grossly in error could be easily mistaken for an appropriate one.

The requests for proper and hence often high resolution must be answered by increased performance of computers and by numerics. In numerical respects a part of this answer is given by local grid refinement. This option is available in a number of contemporary codes, see Sect. 5.6. In a sense more basically it is, however, given by the quality of the numerical algorithm and, to a considerable degree, by the order of approximation.

**Fig. 12:**
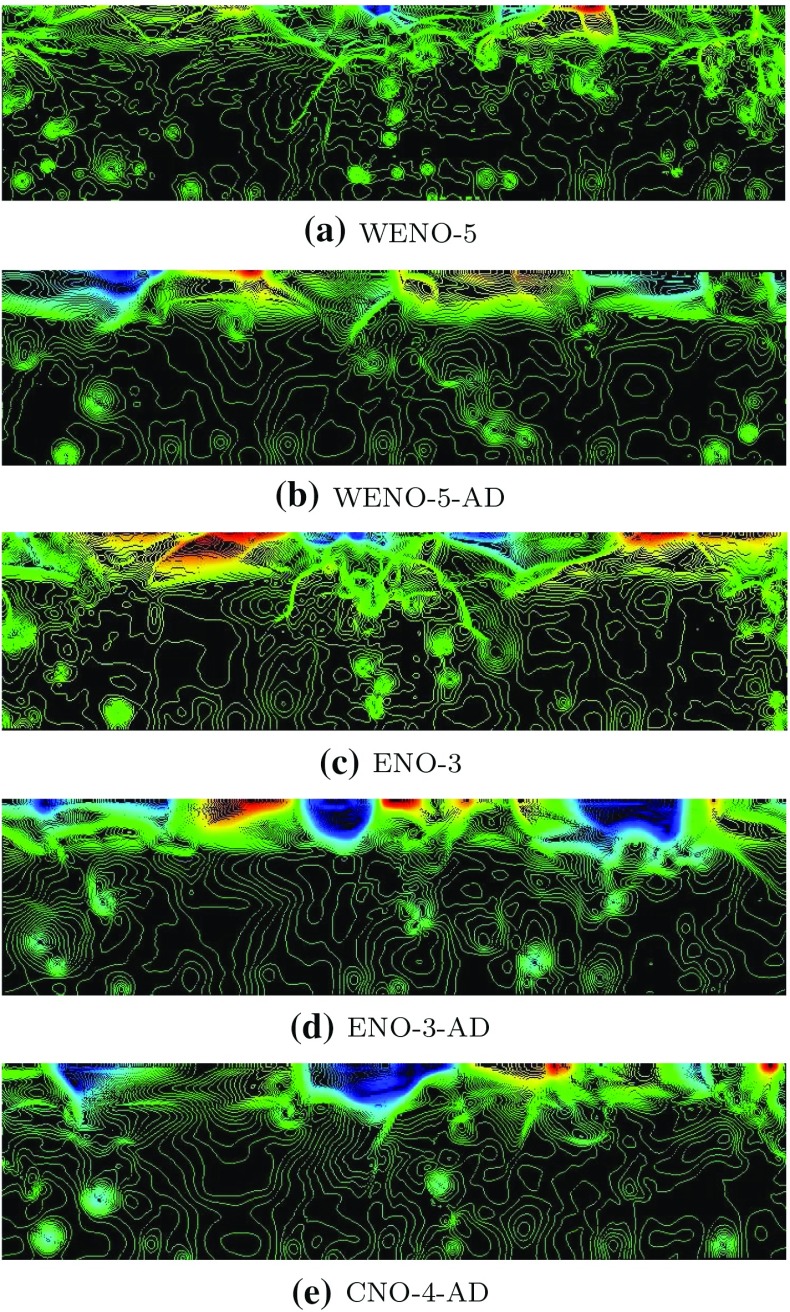
2D simulation of solar surface convection (granulation) with different numerical methods for otherwise identical setups. In particular, the grid spacing is the same in each case. This is illustrated here with plots showing isolines of pressure perturbations computed from the logarithm of pressure with the logarithm of its horizontal average subtracted. All five simulations have the same initial state (25 solar minutes earlier). Note the superiority of the fifth order WENO scheme in comparison with the third order ENO scheme. Resolution is markedly degraded by artificial diffusivities (labelled AD) applied in a manner often used in solar physics where they are needed for some popular numerical methods to maintain stability. Image reproduced with permission from Muthsam et al. (2010), copyright by Elsevier

In this respect, a figure may be quite telling. Figure 13 in Muthsam et al. (2010) which we reproduce here for convenience (Fig. 12) provides a snapshot of solar granulation modelled by various methods, among them a weighted ENO scheme of order 5 and a normal ENO scheme of order 3. The difference in resolution and crispness of the pictures (which is corroborated in runs with a finer grid) is quite appealing. The results are comparable as directly as possible since they are performed by the same software, interpolation methods, etc. (for the meaning of those runs which are labelled AD consult that paper). One reason why higher order methods yield crisper discontinuities may be due to the fact that the jump fed into the Riemann solver is, in general, smaller when higher approximation order is used which, in turn, leads to less numerical diffusion.

Comparison of the above runs shows that two or three times as many gridpoints are needed per direction to resolve a feature for the poorer methods than for weighted ENO 5. Conservatively estimated, that leads in 3D to an 8-fold increase in gridpoint numbers and 16-fold increase in CPU time (because of the smaller time step necessary) for the simpler methods. (The factor in time may be smaller because ENO has to do more computational work; on the other hand, we have cautiously started only with a factor of 2 in 1D.)

Perhaps the best example for early recognition of the value of high resolution per gridpoint in the astrophysical context is the development of the piecewise parabolic method (PPM) by Colella and Woodward (1984). Presently, many of the newer codes presented in Sect. 5.6 embrace this aspect and pay full attention to advanced numerics.

Of course, a detailed comparison of different methods in present use in this area would be valuable. At the same time that would not be easy setting aside the task of having it performed at all. Details of coding, of memory access patterns or even of the hardware originally held in mind may influence the results considering that now even graphics cards commence to be used for hydrodynamical simulations. Furthermore, a measure reflecting both CPU and memory demands would have to be defined which might not be possible unambiguously.

### Time marching

From the viewpoint of time integration, both the Euler and Navier–Stokes equations and also the approximations to them which we have discussed in Sect. 4 are actually quite simple: they just require the computation of an explicit, first order, partial derivative of type $\partial _t f$, as common for evolution equations of dynamical systems. The coupling of spatial and temporal dependencies in special and general relativity complicates this simple relationship and requires additional methods and approximations which we are not dealing with in this review, since we restrict ourselves here to the case of hydrodynamics as part of classical mechanics and classical statistical physics. We briefly review some of the most important concepts and methods used in this context.

#### Method of lines and a note on splitting and partitioning

Although we are dealing with *partial differential equations* (PDEs) when solving (42a)–(42c) coupled to any of (44)–(46), for example, or when solving approximative equations such as (50) and (51), (52), (56), or (59), each of them could be considered to be at first discretized in space only. This *semi-discretization* yields a set of coupled *ordinary differential equations* (ODEs) with a very simple left-hand side $\partial _t f_i$ for a vector $\mathbf {f}$ consisting of components $f_i$ and a complicated right-hand side, which, however, contains no derivatives in *t*. This right-hand side could thus be passed to an ODE solver package that integrates this system in time. That approach is known as the *method of lines* (MOL). Special care in this context has only to be given to the boundary conditions of the PDE. In most cases it is possible to implement them such that the MOL approach can be used. For example, one can exclude the boundary conditions and the associated spatial grid points from time integration and apply them as a constraint after each time step, in case they are stationary. Or one can also integrate them in time as yet another set of ODEs coupled to the other equations which describe the dynamical evolution in the interior of the spatial domain on which the PDEs are to be solved. The advantage of this approach is the huge number of methods and mathematical, analytical tools available this way from the theory of ODEs and their numerical approximation for the case of initial value problems, rather than developing custom-made solvers straight for the PDEs for the full initial-boundary-value problem each time (we demonstrate the benefits from this approach with examples in Sect. 5.5.4).

Historically, a number of basic, “text book methods” for hyperbolic equations (such as Euler’s equations, the wave equation, etc.) and parabolic equations (such as the heat equation and other diffusion equations) have been developed starting directly from the PDE framework. Examples are the leap frog method, the Lax–Friedrichs method, and the Lax–Wendroff method for the hyperbolic case or the Crank–Nicolson method and the closely related $\vartheta$-method for diffusion equations (the latter contains the former as a special case when $\vartheta =1/2$). We refer to Richtmyer and Morton (1967) and Strikwerda (1989) for each of them and for classical derivations. However, especially in the context of generalizing these methods from PDEs depending on one spatial dimension to the multidimensional case, it turned out to be advantageous to view, e.g., the Lax–Friedrichs method and the Lax–Wendroff method as approximations to compute the numerical fluxes of semidiscretized hyperbolic conservation laws, Eq. (60). They are distinguished from the simple, explicit approach, Eq. (61), by introducing *artificial diffusion* (cf. Richtmyer and Morton 1967) without resorting to an upwind principle as in Eq. (66) for Godunov’s method (no considerations with respect to the direction the signal is coming from are made in their cases). This way many classical PDE schemes can also be understood in the framework of ODE MOL solvers, but with a built-in viscosity added in the spatial discretization. Likewise, the leap frog method is just a *multistep method* which is applied to a hyperbolic conservation law (see Sect. 5.5.3 and cf. the analyses in Strikwerda 1989, Richtmyer and Morton 1967) and also many methods for the time integration for diffusion equations can be understood in that framework. Indeed, most time integration methods for PDEs are either MOL solvers applied to (spatially) semi-discretized PDEs or can at least be reinterpreted this way. Due to the mathematical properties of the hydrodynamical equations the ODE methods in use for time integration fall into the category of *general linear methods* (Butcher 1965, for an overview we refer to Hairer et al. 1993, Hairer and Wanner 1996, and to Butcher 2006). Runge–Kutta methods and linear multistep methods are their most important special cases. Due to their importance for the numerical simulation of stellar convection we discuss the role of those two in the context of hydrodynamical codes below.

Before we give an overview on those methods we briefly turn to an approach of time marching which is more difficult to interpret in the MOL framework. In the case of more than one spatial dimension, so-called time split schemes (Chap. 7.2 in Strikwerda 1989, in the literature this approach is also known as *dimensional splitting*) are common to reduce multidimensional problems to problems in one space dimension (Yanenko 1971). Given an additive structure of the spatial operator (such as the Laplacian) one proceeds, for example in the case of two spatial dimensions, with only one spatial component acting at twice the rate over half the time step. Then the second spatial component is dealt with the same way. Thus, each sub-problem solved in each fractional step deals with spatial derivatives acting on just one dimension. In practice, spatial directions have to be interchanged subsequently or even the first step is reiterated to construct methods which are second order in time (see Strang 1968; Gottlieb 1972). This principle can be generalized to perform such a splitting not merely with respect to spatial directions but according to other properties, i.e., considering operators relating to advection and diffusion as the two sub-problems which are dealt with by splitting (with the non-iterated approach known as Lie–Trotter splitting and the reiterated one that ensures second order in time as Strang splitting). Best known among those schemes in computational astrophysics is probably the time split MacCormack scheme (MacCormack 1971). Another, powerful method which also aims at reducing multidimensional problems into problems in one spatial dimension is the alternating direction implicit method (ADI, for an introduction and many literature references see Chap. 7.3 in Strikwerda 1989). The main idea there is that the coupling terms which involve the mixed spatial derivatives are often of higher order when discretized numerically and can hence be dropped. Then again one solves a sequence of problems with derivatives in just one spatial dimension (and possibly interchanging them in subsequent time steps). In numerical simulations of stellar convection this method has been used by Kim and Chan (1998) and later on by Robinson et al. (2003), e.g., to shorten the time it requires to proceed through $t_\mathrm{rel}$, the relaxation time of the problem, with an Euler backward method used for a fully implicit time integration of the fully compressible Navier–Stokes equations. A related “splitting” approach which is used in the context of Runge–Kutta methods for solving PDEs with the MOL concept is to consider the spatial operators such as advection and diffusion as separate operators which are integrated in time during each step with separate methods, for instance, an explicit method for advection and an implicit one for diffusion. We return to these *partitioned Runge–Kutta methods* (Hairer et al. 1993), also known as *additive Runge–Kutta schemes*, below.

#### Runge–Kutta methods

Among the general linear methods for ODEs *Runge–Kutta methods* are distinguished by being *one-step methods*. Thus, no information on the solution at previous time steps is needed to start time integration and achieve full order of accuracy (cf. Hairer et al. 1993). Instead, single Euler forward or Euler backward steps as in (61) and (62), or sometimes also time centred steps are used to obtain an intermediate solution in a first *stage*. The result from this stage may then be used during the construction of further stages. Finally, the available intermediate results from the different stages are used to complete the time step through adding them up weighted such that error cancellation allows reaching higher than first order. An immediate advantage of Runge–Kutta methods is that the time step may easily be changed at each new step. The main downside is the necessity to evaluate the right-hand side of the ODE several times to achieve higher order (e.g., four times for an explicit method to reach fourth order). *For hydrodynamics this is not very important, since in most cases the overall accuracy of the numerical integration is limited by the spatial discretization, especially in the LES case.* Evidently, this always holds for realistic multidimensional simulations of stellar convection (see also Grimm-Strele et al. 2015b). By proper design of the method per se or by rearranging the intermediate results from the different stages it is also possible to avoid having to store them all until the end of each time step. A Runge–Kutta method with *s* stages per time step to solve $\partial _t y=F(y(t))$ is formally written as

97 $$\begin{aligned}&y_i = y_\mathrm{old} + \varDelta t \sum _{j=1}^s a_{i,j} F(y_j) \quad \hbox {for each}\quad i=1,\ldots ,s, \nonumber \\&y_\mathrm{new} = y_\mathrm{old} + \varDelta t \sum _{j=1}^s b_{j} F(y_j), \end{aligned}$$

where $\varDelta t$ is the time step, $y_\mathrm{old}$ and $y_\mathrm{new}$ are numerical approximations at old and new points in time separated by $\varDelta t$, the $y_i$ are intermediate values for each stage within a time step, $A=(a_{i,j})$ is the coefficient matrix of the scheme, and the weights for the final summation of each time step can be collected into a vector $b=(b_1, \ldots , b_s)$. If ${a}_{i,j}=0$ for $j\ge i$, the method is *explicit*, otherwise it is *implicit*. An *s*-stage *partitioned Runge–Kutta method* to solve $\partial _t y=F(y(t))+G(y(t))$ is characterized by coefficient matrices $A=(a_{i,j})$ and ${\tilde{A}} = ({\tilde{a}}_{i,j})$ and stage summation vectors $b=(b_1, \ldots , b_s)$ and $\tilde{b}=(\tilde{b}_1, \ldots , \tilde{b}_s)$ such that

98 $$\begin{aligned} y_i= & {} y_\mathrm{old} + \varDelta t \sum _{j=1}^s a_{i,j} F(y_j) + \varDelta t \sum _{j=1}^s \tilde{a}_{i,j} G(y_j),\quad i=1,\ldots ,s,\nonumber \\ y_\mathrm{new}= & {} y_\mathrm{old} + \varDelta t \sum _{j=1}^s b_{j} F(y_j) + \varDelta t \sum _{j=1}^s \tilde{b}_{j} G(y_j). \end{aligned}$$

If ${a}_{i,j}=0$ for $j\ge i$ whereas $\tilde{a}_{i,j}\ne 0$ for at least one $j\ge i$, the method is referred to as an *implicit–explicit (IMEX)* method. We point out here an important property of Runge–Kutta schemes. Their numerical parameters (coefficients), which determine the discrete time of evolution of the right-hand side of the ODE in each stage and to what extent they are taken into account during the next stage as well as how they are weighted in the final summation concluding each time step, are the result of a *mathematical optimization process*. Thus, an infinite number of Runge–Kutta methods exists and their coefficients are the result of optimizing for

1. fulfilling internal consistency conditions,
2. implicit or explicit nature of the method,
3. order of accuracy,
4. range of stability in the complex plane for the case of linear, constant-coefficient ODEs,
5. minimizing the error constant in the given order of accuracy,
6. and any other stability or other property deemed useful,

once a fixed number of stages for the method has been chosen. Only the first two of these six constraints are absolutely necessary, the others are optional. No “perfect” method exists which is optimal in all respects. *This holds particularly for “text book methods”, which have usually been developed a long time ago and have thus been optimized only for order of accuracy and computational simplicity with the ensuing stability region usually being just a result from the other constraints that had to be fulfilled.* New Runge–Kutta methods are thus being developed even today, because for many applications, in which ODEs occur, numerical time integration methods are now demanded which more comprehensively fulfill the mathematical properties of the underlying problem. We return to this point in Sect. 5.5.4.

Examples for multidimensional simulation codes used to study stellar convection which at least originally have used or still use Runge–Kutta methods include StellarBox (classical, explicit, 4-stage, 4th order scheme, Wray et al. 2015), MuRAM (alternative, explicit, 4-stage, 4th order scheme, Vögler et al. 2005), for example, or ANTARES (Muthsam et al. 2010), which uses a special class of such methods to which we turn in Sect. 5.5.4. Further details may also be found in the references given in Sect. 5.6.

#### Linear multistep methods

The most well-known linear multistep methods are the already mentioned leap frog scheme, the predictor–corrector methods (see Hairer et al. 1993), and the backward differencing formulae for stiff problems (see Hairer and Wanner 1996). They achieve a higher approximation order by storing information from previous time steps and reusing them to construct extrapolations for the new time step (just as Runge–Kutta methods do, but with a single stage involving the stored information from the last step or few steps). Their main advantage is to achieve a high order of approximation with typically just one or two evaluations of the right-hand side of the differential equation per time step (i.e., for hyperbolic conservation laws: the fluxes at the grid cell boundary). The leap frog method indeed just needs one evaluation, predictor–corrector methods two, and backward differencing formulae one although they typically have to be evaluated iteratively, since these are implicit time integration methods (with Euler backward being again the simplest case as for implicit Runge–Kutta methods). This comes at two main disadvantages: changing the time step while keeping the full integration order requires a lot of extra work and information from previous time steps may have to be stored which can less easily be rearranged to minimize storage, especially for the case of higher order methods. This may be at the root of why at least the higher order methods in this class are less commonly used today. Contrary to wide spread opinion also the linear multistep methods are the result of an optimization process. Various building blocks (Adams–Bashforth, leap frog, etc.) can be used for the prediction step of a predictor corrector method and as for Runge–Kutta methods their approximation order does not have to be maximized for a given number of time steps for which information has been kept. Rather, additional properties can be optimized as well and we return to this point in Sect. 5.5.4.

As examples for using this approach in multidimensional simulations of stellar convection we mention Kim and Chan (1998) and Robinson et al. (2003) who perform thermal relaxation with the fully implicit Euler backward method accelerated by directional operator splitting, as mentioned above, and proceed for statistical evaluations of the simulation runs with a second order predictor–corrector method. Quite a different example is Stagger (for references see Sect. 5.6.20) which uses the Hyman predictor–corrector method for time integration that is based on a leap frog prediction step and a corrector step which ensures stability for both hyperbolic (advective) and parabolic (diffusive) terms of the hydrodynamical equations while also achieving third order and a less restrictive advective time step, Eq. (11), than many other methods of similar order of accuracy.

#### Strong stability preserving (SSP) methods

The advent of higher than first order Riemann solvers described in Sect. 5.2.4 as well as Sects. 5.2.5 and 5.2.6, in particular of schemes having total variation diminishing (TVD) and ENO properties, introduced new demands on time integration schemes which had previously not been considered. In short, if an initial condition is “measured” by the so-called TVD semi-norm, the time integration scheme has to prohibit that this semi-norm increases as a function of time when measuring the solution as a function of time. The idea behind this is that oscillations occurring around a discontinuity have to be avoided or, rather, suppressed to ensure that the Rankine–Hugoniot property (see Sect. 5.2.4) remains fulfilled and hence the entropy solution is followed during time integration. The importance of the TVD property for numerical hydrodynamics was impressively shown by Gottlieb and Shu (1998). Likewise, time integrators used in combination with ENO schemes which in turn take care of the spatial discretization of the advective (and pressure gradient) part of the hydrodynamical equations are required to also not change the ENO properties of the numerical solution as a function of time. Originally, this property of the time integrator was guaranteed by developing time integration methods alongside the spatial discretization.

For the case of ENO methods, e.g., Shu and Osher (1988, 1989) derived explicit Runge–Kutta methods of second and third order and an implicit one of fourth order in addition to the (in practice useless) explicit Euler forward method. While the second order scheme was identified as a classical method already proposed by Heun (1900), it later on turned out that the third order method had previously been derived by Fehlberg (1970). Moreover, the fourth order scheme appeared complicated and the conditions specifying the maximum time step under which ENO properties remain preserved for such methods remained unclear. This completely changed once it was realized that this complicated theoretical work was unnecessary, since it could be achieved by resorting to results from the theory of the numerical solution of ODEs. Already several years before the invention of ENO methods, Spijker (1983) had introduced and defined the conditions of *contractivity* for the numerical solution of initial value problems which led to the definition of *strong stability preserving (SSP)* methods. These have been motivated by ODEs for which the analytical solution is *contractive*, i.e., the difference between two different initial conditions $v(t_0)$ and $u(t_0)$ in some norm, $||v-u||$, is monotonically decreasing as a function of time. A contractive numerical time integrator ensures that this property holds also for the numerical approximation and for Runge–Kutta methods in particular this property is required to hold for each individual stage. It has turned out that the same class of time integration schemes is also suitable to ensure monotonicity of a solution ($||u(t)||\leqslant ||u(t_0)||$ for any $t > t_0$), monotonicity with respect to two different initial conditions, and boundedness of the solution. SSP methods ensure these properties and, as was realized for the special case of the norm used to define the TVD property already by Shu (1988), if a numerical time integration scheme has this property, it suffices to show that the numerical solution of a differential equation has the desired stability property (such as TVD or ENO) with the Euler forward scheme for just sufficiently small time steps. The analysis then is automatically valid for any SSP time integration scheme provided the time step is less than a method dependent maximum time step which in turn depends on the spatial discretization. The derivation of that time step limit is completely done within the numerical analysis of ODEs. Hence, it is completely unnecessary to analyse the different ENO schemes for each Runge–Kutta or linear multistep method separately: an analysis of the Euler forward scheme suffices for each ENO scheme and for considering a particular general linear method for solving ODEs one can just resort to its analysis within ODE numerical approximation theory. In theoretical work on time integration methods used for partial differential equations the focus eventually changed from the TVD to the SSP property, since it is not restricted to the use of a particular norm (cf. Gottlieb et al. 2001). A review on the developments of the entire field can be found in Gottlieb et al. (2009). This also demonstrates the superiority of the method of lines approach over “custom-made” time integration methods for partial differential equations. Indeed, a lot of open questions with respect to Runge–Kutta methods and their application to ENO methods had already been resolved in the seminal paper of Kraaijevanger (1991) on the contractivity of Runge–Kutta methods. He not only demonstrated that the maximum “SSP radius” (i.e., the maximum time step for which the SSP property is ensured) can be uniquely determined for *any* Runge–Kutta method (something that is not clear at all from Shu and Osher 1988, 1989), but that the maximum order in time for any such explicit method is 4 and such a 4$^\mathrm{th}$ order method has to have at least five stages (thus, the methods used in Wray et al. 2015 and Vögler et al. 2005 cannot be SSP methods). Moreover, the methods of second and third order re-derived in Shu and Osher (1988, 1989) are indeed the optimum methods of 2nd and 3rd order (with 2 and 3 stages, respectively). Implicit methods of this kind can be of at most $6^\mathrm{th}$ order (that proof was completed later on by explicitly constructing such a method, see Ketcheson et al. 2009).

On the fly, a whole number of new Runge–Kutta methods were derived in Kraaijevanger (1991), e.g., the method of three stages with largest time step in the SSP sense, which is of second order and called now SSP(3, 2). It is an example of trading convergence order for maximum time step size or “radius of contractivity” (in the language of Kraaijevanger 1991). Since, as already mentioned above, the spatial discretization error is much larger than the time discretization error at grid resolutions achievable in numerical simulations of stellar convection, Grimm-Strele et al. (2015b) indeed found this method to not only outperform its third order cousin from Shu and Osher (1988, 1989), but also in respect to the computational expenses needed to achieve a given accuracy limit and thus in overall efficiency. We recall the most useful explicit Runge–Kutta schemes with SSP property here for convenience by providing their *Butcher arrays*. These list their coefficient matrix *A*, see Eq. (97), to which a leading column is added that contains the relative points in time of each stage (with 0 referring to the old and 1 referring to the new point in time). Those are trivially obtained from a summation of *A* over each row. The final row contains the summation vector *b* of the scheme. The TVD2 scheme (also known as Heun’s second order Runge–Kutta method, Heun 1900) hence reads99while the SSP(3, 2) method of Kraaijevanger (1991) is given by100and the TVD3 method of Fehlberg (1970) reads101—following the analysis in Kupka et al. (2012) the SSP(3, 2) method has become the standard time integration scheme in ANTARES instead of the previously used second and third order methods of Heun (1900) and Fehlberg (1970), respectively.

In the mean time, also for linear multistep methods a thorough analysis of their SSP properties has been made (e.g., Hundsdorfer et al. 2003). Since the whole development is driven by ODE methods and tools, progress is much faster and now also includes operator splitting and partitioned methods such as implicit–explicit Runge–Kutta (IMEX RK) methods which fulfil the SSP property under conditions derived by Higueras (2005, 2006, 2009). This permits to integrate, for example, the hyperbolic fluxes as listed in Table 3, while terms representing diffusion may be integrated by an implicit method, thereby removing time step restrictions such as Eq. (12). This strategy was shown to work for the case of semiconvection in Kupka et al. (2012). In their work, the authors also demonstrated the effect of using a non-SSP method, a classical third order Runge–Kutta method by Heun (1900), for solar granulation simulations (in 2D for that example): with this method the simulation crashed after less than 10 sound crossing times while the second and third order methods of Shu and Osher (1988, 1989) had no problem in completing the intended time interval of 20 sound crossing times from the same initial condition. In Kupka et al. (2012) it was also demonstrated that the same happens for an IMEX RK method method which violates the conditions derived in Higueras (2005, 2006, 2009) and is thus a non-SSP method in this class: the (2D) semiconvection simulation got stuck as soon as strong convection kicked in after an initial diffusive phase. Nothing like that happened for the SSP counterparts. The same held for an apparently ideal IMEX RK method which again does not possess the SSP property: it failed at even increasing the time step beyond that one of an explicit method in the diffusive part. The conclusion from Kupka et al. (2012) was thus that SSP is indeed a very important property for a numerical time integration scheme to be used in the simulation of stellar or gas giant planet convection, if it is based on an ENO-type discretization of the hyperbolic fluxes as listed in Table 3. While the authors had already considered the optimization of an IMEX RK method with two stages and SSP property, it was also clear that better schemes would be possible if a third stage were allowed for. In Higueras et al. (2014) new IMEX RK methods were hence derived. These contain optimizations such that they simultaneously ensure the SSP property for finite time steps as demanded by the criteria of Higueras (2005, 2006, 2009), *uniform* convergence of second order, L-stability (thus relaxation to stationary limits is possible with arbitrarily large time step), and positivity of the implicitly integrated term (if the SSP property is violated with respect to such a term as it is usually more restrictive than necessary in practice), among others. Indeed, these properties follow naturally from what we expect for solutions of advection–diffusion equations in general and the Euler and Navier–Stokes equations in particular and the benefit from these properties was demonstrated in Higueras et al. (2014). We list some useful IMEX RK schemes with SSP property for convenience of the reader. The so-called SSP2(2, 2, 2) scheme (see Pareschi and Russo 2005 for an explanation of this naming) is given by102using the notation introduced with Eq. (98). One can choose $\gamma$ to ensure a particular property of the scheme. In Kupka et al. (2012) it was suggested to choose $\gamma < 0.25$ to ensure positivity of the implicit part of the scheme and the value itself taken as large as necessary to ensure stability and as small as possible to minimize the time integration error. To ensure sufficiently rapid damping for “arbitrarily” large diffusivities would require L-stability. This can be ensured by choosing $\gamma = 1-1/\sqrt{2}\approx 0.2929$ and in this form the implicit part of the scheme has been known for a long time (see Chap. II.7 of Hairer et al. 1993 and Chap. IV.6 of Hairer and Wanner 1996), although its potential to be combined into an IMEX SSP method appears to have been considered first in Pareschi and Russo (2005) and its SSP properties in the now commonly accepted sense of Higueras (2006) were shown in that latter paper. However, with that choice of $\gamma$ the method violates positivity. In either case the scheme does not have uniform second order convergence. To ensure sufficiently rapid damping, positivity, and uniform convergence all at the same time inevitably requires to resort to a scheme with three stages, as is discussed in (Higueras et al. 2014), who also derived the following two schemes with these properties:103and104where the second scheme, Eq. (104), also provides damping for pure advection operators at the cost of a somewhat smaller time step than the scheme of Eq. (103), if the time step limitation is due to the explicit part of the scheme.

What happens, if a scheme does not fulfil these properties? Indeed, one might argue that the SSP together with ENO properties are *sufficient* to compute entropy solutions of the Navier–Stokes and Euler equations, but *not necessary*. What most likely happens in practice is that if stability problems are encountered, a lack of numerical viscosity, artificial diffusion, hyperdiffusion, or subgrid scale viscosity, or the like are blamed to be responsible for that in addition to lack of resolution. As a “cure”, the viscosity could be boosted. We suggest that instead also the time integration method should be checked. Instead of losing resolution by boosting viscosity, it might be sufficient to change to a more appropriate time integration method. Since “text book methods” have been derived long before this theoretical branch of numerics of ODEs has been developed to its present state, such methods can be expected to have the suitable properties only by chance, as they are usually not the topic of introductory books on the subject.

### Some codes

This subsection provides a short overview on a number of codes. We preferentially include codes which

- are used in convection studies or
- seem to be of a scope as to be useful for such a purpose and
- for which there exists a fairly detailed description or even documentation.

Beyond that we have mainly included newer codes because recent years seem to have witnessed a change towards more detailed discussion of numerical methodology in papers introducing a code. One further point was that the methods used should possess potential for the future. Given the advances in numerical methods that also leads to a bias towards newer codes.

As a consequence, meritorious software with to which we owe important results in convection studies, perhaps conducted even today, may go unmentioned here because of the long useful lifespan of such codes. Last but not least want of space and ignorance on our part may be made responsible for omissions in the alphabetic listing below.

#### ANTARES: A Numerical Tool for Astrophysical Research

*Basic reference:* Muthsam et al. (2010).

**Fig. 13:**
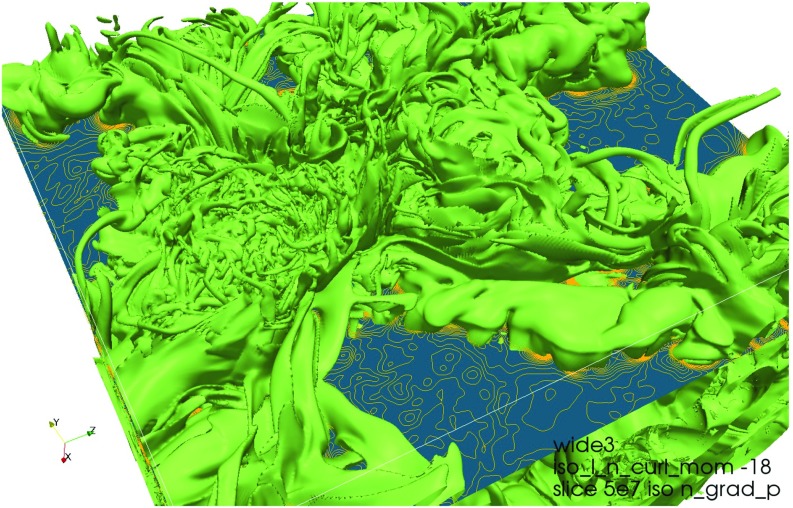
Solar surface convection (granulation) in high resolution. The domain is 1.2 Mm wide (secondary grid refinement in a larger domain). Essentially above the horizontal cut (*orange lines*: isolines of pressure gradient) is the photosphere. In the middle of the domain is a granular downdraft. The turbulent nature of the downdraft manifests itself in numerous vortex tubes made visible in the green isosurface of vorticity. Figure courtesy H. Grimm–Strele

**Fig. 14:**
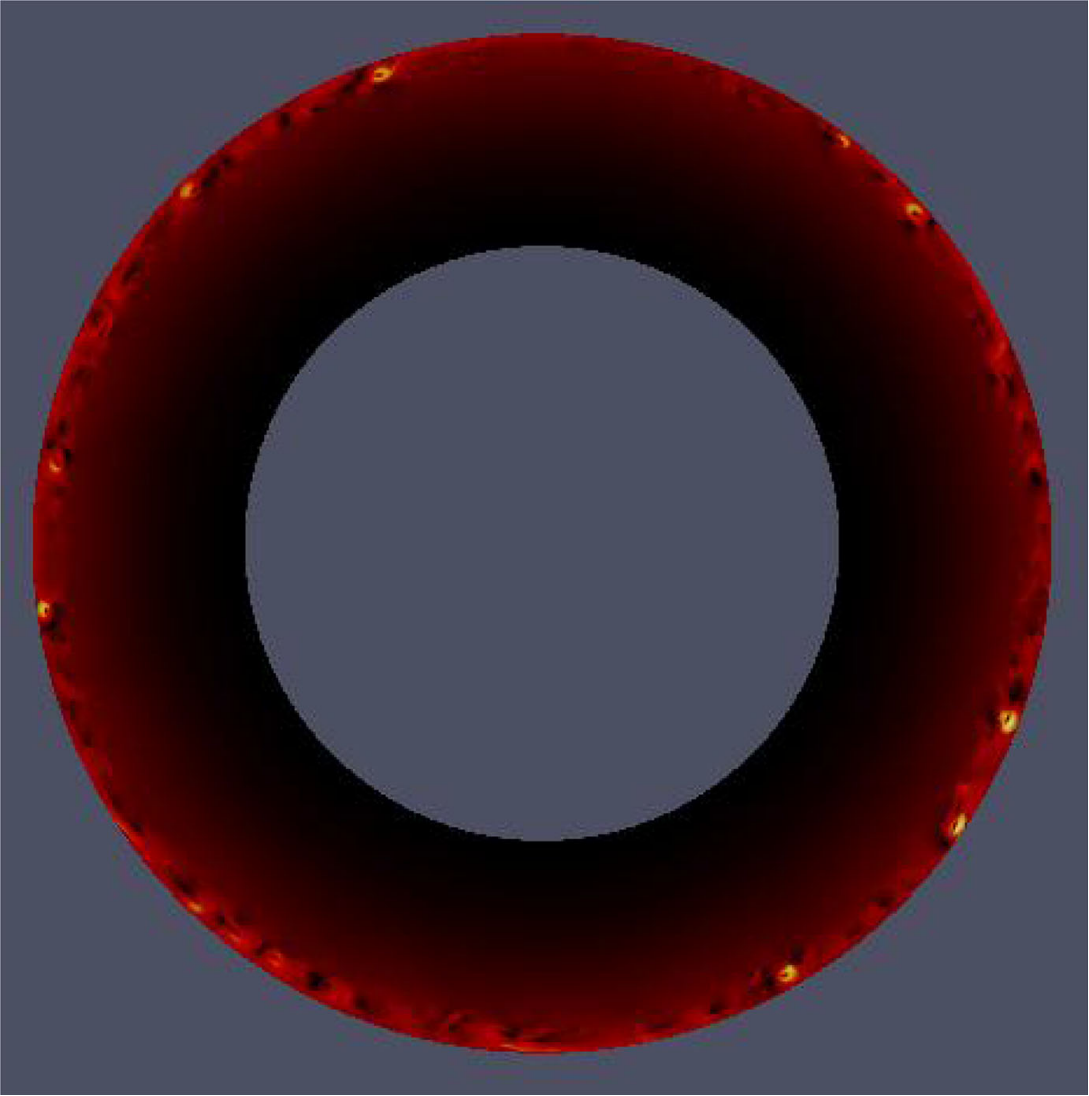
He ii ionization zone (near the *top*) of a Cepheid, a radially pulsating star, for studying pulsation–convection interaction. *Colours* magnitude of velocity after subtraction of the radial pulsation component. The H $+$ He i convection zones and the atmosphere (not calculated in that model but for related ones) would barely be visible at that scale. Although these calculations are only 2D, they are quite demanding in terms of computer time. Figure courtesy E. Mundprecht

ANTARES is a code for 1D, 2D and 3D (radiation-)(magneto-) hydrodynamics. It relies on various types of ENO methods, weighted ENO of order 5 being most frequently used in practice. It includes idealized or realistic microphysics and opacities. Radiative transfer can be treated both in the diffusion approximation and with the static transfer equation. Opacity can be non-grey via opacity binning. Grid refinement in logically rectangular patches (and in time as well) is possible, even recursively. For studies of pulsating stars, polar coordinates and a radially moving grid are included, presently in 1D and 2D (e.g., Mundprecht et al. 2015). A different opportunity for flows (in 2D as of now) is given by the use of curved grids (Grimm-Strele et al. 2014). Also included are the Boussinesq approximation (Zaussinger and Spruit 2013) and the low Mach number method by Kwatra et al., discussed in Sect. 5.3.1, even for binary mixtures (Happenhofer et al. 2013). As an example for the versatility of a modern code we include figures on solar granulation, Fig. 13, and on the He ii convection zone of a Cepheid, Fig. 14.

#### APSARA

*Basic reference:* Wongwathanarat et al. (2016).

APSARA is a 4th order explicit hydrocode for the Euler equations. It is built on a method initiated by Colella et al. (2011) and later extended in McCorquodale and Colella (2011) and Guzik et al. (2012). It is a finite volume method using mapped Cartesian coordinates in 3D.

#### ASH: Anelastic Spherical Harmonic

*Basic reference:* Clune et al. (1999).

The ASH code considers anelastic magnetohydrodynamic equations in a spherical shell, potentially including Coriolis effects. Sphericity is accounted for by expansion in spherical harmonics in the lateral directions. See also Nelson et al. (2013). It is an outgrowth of the early Glatzmaier (1984) code where issues of numerics, physical scope and implementation have been developed independently.

#### CASTRO

*Basic reference:* Almgren et al. (2010).

The numerics of the hydrodynamic solver is mainly based on an unsplit (instead of directionally split) version of PPM (Miller and Colella 2002) with improved limiters better taking care of smooth extrema. Automatic mesh refinement in space and time is provided. CASTRO has an option for self gravity and nuclear reaction. There exists an online users guide.

#### CHOLLA: Computational Hydrodynamics on Parallel Architectures

*Basic reference:* Schneider and Robertson (2015).

CHOLLA is designed for MHD. This is witnessed by the basic numerics (Corner Transport Upwind, CTU). The reference above mainly deals with the hydrodynamic aspects. Various reconstruction techniques are used, among them two variants of PPM. Notably, CHOLLA heavily uses hardware acceleration provided by graphics cards.

#### CHORUS: Compressible High-Order Unstructured Spectral Difference

*Basic reference:* Wang et al. (2015).

Designed for convection in rotating spherical shells, CHORUS uses spectral difference methods on an unstructured grid. Numerics is designed for Mach numbers of order *O*(1). With long-time simulations in mind, a correction step enforcing angular momentum conservation is included.

#### CO5BOLD: Conservative Code for the Computation of Compressible Convection in a Box of L Dimensions, L $=$ 2, 3

*Basic reference:* Freytag et al. (2012).

This is a (radiation-)(magneto-) hydrodynamic code. It can be used in two modes: star-in-a-box (for simulating a whole star) or box-in-a-star. The numerics is more closely being described in the reference given above. It makes use of Riemann solvers, adapted for specific needs of the problems, flux limiting concepts and specially designed viscosities for further stabilization. Realistic microphysics and opacities (for use with radiative transfer in the diffusion approximation or invoking the static transfer equation) are provided. Via opacity binning radiative transfer can be non-grey.

#### DEDALUS

*Basic reference:* Dedalus project homepage.^9^

Dedalus is a framework implementing spectral methods with various basis functions for different dimensionalities. Basically designed for but not limited to fluid dynamics, it allows for specification of equations and invocation of solvers in a symbolic manner. Dedalus is Python based and MPI parallelized. Besides the existing tutorial and examples on the webpage, a more detailed prescription is in preparation.

#### ENZO

*Basic reference:* Bryan et al. (2014).

ENZO is an MHD code originally intended for cosmological hydrodynamics. It has evolved adding a number of modules which render it useful for many purposes. Included are various solvers for the hydrodynamic equations, among them a PPM variant and a MUSCL variant. Radiative transfer can also be included by ray tracing. Among the options there is also mesh refinement. A part of the code has been ported to the CUDA framework so as to make use of graphics cards for computation.

#### FLASH

*Basic reference:* Flash Center for Computational Science.^10^

The link given above provides easy guidance to the options of FLASH. In the hydrodynamic mode, various flavours of PPM and ENO are included. MHD is possible via a staggered mesh algorithm. Radiative transfer can be included using flux limited diffusion approximation. Various kinds of microphysics are available. Automatic mesh refinement is included as are a number of other options.

#### Glatzmaier code

*Basic reference:* Glatzmaier (1984).

This is a code envisaging spherical shells, used in astro- and geophysics based on the anelastic approximation. Laterally, functions are represented by spherical harmonics, the radial part is treated by finite differences or representation via Chebyshev polynomials. A newer version uses the Lantz–Braginsky–Roberts approximation. The Glatzmaier code is the basis for a number of other codes, further developed independently (ASH, MAGIC and Rayleigh codes, described in their place). An illustrative figure how such a code evolves is given on page 3 of this presentation^11^of the Rayleigh code.

#### Leeds code

*Basic reference:* Jones and Kuzanyan (2009).

This is a pseudospectral code for possibly rotating spherical shells. It adopts the Boussinesq and anelastic approximations. While not providing a description of the numerics of the code, the reference above yields insights as to its scope.

#### MAESTRO

*Basic reference:* Nonaka et al. (2010).

MAESTRO is based on a set of low Mach number equations. It works in a local mode in 2D and 3D and, for a shell, in 3D. It allows for a time dependent base state plus automatic mesh refinement. Code and description are available here.^12^

#### MAGIC

*Basic reference:* MAGIC.^13^

MAGIC allows for investigations of (magneto-) hydrodynamic problems in spherical shell with Coriolis forces using the Navier–Stokes, anelastic, or Boussinesq equations. It is an outgrowth of the early Glatzmaier (1984) code where a number of aspects, including magnetohydrodynamics, parallelization and postprocessing have been developed independently. For details the link above and Wicht (2002).

#### MURaM: the Max Planck Institute for Aeronomy/University of Chicago Radiation Magnetohydrodynamics code

*Basic reference:* Vögler et al. (2005).

MURaM is designed for investigating granulation and the upper part of the convection zone of the Sun and solar-like stars. For MHD it uses centered differences which are stabilized by artificial viscosities. Realistic microphysics and opacities are included, non-grey radiative transfer is provided via opacity binning.

#### MUSIC: Multidimensional Stellar Implicit Code

*Basic reference:* Viallet et al. (2011, 2016).

This code treats not only radiative transfer (in the diffusion approximation) implicitly but includes also the Euler equations in the implicit treatment (2D, 3D). Recently, a 3D module has been developed. It uses a *semi*implicit method (Viallet et al. 2016) designed for good stability under large timesteps. It will eventually serve as a preconditioner for the fully implicit method. MUSIC is intended for simulating large portions of a star.

#### PENCIL

*Basic reference:* Pencil code homepage.^14^

This is an MHD code for the compressible case, presently developed further by a community. It is based on sixth order finite differences and viscosities for stabilization. An extensive manual is provided at the homepage for use of the code, also with a few details on numerics.

#### Prometheus and PROMPI

*Basic reference:* Prometheus: Fryxell et al. (1989); PROMPI: Meakin and Arnett (2007).

Prometheus is a PPM-based code designed for situations with abundance changes. Its PPM core has been adopted in PROMPI (and also in the FLASH code) for box-in-a-star simulations.

#### Rayleigh code

*Basic reference:* Rayleigh code overview.^15^

The Rayleigh code is also a descendant of Glatzmaier’s code. The basic reference cited above gives a good overview in particular on the efforts undertaken to massively parallel programming, obviously in particular with architectures such as the IBM Blue Gene in mind.

#### Stagger

*Basic reference:* Stagger code homepage.^16^

The Stagger code has a long history. It is a (radiation-)(magneto-) hydrodynamics code mainly used for realistic 3D modelling of stellar atmospheres and some regions below. Its numerics is based on sixth order accurate derivatives and fifth order interpolation, making use of a staggered mesh. The numerics is stabilized by hyperviscosity. A short description of the present state can be found in Magic et al. (2013). For a more original version, see the Nordlund and Galsgaard paper from 1995, available here.^17^

#### StellarBox

*Basic reference:* Wray et al. (2015).

StellarBox is a (radiation-)(magneto-) hydrodynamic code intended for realistic simulation of solar and stellar convection. The basic numerics is a finite difference scheme. The use of Padé approximations for calculating derivatives allows a small (3 nodes) stencil for fourth order. Various subgrid models purporting also stabilization are included.

#### TENET

*Basic reference:* Bauer et al. (2016).

The numerics in TENET which aims at solving the Euler equations of hydrodynamics is based on a discontinuous Galerkin method augmented by slope limiting strategies on cubic cells. The basis function within each cell are products of Legendre polynomials. While the results are promising in terms of efficiency, the new methodology has to be considered as being still under development (as the authors themselves give to understand) for issues such as representation of shocks.

#### XTROEM-FV

XTROEM-FV (Núñez-de la Rosa and Munz 2016a) is an MHD code based on very high order finite volume WENO schemes with switches to lower order for shocks. Much emphasis is devoted on *magneto*hydrodynamic aspects (also for relativity, Núñez-de la Rosa and Munz 2016b). 1D and 2D tests are provided in that paper.

## Modelling: limitations, tasks, and chances

Each way of modelling stellar convection, whether based on numerical solutions of (1)–(10), or any simplification thereof as discussed in Sect. 4, or based on additional hypotheses introduced for the 1D models discussed in Sect. 3, has its own limitations with respect to applicability and achievable, predictive accuracy. We discuss quite a few of them in Sects. 2–5. In this concluding section we want to summarize the strengths and weaknesses determining the region of applicability of both 1D models and 3D numerical simulations. Before we review them in further detail we introduce a set of criteria useful for judging the methods we have to model stellar convection.

### Requirements for astrophysical convection modelling

In addition to the evident criterion of successfully recovering and explaining astrophysical observations, two properties come immediately into mind in discussions about what requirements convection modelling should fulfil in astrophysics: low computational costs and “lack of free parameters”. While the first one is undeniably important in the sense that computations, no matter what they are based on, have to be finished in an acceptable and definitely finite amount of time, the debate on the presence or lack of “free parameters” has been one of the most vivid and arguably one of the least helpful discussions held in stellar astrophysics during the last decades. Before we present an alternative set of criteria which includes the requirement of agreement with observations and of computational affordability, we hence discuss the issue of parameter freeness in Sect. 6.1.1 and explain why we do not include it among the criteria listed in Sect. 6.1.2.

#### The issue of parameter freeness

The mixing length *l* of MLT and the “free” parameter it entails, typically $\alpha = l/H_p$, have provided a continuous source of discomfort in stellar astrophysical modelling. Although $\alpha$ might be calibrated so as to match the present Sun (Gough and Weiss 1976), it has to be kept changing in applications: whether one would like to include a non-grey model atmosphere on top which also matches both the Balmer lines $\hbox {H}_{\alpha }$ and $\hbox {H}_{\beta }$ at the same time (Smalley et al. 2002; Heiter et al. 2002) ($\alpha = 0.5$) as well as the solar radius (Montalbán et al. 2004) ($\alpha = 1.6$), or add microscopic diffusion (Samadi et al. 2006) ($\alpha = 1.76$) or account for both a non-grey model atmosphere with Balmer lines matching observations and the correct solar radius at the same time (switch from $\alpha = 0.5$ to $\alpha = 2.51$ as a function of depth), already for the Sun $\alpha$ is anything but constant even completely within the framework of 1D modelling. Changing abundances or opacities is one more reason for modifying $\alpha$, for instance, in stellar evolution models for the red giant branch (Ferraro et al. 2006). The necessity of strongly varying $\alpha$ to fit observational data of the red giants branch for different stellar masses (Stothers and Chin 1995) (from $\alpha = 2.8$ to 1.8 for masses $M/M_{\odot }$ between 3 and 10, where $M_{\odot }$ is the solar mass) or opposite to the red giant branch stars when considering A-type stars instead (Smalley and Kupka 1997; Smalley et al. 2002; Heiter et al. 2002; Kupka and Montgomery 2002) (Strömgren metallicity index, Balmer lines, maximum of the convective flux, all pointing towards $\alpha \lesssim 0.5$) are well-known examples out of many others published in the literature and explain the frustration associated with this parameter: its choice depends on the input physics, the observational quantity considered, and the stellar parameters of the object to be described with a stellar model. Clearly, one would like to get rid of such a parameter.

There is just one problem: *there is no approach without model parameters unless the flow considered is so idealized as to have little to do with a real world flow* (see also Sect. 3.2.3).

The good news about this problem is: *it does not always matter*. The problem with $\alpha$ in MLT is not the need to determine it, but the properties that model has as a whole: it is highly sensitive to and depends on changes of $\alpha$. Changes are often necessary as soon as different stellar conditions are considered, the input (microphysics) is modified, or a variety of observational quantities has to be predicted. In the conclusions of Canuto (1996) it was pointed out that the ability to adapt MLT to different circumstances may have been responsible for the *“illusory resiliency”* of the model and the flexibility provided through modifying $\alpha$ has been *“misconstrued as a sign of physical robustness”* rather than as a means of escaping falsification. Indeed, its results are *not robust* to small changes of the parameter which in turn is highly dependent on the type of observations studied and even the type of object considered, thus *anything but universal*, nor is it *independent of the object modelled*, and it tries to escape falsification even though it eventually fails in this, too (cf. Samadi et al. 2006). This is a deficiency of much greater importance than the necessity to calibrate a parameter per se. We come back to these points below but first we demonstrate that there is no escape from parameters as such, before we argue why parameter freeness is of much more limited importance than a set of properties the modelling approach should have as a whole.

Clearly, the *physical parameters* describing the star as a whole such as *effective temperature* or *mass* or *radius*, cannot be considered a problem, since they are related to *observable quantities* which have to be determined *for each object* and set the stage for modelling. But there are more *parameters which cannot be avoided*.

*No escape from parameters introduced through the numerical method* Parameters of the numerical method itself appear in both numerical simulations as well as 1D models. They may stem from spatial discretization (see Sect. 5), but in particular they are built into the time integration method. A famous example is the so-called $\vartheta$-parameter of the generalized time integration method $({\bar{u}_{i,j}^{n+1} - \bar{u}_{i,j}^{n}})/\tau = \vartheta f_{i,j}^{n+1} + (1-\vartheta ) f_{i,j}^{n}$ for an ordinary differential equation (ODE) $\partial _t u = f(u)$ as obtained for a partial differential equation after spatial discretization at points indexed by $\{i,j\}$ at a time level *n* (see, e.g., Richtmyer and Morton 1967). A choice of 0 yields the Euler forward method, 1 / 2 the Crank–Nicolson method, and 1 the Euler backward method. By varying $\vartheta$ one can trade accuracy and order (maximal if $\vartheta = 1/2$) for stability (optimal if $\vartheta = 1$). The numerical parameters of time integration schemes are thus the results of *optimization procedures* and this holds in fact for *any* of them (see Sect. 5.5). But that is not a problem: these parameters are usually chosen on purely mathematical grounds unrelated to the specific application at hand. For instance, they should ensure properties of the numerical solution which the exact differential equations have (L–stability and positivity, flux conservation, etc.) while at the same time minimizing the integration error. The $\vartheta$-method is an exception: because it only depends on a single parameter, ODE/PDE packages often permit the user to change $\vartheta$ whereas for different versions of Runge–Kutta time integrators or predictor–corrector schemes only fixed sets are “offered”. The key point here is that the *numerical parameters* should be seen as quantities solely chosen on mathematical grounds. As long as one does not attempt to optimize them in direct connection with the physical problem to be solved, they are just part of optimizing the numerical approximation itself before even beginning with any astrophysical investigations. Of course, a numerical method suspect to failure can and should always be changed. The most important property which has to hold for any good numerical approximation of an analytical solution is that such details do not matter: a sequence of better resolved simulations indicates convergence to a (sensible) solution and changing the numerical method by another, adequate one only leads to possibly a different convergence rate (more or less grid points might be required, etc.), but not other fundamental differences. Clearly, *parameters of the numerical method do not matter*, as long as the *solutions it provides are robust and physically sensible*.

*The parametrization of boundary conditions* Requiring horizontally periodic boundary conditions is mathematically simple. But for the vertical boundaries of numerical simulations of stellar convection a difficult choice has to be made: shall one impose a zero vertical velocity or not? This is an easier choice for objects with shallow surface convection zones where the entire convective and overshooting region can be included in the simulation box (cf. Fig. 4 in Sect. 2.3.4 or various of the simulations presented in Tremblay et al. 2015, e.g.). But how about simulations of solar granulation? In this case, two options have been pursued which we consider here for the lower boundary condition. To indeed impose $u_\mathrm{vert} = 0$ (inside the convection zone!) and introduce a boundary layer with artificially enhanced diffusivity which transports the total flux into the domain, for instance, as in Kim and Chan (1998), Robinson et al. (2003) and Muthsam et al. (2010). Alternatively, a free outflow is permitted with constraints on the inflow such as being isentropic and either strictly vertical or require $\partial _x u_\mathrm{vert} = 0$ (i.e., a constant velocity). Examples for this approach include Stein and Nordlund (1998), Vögler et al. (2005) and Freytag et al. (2012). A detailed comparison of both methods is given in Grimm-Strele et al. (2015a). This might seem “parameter free”, but it is not. The approach in Kim and Chan (1998), Robinson et al. (2003) and Muthsam et al. (2010) involves the specification of an arbitrary function to boost the radiative conductivity to a value such that $F_\mathrm{total} = -K_\mathrm{boosted} \partial _x T$ with $K_\mathrm{boosted} \gg K_\mathrm{rad}$ at the boundary while a few grid cells further above $K_\mathrm{boosted} \rightarrow K_\mathrm{rad}$. The approach of Freytag et al. (2012) as discussed also in Grimm-Strele et al. (2015a) requires to also specify *two relaxation constants* in addition to $\partial _x u_\mathrm{vert} = 0$ which ensure no artificial flow features are generated at inflows which could develop erroneous shocks and it was also considered more realistic to permit some fluctuation of entropy around its mean value. In Vögler et al. (2005) a relaxation rate was coded into the specification of the influx internal energy based on the radiative flux emitted at the surface (see also Grimm-Strele et al. 2015a). Finally, Stein and Nordlund (1998) mention to apply damping to the horizontal and vertical velocity of the incoming fluid *“using a long time constant”*. At face value, *any of these models of the lower vertical boundary of the granulation simulations entails some constants*. The key point again, however, is: does it really matter? Beeck et al. (2012) compared vertical temperature and density profiles, center to limb variation of the continuum intensity, and other quantities and found good to very good agreement between the STAGGER, MuRAM, and CO$^5$BOLD codes *despite their different boundary conditions* of Stein and Nordlund (1998), Vögler et al. (2005) and Freytag et al. (2012) (in updated versions for the former two). In fact, even when comparing solar granulation simulations made with CO$^5$BOLD with simulations based on closed boundary conditions as described in Robinson et al. (2003) and Muthsam et al. (2010), the temperature profile and other quantities close to the region where the temperature gradient is steepest, i.e., the superadiabatic peak, yield acceptable to good agreement (Kupka 2009a). Evidently, the thermal structure or spectral line profiles are robust to changes of even the entire boundary condition model. Thus, for the quantities usually predicted also with MLT the results of 3D LES are robust with respect to the details of the vertical boundary conditions and in particular to the parameters their models involve. However, the actual velocity distribution function does depend sensitively on those details (Grimm-Strele et al. 2015a), particularly on the choices of parameters in the boundary conditions, although these are quantities which MLT could not even predict to begin with.

*Inevitability of numerical viscosity* As explained in Sect. 4.2.3 some kind of viscosity, whether it be built into the numerical scheme as in Muthsam et al. (2010) or explicitly added as in Stein and Nordlund (1998), Robinson et al. (2003), Vögler et al. (2005) and Freytag et al. (2012) is inevitable to any method which is supposed to compute the hyperbolic (advective fluxes) of the Euler and Navier–Stokes. This cannot be avoided even for direct numerical simulations which resolve all scales down to $l_\mathrm{d}$ (Sect. 2.2.1) and comes in addition to accounting for processes on unresolved scales in LES through some subgrid scale model (see Pope 2000 and Sect. 2.1.1) whether it be hyperviscosity, such as in Stein and Nordlund (1998) and Vögler et al. (2005), or a Smagorinsky type viscosity as used in Robinson et al. (2003), Freytag et al. (2012), or Wray et al. (2015), and optionally also in Muthsam et al. (2010) and Mundprecht et al. (2013). Reasons have been published to prefer one approach in comparison with another (Jacoutot et al. 2008). But returning to the comparisons shown in Kupka (2009a) and Beeck et al. (2012) it is found that the bulk properties of simulations of solar granulation, for example, are not very sensitive to that choice *as long as a sufficient minimum resolution* is achieved. For all the cases studied in Beeck et al. (2012), for instance, this clearly holds true (vertically, $h \lesssim 15$ km for those simulations).

*But the key issue is: show that and show why it does not matter* We recall here that the comments on parameters of 3D LES of stellar convection made in the three preceding paragraphs are restricted to the case of numerical simulations of convection at the surface of stars. But at least in principle the discussion can be carried over also to other cases. In the end also the models of stellar convection based on 3D numerical simulations are not free from parameters. But these have a very different nature than the $\alpha$ parameter of MLT: small changes in the parameters have little effect on the results provided the simulation model is physically complete enough and sufficiently well resolved. They do not have to be varied, neither as a function of stellar parameters, nor in dependence of the observable to be studied. They can to a large extent (depending on the specific methods used) be calibrated completely without any use of astrophysical observations. Thus, the 3D LES of stellar surface convection can be compared to observations and no need is generally found to adjust parameters to “escape” falsification as one continuously has to do when using MLT for similar purpose. If a part of the model, say the vertical boundary condition, is replaced, it is generally done so for improvement of the model from mathematical or physical points of view, but not as a “routine exercise” to fit some observational data for a particular object. This demonstrates that “parameter freeness” of a model is not a first order problem: rather, it is the nature of the entire model and how its parameters are adjusted which are of primary importance. Motivated by this discussion we hence suggest an alternative approach to decide on the physical reliability, feasibility, and trustworthiness of the different ways of modelling convection.

#### An alternative set of criteria

Since the quest for a parameter free modelling of stellar convection is of limited use in practice, we would like to suggest an alternative set of criteria which is more related to physical and mathematical model properties. Some of them have already been introduced in Sect. 3.1. We recall them here for convenience and note that they are also important to judge the reliability of 3D numerical simulations of stellar convection.

1. correct physical *dimension*;
2. *tensor invariance* and in general proper behaviour under standard coordinate transformations (Galilean invariance, etc.);
3. respecting *symmetries* of the basic variables;
4. physical and mathematical *realizability* of the approximation.

While correct physical dimension is expected to be trivially fulfilled by any well-tested 3D numerical simulation code, tensor invariance and respect of standard coordinate transformations is a property that the mathematical discretization and possibly the subgrid scale model as well as the chosen boundary conditions should ensure. Similar should hold for, say, symmetries with respect to sign change (where appropriate) and, of course, also a 3D simulation should yield a solution that is physically and mathematically realizable. We now extend these criteria by the following ones:

5. *robustness*: the predictions of a model of convection or a numerical simulation thereof should be robust to reasonable changes in model internal parameters or to a replacement of the model component containing such a parameter by a physically and mathematically acceptable alternative possibly containing other parameters which should be robust in this sense, too;
6. *universality*: it should not be necessary to recalibrate internal model parameters for different types of objects, say, for the simulation or modelling of an A-type star or DA type white dwarf instead of the Sun;
7. *computability*: the modelling approach, whether a 1D local, a non-local model, or 2D or 3D simulation, should yield a formalism, a set of equations, which can at least approximatively be solved on a time scale affordable to *present* means of computing;
8. *physical verifiability*: the model or simulation should make predictions which are physically verifiable, or rather, falsifiable, either by agreement with observations or by comparisons to direct (all scale resolving) numerical simulations or by comparisons to an approach which has successfully passed such tests;
9. *independence of choice of model internal parameters from the object to be modelled:* the internal parameters of any model or simulation should not be calibrated by modifying them until the observational data of the physical object to be studied are reproduced. At best, the parameter calibrations should not be done on any astrophysical data at all.

Whereas the exact *quantification* of, for instance, *robustness* is certainly subject to further debate, the main intention is quite clear: if the success of a modelling approach depends on fine tuning a parameter of some closure approximation, a scale length, or a numerical viscosity, by 1%, it is intuitively unacceptably sensitive to the parameter which then becomes a critical issue. If one may change the Smagorinsky subgrid scale parameter (Smagorinsky 1963) from 0.1 to 0.2 (i.e., a 100% change) or even entirely change the concept of effective viscosity (to artificial diffusion, etc.) and one still can recover the same results, for instance, when comparing spectral line profiles such as was done in Beeck et al. (2012), the modelling approach is clearly robust. The desire for *universality* is clearly what has been behind the quest for “parameter free modelling”, but we consider this milder requirement sufficient, since it can also be quantified (i.e., the level of parameter change and the amount of uncertainty which follows from it can more easily be made subject of a quantitative study). Obviously, whatever the means of constructing a model, it is of no use if we cannot finish the calculations it requires in acceptable time, hence the necessity for an approach to be *computable*. For the same reason we suggest both the model predictions itself and the uncertainties introduced by model internal parameters be subject to experimental *tests* whether through astrophysical observations or numerical modelling free from their influence or through a carefully verified chain of such comparisons. Finally, if model parameters cannot be avoided, they should not be *tuned* such as to improve agreement with observations of an astrophysical object and support its interpretation this way. Such an approach hides errors of the way convection is modelled or mixes them up with other modelling errors. In the end such an approach reverts the modelling process from a prognostic one to a completely diagnostic one and removes the possibility to find deficiencies in the model. It would thus prevent physical verifiability and the falsification of physical hypotheses. In practice, small adjustments of some parameter in a model might remain necessary, e.g., in a stellar evolution calculation some reference property must be reproduced which may be possible for one set of input microphysics but not another—but this is related to the overall uncertainty of the entire model and the treatment of convection should not be the motivation to make such changes.

We now have a closer look on how well different one and multidimensional modelling approaches deal with these criteria to obtain a better insight into their strengths and limitations.

### One dimensional models

#### Local models

We consider the two most frequently used strands of local models of stellar convection more closely.

*MLT* As discussed already in Sect. 6.1.1, the empirical calibrations of “free” parameters of the classical local models, in particular of MLT, have become a major source of discomfort. MLT has been essential to develop the theory of stellar structure and evolution to its present state. It played an important role also in the development of 1D stellar atmospheres which have been used for abundance analyses, synthetic photometry, and spectrophotometry. Stellar population synthesis depends on each of these fields. And finally the modelling of pulsating stars initially also relied on MLT as a model of convection, but indeed this is where its inadequacies became apparent first (cf. Unno 1967; Gough 1977b). For a long time both calibrations of its parameter $\alpha$ to obtain the correct radius and luminosity for the present Sun, as studied in Gough and Weiss (1976), as well as photometric calibrations of red giants and globular cluster stars (Alonso et al. 2000; Ferraro et al. 2006) have been used. While in past decades this was considered acceptable given the limited precision of observations when the model had originally been developed and used, the situation has changed during the last two decades thanks to precision photometry, spectroscopy, and progress due to helio- and asteroseismology. In this context we argue that the difficulty with MLT is not the necessity of adjusting parameters per se, but its lack of physical completeness and how the parameter adjustments have to be made to keep it “working”. Clearly, the criteria of correct dimension, tensor invariance, respect of symmetries, and realizability are not a problem for MLT. With respect to *computability* the model is difficult to beat due to its formal simplicity. However, the *robustness* of the calibrations is poor (cf. the sensitivity report in Kupka and Montgomery 2002). Evidently, the huge parameter spread discussed in Sect. 6.1.1 demonstrates that the calibrations are by no means *universal*. Only as long as $\alpha$ is not tuned to reproduce various sets of observations, *physical verifiability* is ensured. The results obtained through comparisons with mode driving (Samadi et al. 2006) or stellar photometry of central main sequence stars (Smalley and Kupka 1997) provided falsifications which cannot be “corrected by parameter tuning”. Arguably the biggest deficiency of the entire approach from the viewpoint of the criteria listed in Sect. 6.1.2 is probably that the *internal model parameters are not independent from the object being modelled*. Indeed, just the opposite is the case if parameters get adjusted case by case to reproduce observational data. This is highly unsatisfactory as it takes away the prognostic power of the model. Given the limitations the model has from a physical point of view, it is not surprising that this has happened and that a replacement of the model is much wanted (we recall that the full MLT model actually has more adjustable parameters but tuning them appears to have been of little use to overcome its limitations).

*CM/CGM models* The series of models published by Canuto and Mazzitelli (1991, 1992) and Canuto et al. (1996) have found some use as alternative and replacement of MLT. They were derived with two-point closure turbulence models (cf. Sect. 3.3) to replace $\varPhi (S) = \varPhi ^\mathrm{MLT}$ in Eq. (26) with new functions $\varPhi (S)^\mathrm{CM}$ and $\varPhi (S)^\mathrm{CGM}$, respectively. A summary of these “turbulent heat conductivities” can also be found in Heiter et al. (2002). Naturally, the turbulence models are also subject to closure hypotheses and entail parameters which are, however, determined ahead of applying the final result to astrophysical problems. In this sense its parameters are “determined” except for a scale length *l* which again has to be calibrated in some form. The original proposal (Canuto and Mazzitelli 1991) had been to take $l = z$ with *z* being the distance to the nearest stable layer, similar to the mixing length used in engineering applications, but in fact already proposed in the context of MLT by Böhm and Stückl (1967). For if the local picture of a convection model should hold, any structure (bubble, eddy) should not reach into stable layers (thus, *l* was taken as the minimum of *z* and $H_p$ in Böhm and Stückl 1967). In this form the model quickly failed the test of matching the present Sun (Canuto and Mazzitelli 1992) as soon as input opacities assumed for the solar model changed and in the end Canuto et al. (1996) proposed to use $l = z + \alpha _{*} H_{p, \mathrm top}$ with $\alpha _{*} < 0.2$ to account for the possibility of a small amount of overshooting into stable layers (and thus allowing for somewhat larger eddies). In practice, however, many authors have used the model of Canuto and Mazzitelli (1991) and particularly Canuto et al. (1996) with $l = \alpha H_p$, e.g., Samadi et al. (2006). In these applications, however, $\alpha < 1$ was typically found to suffice (which one would require within the Boussinesq approximation underlying these models as well as MLT anyway). With respect to correct dimension, invariance with respect to transformation, symmetry properties, realizability, and computability these models come close to MLT. The overall judgement with respect to the weaknesses of MLT appears to be that for the CM/CGM models the scale length *l* requires less tuning and more observational quantities can be recovered without changing *l* (e.g., Baturin and Mironova 1995; Stothers and Chin 1995; Smalley and Kupka 1997; Smalley et al. 2002) or at least provide a quantitative improvement (e.g., Samadi et al. 2006). However, as also pointed out in Canuto (1996), a model of stellar convection must be falsifiable just as any other physical model. Indeed, problems have been identified, e.g., in Smalley and Kupka (1997) and in Samadi et al. (2006), and the discrepancy of the CM/CGM model predictions of the mean superadiabatic temperature gradient of the Sun with the now very well tested 3D LES (Nordlund et al. 2009), which can be seen when comparing Canuto and Mazzitelli (1991), Baturin and Mironova (1995) and Canuto et al. (1996), for instance, with the simulations of Rosenthal et al. (1999), is no longer considered acceptable. The dependence on a scale length still has to be specified and the latter has to be calibrated. Moreover, the locality of the model in the end can only be overcome by a more general, non-local approach.

#### Non-local models

A whole number of non-local models of stellar convection have been developed along the lines of the MLT model and we have just briefly summarized some of them in Sect. 3.2.4. By allowing for overshooting and a self-consistent (mathematically well defined) turbulent pressure they have become a tool to improve the modelling of stellar pulsation (see Houdek and Dupret 2015, for a review). Since this actually concerns a whole variety of convection models, it is more difficult to provide a concise overview. We just mention a few main properties which distinguish such models from their local siblings. Dimension, transformation invariance, symmetries, and realizability are usually not a problem for them. Concerning computability, the models are clearly more demanding, as they introduce extra non-linear differential equations which have to be added to those describing the mean structure of a star. But since the models they are used within depend on one spatial dimension only, affordability is usually not a problem, but rather, numerical stability in long-term calculations as required for stellar evolution. Non-local models of stellar convection thus remain used only for computing more detailed models of instantaneous stellar structure (to compute perturbations related to stellar oscillations, Houdek and Dupret 2015) or to follow the actual pulsation say of Cepheids in time dependent calculations over a number of pulsation periods (cf. the review of Buchler and Kolláth 2000). But how about the aspects of robustness, universality, falsifiability, and independence of model internal parameters from the objects being modelled? Originally, the prediction of where double-mode Cepheids appear in the Hertzsprung–Russell (HR) diagram in addition to the correct location of the red edge of the Cepheid instability strip has been announced as a success of this formalism. Smolec and Moskalik (2008) have published a critical study which demonstrated that the results for these objects obtained through the non-local models in use are very sensitive to the choice of parameters. In Mundprecht et al. (2015) the algebraic expression for the convective flux in the standard non-local convection models used in that field (Stellingwerf 1982; Kuhfuß 1986) was investigated by means of a 2D LES of a Cepheid (Mundprecht et al. 2013). It was concluded that one of the “constants” appearing in both models is anything but constant as a function of phase and position inside or outside the convection zone. Variations of more than a factor of 2 are at least necessary as a function of phase and also of position to find agreement with the simulations (Mundprecht et al. 2015). Thus, the criteria of robustness, universality, independence of parameter choice from the object being modelled and falsifiability are only somewhat better fulfilled by the most widely used non-local models than by, say, MLT. Clearly, there is much room and need for improvement.

#### Reynolds stress approach

**Fig. 15:**
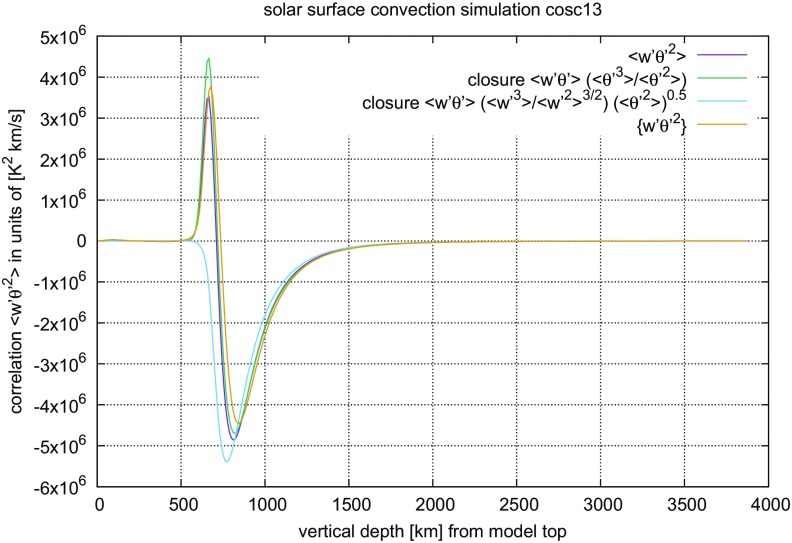
Correlation $\overline{w'\theta '^2}$ at the solar surface computed from a numerical simulation with ANTARES (Muthsam et al. 2010) (details on the simulation: Belkacem et al. 2015, in prep.) in comparison with two closure relations and the Favre average $\{w'\theta '^2\}=\overline{\rho w'\theta '^2}/{\overline{\rho }}$. For details see text and Sect. 3.3

**Fig. 16:**
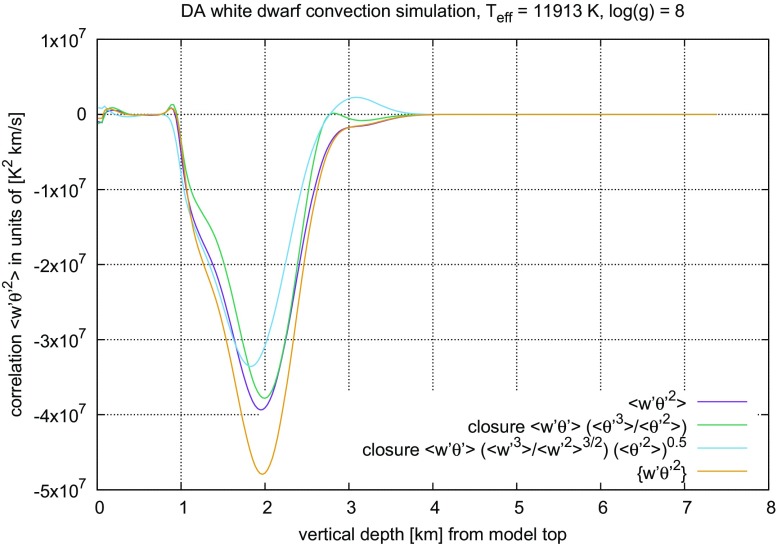
Correlation $\overline{w'\theta '^2}$ at the surface of a DA white dwarf computed from a numerical simulation with ANTARES (Muthsam et al. 2010) (details on the simulation: Kupka et al. 2017, submitted) in comparison with two closure relations and the Favre average $\{w'\theta '^2\}=\overline{\rho w'\theta '^2}/{\overline{\rho }}$. For details see text and Sect. 3.3

Full Reynolds stress models of convection are much more rarely used in solar and stellar astrophysics. The suite of models developed in Xiong (1978, 1985) and Xiong et al. (1997) has found applications to calculate mixing by overshooting in massive stars (Xiong 1986), in the envelope of B–F type stars (Xiong 1990), below the solar convection zone (Xiong and Deng 2001), and to compute pulsational stability (Xiong et al. 2015), among others. These models still require a mixing length but rather to compute the dissipation rate of kinetic energy as in Eq. (29). Canuto (1992, 1993, 1997a), Canuto and Dubovikov (1998), and Canuto (1999, 2011) suggested fully non-local Reynolds stress models which means that they avoid Eq. (29) but instead require the solution of a dynamical equation for the dissipation rate of turbulent kinetic energy, $\epsilon$. In this form it has so far been used only in a few cases in stellar astrophysics, essentially to study overshooting in A-type stars (Kupka and Montgomery 2002) and hot DA-type white dwarfs (Montgomery and Kupka 2004). Given that these models are considerably more complex than any of the previous ones, the demands with respect to dimension, transformation invariance, symmetries, and realizability become more stringent. As an example, consider the closure relation

105 $$\begin{aligned} \overline{w'\theta '^2} \approx \overline{w'\theta '} (\overline{\theta '^3}/\overline{\theta '^2}) \end{aligned}$$

suggested by Mironov et al. (1999) as opposed to

106 $$\begin{aligned} \overline{w'\theta '^2} \approx \overline{w'\theta '}\,\overline{\theta '^2}^{1/2} (\overline{w'^3}/\overline{w'^2}^{3/2}) \end{aligned}$$

proposed by various other authors and critically reviewed in Mironov et al. (1999). Whereas Eq. (105) is invariant under transformations $w' \rightarrow -w'$ and $\theta ' \rightarrow -\theta '$, this is not the case for Eq. (106). The latter hence does not respect the symmetry with respect to sign change which the correlation $\overline{w'\theta '^2}$ as well as Eq. (105) do. We compare these relations for both a solar simulation and the case of a white dwarf, as already discussed in Sects. 2 and 3, in Figs. 15 and 16. Although the coefficient on the right-hand side is just 1 for Eq. (105), the closure relation with input data taken from the numerical simulation reproduces $\overline{w'\theta '^2}$ as computed directly from the same simulation agrees quite well for both the solar and the white dwarf case. This is despite the simulations address very different physical situations. In comparison Eq. (106) predicts the wrong sign for the photosphere (upper overshooting) of the solar simulation and both upper (photospheric) and lower (beneath the convection zone) overshooting zones of the white dwarf simulation. This shows the importance of sign symmetry stressed in Mironov et al. (1999). It also demonstrates that *it is possible to derive closure relations which are fairly robust and universal with closure parameters derived totally independently of stellar astrophysics, hence yielding a falsifiable modelling approach which has already passed a whole number of tests.* Remarkably, compressibility effects are not such an issue here, as is demonstrated by the differences to the Favre average staying below 25% in the convective and overshooting zones for both cases. Clearly, a full Reynolds stress model is more expensive than any local model, but built into a 1D framework it is much more affordable than an LES, whether made for 2D or 3D geometry. As of now, a Reynolds stress model fulfilling all these conditions with respect to all its underlying closure hypotheses apparently has not been proposed yet. Taking into account the physical completeness (cf. Canuto 1999, 2011) this class of models allows for it seems worthwhile to pursue further research on them to have a better model available for, ideally, whenever full numerical simulations of stellar convection are not affordable.

#### 1D models derived from 3D simulations and related techniques

Three different approaches are currently used for trying to improve 1D modelling by means of (chiefly 3D) LES of stellar convection. We provide a short overview on them from the viewpoint of model properties similar to the above discussion on “genuine” 1D models.

*Calibrating 1D models by means of LES of stellar convection* So far these have almost exclusively focussed on calibrating the scale height parameter $\alpha$ of MLT or the related scale length in the local convection model of Canuto and Mazzitelli (1991) (CM model). Both had been calibrated in the work of Ludwig et al. (1999) on the basis of 2D LES for stellar surface convection, chiefly for spectral types F to K and main sequence stars as well as for giants in the same region of effective temperatures. Such an effort necessarily calibrates just one global quantity, for instance, the entropy of the outer part of the quasi-adiabatic convective envelope, which has also been subject to calibrations based on recent 3D LES such as Magic et al. (2015) and Tanner et al. (2016). A similar calibration has been provided in Tremblay et al. (2015) for DA white dwarfs. While this approach certainly has some merits in the sense that it avoids introducing other aspects of astrophysical modelling such as computing astrophysical observables from the models and then compare them to data as in empirical calibrations, e.g., of Alonso et al. (2000), Ferraro et al. (2006), and Wu et al. (2015), it does not correct any deficiency of the underlying model. Indeed, already from comparisons to earlier 2D LES (Schlattl et al. 1997) it was obvious that a *depth-dependent* calibration of $\alpha$ were needed to reproduce the temperature structure of the simulation and similar can be concluded from Fig. 1 in Tanner et al. (2016). Thus, while the lack of *robustness* is partially lifted, the other issues of MLT remain unchanged, including its falsifications via helioseismology and discrepancies revealed from comparisons to 3D LES. In Trampedach and Stein (2011) the physical interpretation of some of the concepts underlying MLT was investigated. While one can ascribe the typical length over which ascending fluid turns over and starts to descend to mass conservation and identify that as a property also revealed by the simulations, other concepts such as those of rising and ascending “bubbles” cannot (cf. Trampedach and Stein 2011). However, even if the meaning of *l* were revised this way, the approach of “calibrating the mixing length while keeping the rest of the model unaltered” can correct only one deficiency of such models. Hence, the performance of MLT with respect to the criteria list in Sect. 6.1.2 is not that much improved and the calibration of a physically more complete model of stellar convection which better performs in that respect from the very beginning would be more attractive for future work along this direction.

*Derive interpolated functions from 3D LES* The analysis of Trampedach and Stein (2011) and experience from earlier work (Trampedach et al. 1999) led Trampedach et al. (2014a, b) to suggest entire calibrated functions of physical properties as a function of optical depth such as temperature (Trampedach et al. 2014a) and turbulent pressure (Trampedach et al. 2014b). In their attempts to use these calibrations, Salaris and Cassisi (2015) mentioned that using the averaged simulation data directly may be necessary, if a more accurate description of the superadiabatic layer is needed. This is, however, not an option if a 3D LES consistent with the stellar parameters of interest (luminosity, radius, mass, etc.) is not available, hence fits of $T(\tau )$ (temperature as a function of optical depth) with universal parameters or parameters taken from tables provide a much wanted alternative. Although this approach on first sight does a much better job on the criteria listed in Sect. 6.1.2, physical quantities have to be considered case by case and matching them consistently to the interior stellar structure is a non-trivial issue. Magic (2016) has computed more accurate fit functions for the specific case of the entropy profile from comparing 3D LES for different stellar parameters. An inspection of Fig. 6 of Magic (2016) reveals notable differences in the temperature and pressure profiles while the profile of entropy is matched much better. However, this is not sufficient to construct a complete model of stellar structure which would be a prerequisite for use in stellar evolution modelling. It is also not obvious whether this provides any advantage over interpolating the basic variables (*T*, *p*, etc.) directly from a grid of 3D simulations and use the latter as “upper boundary conditions” moved into the convective zone while at the same time a detailed interpretation of seismological data is most likely requiring a dedicated 3D LES of the target object anyway. At the moment the advantages of the interpolation approach with respect to *robustness* and *universality* when compared to the MLT calibrations discussed in the previous paragraph appear not yet obvious, especially because discrepancies of any 1D model with respect to averages directly obtained from a 3D simulation remain.

*Direct usage of 3D LES results* The direct usage of numerical simulation results to applications in helioseismology in particular and thus also to problems in the physics of stellar interiors modelling was pioneered by Rosenthal et al. (1999) with their work on the near-surface effect. This way of using 3D simulations differs from what is described below in Sect. 6.3 in the sense that here the 3D simulation has to be consistently matched to a 1D model to compute the desired quantities such as solar oscillation frequencies. It has been revisited by Sonoi et al. (2015) who aimed at quantifying the difference between oscillation frequencies of (solar-like) p-modes for a set of 3D simulation models and by Ball et al. (2016). For the solar case the effect of magnetic fields was accounted for in the work of Magic and Weiss (2016). The main idea here is to shift the upper boundary of the stellar interiors computation inwards and use the 3D simulation data as boundary condition such that the 1D model is constructed consistently with respect to surface energy flux, gravity, and stellar radius. Hence, the 3D simulation can be “put on top of the 1D model”. It thus successfully combines the advantages of 1D models as used for the deep interior for the stars investigated with those of 3D simulations: the complete stellar structure model becomes computable while benefitting from the robustness and the large region of applicability of the simulations (with respect to universality, physical variability, and independence of model internal parameters). The success as also reported in Rosenthal et al. (1999) and in Magic and Weiss (2016), e.g., with respect to explaining the near-surface effect for the case of the Sun makes this approach particularly attractive. However, while affordable for a few target objects, it still does come at the expense of state-of-the-art 3D LES contrary to their 1D models constructed for the same objects. Thus, also for such applications it would be convenient if instead 1D models could be used that avoid at least some of the deficiencies of MLT-like models from the beginning.

We summarize the discussion of the current state of 1D models derived from or calibrated with 3D LES of stellar surface convection by a few general remarks. While this strand of convection modelling is readily applicable to the case of solar-like surface convection and thus to stars at the lower central main sequence (spectral types F to M) and the red giant branch as well as their equivalent among the cool DA type white dwarfs, it cannot provide any improvements related to convection in the deep interior, such as overshooting below the solar convection zone, the influence of rotation on mixing in the stellar interior, core convection, semi-convection, for example, and even for the case of surface convection in A-stars the approach has not yet been developed. For some of these problems more idealized numerical simulations have been made, but contrary to LES including the stellar surface they usually require extrapolations to the exact astrophysical parameter range or they lack sufficient resolution. The entire approach is necessarily restricted to *computable* cases with respect to the underlying numerical simulations and as soon as high accuracy is necessary such as in the applications of Rosenthal et al. (1999), Sonoi et al. (2015), Ball et al. (2016), or Magic and Weiss (2016), it can no longer support classical, local MLT. The latter has been falsified in this context anyway, not just with respect to the near-surface effect (Rosenthal et al. 1999), but also with respect to p-mode driving (cf. Samadi et al. 2006), where more advanced models are needed (Belkacem et al. 2006a, b; Samadi et al. 2008), as well as in the case of p-mode damping (see also Houdek and Dupret 2015). Rather, direct use of the 3D LES or their application together with non-local convection models is needed for such problems (cf. Belkacem et al. 2006a; Houdek and Dupret 2015). We expect that this strand of research releases its full potential once it is no longer seen as just a device to improve the classical, local models.

### Multidimensional models

With respect to the criteria mentioned in Sect. 6.1.2 the multidimensional simulations have the main advantage that their most important restrictions stem from *computability*. In spite of that they provide just models and a model of a convection zone is not the convection zone itself. Given that basic issue one may pose the question about scope and limitations of multidimensional modelling of stellar convection zones. The answer simply reflects the diversity of stellar convection zones. Solar granulation, the oldest and most successfully tackled item in multidimensional stellar convection investigations, can be modelled by now with a stunning degree of realism, see, e.g., the review in Nordlund et al. (2009). Staying with the Sun, modelling of the global convection zone leads to extreme challenges because of the diversity of spatial and temporal parameters. Hence the models, while interesting and indicative, have quite different a degree of realism than those for solar granulation (cf. Miesch 2005).

On the side of numerical astrophysics the task is therefore to provide the best tools for model construction. Naturally that has to be augmented by asking the best questions once the codes are at hand.

The numerical community has responded to those requests. As is evident from the list of codes provided in Sect. 5.6 with many of the entries being rather recent or under continuous development there is vivid activity ongoing. This activity comes with now full awareness of the importance of numerics of high quality which is implemented and developed further.

An emerging trend is a tendency of increasing *universality*. While originally (and probably even now) such codes preferentially emerged in response to questions within a specific field of astrophysics, generality of the underlying equations together with modularity facilitated by modern programming languages has opened up possibilities to render such codes useful in quite different areas of stellar physics or more general astrophysics. Indeed, a number of codes have already successfully been applied in a whole variety of fields.

That has, of course, to do with the inclusion of more than just pure hydrodynamics and microphysics for the situation under investigation. The most important aspects are

- magnetic fields (naturally)
- grid refinement for placing resolution where needed
- options for geometry (in particular sphericity).

These points are increasingly addressed. Of course, there are additional aspects such as radiative transfer for atmospheres (possibly even non-LTE line formation), moving grids for pulsating stars, two-component Navier–Stokes equations for semiconvection, and others.

Such endeavours are vigorously pursued, currently and without any question also in the future. So, what are presently true *branching points* in this area so that one cannot, at this time, predict future development?

We mainly see two aspects in multidimensional modelling techniques, namely the following ones:

- *Low Mach Number modelling* As is seen from the discussion in Sects. 4.3–4.6 on the one hand and Sect. 5.3 on the other there are now two competing approaches in computing low Mach number flows. They consist in either eliminating sound waves by modifying the basic equations themselves or in keeping them unharmful by properly designed numerical methods. As is evident from the discussion in Sect. 4.3 the first approach faces considerable difficulties concerning validity of the approximate equations in particular for situations for which they were not found applicable directly by derivation (but which are important astrophysically). The second, purely numerical approach does not face those difficulties, although one must hasten to say that it has been introduced in stellar convection modelling only quite recently. So, lack of difficulties may possibly and rather be lack of knowledge about difficulties, and extensive testing is needed. Ultimately, the two kinds of methods will have to be compared in terms of applicability and efficiency which will lead to survival of the fittest.
- *Hardware issues* The performance of traditional CPUs per computational core is more or less stalling by now. To a considerable extent the increase in available productivity on clusters as most often used in such a type of calculations come now, in the first instance, from increasing the number of CPUs in a cluster and, to some degree, from the larger number of computational cores now available in a single CPU. Yet, it is quite common that the wall clock times for high resolution computations as under consideration here amount to several months. In addition to resolution only, there may come strong demands on physical time in terms of model relaxation as more closely discussed in Sect. 2. Shortening the production cycle of models is therefore a task of major importance.
  With the demand for acceleration of computations in mind, resorting to graphics cards (or rather its graphics processing units, GPUs) or to specialized coprocessors may be an alternative. This alternative is already being recognized within the present computational community, e.g., in the CHOLLA code described in Schneider and Robertson (2015). That paper provides a rather detailed impression about what switching to graphics card computation may mean for basic numerics, algorithmics, and implementation. The ratio of the performance of a graphics card compared to a CPU can be impressive as evident from Fig. 12 of Schäfer et al. (2016) even if it should be kept in mind that the comparison is with *one* core of the CPU and, by the way, a smoothed particle hydrodynamics code is described there rather than one applicable in the present context.
  Surely, it remains to be seen whether this approach will prove convincing and widely used in the future. However, there may be profound implications for numerical methods and codes, if this eventually becomes mainstream, given the quite different properties of GPUs in terms of penalty for memory access or branching as opposed to their advantages in terms of raw computational performance.

### Final remarks

The present vibrant activity in the investigation, observationally and theoretically, of so many aspects of stellar physics needs in particular activity on the side of stellar convection modelling. The areas most frequently worked on at present include convection in atmospheres with detailed theoretical spectroscopy for investigating individual stars and stellar populations, excitation of stellar oscillations for diagnosing interiors of stars, investigations on overshooting and mixing for better models of stellar structure and evolution, supernova explosions, core convection in giants and late dwarfs (as opposed to the usual convection in shells), differential rotation and convection, etc. Or course, the numerous aspects of convection and magnetism (dynamo action, flux expulsion, $\ldots$) have to be kept in mind. As a consequence, researchers in stellar convection modelling face a rich variety of research questions allowing fruitful applications of their tools.

More recently, the scope of this kind of research broadens even more. Both, one- and multidimensional modelling face completely new needs and opportunities in the rapidly increasing research on extrasolar planets (interiors and atmospheres).

Multidimensional modelling offers particularly bright perspectives: while in earlier times programs with stellar convection in mind were quite specialized, many of the newer codes can be applied or expanded for much broader research, even for topics far away from stellar or planetary physics.

Consequently, the adept of multidimensional modelling may contribute to an impressive list of quite diverse areas. That may be considered an exciting opportunity in the age of increasing specialization.

## Footnotes

1 We point out that this term has been used by Zahn (1991) to describe the layer underneath the solar convection zone where radiative diffusion starts to operate faster than advection and thereby terminates “convective penetration” into stably stratified fluid. This is related to our own definition, but it also involves the interaction of a flow with diffusion, thus $L_\mathrm{t} > \delta _\mathrm{min}$.

2 The more detailed estimate of Zahn (1991) yields $L_\mathrm{t}\approx 1\,\mathrm{km}$ in his definition of a “thermal boundary layer of depth $L_\mathrm{t}$ to convective penetration below the solar convection zone”.

3 We have to emphasize here that extremely large Prandtl numbers in the Earth interior are restricted to the Earth mantle ($\mathrm{Pr} \sim 3.3\times 10^{23}$, see Vol. 7 in Schubert 2015) and to the inner core ($\mathrm{Pr} \sim 10^{16}$, see also Table 3 in Olson 2011). The outer core is characterized by relatively moderate Prandtl numbers ($\mathrm{Pr} \sim 0.1\ldots 0.5$, see also Table 2 in Olson 2011).

4 We note that Trampedach and Stein (2011) defined a “*mass mixing length*” which can be used to describe *how only a very small fraction of the mass in the upflows makes it from the interior and all the way up to the photosphere, simply due to mass conservation in a stratified atmosphere* (Sect. 2 of their paper). While their concept is useful to understand how mixing occurs over a well defined fraction (actually, multiple) of a pressure scale height that also coincides with the numerical length used in the standard MLT model, it refers to a different kind of length when compared to Eq. (29). This difference becomes evident as soon as stars with shallow convection zones and large overshooting regions are considered, such as A-type stars (Kupka and Montgomery 2002) or hot DA white dwarfs (cf. Fig. 4 in this review) where $l_\mathrm{m}$ is much smaller than a pressure scale height for the former while mass is readily transported over a multiple thereof. Thus, we do not consider their results about the mass mixing length as an incentive to continue using MLT, but rather as a step towards providing calibrations of 1D models based on 3D LES which we discuss in Sect. 6.2.4.

5 We note here that the closure for the stationary third order moments in Canuto (1993), derived under the assumption of the eddy-damped quasi normal approximation in that paper, does *not* depend on a *huge* number of “free parameters”, but only on the usual about a dozen or so that appear in Reynolds stress models. This notion has been based on a misunderstanding, since the apparent parameters (front factors of gradients of the second order moments) are the result of explicitly deriving an analytical solution in the stationary limit which depends only on the basic closure parameters of the Reynolds stress models and thus the “free parameters” are not free to choose at all, but fixed and only computed explicitly for a particular choice of closure parameters to provide some implementation convenience.

6 We note that in Canuto and Dubovikov (1997) a numerical solution by Antia of the model of Canuto (1992) assuming the downgradient approximation for third order moments and Eq. (29) for $\epsilon$ to compute the amount of overshooting below the solar convection is reported, but at this level the model is quite similar to that one of Xiong (1978, 1985) and not a fully non-local Reynolds stress model in the sense just defined.

7 This is again a point, where the Courant–Friedrichs–Lewy condition for explicit schemes pops up.

8 We have $\mathcal {A}\varDelta =\varDelta \mathcal {A}$. Namely,

$$\begin{aligned} h(\varDelta \mathcal {A}f)(x)=h(\mathcal {A}f(x+h/2)-\mathcal {A}f(x-h/2))=\int _{x}^{x+h}f(\xi )d\xi -\int _{x-h}^{x}f(\xi )d\xi , \end{aligned}$$

and similarly $h(\mathcal {A}\varDelta f)(x)$ leads to the same value. Multiplying $\mathcal {A}\varDelta =\varDelta \mathcal {A}$ from both sides with $\mathcal {A}^{-1}$ yields indeed $\mathcal {A}^{-1}\varDelta =\varDelta \mathcal {A}^{-1}$.

9
http://dedalus-project.org.

10
http://flash.uchicago.edu/site/flashcode.

11
https://geodynamics.org/cig/files/7214/2543/8678/Featherstone_cig_2015_bm_rayleigh_2.pdf.

12
https://ccse.lbl.gov/Research/MAESTRO/.

13
http://magic-sph.github.io.

14
http://pencil-code.nordita.org/doc.php.

15
https://geodynamics.org/cig/files/7214/2543/8678/Featherstone_cig_2015_bm_rayleigh_2.pdf.

16
http://comp.astro.ku.dk/Twiki/view/CompAstro/StaggerCode.

17
http://www.astro.ku.dk/~kg/Papers/MHD_code.ps.gz.
